# Supplementary material for: Ruthenium(II)-catalysed remote C–H alkylations as a versatile platform to meta-decorated arenes
Source: Nat Commun. 2017 Jun 9;8:15430. doi: 10.1038/ncomms15430 (PMC5472771; doi:10.1038/ncomms15430)
Supplement: Supplementary Information — Supplementary figures, supplementary tables, supplementary methods and supplementary references. [file ncomms15430-s1.pdf]

Supplementary Table 1: Optimization studies for the ruthenium(II)-catalyzed direct *meta*-alkylation of ketimine 1[a]

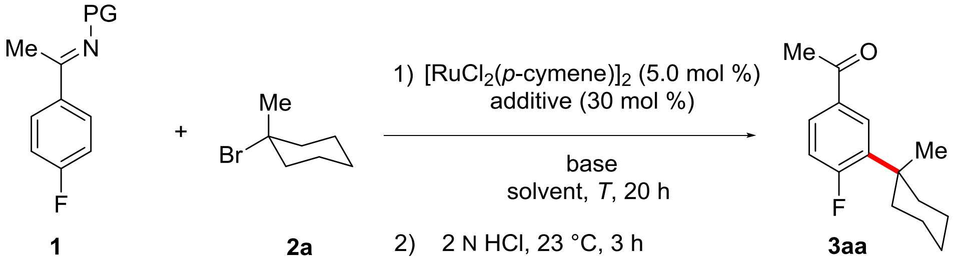

Entry	PG	Additive	Base	Solvent	T (°C)	Yield (%)
1	PMP	Piv-Val-OH	K_2CO_3	1,4-dioxane	120	22
2	PMP	$MesCO_2H$	K_2CO_3	1,4-dioxane	120	26[b]
3	PMP	1-$AdCO_2H$	K_2CO_3	1,4-dioxane	120	50
4	PMP	1-$AdCO_2H$	K_2CO_3	1,4-dioxane	100	0
5	PMP	1-$AdCO_2H$	K_2CO_3	1,4-dioxane	140	47
6	PMP	1-$AdCO_2H$	K_2CO_3	NMP	120	0
7	PMP	1-$AdCO_2H$	K_2CO_3	*o*-xylene	120	49
8	PMP	1-$AdCO_2H$	K_2CO_3	DME	120	36
9	PMP	1-$AdCO_2H$	K_2CO_3	PhMe	120	52
10	PMP	1-$AdCO_2H$	Na_2CO_3	PhMe	120	<5
11	PMP	1-$AdCO_2H$	Cs_2CO_3	PhMe	120	49
12	PMP	1-$AdCO_2H$	Ag_2CO_3	PhMe	120	0
13	PMP	-	KOAc	PhMe	120	30[b]
14	PMP	-	NaOAc	PhMe	120	<5
15	PMP	Piv-Val-OH	K_2CO_3	PhMe	120	30[b]
16	Bn	1-$AdCO_2H$	K_2CO_3	PhMe	120	30
17	TMP	1-$AdCO_2H$	K_2CO_3	PhMe	120	58
18	TMP	1-$AdCO_2H$	K_2CO_3	1,4-dioxane	120	87,[c] 66,[b] 52
19	TMP	Piv-Val-OH	K_2CO_3	PhMe	120	26,[b] 17
**20**	**TMP**	**1-$AdCO_2H$**	**K_2CO_3**	**$PhCMe_3$**	**120**	**73**
21	TMP	1-$AdCO_2H$	K_2CO_3	PhH	120	54
22	TMP	1-$AdCO_2H$	K_2CO_3	$PhCF_3$	120	52
23	TMP	-	K_2CO_3	$PhCMe_3$	120	0

Entry	PG	Additive	Base	Solvent	T (°C)	Yield (%)
24	TMP	1-AdCO$_2$H	K$_2$CO$_3$	PhCMe$_3$	120	73[d]
25	TMP	1-AdCO$_2$H	K$_2$CO$_3$	1,4-dioxane	120	51[d]
26	TMP	MesCO$_2$H	K$_2$CO$_3$	1,4-dioxane	120	30
27	TMP	AcOH	K$_2$CO$_3$	PhCMe$_3$	120	14
28	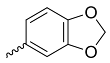	1-AdCO$_2$H	K$_2$CO$_3$	PhMe	120	51
29	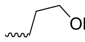	1-AdCO$_2$H	K$_2$CO$_3$	PhMe	120	50[b]
30	TMP	Boc-Val-OH	K$_2$CO$_3$	PhMe	120	26
31	TMP	Boc-Ile-OH	K$_2$CO$_3$	PhMe	120	28
32	TMP	Piv-Ile-OH	K$_2$CO$_3$	PhMe	120	33
**33**	**TMP**	**Piv-Ile-OH**	**K$_2$CO$_3$**	**PhCMe$_3$**	**120**	**64**
34	TMP	Piv-Ile-OH	K$_2$CO$_3$	PhCF$_3$	120	41
35	TMP	Piv-Ile-OH	K$_2$CO$_3$	PhCMe$_3$	120	0[e]
36	TMP	Piv-Ile-OH	K$_2$CO$_3$	PhCMe$_3$	120	0[f]
37	PMP	Piv-Ile-OH	K$_2$CO$_3$	PhCMe$_3$	120	44

[a] Reaction conditions: **1** (0.5 mmol), **2a** (1.5 mmol), [RuCl$_2$(*p*-cymene)]$_2$ (5.0 mol %), additive (30 mol %), base (1.0 mmol), solvent (2 mL), 20 h, under N$_2$. Yield of isolated products. [b] NMR yield with CH$_2$Br$_2$ as the internal standard. [c] GC-conversion. [d] [RuCl$_2$(benzene)]$_2$ (5.0 mol %) was used. [e] RuCl$_3$ (5.0 mol%) was used. [f] RuCl$_3$·3 H$_2$O (5.0 mol %) was used. PMP = 4-Methoxyphenyl, TMP = 3,4,5-Trimethoxyphenyl.

Supplementary Table 2: Probing of alkyl halides for the *meta*-C–H-alkylation of ketimine 1a

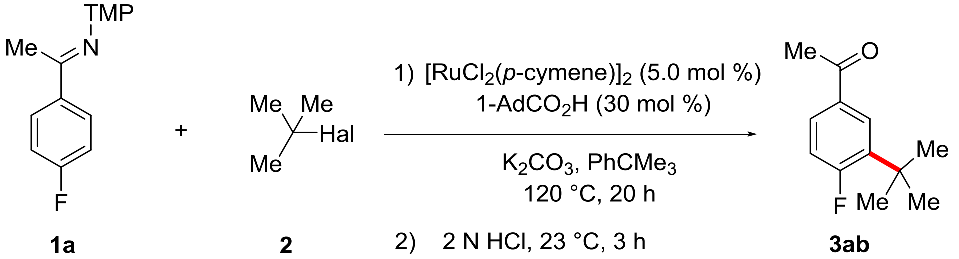

Entry	Hal	Yield(%)[a]
1	I	52
2	Br	74
3	Cl	72

[a] Reaction conditions: **1a** (0.5 mmol), **2** (1.5 mmol), K_2CO_3 (1.0 mmol), $PhCMe_3$ (2.0 mL), 120 °C, 20 h. Yield of isolated product.

Supplementary Methods

General Procedure A: Ruthenium(II)-catalyzed direct *meta*-C–H-alkylation of ketimines 1 using 1-AdCO$_2$H (4) as the ligand

Ketimine **1** (0.50 mmol), [RuCl$_2$(*p*-cymene)]$_2$ (15.3 mg, 25.0 µmol, 5.0 mol %), 1-AdCO$_2$H (**4**) (27.3 mg, 0.15 mmol, 30 mol %) and K$_2$CO$_3$ (138 mg, 1.00 mmol) were placed in a pre-dried 25 mL pressure tube. The tube was evacuated and purged with N$_2$ for three times. Alkyl bromide **2** (1.50 mmol) and PhCMe$_3$ (2.0 mL) were then added and the mixture was stirred at 120 °C for 20 h. At ambient temperature, HCl (2 N, 3.0 mL) was added, and the resulting mixture was stirred for additional 3 h, and then extracted with EtOAc or Et$_2$O (3 × 20 mL). The combined organic layers were dried over Na$_2$SO$_4$ and concentrated *in vacuo*. Purification of the residue by column chromatography (*n*-hexane/EtOAc or *n*-pentane/Et$_2$O) yielded phenone **3**.

General Procedure B: Ruthenium(II)-catalyzed direct *meta*-C–H-alkylation of ketimines 1 using Piv-Ile-OH (5) as the ligand

Ketimine **1** (0.50 mmol), [RuCl$_2$(*p*-cymene)]$_2$ (15.3 mg, 25.0 µmol, 5.0 mol %), Piv-Ile-OH (**5**) (32.3 mg, 0.15 mmol, 30 mol %) and K$_2$CO$_3$ (138 mg, 1.00 mmol) were placed in a pre-dried 25 mL pressure tube. The tube was evacuated and purged with N$_2$ for three times. Alkyl bromide **2** (1.50 mmol) and PhCMe$_3$ (2.0 mL) were then added and the mixture was stirred at 120 °C for 20 h. At ambient temperature, HCl (2 N, 3.0 mL) was added, and the resulting mixture was stirred for additional 3 h, and then extracted with EtOAc or Et$_2$O (3 × 20 mL). The combined organic layers were dried over Na$_2$SO$_4$ and concentrated *in vacuo*. Purification of the residue by column chromatography (*n*-hexane/EtOAc or *n*-pentane/Et$_2$O) yielded phenone **3**.

General Procedure C: Baeyer-Villiger oxidation of phenones

Phenone **3** (0.11 or 0.50 mmol) and *m*-CPBA (3 equiv.) were placed in a pre-dried 10 mL pressure tube and CH$_2$Cl$_2$ (2.0 mL) was added. The mixture was stirred at 60 °C for 12 h. Then, at ambient temperature, sat. aq. Na$_2$S$_2$O$_3$ and sat. aq. NaHCO$_3$ solutions (1:1, 20 mL) were added and the resulting mixture was stirred for additional 30 min. The aqueous layer was extracted with CH$_2$Cl$_2$ (3 × 20 mL) and the combined organic layers were concentrated *in vacuo*. To the crude mixture was added NaOH (2 M, 2.0 mL) and EtOH/water (3:1, 4 mL) and the mixture was stirred at ambient temperature for 3 h. Then HCl (1 N, 20 mL) and CH$_2$Cl$_2$ (20 mL) were added. The layers were separated and the aqueous phase was extracted with

CH_2Cl_2 (3 × 20 mL). The combined organic phase was dried over Na_2SO_4 and concentrated *in vacuo*. Purification of the residue by column chromatography (*n*-pentane/Et_2O) yielded phenol **13**.

Characterization Data

1-[(3-(*tert*-Butyl)phenyl]ethan-1-one (3bb)

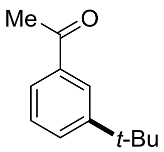

The general procedure **A** was followed using substrate **1b** (143 mg, 0.50 mmol) and bromide **2b** (206 mg, 1.50 mmol). After 20 h, purification by column chromatography (*n*-pentane/Et_2O 50:1) yielded **3bb** (63 mg, 71%) as a colorless oil.

The general procedure **B** was followed using substrate **1b** (143 mg, 0.50 mmol) and bromide **2b** (206 mg, 1.50 mmol). After 20 h, purification by column chromatography (*n*-pentane/Et_2O 50:1) yielded **3bb** (48 mg, 54%) as a colorless oil.

^{1}H-NMR (300 MHz, $CDCl_3$): δ = 7.99 (ddd, J = 2.1, 1.8, 0.5 Hz, 1H), 7.74 (ddd, J = 7.8, 1.8, 1.1 Hz, 1H), 7.59 (ddd, J = 7.8, 2.1, 1.1 Hz, 1H), 7.38 (ddd, J = 7.8, 7.8, 0.5 Hz, 1H), 2.59 (s, 3H), 1.34 (s, 9H).

^{13}C-NMR (125 MHz, $CDCl_3$): δ = 198.3 (C_q), 151.6 (C_q), 136.9 (C_q), 130.2 (CH), 128.2 (CH), 125.7 (CH), 124.8 (CH), 34.9 (C_q), 31.3 (CH_3), 26.8 (CH_3).

IR (ATR): $\tilde{\nu}$ = 2962, 2869, 1682, 1581, 1460, 1353, 1283, 967, 795 cm^{-1}.

MS (EI) *m*/*z* (relative intensity): 176 (21) $[M]^+$, 161 (100) $[M–Me]^+$, 133 (23) $[M–Ac]^+$, 115 (8).

HR-MS (EI): *m*/*z* calcd for $C_{12}H_{16}O^+$ $[M]^+$ 176.1196, found 176.1203.

The spectral data are in accordance with those reported in the literature.[6]

1-[3-(*tert*-Butyl)-4-fluorophenyl]ethan-1-one (3ab)

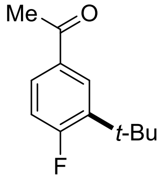

The general procedure **A** was followed using substrate **1a** (152 mg, 0.50 mmol) and bromide **2b** (206 mg, 1.50 mmol). After 20 h, purification by column chromatography (*n*-pentane/Et_2O 50:1) yielded **3ab** (72 mg, 74%) as a colorless oil.

The general procedure **B** was followed using substrate **1a** (152 mg, 0.50 mmol) and bromide **2b** (206 mg, 1.50 mmol). After 20 h, purification by column chromatography (*n*-pentane/Et_2O 50:1) yielded **3ab** (63 mg, 65%) as a colorless oil.

^{1}H-NMR (300 MHz, $CDCl_3$): δ = 7.95 (dd, J = 8.1, 2.3 Hz, 1H), 7.78 (ddd, J = 8.4, 4.5, 2.3 Hz, 1H), 7.04 (dd, J = 12.0, 8.4 Hz, 1H), 2.57 (s, 3H), 1.39 (d, J = 1.1 Hz, 9H).

^{13}C-NMR (125 MHz, $CDCl_3$): δ = 196.8 (C_q), 165.0 (d, $^1J_{C\text{-}F}$ = 257 Hz, C_q), 137.5 (d, $^2J_{C\text{-}F}$ = 12 Hz, C_q), 133.1 (d, $^4J_{C\text{-}F}$ = 3 Hz, C_q), 128.5 (d, $^3J_{C\text{-}F}$ = 10 Hz, CH), 127.9 (d, $^3J_{C\text{-}F}$ = 8 Hz, CH), 116.4 (d, $^2J_{C\text{-}F}$ = 25 Hz, CH), 34.5 (d, $^3J_{C\text{-}F}$ = 3 Hz, C_q), 29.8 (d, $^4J_{C\text{-}F}$ = 3 Hz, CH_3), 26.6 (CH_3).

^{19}F-NMR (376 MHz, $CDCl_3$): δ = (–101.5) – (–101.8) (m).

IR (ATR): $\tilde{\nu}$ = 2961, 2873, 1683, 1606, 1490, 1355, 1235, 1094, 817 cm^{-1}.

MS (EI) *m*/*z* (relative intensity): 194 (18) $[M]^+$, 179 (100) $[M–Me]^+$, 151 (58) $[M–Ac]^+$, 136 (10).

HR-MS (EI): *m*/*z* calcd for $C_{12}H_{15}FO^+$ $[M]^+$ 194.1101, found 194.1106.

1-[3-(*tert*-Butyl)-4-methoxyphenyl]ethan-1-one (3cb):

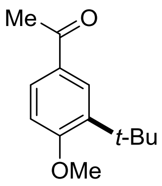

The general procedure **A** was followed using substrate **1c** (158 mg, 0.50 mmol) and bromide **2b** (206 mg, 1.50 mmol). After 20 h, purification by column chromatography (*n*-pentane/Et_2O 10:1) yielded **3cb** (84 mg, 81%) as a colorless oil.

The general procedure **B** was followed using substrate **1c** (158 mg, 0.50 mmol) and bromide **2b** (206 mg, 1.50 mmol). After 20 h, purification by column chromatography (*n*-pentane/Et_2O 10:1) yielded **3cb** (68 mg, 66%) as a colorless oil.

^{1}H-NMR (300 MHz, $CDCl_3$): δ = 7.92 (d, J = 2.3 Hz, 1H), 7.81 (dd, J = 8.5, 2.3 Hz, 1H), 6.87 (d, J = 8.5 Hz, 1H), 3.89 (s, 3H), 2.54 (s, 3H), 1.37 (s, 9H).

^{13}C-NMR (125 MHz, $CDCl_3$): δ = 197.1 (C_q), 162.4 (C_q), 138.2 (C_q), 129.7 (C_q), 128.5 (CH), 127.0 (CH), 110.6 (CH), 55.2 (CH_3), 35.0 (C_q), 29.6 (CH_3), 26.4 (CH_3).

IR (ATR): $\tilde{\nu}$ = 2958, 1674, 1595, 1495, 1457, 1357, 1237, 1182, 1026, 970 cm^{-1}.

MS (EI) *m*/*z* (relative intensity): 206 (36) $[M]^+$, 191 (100) $[M–Me]^+$, 163 (42) $[M–Ac]^+$, 133 (18).

HR-MS (EI): *m*/*z* calcd for $C_{13}H_{18}O_2^+$ $[M]^+$ 206.1301, found 206.1297.

The spectral data are in accordance with those reported in the literature.[7]

1-[3-(*tert*-Butyl)-4-chlorophenyl]ethan-1-one (3db):

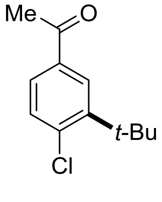

The general procedure **A** was followed using substrate **1d** (160 mg, 0.50 mmol) and bromide **2b** (206 mg, 1.50 mmol). After 20 h, purification by column chromatography (*n*-pentane/Et_2O 50:1) yielded **3db** (65 mg, 62%) as a

colorless oil.

The general procedure **B** was followed using substrate **1d** (160 mg, 0.50 mmol) and bromide **2b** (206 mg, 1.50 mmol). After 20 h, purification by column chromatography (*n*-pentane/Et_2O 50:1) yielded **3db** (69 mg, 65%) as a colorless oil.

^{1}H-NMR (300 MHz, $CDCl_3$): δ = 8.05 (d, *J* = 2.2 Hz, 1H), 7.69 (dd, *J* = 8.2, 2.2 Hz, 1H), 7.43 (d, *J* = 8.2 Hz, 1H), 2.59 (s, 3H), 1.51 (s, 9H).

^{13}C-NMR (125 MHz, $CDCl_3$): δ = 197.2 (C_q), 146.9 (C_q), 139.0 (C_q), 135.3 (C_q), 132.1 (CH), 127.6 (CH), 127.0 (CH), 36.3 (C_q), 29.5 (CH_3), 26.6 (CH_3).

IR (ATR): $\tilde{\nu}$ = 2964, 1684, 1588, 1353, 1233, 1038, 818, 529 cm^{-1}.

MS (EI) *m*/*z* (relative intensity): 210 (25) $[M]^+$, 195 (82) $[M–Me]^+$, 167 (100) $[M–Ac]^+$, 115 (26), 91 (13), 75 (9), 57 (10), 43 (81).

HR-MS (ESI): *m*/*z* calcd for $C_{12}H_{16}ClO^+$ $[M+H]^+$ 211.0884, found 211.0885.

1-[3-(1-Methylcyclohexyl)phenyl]ethan-1-one (3ba):

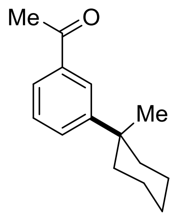

The general procedure **A** was followed using substrate **1b** (143 mg, 0.50 mmol) and bromide **2a** (266 mg, 1.50 mmol). After 20 h, purification by column chromatography (*n*-pentane/Et_2O 50:1) yielded **3ba** (65 mg, 60%) as a colorless oil.

The general procedure **B** was followed using substrate **1b** (143 mg, 0.50 mmol) and bromide **2a** (266 mg, 1.50 mmol). After 20 h, purification by column chromatography (*n*-pentane/Et_2O 50:1) yielded **3ba** (55 mg, 51%) as a colorless oil.

^{1}H-NMR (400 MHz, $CDCl_3$): δ = 7.98 (dd, *J* = 1.9, 1.9 Hz, 1H), 7.74 (ddd, *J* = 7.7, 1.7, 1.1 Hz, 1H), 7.57 (ddd, *J* = 7.7, 2.1, 1.1 Hz, 1H), 7.38 (ddd, *J* = 7.7, 7.7, 0.5 Hz, 1H), 2.58 (s, 3H), 2.06–1.94 (m, 2H), 1.66–1.49 (m, 4H), 1.49–1.32 (m, 4H), 1.18 (s, 3H).

^{13}C-NMR (100 MHz, $CDCl_3$): δ = 198.5 (C_q), 150.6 (C_q), 137.1 (C_q), 130.9 (CH), 128.4 (CH), 125.6 (CH), 125.6 (CH), 38.0 (C_q), 37.8 (CH_2), 30.3 (CH_3), 26.7 (CH_3), 26.2 (CH_2), 22.5 (CH_2).

IR (ATR): $\tilde{\nu}$ = 2925, 2856, 1682, 1597, 1425, 1356, 1256, 1196, 1080, 965 cm^{-1}.

MS (EI) *m*/*z* (relative intensity): 216 (40) $[M]^+$, 201 (75) $[M–Me]^+$, 173 (26) $[M–Ac]^+$, 160 (32), 145 (48).

HR-MS (EI): *m*/*z* calcd for $C_{15}H_{20}O^+$ $[M]^+$ 216.1509, found 216.1512.

1-[4-Fluoro-3-(1-methylcyclohexyl)phenyl]ethan-1-one (3aa)

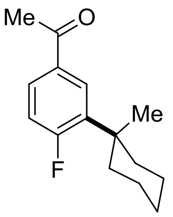

The general procedure **A** was followed using substrate **1a** (152 mg, 0.50 mmol) and bromide **2a** (266 mg, 1.50 mmol). After 20 h, purification by column chromatography (*n*-pentane/Et_2O 50:1) yielded **3aa** (85 mg, 73%) as a colorless oil.

The general procedure **B** was followed using substrate **1a** (152 mg, 0.50 mmol) and bromide **2a** (266 mg, 1.50 mmol). After 20 h, purification by column chromatography (*n*-pentane/Et_2O 50:1) yielded **3aa** (75 mg, 64%) as a colorless oil.

^{1}H-NMR (400 MHz, $CDCl_3$): δ = 7.97 (dd, J = 8.1, 2.3 Hz, 1H), 7.76 (ddd, J = 8.4, 4.5, 2.3 Hz, 1H), 7.02 (dd, J = 12.4, 8.4 Hz, 1H), 2.55 (s, 3H), 2.11–1.99 (m, 2H), 1.71–1.51 (m, 4H), 1.51–1.31 (m, 4H), 1.27 (d, J = 1.1 Hz, 3H).

^{13}C-NMR (100 MHz, $CDCl_3$): δ = 196.9 (C_q), 165.2 (d, $^1J_{C-F}$ = 257 Hz, C_q), 136.8 (d, $^2J_{C-F}$ = 12 Hz, C_q), 133.2 (d, $^4J_{C-F}$ = 3 Hz, C_q), 129.1 (d, $^3J_{C-F}$ = 8 Hz, CH), 128.3 (d, $^3J_{C-F}$ = 11 Hz, CH), 116.7 (d, $^2J_{C-F}$ = 26 Hz, CH), 37.9 (d, $^3J_{C-F}$ = 4 Hz, C_q), 37.0 (d, $^4J_{C-F}$ = 4 Hz, CH_2), 26.5 (CH_3), 26.4 (CH_3), 26.2 (CH_2), 22.5 (CH_2).

^{19}F-NMR (376 MHz, $CDCl_3$): δ = –101.0 (ddd, J = 12.7, 7.9, 4.6 Hz).

IR (ATR): $\tilde{\nu}$ = 2953, 2870, 1687, 1590, 1340, 1280, 1067, 830 cm^{-1}.

MS (EI) *m*/*z* (relative intensity): 234 (24) $[M]^+$, 219 (60) $[M–Me]^+$, 178 (35), 163 (62).

HR-MS (EI): *m*/*z* calcd for $C_{15}H_{19}FO^+$ $[M]^+$ 234.1414, found 234.1420.

1-[4-Fluoro-3-(1-methylcyclohexyl)phenyl]propan-1-one (3ea)

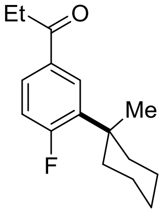

The general procedure **A** was followed using substrate **1e** (159 mg, 0.50 mmol) and bromide **2a** (266 mg, 1.50 mmol). After 20 h, purification by column chromatography (*n*-pentane/Et_2O 70:1) yielded **3ea** (87 mg, 70%) as a colorless oil.

The general procedure **B** was followed using substrate **1e** (159 mg, 0.50 mmol) and bromide **2a** (266 mg, 1.50 mmol). After 20 h, purification by column chromatography (*n*-pentane/Et_2O 70:1) yielded **3ea** (69 mg, 56%) as a colorless oil.

^{1}H-NMR (300 MHz, $CDCl_3$): δ = 7.98 (dd, J = 8.1, 2.3 Hz, 1H), 7.78 (ddd, J = 8.4, 4.5, 2.3 Hz, 1H), 7.02 (dd, J = 12.4, 8.4 Hz, 1H), 2.96 (q, J = 7.2 Hz, 2H), 2.14–1.99 (m, 2H), 1.73–1.51 (m, 4H), 1.51–1.32 (m, 4H), 1.28 (d, J = 1.0 Hz, 3H), 1.20 (t, J = 7.2 Hz, 3H).

^{13}C-NMR (125 MHz, $CDCl_3$): δ = 199.5 (C_q), 165.0 (d, $^1J_{C-F}$ = 256 Hz, C_q), 136.7 (d, $^2J_{C-F}$ = 11 Hz, C_q), 132.8 (d, $^4J_{C-F}$ = 3 Hz, C_q), 128.8 (d, $^3J_{C-F}$ = 8 Hz, CH), 127.7 (d, $^3J_{C-F}$ = 10 Hz,

CH), 116.7 (d, $^{2}J_{C\text{-}F}$ = 26 Hz, CH), 38.0 (d, $^{3}J_{C\text{-}F}$ = 4 Hz, C_q), 37.1 (d, $^{4}J_{C\text{-}F}$ = 4 Hz, CH_2), 31.7 (CH_2), 26.6 (CH_3), 26.4 (CH_2), 22.7 (CH_2), 8.4 (CH_3).

^{19}F-NMR (282 MHz, $CDCl_3$): δ = –101.4 (ddd, J = 12.7, 8.2, 4.8 Hz).

IR (ATR): $\tilde{\nu}$ = 2927, 2858, 1685, 1581, 1488, 1451, 1228, 1182, 799 cm^{-1}.

MS (EI) *m/z* (relative intensity): 248 (8) [M]$^+$, 219 (100) [M–Et]$^+$, 163 (22), 133 (12).

HR-MS (EI): *m/z* calcd for $C_{16}H_{21}FO^+$ [M]$^+$ 248.1571, found 248.1579.

1-[4-Fluoro-3-(*tert*-pentyl)phenyl]ethan-1-one (3ac)

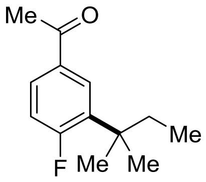

The general procedure **A** was followed using substrate **1a** (152 mg, 0.50 mmol) and bromide **2c** (227 mg, 1.50 mmol). After 20 h, purification by column chromatography (*n*-pentane/Et_2O 50:1) yielded **3ac** (79 mg, 76%) as a colorless oil.

The general procedure **B** was followed using substrate **1a** (152 mg, 0.50 mmol) and bromide **2c** (227 mg, 1.50 mmol). After 20 h, purification by column chromatography (*n*-hexane/EtOAc 50:1) yielded **3ac** (69 mg, 66%) as a colorless oil.

^{1}H-NMR (400 MHz, $CDCl_3$): δ = 7.89 (dd, J = 8.1, 2.3 Hz, 1H), 7.77 (ddd, J = 8.4, 4.5, 2.3 Hz, 1H), 7.01 (dd, J = 12.1, 8.4 Hz, 1H), 2.55 (s, 3H), 1.76 (qd, J = 7.5, 1.6 Hz, 2H), 1.34 (d, J = 1.2 Hz, 6H), 0.64 (td, J = 7.5, 0.6 Hz, 3H).

^{13}C-NMR (100 MHz, $CDCl_3$): δ = 196.9 (C_q), 164.9 (d, $^{1}J_{C\text{-}F}$ = 257 Hz, C_q), 136.0 (d, $^{2}J_{C\text{-}F}$ = 12 Hz, C_q), 133.1 (d, $^{4}J_{C\text{-}F}$ = 3 Hz, C_q), 129.1 (d, $^{3}J_{C\text{-}F}$ = 8 Hz, CH), 128.6 (d, $^{3}J_{C\text{-}F}$ = 10 Hz, CH), 116.3 (d, $^{2}J_{C\text{-}F}$ = 26 Hz, CH), 38.1 (d, $^{3}J_{C\text{-}F}$ = 3 Hz, C_q), 34.0 (d, $^{4}J_{C\text{-}F}$ = 4 Hz, CH_2), 27.6 (d, $^{4}J_{C\text{-}F}$ = 3 Hz, CH_3), 26.5 (CH_3), 9.3 (CH_3).

^{19}F-NMR (376 MHz, $CDCl_3$): δ = (–101.5) – (–101.7) (m).

IR (ATR): $\tilde{\nu}$ = 2965, 2877, 1683, 1604, 1491, 1355, 1252, 1094, 822 cm^{-1}.

MS (EI) *m/z* (relative intensity): 208 (7) [M]$^+$, 179 (100) [M–Et]$^+$, 151 (65), 136 (10).

HR-MS (EI): *m/z* calcd for $C_{13}H_{17}FO^+$ [M]$^+$ 208.1258, found 208.1266.

1-[3-(2-Methyl-4-phenylbutan-2-yl)phenyl]ethan-1-one (3ad)

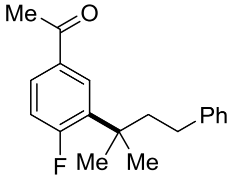

The general procedure **A** was followed using substrate **1a** (152 mg, 0.50 mmol) and bromide **2d** (340 mg, 1.50 mmol). After 20 h, purification by column chromatography (*n*-pentane/Et_2O 50:1) yielded **3ad** (84 mg, 59%) as a colorless oil.

The general procedure **B** was followed using substrate **1a** (152 mg, 0.50 mmol) and bromide **2d** (340 mg, 1.50 mmol). After 20 h, purification by column chromatography (*n*-pentane/Et_2O

50:1) yielded **3ad** (72 mg, 51%) as a colorless oil.

^{1}H-NMR (500 MHz, $CDCl_3$): δ = 7.96 (dd, J = 8.0, 2.3 Hz, 1H), 7.81 (ddd, J = 8.4, 4.5, 2.3 Hz, 1H), 7.24–7.19 (m, 2H), 7.15–7.10 (m, 1H), 7.10–7.04 (m, 3H), 2.59 (s, 3H), 2.34–2.28 (m, 2H), 2.10–2.04 (m, 2H), 1.45 (d, J = 1.0 Hz, 6H).

^{13}C-NMR (125 MHz, $CDCl_3$): δ = 197.0 (C_q), 164.9 (d, $^1J_{C-F}$ = 257 Hz, C_q), 142.5 (C_q), 135.7 (d, $^2J_{C-F}$ = 12 Hz, C_q), 133.2 (d, $^4J_{C-F}$ = 3 Hz, C_q), 129.0 (d, $^3J_{C-F}$ = 8 Hz, CH), 128.9 (d, $^3J_{C-F}$ = 10 Hz, CH), 128.3 (CH), 128.2 (CH), 125.6 (CH), 116.5 (d, $^2J_{C-F}$ = 26 Hz, CH), 43.6 (d, $^4J_{C-F}$ = 5 Hz, CH_2), 38.0 (d, $^3J_{C-F}$ = 3 Hz, C_q), 31.7 (CH_2), 28.2 (d, $^4J_{C-F}$ = 3 Hz, CH_3), 26.6 (CH_3).

^{19}F-NMR (470 MHz, $CDCl_3$): δ = (–101.5) – (–101.7) (m).

IR (ATR): $\tilde{\nu}$ = 3026, 2965, 1683, 1491, 1355, 1257, 1220, 823, 698 cm^{-1}.

MS (EI) m/z (relative intensity): 284 (14) $[M]^+$, 179 (100), 151 (53), 105 (36), 91 (43), 77 (9), 65 (11), 43 (42).

HR-MS (EI): m/z calcd for $C_{19}H_{21}FO^+$ $[M]^+$ 284.1571, found 284.1577.

1-[3-(5-Chloro-2-methylpentan-2-yl)-4-fluorophenyl]ethan-1-one (3ae)

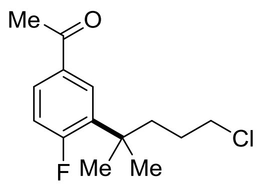

The general procedure **A** was followed using substrate **1a** (152 mg, 0.50 mmol) and bromide **2e** (299 mg, 1.50 mmol). After 20 h, purification by column chromatography (n-pentane/Et_2O 50:1) yielded **3ae** (71 mg, 55%) as a colorless oil.

The general procedure **B** was followed using substrate **1a** (152 mg, 0.50 mmol) and bromide **2e** (299 mg, 1.50 mmol). After 20 h, purification by column chromatography (n-pentane/Et_2O 50:1) yielded **3ae** (28 mg, 22%) as a colorless oil.

^{1}H-NMR (500 MHz, $CDCl_3$): δ = 7.89 (dd, J = 8.0, 2.3 Hz, 1H), 7.79 (ddd, J = 8.4, 4.5, 2.3 Hz, 1H), 7.05 (dd, J = 12.1, 8.4 Hz, 1H), 3.42 (t, J = 6.7 Hz, 2H), 2.56 (s, 3H), 1.90–1.84 (m, 2H), 1.52–1.44 (m, 2H), 1.39 (d, J = 1.0 Hz, 6H).

^{13}C-NMR (125 MHz, $CDCl_3$): δ = 196.9 (C_q), 164.8 (d, $^1J_{C-F}$ = 257 Hz, C_q), 135.4 (d, $^2J_{C-F}$ = 12 Hz, C_q), 133.2 (d, $^4J_{C-F}$ = 3 Hz, C_q), 128.9 (d, $^3J_{C-F}$ = 10 Hz, CH), 128.8 (d, $^3J_{C-F}$ = 8 Hz, CH), 116.5 (d, $^2J_{C-F}$ = 26 Hz, CH), 45.4 (CH_2), 38.8 (d, $^4J_{C-F}$ = 4 Hz, CH_2), 37.5 (d, $^3J_{C-F}$ = 3 Hz, C_q), 28.6 (CH_2), 28.1 (d, $^4J_{C-F}$ = 3 Hz, CH_3), 26.5 (CH_3).

^{19}F-NMR (470 MHz, $CDCl_3$): δ = (–101.6) – (–101.7) (m).

IR (ATR): $\tilde{\nu}$ = 2961, 2874, 1682, 1581, 1477, 1258, 1090, 822 cm^{-1}.

MS (EI) m/z (relative intensity): 258/256 (1/3) $[M]^+$, 179 (100), 151 (48), 115 (5).

HR-MS (ESI): m/z calcd for $C_{14}H_{19}FClO^+$ $[M+H]^+$ 257.1103, found 257.1103.

1-[3-(*tert*-Butyl)phenyl]propan-1-one (3fb)

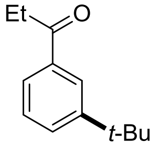

The general procedure **A** was followed using substrate **1f** (150 mg, 0.50 mmol) and bromide **2b** (206 mg, 1.50 mmol). After 20 h, purification by column chromatography (*n*-pentane/Et_2O 80:1) yielded **3fb** (76 mg, 80%) as a colorless oil.

The general procedure **B** was followed using substrate **1f** (150 mg, 0.50 mmol) and bromide **2b** (206 mg, 1.50 mmol). After 20 h, purification by column chromatography (*n*-pentane/Et_2O 80:1) yielded **3fb** (56 mg, 59%) as a colorless oil.

^{1}H-NMR (400 MHz, $CDCl_3$): δ = 8.02 (ddd, J = 2.0, 1.9, 0.5 Hz, 1H), 7.77 (ddd, J = 7.8, 1.9, 1.1 Hz, 1H), 7.59 (ddd, J = 7.8, 2.0, 1.1 Hz, 1H), 7.38 (ddd, J = 7.8, 7.8, 0.5 Hz, 1H), 3.01 (q, J = 7.3 Hz, 2H), 1.35 (s, 9H), 1.23 (t, J = 7.3 Hz, 3H).

^{13}C-NMR (100 MHz, $CDCl_3$): δ = 201.2 (C_q), 151.6 (C_q), 136.8 (C_q), 130.0 (CH), 128.2 (CH), 125.3 (CH), 124.7 (CH), 34.8 (C_q), 31.8 (CH_2), 31.2 (CH_3), 8.3 (CH_3).

IR (ATR): $\tilde{\nu}$ = 2963, 2872, 1685, 1581, 1459, 1364, 1209, 850 cm^{-1}.

MS (EI) *m/z* (relative intensity): 190 (6) $[M]^+$, 161 (100) $[M–Et]^+$, 133 (13), 115 (10).

HR-MS (ESI): *m/z* calcd for $C_{13}H_{19}O^+$ $[M+H]^+$ 191.1430, found 191.1436.

1-[3-(*tert*-Butyl)-4-fluorophenyl]propan-1-one (3eb):

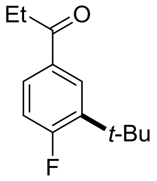

The general procedure **A** was followed using substrate **1e** (159 mg, 0.50 mmol) and bromide **2b** (206 mg, 1.50 mmol). After 20 h, purification by column chromatography (*n*-pentane/Et_2O 80:1) yielded **3eb** (86 mg, 83%) as a white solid.

The general procedure **B** was followed using substrate **1e** (159 mg, 0.50 mmol) and bromide **2b** (206 mg, 1.50 mmol). After 20 h, purification by column chromatography (*n*-pentane/Et_2O 80:1) yielded **3eb** (61 mg, 59%) as a white solid.

^{1}H-NMR (500 MHz, $CDCl_3$): δ = 7.96 (dd, J = 8.1, 2.3 Hz, 1H), 7.78 (ddd, J = 8.5, 4.5, 2.3 Hz, 1H), 7.03 (dd, J = 12.0, 8.5 Hz, 1H), 2.95 (q, J = 7.3 Hz, 2H), 1.38 (d, J = 1.2 Hz, 9H), 1.20 (t, J = 7.3 Hz, 3H).

^{13}C-NMR (125 MHz, $CDCl_3$): δ = 199.7 (C_q), 164.9 (d, $^1J_{C\text{-}F}$ = 257 Hz, C_q), 137.5 (d, $^2J_{C\text{-}F}$ = 12 Hz, C_q), 132.9 (d, $^4J_{C\text{-}F}$ = 3 Hz, C_q), 128.1 (d, $^3J_{C\text{-}F}$ = 10 Hz, CH), 127.7 (d, $^3J_{C\text{-}F}$ = 7 Hz, CH), 116.4 (d, $^2J_{C\text{-}F}$ = 25 Hz, CH), 34.4 (d, $^3J_{C\text{-}F}$ = 3 Hz, C_q), 31.7 (CH_2), 29.7 (d, $^4J_{C\text{-}F}$ = 4 Hz, CH_3), 8.3 (CH_3).

^{19}F-NMR (470 MHz, $CDCl_3$) δ = (–102.0) – (–102.1) (m).

IR (ATR): $\tilde{\nu}$ = 2945, 2022, 1686, 1605, 1458, 1366, 1210, 1089, 800 cm^{-1}.

m.p.: 44–45 °C.

MS (EI) *m*/*z* (relative intensity): 208 (7) $[M]^+$, 193 (14) $[M–Me]^+$, 179 (100) $[M–Et]^+$, 165 (22).

HR-MS (EI): *m*/*z* calcd for $C_{13}H_{17}FO^+$ $[M]^+$ 208.1258, found 208.1263.

1-[3-(*tert*-Butyl)phenyl]pentan-1-one (3gb):

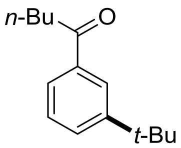

The general procedure **A** was followed using substrate **1g** (164 mg, 0.50 mmol) and bromide **2b** (206 mg, 1.50 mmol). After 20 h, purification by column chromatography (*n*-pentane/Et_2O 100:1) yielded **3gb** (81 mg, 74%) as a colorless oil.

The general procedure **B** was followed using substrate **1g** (164 mg, 0.50 mmol) and bromide **2b** (206 mg, 1.50 mmol). After 20 h, purification by column chromatography (*n*-pentane/Et_2O 100:1) yielded **3gb** (62 mg, 57%) as a colorless oil.

^{1}H-NMR (400 MHz, $CDCl_3$): δ = 8.02–7.99 (m, 1H), 7.76 (ddd, *J* = 7.8, 1.7, 1.1 Hz, 1H), 7.59 (ddd, *J* = 7.8, 2.1, 1.1 Hz, 1H), 7.39 (ddd, *J* = 7.8, 7.8, 0.5 Hz, 1H), 2.97 (dd, *J* = 7.7, 7.1 Hz, 2H), 1.77–1.68 (m, 2H), 1.47–1.37 (m, 2H), 1.35 (s, 9H), 0.96 (t, *J* = 7.3 Hz, 3H).

^{13}C-NMR (100 MHz, $CDCl_3$): δ = 200.9 (C_q), 151.6 (C_q), 136.9 (C_q), 130.0 (CH), 128.2 (CH), 125.4 (CH), 124.7 (CH), 38.4 (CH_2), 34.8 (C_q), 31.3 (CH_3), 26.6 (CH_2), 22.5 (CH_2), 13.9 (CH_3).

IR (ATR): $\tilde{\nu}$ = 2959, 2871, 1684, 1598, 1462, 1365, 1285, 1243, 1020, 793 cm^{-1}.

MS (EI) *m*/*z* (relative intensity): 218 (5) $[M]^+$, 203 (8) $[M–Me]^+$, 176 (32) $[M–Pr]^+$, 161 (100) $[M–Bu]^+$.

HR-MS (EI): *m*/*z* calcd for $C_{15}H_{22}O^+$ $[M]^+$ 218.1665, found 218.1682.

1-(3-Cycloheptylphenyl)ethan-1-one (3bf)

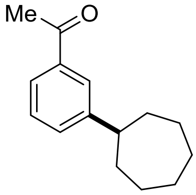

The general procedure **A** was followed using substrate **3b** (143 mg, 0.50 mmol) and bromide **2f** (266 mg, 1.50 mmol). After 20 h, purification by column chromatography (*n*-pentane/Et_2O 50:1) yielded **3bf** (75 mg, 69%) as a colorless oil.

^{1}H-NMR (300 MHz, $CDCl_3$): δ = 7.79–7.75 (m, 1H), 7.72 (ddd, *J* = 7.0, 1.8, 1.8 Hz, 1H), 7.40–7.30 (m, 2H), 2.77–2.65 (m, 1H), 2.57 (s, 3H), 1.95–1.45 (m, 12H).

^{13}C-NMR (125 MHz, $CDCl_3$): δ = 198.2 (C_q), 150.3 (C_q), 137.1 (C_q), 131.5 (CH), 128.4 (CH), 126.3 (CH), 125.7 (CH), 47.0 (CH), 36.7 (CH_2), 27.9 (CH_2), 27.2 (CH_2), 26.7 (CH_3).

IR (ATR): $\tilde{\nu}$ = 3352, 2921, 2853, 1681, 1582, 1434, 1356, 1270, 793 cm^{-1}.

MS (EI) *m*/*z* (relative intensity): 216 (60) $[M]^+$, 201 (100) $[M–Me]^+$, 146 (36), 131 (64).

HR-MS (EI): *m*/*z* calcd for $C_{15}H_{20}O^+$ $[M]^+$ 216.1509, found 216.1510.

1-(3-Cycloheptyl-4-methylphenyl)ethan-1-one (3hf)

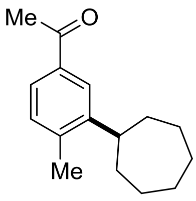

The general procedure **A** was followed using substrate **1h** (150 mg, 0.50 mmol) and bromide **2f** (266 mg, 1.50 mmol). After 20 h, purification by column chromatography (*n*-pentane/Et_2O 50:1) yielded **3hf** (82 mg, 71%) as a colorless oil.

^{1}H-NMR (300 MHz, $CDCl_3$): δ = 7.81 (d, *J* = 1.8 Hz, 1H), 7.63 (dd, *J* = 7.9, 1.8 Hz, 1H), 7.18 (d, *J* = 7.9 Hz, 1H), 2.93–2.81 (m, 1H), 2.55 (s, 3H), 2.36 (s, 3H), 1.88–1.76 (m, 4H), 1.76–1.44 (m, 8H).

^{13}C-NMR (75 MHz, $CDCl_3$): δ = 198.1 (C_q), 148.3 (C_q), 140.4 (C_q), 135.4 (C_q), 130.2 (CH), 125.5 (CH), 125.4 (CH), 41.8 (CH), 35.9 (CH_2), 27.6 (CH_2), 27.5 (CH_2), 26.5 (CH_3), 19.7 (CH_3).

IR (ATR): $\tilde{\nu}$ = 2920, 2853, 1678, 1602, 1444, 1353, 1242, 813 cm^{-1}.

MS (EI) *m*/*z* (relative intensity): 230 (42) $[M]^+$, 215 (100) $[M–Me]^+$, 145 (40), 115 (18).

HR-MS (EI): *m*/*z* calcd for $C_{16}H_{22}O^+$ $[M]^+$ 230.1665, found 230.1673.

1-(3-Cycloheptyl-4-methoxyphenyl)ethan-1-one (3cf)

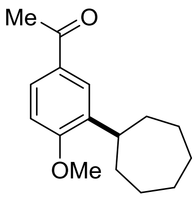

The general procedure **A** was followed using substrate **1c** (158 mg, 0.50 mmol) and bromide **2f** (266 mg, 1.50 mmol). After 20 h, purification by column chromatography (*n*-pentane/Et_2O 10:1) yielded **3cf** (100 mg, 81%) as a colorless oil.

^{1}H-NMR (300 MHz, $CDCl_3$): δ = 7.81 (d, *J* = 2.3 Hz, 1H), 7.76 (dd, *J* = 8.5, 2.3 Hz, 1H), 6.82 (d, *J* = 8.5 Hz, 1H), 3.86 (s, 3H), 3.15–3.00 (m, 1H), 2.52 (s, 3H), 1.91–1.43 (m, 12H).

^{13}C-NMR (75 MHz, $CDCl_3$): δ = 197.1 (C_q), 160.2 (C_q), 138.2 (C_q), 130.0 (C_q), 127.9 (CH), 127.1 (CH), 109.5 (CH), 55.5 (CH_3), 38.9 (CH), 35.2 (CH_2), 27.8 (CH_2), 27.4 (CH_2), 26.2 (CH_3).

IR (ATR): $\tilde{\nu}$ = 2919, 2852, 1672, 1596, 1495, 1354, 1241, 1025, 810 cm^{-1}.

MS (EI) *m*/*z* (relative intensity): 246 (95) $[M]^+$, 231 (100) $[M–Me]^+$, 161 (57), 147 (26).

HR-MS (EI): *m*/*z* calcd for $C_{16}H_{22}O_2^+$ $[M]^+$ 246.1614, found 246.1630.

1-[3-Cycloheptyl-4-(trifluoromethyl)phenyl]ethan-1-one (3if)

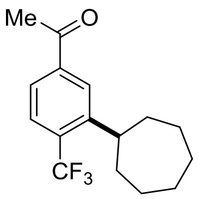

The general procedure **A** was followed using substrate **1i** (177 mg, 0.50 mmol) and bromide **2f** (266 mg, 1.50 mmol). After 20 h, purification by column chromatography (*n*-pentane/Et_2O 50:1) yielded **3if** (84 mg, 59%) as a colorless oil.

^{1}H-NMR (400 MHz, $CDCl_3$): δ = 7.99 (s, 1H), 7.78 (dq, J = 8.2, 0.8 Hz, 1H), 7.68 (d, J = 8.2 Hz, 1H), 3.15–3.06 (m, 1H), 2.63 (s, 3H), 1.91–1.78 (m, 4H), 1.78–1.51 (m, 8H).

^{13}C-NMR (100 MHz, $CDCl_3$): δ = 197.4 (C_q), 149.9 (q, $^3J_{C\text{-}F}$ = 2 Hz, C_q), 139.8 (C_q), 130.7 (q, $^2J_{C\text{-}F}$ = 30 Hz, C_q), 127.8 (CH), 125.9 (q, $^3J_{C\text{-}F}$ = 6 Hz, CH), 125.2 (CH), 124.1 (q, $^1J_{C\text{-}F}$ = 273 Hz, C_q), 41.6 (q, $^4J_{C\text{-}F}$ = 2 Hz, CH), 36.9 (CH_2), 27.5 (CH_2), 27.4 (CH_2), 26.8 (CH_3).

^{19}F-NMR (376 MHz, $CDCl_3$): δ = –59.5 (s).

IR (ATR): $\tilde{\nu}$ = 2925, 2856, 1692, 1574, 1415, 1310, 1238, 1154, 1035, 829 cm^{-1}.

MS (EI) *m/z* (relative intensity): 284 (35) $[M]^+$, 214 (55), 199 (100), 151 (23).

HR-MS (ESI): *m/z* calcd for $C_{16}H_{19}F_3NaO^+$ $[M+Na]^+$ 307.1280, found 307.1286.

1-(2-Cycloheptyl-[1,1'-biphenyl]-4-yl)ethan-1-one (3jf)

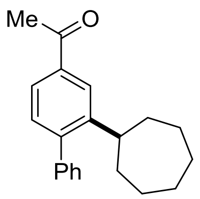

The general procedure **A** was followed using substrate **1j** (181 mg, 0.50 mmol) and bromide **2f** (266 mg, 1.50 mmol). After 20 h, purification by column chromatography (*n*-pentane/Et_2O 50:1) yielded **3jf** (92 mg, 63%) as a colorless oil.

^{1}H-NMR (300 MHz, $CDCl_3$): δ = 7.95 (d, J = 1.8 Hz, 1H), 7.75 (dd, J = 8.0, 1.8 Hz, 1H), 7.46–7.32 (m, 3H), 7.28–7.22 (m, 3H), 2.89–2.77 (m, 1H), 2.62 (s, 3H), 1.85–1.61 (m, 6H), 1.57–1.46 (m, 4H), 1.39–1.23 (m, 2H).

^{13}C-NMR (125 MHz, $CDCl_3$): δ = 197.9 (C_q), 147.9 (C_q), 145.2 (C_q), 140.9 (C_q), 136.4 (C_q), 130.0 (CH), 128.8 (CH), 128.0 (CH), 127.1 (CH), 126.3 (CH), 125.1 (CH), 41.6 (CH), 36.8 (CH_2), 27.7 (CH_2), 27.3 (CH_2), 26.7 (CH_3).

IR (ATR): $\tilde{\nu}$ = 2919, 2852, 1681, 1597, 1458, 1353, 1277, 1008, 827 cm^{-1}.

MS (EI) *m/z* (relative intensity): 292 (85) $[M]^+$, 221 (46), 165 (41), 115 (6).

HR-MS (ESI): *m/z* calcd for $C_{21}H_{25}O^+$ $[M+H]^+$ 293.1900, found 293.1905.

Methyl 4-acetyl-2-cycloheptylbenzoate (3kf)

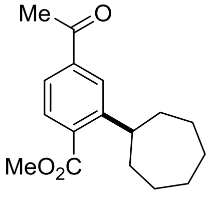

The general procedure **A** was followed using substrate **1k** (172 mg, 0.50 mmol) and bromide **2f** (266 mg, 1.50 mmol). After 20 h, purification by column chromatography (*n*-pentane/Et_2O 10:1) yielded **3kf** (98 mg,

71%) as a colorless oil.

^{1}H-NMR (300 MHz, $CDCl_3$): δ = 7.94 (dd, J = 1.2, 1.2 Hz, 1H), 7.76–7.72 (m, 2H), 3.93 (s, 3H), 3.40 (tt, J = 10.3, 3.2 Hz, 1H), 2.62 (s, 3H), 1.99–1.49 (m, 12H).

^{13}C-NMR (125 MHz, $CDCl_3$): δ = 197.6 (C_q), 168.1 (C_q), 150.8 (C_q), 139.1 (C_q), 133.4 (C_q), 129.6 (CH), 126.8 (CH), 125.0 (CH), 52.3 (CH_3), 42.1 (CH), 36.8 (CH_2), 27.8 (CH_2), 27.6 (CH_2), 26.9 (CH_3).

IR (ATR): $\tilde{\nu}$ = 2921, 2854, 1723, 1686, 1433, 1270, 1233, 1096, 1065, 785 cm^{-1}.

MS (EI) *m*/*z* (relative intensity): 274 (28) $[M]^+$, 259 (17) $[M–Me]^+$, 243 (73), 199 (34), 181 (28), 115 (23), 59 (18), 43 (100).

HR-MS (ESI): *m*/*z* calcd for $C_{17}H_{23}O_3^+$ $[M+H]^+$ 275.1642, found 275.1646.

1-(3-Cycloheptylphenyl)propan-1-one (3ff)

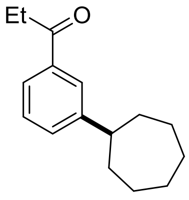

The general procedure **A** was followed using substrate **1f** (150 mg, 0.50 mmol) and bromide **2f** (266 mg, 1.50 mmol). After 20 h, purification by column chromatography (*n*-pentane/Et_2O 80:1) yielded **3ff** (68 mg, 59%) as a colorless oil.

^{1}H-NMR (300 MHz, $CDCl_3$): δ = 7.81–7.77 (m, 1H), 7.77–7.72 (m, 1H), 7.41–7.30 (m, 2H), 2.99 (q, J = 7.3 Hz, 2H), 2.78–2.66 (m, 1H), 1.97–1.45 (m, 12H), 1.22 (t, J = 7.3 Hz, 3H).

^{13}C-NMR (125 MHz, $CDCl_3$): δ = 201.0 (C_q), 150.3 (C_q), 136.9 (C_q), 131.3 (CH), 128.4 (CH), 126.1 (CH), 125.3 (CH), 47.0 (CH), 36.7 (CH_2), 31.8 (CH_2), 27.9 (CH_2), 27.2 (CH_2), 8.4 (CH_3).

IR (ATR): $\tilde{\nu}$ = 3391, 2921, 2853, 1683, 1582, 1482, 1348, 1233, 1161, 781 cm^{-1}.

MS (EI) *m*/*z* (relative intensity): 230 (5) $[M]^+$, 201 (100) $[M–Et]^+$, 179 (13), 131 (8).

HR-MS (ESI): *m*/*z* calcd for $C_{16}H_{23}O^+$ $[M+H]^+$ 231.1743, found 231.1749.

1-(3-Cyclopentyl-4-fluorophenyl)ethan-1-one (3ag)

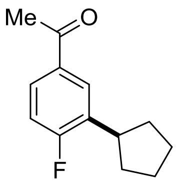

The general procedure **A** was followed using substrate **1a** (152 mg, 0.50 mmol) and bromide **2g** (224 mg, 1.50 mmol). After 20 h, purification by column chromatography (*n*-pentane/Et_2O 50:1) yielded **3ag** (73 mg, 71%) as a colorless oil.

^{1}H-NMR (300 MHz, $CDCl_3$): δ = 7.90 (dd, J = 7.3, 2.3 Hz, 1H), 7.76 (ddd, J = 8.5, 4.9, 2.3 Hz, 1H), 7.04 (dd, J = 9.9, 8.5 Hz, 1H), 3.32–3.16 (m, 1H), 2.57 (d, J = 0.5 Hz, 3H), 2.14–1.97 (m, 2H), 1.91–1.52 (m, 6H).

^{13}C-NMR (75 MHz, $CDCl_3$): δ = 196.6 (C_q), 164.0 (d, $^1J_{C\text{-}F}$ = 253 Hz, C_q), 133.4 (d, $^2J_{C\text{-}F}$ = 16 Hz, C_q), 133.3 (d, $^4J_{C\text{-}F}$ = 3 Hz, C_q), 128.5 (d, $^3J_{C\text{-}F}$ = 7 Hz, CH), 128.0 (d, $^3J_{C\text{-}F}$ = 10 Hz, CH), 115.3 (d, $^2J_{C\text{-}F}$ = 24 Hz, CH), 38.8 (d, $^3J_{C\text{-}F}$ = 1 Hz, CH), 33.1 (d, $^4J_{C\text{-}F}$ = 1 Hz, CH_2), 26.5 (CH_3), 25.4 (CH_2).

^{19}F-NMR (282 MHz, $CDCl_3$): δ = –109.7 (ddd, J = 9.9, 7.3, 5.0 Hz).

IR (ATR): $\tilde{\nu}$ = 3348, 2954, 2871, 1682, 1585, 1492, 1356, 1250, 1112, 822 cm^{-1}.

MS (EI) *m/z* (relative intensity): 206 (23) $[M]^+$, 191 (100) $[M\text{–}Me]^+$, 163 (16), 149 (20).

HR-MS (EI): *m/z* calcd for $C_{13}H_{15}FO^+$ $[M]^+$ 206.1101, found 206.1112.

1-(3-Cyclohexyl-4-fluorophenyl)ethan-1-one (3ah)

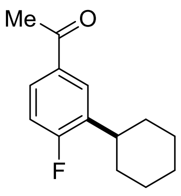

The general procedure **A** was followed using substrate **1a** (152 mg, 0.50 mmol) and bromide **2h** (245 mg, 1.50 mmol). After 20 h, purification by column chromatography (*n*-pentane/Et_2O 50:1) yielded **3ah** (76 mg, 69%) as a colorless oil

^{1}H-NMR (300 MHz, $CDCl_3$): δ = 7.88 (dd, J = 7.2, 2.3 Hz, 1H), 7.77 (ddd, J = 8.5, 4.9, 2.3 Hz, 1H), 7.05 (dd, J = 9.9, 8.5 Hz, 1H), 2.94–2.81 (m, 1H), 2.57 (s, 3H), 1.90–1.71 (m, 5H), 1.57–1.18 (m, 5H).

^{13}C-NMR (125 MHz, $CDCl_3$): δ = 196.7 (C_q), 163.5 (d, $^1J_{C\text{-}F}$ = 253 Hz, C_q), 134.9 (d, $^2J_{C\text{-}F}$ = 16 Hz, C_q), 133.4 (d, $^4J_{C\text{-}F}$ = 3 Hz, C_q), 128.3 (d, $^3J_{C\text{-}F}$ = 7 Hz, CH), 128.0 (d, $^3J_{C\text{-}F}$ = 10 Hz, CH), 115.3 (d, $^2J_{C\text{-}F}$ = 24 Hz, CH), 37.2 (d, $^3J_{C\text{-}F}$ = 2 Hz, CH), 32.9 (CH_2), 26.8 (CH_2), 26.6 (CH_3), 26.1 (CH_2).

^{19}F-NMR (282 MHz, $CDCl_3$): δ = –111.6 (ddd, J = 9.9, 7.2, 4.9 Hz).

IR (ATR): $\tilde{\nu}$ = 2926, 2852, 1682, 1586, 1492, 1355, 1254, 1107, 820 cm^{-1}.

MS (EI) *m/z* (relative intensity): 220 (23) $[M]^+$, 205 (100) $[M\text{–}Me]^+$, 149 (23), 109 (12).

HR-MS (EI): *m/z* calcd for $C_{14}H_{17}FO^+$ $[M]^+$ 220.1258, found 220.1262.

1-(3-Cycloheptyl-4-fluorophenyl)ethan-1-one (3af)

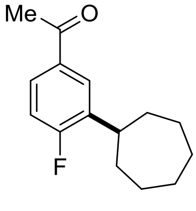

The general procedure **A** was followed using substrate **1a** (152 mg, 0.50 mmol) and bromide **2f** (266 mg, 1.50 mmol). After 20 h, purification by column chromatography (*n*-pentane/Et_2O 50:1) yielded **3af** (95 mg, 81%) as a colorless oil.

For gram-scale reaction: The general procedure **A** was followed using ketimine **1a** (1.00 g, 3.30 mmol), bromide **2f** (1.36 mL, 9.90 mmol), $[RuCl_2(p\text{-cymene})]_2$ (101 mg, 0.16 mmol), 1-$AdCO_2H$ (178 mg, 0.99 mmol) and K_2CO_3 (911 mg, 6.59 mmol) in $PhCMe_3$ (13 mL).

Purification by column chromatography (*n*-pentane/Et_2O 50:1) yielded **3af** (665 mg, 86%) as a colorless oil.

^{1}H-NMR (300 MHz, $CDCl_3$): δ = 7.86 (dd, J = 7.3, 2.3 Hz, 1H), 7.73 (ddd, J = 8.5, 4.9, 2.3 Hz, 1H), 7.02 (dd, J = 9.8, 8.5 Hz, 1H), 3.06–2.92 (m, 1H), 2.55 (s, 3H), 1.93–1.44 (m, 12H).

^{13}C-NMR (75 MHz, $CDCl_3$): δ = 196.9 (C_q), 163.1 (d, $^{1}J_{C\text{-}F}$ = 253 Hz, C_q), 136.8 (d, $^{2}J_{C\text{-}F}$ = 16 Hz, C_q), 133.5 (d, $^{4}J_{C\text{-}F}$ = 3 Hz, C_q), 128.6 (d, $^{3}J_{C\text{-}F}$ = 7 Hz, CH), 127.9 (d, $^{3}J_{C\text{-}F}$ = 10 Hz, CH), 115.4 (d, $^{2}J_{C\text{-}F}$ = 24 Hz, CH), 39.5 (d, $^{3}J_{C\text{-}F}$ = 1 Hz, CH), 35.2 (d, $^{4}J_{C\text{-}F}$ = 1 Hz, CH_2), 27.7 (CH_2), 27.2 (CH_2), 26.5 (CH_3).

^{19}F-NMR (282 MHz, $CDCl_3$): δ = (–111.0) – (–111.1) (m).

IR (ATR): $\tilde{\nu}$ = 2921, 1682, 1585, 1492, 1416, 1355, 1243, 1170, 1104, 819 cm^{-1}.

MS (EI) *m*/*z* (relative intensity): 234 (41) $[M]^+$, 219 (100) $[M–Me]^+$, 164 (40), 149 (70).

HR-MS (EI): *m*/*z* calcd for $C_{15}H_{19}FO^+$ $[M]^+$ 234.1414, found 234.1416.

1-(3-Cyclooctyl-4-fluorophenyl)ethan-1-one (3ai)

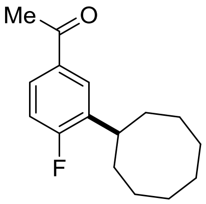

The general procedure **A** was followed using substrate **1a** (152 mg, 0.50 mmol) and bromide **2i** (287 mg, 1.50 mmol). After 20 h, purification by column chromatography (*n*-pentane/Et_2O 50:1) yielded **3ai** (83 mg, 67%) as a colorless oil.

^{1}H-NMR (300 MHz, $CDCl_3$): δ = 7.87 (dd, J = 7.3, 2.3 Hz, 1H), 7.75 (ddd, J = 8.5, 4.9, 2.3 Hz, 1H), 7.04 (dd, J = 9.9, 8.5 Hz, 1H), 3.20–3.05 (m, 1H), 2.57 (s, 3H), 1.90–1.73 (m, 6H), 1.73–1.50 (m, 8H).

^{13}C-NMR (125 MHz, $CDCl_3$): δ = 196.7 (C_q), 163.1 (d, $^{1}J_{C\text{-}F}$ = 253 Hz, C_q), 137.2 (d, $^{2}J_{C\text{-}F}$ = 16 Hz, C_q), 133.4 (d, $^{4}J_{C\text{-}F}$ = 3 Hz, C_q), 128.8 (d, $^{3}J_{C\text{-}F}$ = 7 Hz, CH), 127.8 (d, $^{3}J_{C\text{-}F}$ = 10 Hz, CH), 115.4 (d, $^{2}J_{C\text{-}F}$ = 24 Hz, CH), 37.3 (CH), 33.4 (CH_2), 26.7 (CH_2), 26.6 (CH_3), 26.4 (CH_2), 26.0 (CH_2).

^{19}F-NMR (282 MHz, $CDCl_3$): δ = (–110.5) – (–110.7) (m).

IR (ATR): $\tilde{\nu}$ = 2919, 2852, 1682, 1585, 1492, 1355, 1283, 1108, 822 cm^{-1}.

MS (EI) *m*/*z* (relative intensity): 248 (47) $[M]^+$, 233 (38) $[M–Me]^+$, 164 (69), 149 (100).

HR-MS (ESI): *m*/*z* calcd for $C_{16}H_{22}FO^+$ $[M+H]^+$ 249.1649, found 249.1654.

1-(3-Cycloheptyl-4-fluorophenyl)propan-1-one (3ef)

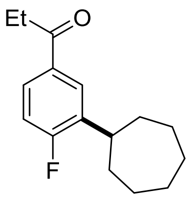

The general procedure **A** was followed using substrate **1e** (159 mg, 0.50 mmol) and bromide **2f** (266 mg, 1.50 mmol). After 20 h, purification by column chromatography (*n*-pentane/Et_2O 80:1) yielded **3ef** (97 mg, 78%) as a colorless oil.

^{1}H-NMR (300 MHz, $CDCl_3$): δ = 7.86 (dd, J = 7.3, 2.3 Hz, 1H), 7.74 (ddd, J = 8.5, 4.9, 2.3 Hz, 1H), 7.01 (dd, J = 9.9, 8.5 Hz, 1H), 3.05–2.89 (m, 3H), 1.92–1.45 (m, 12H), 1.19 (t, J = 7.2 Hz, 3H).

^{13}C-NMR (125 MHz, $CDCl_3$): δ = 199.4 (C_q), 162.8 (d, $^1J_{C\text{-}F}$ = 252 Hz, C_q), 136.6 (d, $^2J_{C\text{-}F}$ = 16 Hz, C_q), 133.2 (d, $^4J_{C\text{-}F}$ = 3 Hz, C_q), 128.3 (d, $^3J_{C\text{-}F}$ = 7 Hz, CH), 127.3 (d, $^3J_{C\text{-}F}$ = 10 Hz, CH), 115.3 (d, $^2J_{C\text{-}F}$ = 24 Hz, CH), 39.5 (CH), 35.3 (CH_2), 31.7 (CH_2), 27.8 (CH_2), 27.3 (CH_2), 8.4 (CH_3).

^{19}F-NMR (282 MHz, $CDCl_3$): δ = (–111.4) – (–111.6) (m).

IR (ATR): $\tilde{\nu}$ = 2923, 2855, 1685, 1586, 1492, 1350, 1237, 1150, 797 cm^{-1}.

MS (EI) *m*/*z* (relative intensity): 248 (6) $[M]^+$, 219 (100) $[M–Et]^+$, 149 (10), 109 (13).

HR-MS (ESI): *m*/*z* calcd for $C_{16}H_{22}FO^+$ $[M+H]^+$ 249.1649, found 249.1654.

1-(4-Cycloheptylnaphthalen-2-yl)ethan-1-one (3lf)

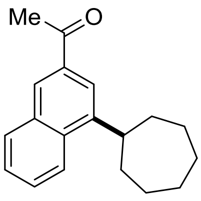

The general procedure **A** was followed using substrate **1l** (168 mg, 0.50 mmol) and bromide **2f** (266 mg, 1.50 mmol). After 20 h, purification by column chromatography (*n*-pentane/Et_2O 50:1) yielded **3lf** (118 mg, 89%) as a light yellow oil.

^{1}H-NMR (300 MHz, $CDCl_3$): δ = 8.29 (br s, 1H), 8.13 (d, J = 8.5 Hz, 1H), 7.99–7.93 (m, 2H), 7.67–7.59 (m, 1H), 7.57–7.49 (m, 1H), 3.55–3.41 (m, 1H), 2.72 (d, J = 1.5 Hz, 3H), 2.14–1.99 (m, 2H), 1.99–1.56 (m, 10H).

^{13}C-NMR (125 MHz, $CDCl_3$): δ = 198.2 (C_q), 146.5 (C_q), 134.0 (C_q), 133.4 (C_q), 133.0 (C_q), 130.5 (CH), 128.5 (CH), 128.1 (CH), 126.0 (CH), 123.4 (CH), 120.3 (CH), 41.1 (CH), 36.3 (CH_2), 27.9 (CH_2), 27.7 (CH_2), 26.6 (CH_3).

IR (ATR): $\tilde{\nu}$ = 2919, 2852, 1674, 1457, 1397, 1260, 1194, 885 cm^{-1}.

MS (EI) *m*/*z* (relative intensity): 266 (100) $[M]^+$, 209 (16), 183 (28), 153 (40).

HR-MS (EI): *m*/*z* calcd for $C_{19}H_{22}O^+$ $[M]^+$ 266.1665, found 266.1661.

1-(4-Isopropylnaphthalen-2-yl)ethan-1-one (3lj)

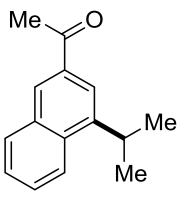

The general procedure **A** was followed using substrate **1l** (168 mg, 0.50 mmol) and bromide **2j** (185 mg, 1.50 mmol). After 20 h, purification by column chromatography (*n*-pentane/Et_2O 50:1) yielded **3lj** (89 mg, 84%) as a white solid.

^{1}H-NMR (300 MHz, $CDCl_3$): δ = 8.30 (br s, 1H), 8.13 (d, J = 8.5 Hz, 1H), 7.99 (d, J = 1.7 Hz, 1H), 7.97 (dd, J = 8.1, 1.2 Hz, 1H), 7.62 (ddd, J = 8.5, 6.8, 1.2 Hz, 1H), 7.52 (ddd, J = 8.1, 6.8, 1.2 Hz, 1H), 3.73 (hept, J = 6.9 Hz, 1H), 2.71 (s, 3H), 1.42 (d, J = 6.9 Hz, 6H).

^{13}C-NMR (125 MHz, $CDCl_3$): δ = 198.2 (C_q), 145.3 (C_q), 134.0 (C_q), 133.6 (C_q), 132.9 (C_q), 130.4 (CH), 128.7 (CH), 128.2 (CH), 126.0 (CH), 123.3 (CH), 119.4 (CH), 28.7 (CH), 26.6 (CH_3), 23.4 (CH_3).

IR (ATR): $\tilde{\nu}$ = 3063, 2960, 1671, 1397, 1271, 1229, 1194, 1142, 882 cm^{-1}.

m.p.: 60–62 °C.

MS (EI) *m*/*z* (relative intensity): 212 (58) $[M]^+$, 197 (100) $[M–Me]^+$, 152 (25), 115 (8).

HR-MS (EI): *m*/*z* calcd for $C_{15}H_{16}O^+$ $[M]^+$ 212.1196, found 212.1209.

1-[4-(*sec*-Butyl)naphthalen-2-yl]ethan-1-one (3lk)

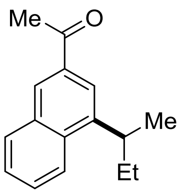

The general procedure **A** was followed using substrate **1l** (168 mg, 0.50 mmol) and bromide **2k** (206 mg, 1.50 mmol). After 20 h, purification by column chromatography (*n*-pentane/Et_2O 50:1) yielded **3lk** (88 mg, 78%) as a colorless oil.

^{1}H-NMR (300 MHz, $CDCl_3$): δ = 8.30 (d, J = 1.5 Hz, 1H), 8.13 (d, J = 8.5 Hz, 1H), 7.99–7.92 (m, 2H), 7.61 (ddd, J = 8.5, 6.8, 1.5 Hz, 1H), 7.52 (ddd, J = 8.0, 6.8, 1.2 Hz, 1H), 3.50 (dt, J = 6.9, 6.9 Hz, 1H), 2.71 (s, 3H), 1.96–1.81 (m, 1H), 1.81–1.65 (m, 1H), 1.39 (d, J = 6.9 Hz, 3H), 0.92 (t, J = 7.4 Hz, 3H).

^{13}C-NMR (125 MHz, $CDCl_3$): δ = 198.2 (C_q), 144.5 (C_q), 134.1 (C_q), 134.0 (C_q), 133.0 (C_q), 130.4 (CH), 128.6 (CH), 128.1 (CH), 126.0 (CH), 123.3 (CH), 120.1 (CH), 35.5 (CH), 30.5 (CH_2), 26.6 (CH_3), 21.1 (CH_3), 12.3 (CH_3).

IR (ATR): $\tilde{\nu}$ = 3056, 2961, 1674, 1622, 1425, 1396, 1278, 1174, 885 cm^{-1}.

MS (EI) *m*/*z* (relative intensity): 226 (52) $[M]^+$, 197 (100) $[M–Et]^+$, 153 (25), 127 (10).

HR-MS (EI): *m*/*z* calcd for $C_{16}H_{18}O^+$ $[M]^+$ 226.1352, found 226.1365.

1-[4-(Pentan-2-yl)naphthalen-2-yl]ethan-1-one (3ll)

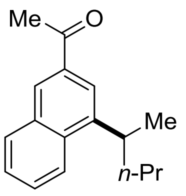

The general procedure **A** was followed using substrate **1l** (168 mg, 0.50 mmol) and bromide **2l** (227 mg, 1.50 mmol). After 20 h, purification by column chromatography (*n*-pentane/Et_2O 50:1) yielded **3ll** (92 mg, 77%) as a light yellow oil.

^{1}H-NMR (300 MHz, $CDCl_3$): δ = 8.29 (d, J = 1.5 Hz, 1H), 8.14 (d, J = 8.6 Hz, 1H), 7.98–7.93 (m, 2H), 7.61 (ddd, J = 8.6, 6.8, 1.5 Hz, 1H), 7.52 (ddd, J = 8.0, 6.8, 1.2 Hz, 1H), 3.59 (dt, J = 6.9, 6.9 Hz, 1H), 2.72 (s, 3H), 1.89–1.59 (m, 2H), 1.46–1.23 (m, 5H), 0.90 (t, J = 7.3 Hz, 3H).

^{13}C-NMR (125 MHz, $CDCl_3$): δ = 198.2 (C_q), 144.8 (C_q), 134.0 (C_q), 134.0 (C_q), 133.0 (C_q), 130.4 (CH), 128.6 (CH), 128.1 (CH), 126.0 (CH), 123.2 (CH), 120.2 (CH), 40.0 (CH_2), 33.6 (CH), 26.6 (CH_3), 21.6 (CH_3), 20.9 (CH_2), 14.3 (CH_3).

IR (ATR): $\tilde{\nu}$ = 2957, 2928, 1675, 1623, 1453, 1375, 1277, 1194, 885 cm^{-1}.

MS (EI) *m*/*z* (relative intensity): 240 (53) $[M]^+$, 197 (100) $[M–Pr]^+$, 153 (26), 127 (11).

HR-MS (EI): *m*/*z* calcd for $C_{17}H_{20}O^+$ $[M]^+$ 240.1509, found 240.1523.

1-(4-(Octan-2-yl)naphthalen-2-yl)ethan-1-one (3lm)

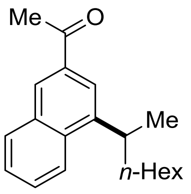

The general procedure **A** was followed using substrate **1l** (168 mg, 0.50 mmol) and bromide **2m** (290 mg, 1.50 mmol). After 20 h, purification by column chromatography (*n*-pentane/Et_2O 50:1) yielded **3lm** (111 mg, 79%) as a colorless oil.

^{1}H-NMR (300 MHz, $CDCl_3$): δ = 8.30 (s, 1H), 8.14 (d, J = 8.5 Hz, 1H), 7.99–7.92 (m, 2H), 7.61 (ddd, J = 8.5, 6.8, 1.5 Hz, 1H), 7.52 (ddd, J = 8.0, 6.8, 1.2 Hz, 1H), 3.57 (dt, J = 6.9, 6.9 Hz, 1H), 2.72 (s, 3H), 1.91–1.77 (m, 1H), 1.77–1.62 (m, 1H), 1.39 (d, J = 6.9 Hz, 3H), 1.35–1.14 (m, 8H), 0.90–0.73 (m, 3H).

^{13}C-NMR (125 MHz, $CDCl_3$): δ = 198.1 (C_q), 144.8 (C_q), 134.0 (C_q), 134.0 (C_q), 133.0 (C_q), 130.4 (CH), 128.6 (CH), 128.1 (CH), 126.0 (CH), 123.2 (CH), 120.1 (CH), 37.8 (CH_2), 33.9 (CH), 31.8 (CH_2), 29.5 (CH_2), 27.8 (CH_2), 26.6 (CH_3), 22.7 (CH_2), 21.6 (CH_3), 14.1 (CH_3).

IR (ATR): $\tilde{\nu}$ = 2956, 2954, 1677, 1454, 1352, 1276, 1195, 885 cm^{-1}.

MS (EI) *m*/*z* (relative intensity): 282 (50) $[M]^+$, 191 (100), 153 (22), 127 (5).

HR-MS (EI): *m*/*z* calcd for $C_{20}H_{26}O^+$ $[M]^+$ 282.1978, found 282.1994.

1-(4-Chloro-3-cycloheptylphenyl)ethan-1-one (3df)

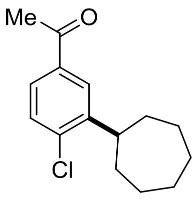

The general procedure **A** was followed using substrate **1d** (160 mg, 0.50 mmol) and bromide **2f** (266 mg, 1.50 mmol). After 20 h, purification by column chromatography (*n*-pentane/Et_2O 50:1) yielded **3df** (68 mg, 54%) as a colorless oil.

^{1}H-NMR (300 MHz, $CDCl_3$): δ = 7.87 (dd, J = 2.2, 0.4 Hz, 1H), 7.66 (dd, J = 8.3, 2.2 Hz, 1H), 7.41 (dd, J = 8.3, 0.4 Hz, 1H), 3.27–3.14 (m, 1H), 2.58 (s, 3H), 1.98–1.52 (m, 12H).

^{13}C-NMR (125 MHz, $CDCl_3$): δ = 197.1 (C_q), 147.2 (C_q), 138.2 (C_q), 135.8 (C_q), 129.5 (CH), 127.2 (CH), 126.5 (CH), 42.3 (CH), 35.4 (CH_2), 27.8 (CH_2), 27.4 (CH_2), 26.6 (CH_3).

IR (ATR): $\tilde{\nu}$ = 2922, 2854, 1684, 1591, 1406, 1355, 1235, 1038, 815, 523 cm^{-1}.

MS (EI) *m*/*z* (relative intensity): 250 (48) $[M]^+$, 235 (81) $[M–Me]^+$, 215 (44) $[M–Cl]^+$, 180 (41), 165 (73), 115 (27), 55 (23), 43 (100).

HR-MS (EI): *m*/*z* calcd for $C_{15}H_{19}ClO^+$ $[M]^+$ 250.1119, found 250.1118.

4-Acetyl-2-cycloheptylphenyl benzoate (3mf)

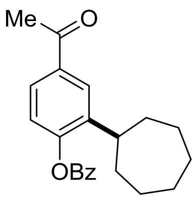

The general procedure **A** was followed using substrate **1m** (203 mg, 0.50 mmol) and bromide **2f** (266 mg, 1.50 mmol). After 20 h, purification by column chromatography (*n*-hexane/EtOAc 95:5) yielded **3mf** (88 mg, 52%) as a colorless oil.

^{1}H-NMR (400 MHz, $CDCl_3$): δ = 8.25–8.20 (m, 2H), 7.98 (d, J = 2.2 Hz, 1H), 7.83 (dd, J = 8.4, 2.2 Hz, 1H), 7.71–7.65 (m, 1H), 7.58–7.52 (m, 2H), 7.23 (d, J = 8.4 Hz, 1H), 2.92 (tt, J = 10.5, 3.4 Hz, 1H), 2.62 (s, 3H), 1.95–1.87 (m, 2H), 1.83–1.39 (m, 10H).

^{13}C-NMR (100 MHz, $CDCl_3$): δ = 197.3 (C_q), 164.8 (C_q), 151.7 (C_q), 141.9 (C_q), 135.2 (C_q), 133.9 (CH), 130.2 (CH), 129.1 (C_q), 128.7 (CH), 127.9 (CH), 127.0 (CH), 122.7 (CH), 40.2 (CH), 35.4 (CH_2), 27.6 (CH_2), 27.4 (CH_2), 26.6 (CH_3).

IR (ATR): $\tilde{\nu}$ = 2922, 1737, 1683, 1600, 1451, 1238, 1081, 1056, 1023, 707 cm^{-1}.

MS (EI) *m*/*z* (relative intensity): 336 (2) $[M]^+$, 231 (10), 135 (15), 105 (100), 77 (35), 51 (5), 43 (13).

HR-MS (ESI): *m*/*z* calcd for $C_{22}H_{24}O_3Na^+$ $[M+Na]^+$ 359.1618, found 359.1622.

4-Acetyl-2-cycloheptylphenyl 4-dimethylaminobenzoate (3nf)

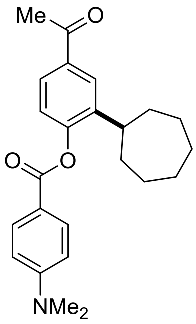

The general procedure **A** was followed using substrate **1n** (221 mg, 0.50 mmol) and bromide **2f** (266 mg, 1.50 mmol). After 20 h, purification by column chromatography (*n*-hexane/EtOAc 8:1) yielded **3nf** (121 mg, 64%) as a colorless solid.

^{1}H-NMR (300 MHz, $CDCl_3$): δ = 8.08 (d, J = 8.8 Hz, 2H), 7.95 (d, J = 2.1 Hz, 1H), 7.81 (dd, J = 8.4, 2.1 Hz, 1H), 7.22 (d, J = 8.4 Hz, 1H), 6.73 (d, J = 8.8 Hz, 2H), 3.10 (s, 6H), 2.99–2.88 (m, 1H), 2.61 (s, 3H), 1.96–1.85 (m, 2H), 1.82–1.39 (m, 10H).

^{13}C-NMR (125 MHz, $CDCl_3$): δ = 197.7 (C_q), 165.2 (C_q), 154.0 (C_q), 152.4 (C_q), 142.2 (C_q), 134.8 (C_q), 132.2 (CH), 127.9 (CH), 127.1 (CH), 123.1 (CH), 115.5 (C_q), 111.0 (CH), 40.4 (CH), 40.2 (CH_3), 35.5 (CH_2), 27.9 (CH_2), 27.6 (CH_2), 26.8 (CH_3).

IR (ATR): $\tilde{\nu}$ = 2917, 1706, 1687, 1600, 1276, 1235, 1161, 1074, 1056, 761 cm^{-1}.

m.p.: 166 °C.

MS (ESI) *m*/*z* (relative intensity): 402 (100) $[M+Na]^+$, 781 (76) $[2M+Na]^+$, 1160 (53) $[3M+Na]^+$.

HR-MS (ESI): *m*/*z* calcd for $C_{24}H_{29}O_3Na^+$ $[M+Na]^+$ 402.2040, found 402.2034.

Cholesterol 4-acetyl-2-cycloheptylbenzoate (3of)

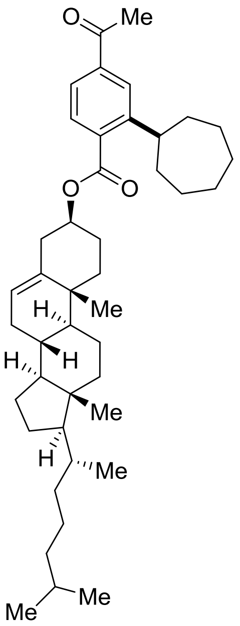

The general procedure **A** was followed using substrate **1o** (175 mg, 0.25 mmol) and bromide **2f** (133 mg, 0.75 mmol). After 20 h, purification by column chromatography (*n*-hexane/EtOAc 20:1) yielded **3of** (89 mg, 57%) as a colorless solid.

^{1}H-NMR (400 MHz, $CDCl_3$): δ = 7.92 (d, J = 1.7 Hz, 1H), 7.74 (dd, J = 8.1, 1.7 Hz, 1H), 7.68 (dd, J = 8.1, 0.4 Hz, 1H), 5.45 (d, J = 5.2 Hz, 1H), 4.94–4.84 (m, 1H), 3.36 (tt, J = 10.6, 3.2 Hz, 1H), 2.61 (s, 3H), 2.52–2.42 (m, 2H), 2.06–1.78 (m, 9H), 1.76–0.95 (m, 32H), 0.92 (d, J = 6.6 Hz, 3H), 0.87 (dd, J = 6.6, 1.8 Hz, 6H), 0.69 (s, 3H).

^{13}C-NMR (100 MHz, $CDCl_3$): δ = 198.0 (C_q), 167.8 (C_q), 150.3 (C_q), 139.6 (C_q), 139.1 (C_q), 134.8 (C_q), 129.6 (CH), 127.0 (CH), 125.3 (CH), 123.2 (CH), 75.4 (CH), 56.9 (CH), 56.3 (CH), 50.2 (CH), 42.5 (C_q), 42.3 (CH), 39.9 (CH_2), 39.7 (CH_2), 38.3 (CH_2), 37.2 (CH_2), 36.9 (CH_2), 36.8 (C_q), 36.4 (CH_2), 36.0 (CH), 32.1 (CH_2), 32.1 (CH), 28.4 (CH_2), 28.2 (CH), 28.0 (CH_2), 27.9 (CH_2), 27.7

(CH_2), 27.0 (CH_3), 24.5 (CH_2), 24.0 (CH_2), 23.0 (CH_3), 22.7 (CH_3), 21.2 (CH_2), 19.5 (CH_3), 18.9 (CH_3).

IR (ATR): $\tilde{\nu}$ = 2931, 2850, 1718, 1690, 1464, 1276, 1234, 1143, 1099, 1060 cm^{-1}.

m.p.: 151 °C.

MS (ESI) *m/z* (relative intensity): 651 (100) $[M+Na]^+$, 1281 (39) $[2M+Na]^+$.

HR-MS (ESI): *m/z* calcd for $C_{43}H_{64}O_3Na^+$ $[M+Na]^+$ 651.4748, found 651.4718.

1-(3-Cycloheptyl-4-morpholinophenyl)ethan-1-one (3pf)

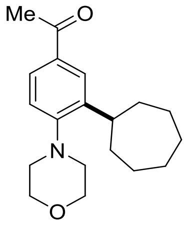

The general procedure **A** was followed using substrate **1p** (186 mg, 0.50 mmol) and bromide **2f** (266 mg, 1.50 mmol). After 20 h, to the reaction mixture was added HCl (2 N, 3.0 mL) and the resulting mixture was stirred for additional 3 h, and then neutralized with sat. aq. $NaHCO_3$ solution until pH 8. The reaction mixture was extracted with Et_2O (3 × 20 mL). The combined organic layers were dried over Na_2SO_4 and concentrated *in vacuo*. Purification of the residue by column chromatography (*n*-hexane/EtOAc 4:1) yielded **3pf** (88 mg, 58%) as a yellow oil.

^{1}H-NMR (300 MHz, $CDCl_3$): δ = 7.85 (d, J = 2.2 Hz, 1H), 7.73 (dd, J = 8.3, 2.2 Hz, 1H), 7.06 (d, J = 8.3 Hz, 1H), 3.93–3.83 (m, 4H), 3.18 (tt, J = 9.9, 3.6 Hz, 1H), 2.98–2.88 (m, 4H), 2.56 (s, 3H), 1.90–1.43 (m, 12H).

^{13}C-NMR (125 MHz, $CDCl_3$): δ = 197.3 (C_q), 154.0 (C_q), 145.1 (C_q), 133.0 (C_q), 127.6 (CH), 126.9 (CH), 119.2 (CH), 67.3 (CH_2), 53.2 (CH_2), 39.1 (CH), 36.8 (CH_2), 28.0 (CH_2), 27.7 (CH_2), 26.5 (CH_3).

IR (ATR): $\tilde{\nu}$ = 2915, 2850, 1677, 1595, 1450, 1355, 1235, 1114, 919, 828 cm^{-1}.

MS (EI) *m/z* (relative intensity): 301 (100) $[M]^+$, 286 (11) $[M–Me]^+$, 244 (18), 228 (29), 219 (28), 200 (24), 186 (25), 172 (40), 144 (16), 130 (15), 43 (37).

HR-MS (EI): *m/z* calcd for $C_{19}H_{27}NO_2^+$ $[M]^+$ 301.2036, found 301.2047.

1-[3-Cycloheptyl-4-(piperidin-1-yl)phenyl]ethan-1-one (3qf)

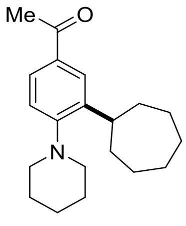

The general procedure **A** was followed using substrate **1q** (184 mg, 0.50 mmol) and bromide **2f** (266 mg, 1.50 mmol). After 20 h, to the reaction mixture was added HCl (2 N, 3.0 mL) and the resulting mixture was stirred for additional 3 h, and then neutralized with sat. aq. $NaHCO_3$ solution until pH 8. The reaction mixture was extracted with Et_2O (3 × 20 mL). The combined organic layers were dried over Na_2SO_4 and concentrated *in vacuo*. Purification of

the residue by column chromatography (*n*-hexane/EtOAc 95:5) yielded **3qf** (105 mg, 70%) as a colorless oil.

^{1}H-NMR (300 MHz, $CDCl_3$): δ = 7.81 (d, J = 2.2 Hz, 1H), 7.68 (dd, J = 8.3, 2.2 Hz, 1H), 6.99 (d, J = 8.3 Hz, 1H), 3.13 (tt, J = 10.1, 3.2 Hz, 1H), 2.84 (dd, J = 5.3, 5.1 Hz, 4H), 2.53 (s, 3H), 1.87–1.43 (m, 18H).

^{13}C-NMR (75 MHz, $CDCl_3$): δ = 197.6 (C_q), 156.0 (C_q), 145.1 (C_q), 132.2 (C_q), 127.4 (CH), 126.9 (CH), 119.0 (CH), 54.2 (CH_2), 38.9 (CH), 36.8 (CH_2), 28.0 (CH_2), 27.7 (CH_2), 26.5 (CH_2), 26.4 (CH_3), 24.3 (CH_2).

IR (ATR): $\tilde{\nu}$ = 2917, 2852, 1676, 1595, 1354, 1266, 920, 827, 599 cm^{-1}.

MS (EI) *m*/*z* (relative intensity): 299 (100) $[M]^+$, 284 (19) $[M–Me]^+$, 242 (30), 228 (35), 217 (35), 200 (19), 186 (38), 172 (27), 144 (14), 130 (14), 43 (42).

HR-MS (EI): *m*/*z* calcd for $C_{20}H_{29}NO^+$ $[M]^+$ 299.2244, found 299.2260.

1-[4-Fluoro-3-(4-methyltetrahydro-2*H*-pyran-4-yl)phenyl]ethan-1-one (3an)

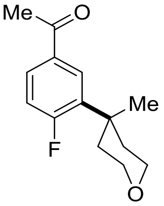

The general procedure **A** was followed using substrate **1a** (152 mg, 0.50 mmol) and bromide **2n** (269 mg, 1.50 mmol). After 20 h, purification by column chromatography (*n*-pentane/Et_2O 3:1) yielded **3an** (67 mg, 57%) as a colorless oil.

^{1}H-NMR (400 MHz, $CDCl_3$): δ = 7.93 (dd, J = 7.9, 2.3 Hz, 1H), 7.82 (ddd, J = 8.4, 4.6, 2.3 Hz, 1H), 7.09 (dd, J = 12.4, 8.4 Hz, 1H), 3.80 (d_{AB}dd, J = 11.0, 9.1, 3.1 Hz, 2H), 3.70 (d_{AB}dd, J = 11.0, 6.2, 3.8 Hz, 2H), 2.58 (s, 3H), 2.20 (ddd, J = 12.7, 9.1, 3.8 Hz, 2H), 1.91–1.82 (m, 2H), 1.40 (d, J = 1.0 Hz, 3H).

^{13}C-NMR (100 MHz, $CDCl_3$): δ = 196.7 (C_q), 165.0 (d, $^1J_{C\text{-}F}$ = 257 Hz, C_q), 135.9 (d, $^2J_{C\text{-}F}$ = 11 Hz, C_q), 133.3 (d, $^4J_{C\text{-}F}$ = 3 Hz, C_q), 128.8 (d, $^3J_{C\text{-}F}$ = 11 Hz, CH), 128.2 (d, $^3J_{C\text{-}F}$ = 8 Hz, CH), 116.9 (d, $^2J_{C\text{-}F}$ = 26 Hz, CH), 64.2 (CH_2), 36.7 (d, $^4J_{C\text{-}F}$ = 4 Hz, CH_2), 35.5 (d, $^3J_{C\text{-}F}$ = 3 Hz, C_q), 26.5 (CH_3), 25.3 (d, $^4J_{C\text{-}F}$ = 3 Hz, CH_3).

^{19}F-NMR (376 MHz, $CDCl_3$): δ = –101.8 (ddd, J = 12.4, 7.9, 4.6 Hz).

IR (ATR): $\tilde{\nu}$ = 2932, 2853, 1683, 1605, 1490, 1356, 1244, 1106, 826 cm^{-1}.

MS (EI) *m*/*z* (relative intensity): 236 (10) $[M]^+$, 221 (14) $[M–Me]^+$, 192 (53), 177 (65), 163 (90), 149 (49), 133 (20), 83 (22), 49 (22), 43 (100).

HR-MS (EI): *m*/*z* calcd for $C_{14}H_{17}FO_2^+$ $[M]^+$ 236.1207, found 236.1226.

tert-Butyl 4-(5-acetyl-2-fluorophenyl)piperidine-1-carboxylate (3ao)

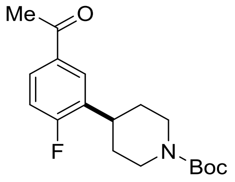

The general procedure **A** was followed using substrate **1a** (152 mg, 0.50 mmol) and bromide **2o** (396 mg, 1.50 mmol) was added in 3 portions after 3 h and 6 h at 140 °C. After 20 h, to the reaction mixture was added HCl (2 N, 3.0 mL) and the resulting mixture was stirred for additional 3 h, and then neutralized with sat. aq. $NaHCO_3$ solution until pH 8. The reaction mixture was extracted with Et_2O (3 × 20 mL). The combined organic layers were dried over Na_2SO_4 and concentrated *in vacuo*. Purification of the residue by column chromatography (*n*-pentane/Et_2O 3:1) yielded **3ao** (83 mg, 52%) as a colorless oil.

^{1}H-NMR (300 MHz, $CDCl_3$): δ = 7.86 (dd, J = 7.2, 2.3 Hz, 1H), 7.81 (ddd, J = 8.5, 5.0, 2.3 Hz, 1H), 7.09 (dd, J = 9.9, 8.5 Hz, 1H), 4.27 (d, J = 13.0 Hz, 2H), 3.03 (tt, J = 12.3, 3.7 Hz, 1H), 2.83 (dd, J = 13.0, 12.6 Hz, 2H), 2.58 (s, 3H), 1.81 (d_{AB}, J = 12.6 Hz, 2H), 1.69 (d_{AB}ddd, J = 12.6, 12.6, 12.3, 4.1 Hz, 2H), 1.49 (s, 9H).

^{13}C-NMR (125 MHz, $CDCl_3$): δ = 196.4 (C_q), 163.5 (d, $^1J_{C\text{-}F}$ = 253 Hz, C_q), 154.6 (C_q), 133.7 (d, $^4J_{C\text{-}F}$ = 3 Hz, C_q), 132.8 (d, $^2J_{C\text{-}F}$ = 15 Hz, C_q), 128.6 (d, $^3J_{C\text{-}F}$ = 10 Hz, CH), 128.2 (d, $^3J_{C\text{-}F}$ = 7 Hz, CH), 115.6 (d, $^2J_{C\text{-}F}$ = 24 Hz, CH), 79.6 (C_q), 44.3 (CH_2), 35.7 (d, $^3J_{C\text{-}F}$ = 2 Hz, CH), 31.7 (CH_2), 28.6 (CH_3), 26.6 (CH_3).

^{19}F-NMR (282 MHz, $CDCl_3$): δ = –111.5 (ddd, J = 9.9, 7.2, 5.0 Hz).

IR (ATR): $\tilde{\nu}$ = 2975, 2930, 1685, 1587, 1420, 1365, 1233, 1166, 1021, 819 cm^{-1}.

MS (EI) *m*/*z* (relative intensity): 321 (2) $[M]^+$, 266 (7) $[M–Bu]^+$, 248 (21) $[M–Bu–Me]^+$, 221 (51), 83 (9), 57 (100), 43 (40).

HR-MS (EI): *m*/*z* calcd for $C_{18}H_{24}FNO_3^+$ $[M]^+$ 321.1735, found 321.1742.

tert-Butyl 4-(3-acetylnaphthalen-1-yl)piperidine-1-carboxylate (3lo)

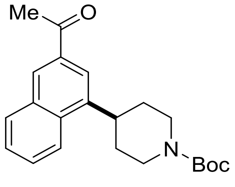

The general procedure **A** was followed using substrate **1l** (168 mg, 0.50 mmol) and bromide **2o** (396 mg, 1.50 mmol) was added by syringe pump over 5.5 h at 140 °C. After 20 h, to the reaction mixture was added HCl (2 N, 3.0 mL) and the resulting mixture was stirred for additional 3 h, and then neutralized with sat. aq. $NaHCO_3$ solution until pH 8. The reaction mixture was extracted with Et_2O (3 × 20 mL). The combined organic layers were dried over Na_2SO_4 and concentrated *in vacuo*. Purification of the residue by column chromatography (*n*-pentane/Et_2O 3:1) yielded **3lo** (96 mg, 54%) as a white solid

^{1}H-NMR (300 MHz, $CDCl_3$): δ = 8.32 (d, J = 1.6 Hz, 1H), 8.11 (d, J = 8.4 Hz, 1H), 8.01–7.96 (m, 1H), 7.93 (d, J = 1.6 Hz, 1H), 7.64 (ddd, J = 8.4, 6.9, 1.5 Hz, 1H), 7.55 (ddd, J = 8.0,

6.9, 1.2 Hz, 1H), 4.34 (br d, J = 13.1 Hz, 2H), 3.46 (tt, J = 11.8, 3.3 Hz, 1H), 2.96 (t, J = 12.8 Hz, 2H), 2.71 (s, 3H), 2.03–1.92 (m, 2H), 1.92–1.72 (m, 2H), 1.50 (s, 9H).

^{13}C-NMR (125 MHz, $CDCl_3$): δ = 197.9 (C_q), 154.6 (C_q), 142.2 (C_q), 134.0 (C_q), 133.4 (C_q), 133.1 (C_q), 130.6 (CH), 129.2 (CH), 128.4 (CH), 126.2 (CH), 122.8 (CH), 120.2 (CH), 79.5 (C_q), 44.6 (CH_2), 37.8 (CH), 32.8 (CH_2), 28.5 (CH_3), 26.5 (CH_3).

IR (ATR): $\tilde{\nu}$ = 2973, 2855, 1673, 1426, 1363, 1228, 1163, 1122, 891, 753 cm^{-1}.

m.p.: 54–56 °C.

MS (EI) *m*/*z* (relative intensity): 353 (10) $[M]^+$, 280 (10) $[M–Ot\text{-}Bu]^+$, 253 (46) $[M–Boc]^+$, 198 (21), 165 (9), 152 (14), 83 (17), 69 (9), 57 (100), 43 (61).

HR-MS (EI): *m*/*z* calcd for $C_{22}H_{27}NO_3^+$ $[M]^+$ 353.1985, found 353.1980.

N-[1-(4-Cycloheptylnaphthalen-2-yl)ethyl]-3,4,5-trimethoxyaniline (9a)

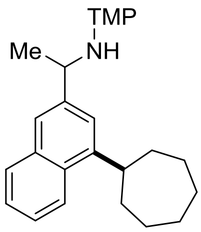

The general procedure **A** was followed using $[RuCl_2(p\text{-cymene})]_2$ (15.3 mg, 25.0 µmol), 1-$AdCO_2H$ (27.1 mg, 0.15 mmol), K_2CO_3 (139 mg, 1.0 mmol), substrate **1l** (168 mg, 0.5 mmol) and bromide **2f** (266 mg, 1.5 mmol). After 20 h, a solution of $ZnCl_2$ in THF (1.0 M, 0.5 mmol), $NaBH_3CN$ (63 mg, 2.0 mmol) and MeOH (1.5 mL) were successively added to the reaction mixture at ambient temperature. The reaction mixture was stirred at ambient temperature for 16 h and then distributed between Et_2O (8 mL) and sat. aq. K_2CO_3 (8 mL). The aqueous phase was extracted with Et_2O (2 × 10 mL). The combined organic layers were dried over Na_2SO_4 and concentrated *in vacuo*. Purification by column chromatography (*n*-hexane/EtOAc 10:1) yielded **9a** (167 mg, 77%) as a white solid as well as alkylated phenone **3lf** (4 mg, 3%) as a colorless oil.

^{1}H-NMR (400 MHz, $CDCl_3$): δ = 8.10 (d, J = 7.9 Hz, 1H), 7.84–7.80 (m, 1H), 7.69 (br s, 1H), 7.52–7.40 (m, 3H), 5.85 (s, 2H), 4.59 (q, J = 6.7 Hz, 1H), 4.07 (br s, 1H), 3.74 (s, 3H), 3.70 (s, 6H), 3.56–3.47 (m, 1H), 2.14–2.03 (m, 2H), 1.96–1.75 (m, 6H), 1.75–1.63 (m, 4H), 1.61 (d, J = 6.7 Hz, 3H).

^{13}C-NMR (100 MHz, $CDCl_3$): δ = 153.6 (C_q), 146.4 (C_q), 144.2 (C_q), 142.3 (C_q), 134.1 (C_q), 130.3 (C_q), 129.8 (C_q), 128.8 (CH), 125.4 (CH), 125.2 (CH), 123.1 (CH), 122.1 (CH), 121.3 (CH), 91.1 (CH), 60.9 (CH_3), 55.7 (CH_3), 54.5 (CH), 40.9 (CH), 36.5 (CH_2), 36.2 (CH_2), 27.8 (CH_2), 27.8 (CH_2), 27.6 (CH_2), 27.6 (CH_2), 24.6 (CH_3).

IR (ATR): $\tilde{\nu}$ = 3362, 2919, 1604, 1507, 1446, 1235, 1123, 1009, 810, 749 cm^{-1}.

m.p.: 158–159 °C.

MS (EI) *m*/*z* (relative intensity): 433 (80) $[M]^+$, 418 (18) $[M–Me]^+$, 251 (100), 183 (24), 168 (35), 155 (16), 55 (24).

HR-MS (EI): *m*/*z* calcd for $C_{28}H_{35}NO_3^+$ $[M]^+$ 433.2611, found 433.2628.

3,4,5-Trimethoxy-*N*-{1-[4-(octan-2-yl)naphthalen-2-yl]ethyl}aniline (9b)

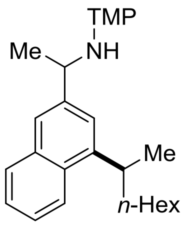

The general procedure **A** was followed using $[RuCl_2(p\text{-cymene})]_2$ (15.3 mg, 25.0 μmol), 1-AdCO$_2$H (27.1 mg, 0.15 mmol), K_2CO_3 (139 mg, 1.0 mmol), substrate **1l** (168 mg, 0.5 mmol) and bromide **2m** (290 mg, 1.50 mmol). After 20 h, a solution of $ZnCl_2$ in THF (1.0 M, 0.5 mmol), $NaBH_3CN$ (63 mg, 2.0 mmol) and MeOH (1.5 mL) were successively added to the reaction mixture at ambient temperature. The reaction mixture was stirred at ambient temperature for 16 h and then distributed between Et_2O (8 mL) and sat. aq. K_2CO_3 (8 mL). The aqueous phase was extracted with Et_2O (2 × 10 mL). The combined organic layers were dried over Na_2SO_4 and concentrated *in vacuo*. Purification by column chromatography (*n*-hexane/EtOAc 10:1) yielded **9b** (169 mg, 75%, dr 1.0:1.2) as a viscous yellow oil.

^{1}H-NMR (300 MHz, $CDCl_3$, 2 diastereomers): δ = 8.11–8.06 (m, 2H), 7.83–7.78 (m, 2H), 7.68 (br s, 2H), 7.49–7.41 (m, 4H), 7.40–7.36 (m, 2H), 5.82 (s, 2H), 5.81 (s, 2H), 4.58 (q, J = 6.7 Hz, 2H), 4.02 (br s, 2H), 3.71 (s, 3H), 3.70 (s, 3H), 3.68 (s, 6H), 3.67 (s, 6H), 3.62–3.52 (m, 2H), 1.89–1.73 (m, 2H), 1.73–1.63 (m, 2H), 1.59 (d, J = 6.7 Hz, 3H), 1.58 (d, J = 6.7 Hz, 3H), 1.36 (d, J = 6.9 Hz, 3H), 1.35 (d, J = 6.9 Hz, 3H), 1.33–1.17 (m, 16H), 0.91–0.80 (m, 6H).

^{13}C-NMR (75 MHz, $CDCl_3$, 2 diastereomers): δ = 153.7 (2 × C_q), 144.8 (C_q), 144.7 (C_q), 144.2 (2 × C_q), 144.2 (2 × C_q), 142.3 (C_q), 142.3 (C_q), 134.2 (C_q), 134.2 (C_q), 131.0 (C_q), 131.0 (C_q), 129.9 (2 × C_q), 128.8 (CH), 128.8 (CH), 125.4 (2 × CH), 125.3 (2 × CH), 123.1 (2 × CH), 122.3 (CH), 122.3 (CH), 121.2 (2 × CH), 91.1 (4 × CH), 61.0 (CH_3), 61.0 (CH_3), 55.7 (4 × CH_3), 54.5 (2 × CH), 38.1 (CH_2), 37.7 (CH_2), 33.8 (CH), 33.7 (CH), 31.8 (CH_2), 31.7 (CH_2), 29.5 (CH_2), 29.4 (CH_2), 27.8 (CH_2), 27.7 (CH_2), 24.8 (2 × CH_3), 22.7 (CH_2), 22.6 (CH_2), 22.0 (CH_3), 21.7 (CH_3), 14.1 (CH_3), 14.0 (CH_3).

IR (ATR): $\tilde{\nu}$ = 3383, 2926, 1608, 1507, 1451, 1232, 1125, 1011, 782, 746 cm^{-1}.

MS (EI) *m*/*z* (relative intensity): 449 (65) $[M]^+$, 434 (19) $[M–Me]^+$, 267 (100), 183 (35), 168 (39), 155 (16), 43 (13).

HR-MS (EI): *m*/*z* calcd for $C_{29}H_{39}NO_3^+$ $[M]^+$ 449.2924, found 449.2921.

N-[1-(3-Cycloheptylphenyl)ethyl]-3,4,5-trimethoxyaniline (9c)

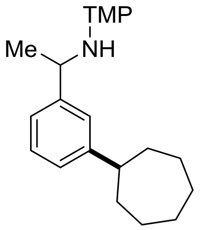

The general procedure **A** was followed using $[RuCl_2(p\text{-cymene})]_2$ (30.6 mg, 50.0 µmol), 1-$AdCO_2H$ (54.1 mg, 0.30 mmol), K_2CO_3 (276 mg, 2.0 mmol), substrate **1b** (285 mg, 1.0 mmol) and bromide **2f** (531 mg, 3.0 mmol). After 20 h, a solution of $ZnCl_2$ in THF (1.0 M, 1.0 mmol), $NaBH_3CN$ (126 mg, 2.0 mmol) and MeOH (3 mL) were successively added to the reaction mixture at ambient temperature. The reaction mixture was stirred at ambient temperature for 16 h and then distributed between Et_2O (15 mL) and sat. aq. K_2CO_3 (15 mL). The aqueous phase was extracted with Et_2O (2 × 20 mL). The combined organic layers were dried over Na_2SO_4 and concentrated *in vacuo*. Purification by column chromatography (*n*-hexane/EtOAc 10:1) yielded **9c** (200 mg, 52%) as a light yellow oil as well as alkylated phenone **3bf** (51 mg, 24%) as a colorless oil.

^{1}H-NMR (500 MHz, $CDCl_3$): δ = 7.23 (dd, *J* = 7.5, 7.5 Hz, 1H), 7.19–7.15 (m, 2H), 7.08–7.04 (m, 1H), 5.76 (s, 2H), 4.39 (q, *J* = 6.7 Hz, 1H), 3.92 (br s, 1H), 3.72 (s, 3H), 3.69 (s, 6H), 2.65 (tt, *J* = 10.6, 3.6 Hz, 1H), 1.92–1.84 (m, 2H), 1.82–1.74 (m, 2H), 1.74–1.53 (m, 8H), 1.51 (d, *J* = 6.7 Hz, 3H).

^{13}C-NMR (125 MHz, $CDCl_3$): δ = 153.6 (C_q), 150.4 (C_q), 145.2 (C_q), 144.2 (C_q), 129.7 (C_q), 128.6 (CH), 125.2 (CH), 124.4 (CH), 122.8 (CH), 90.9 (CH), 61.0 (CH_3), 55.7 (CH_3), 54.4 (CH), 47.0 (CH), 36.9 (CH_2), 36.7 (CH_2), 27.9 (CH_2), 27.9 (CH_2), 27.2 (CH_2), 27.2 (CH_2), 24.8 (CH_3).

IR (ATR): $\tilde{\nu}$ = 3356, 2996, 2850, 1599, 1507, 1447, 1205, 1126, 1008, 812 cm^{-1}.

MS (EI) *m/z* (relative intensity): 383 (92) $[M]^+$, 201 (100), 168 (78), 119 (15).

HR-MS (EI): *m/z* calcd for $C_{24}H_{33}NO_3^+$ $[M]^+$ 383.2455, found 383.2469.

1-[4-(1-Methyl-cyclohexyl)-5-fluoro-4'-methoxy-[1,1'-biphenyl]-2-yl]ethan-1-one (10a):

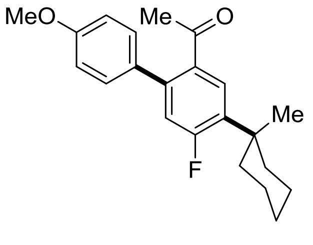

The general procedure **A** was followed using $[RuCl_2(p\text{-cymene})]_2$ (15.3 mg, 25.0 µmol), 1-$AdCO_2H$ (27 mg, 0.15 mmol), substrate **1a** (152 mg, 0.50 mmol), bromide **2a** (266 mg, 1.50 mmol) and K_2CO_3 (276 mg, 2.00 mmol). After 20 h, 4-bromoanisole (140 mg, 0.75 mmol) was added to the reaction at ambient temperature and then the mixture was stirred at 120 °C for 20 h. At ambient temperature, HCl (2 N, 3.0 mL) was added, and the resulting mixture was stirred for 3 h and extracted with EtOAc (3 × 20 mL). The combined organic layers were dried over Na_2SO_4 and concentrated *in vacuo*. Purification by column chromatography (*n*-hexane/EtOAc 25:1) yielded **10a** (118 mg, 69%)

as a colorless solid.

^{1}H-NMR (400 MHz, $CDCl_3$): δ = 7.52 (d, J = 8.6 Hz, 1H), 7.25 (d, J = 8.9 Hz, 2H), 6.99 (d, J = 13.3 Hz, 1H), 6.96 (d, J = 8.9 Hz, 2H), 3.85 (s, 3H), 2.12–2.04 (m, 2H), 1.99 (s, 3H), 1.73–1.65 (m, 2H), 1.64–1.56 (m, 2H), 1.54–1.38 (m, 4H), 1.32 (d, J = 0.8 Hz, 3H).

^{13}C-NMR (100 MHz, $CDCl_3$): δ = 204.5 (C_q), 163.1 (d, $^1J_{C\text{-}F}$ = 254 Hz, C_q), 159.9 (C_q), 140.4 (d, $^2J_{C\text{-}F}$ = 9.6 Hz, C_q), 136.5 (d, $^4J_{C\text{-}F}$ = 2.9 Hz, C_q), 135.6 (d, $^3J_{C\text{-}F}$ = 9.6 Hz, C_q), 132.1 (d, $^4J_{C\text{-}F}$ = 1.7 Hz, C_q), 130.0 (CH), 129.0 (d, $^3J_{C\text{-}F}$ = 7.5 Hz, CH), 118.2 (d, $^2J_{C\text{-}F}$ = 25.9 Hz, CH), 114.4 (CH), 55.5 (CH_3), 38.0 (d, $^3J_{C\text{-}F}$ = 3.5 Hz, C_q), 37.2 (d, $^4J_{C\text{-}F}$ = 3.8 Hz, CH_2), 30.7 (CH_3), 26.6 (CH_2), 26.5 (CH_3), 22.8 (CH_2).

^{19}F-NMR (470 MHz, $CDCl_3$) δ = –105.0 (dd, J = 13.3, 8.6 Hz).

IR (ATR): $\tilde{\nu}$ = 1674, 1608, 1485, 1467, 1267, 1245, 1169, 1031, 833, 532 cm^{-1}.

m.p.: 58 °C.

MS (ESI) m/z (relative intensity): 341 $[M+H]^+$ (75), 363 $[M+Na]^+$ (100), 703 $[2M+Na]^+$ (90).

HR-MS (ESI): m/z calcd for $C_{22}H_{25}FO_2^+$ $[M+H]^+$ 341.1911, found 341.1908.

1-[1-*n*-Hexyl-5-(1-methyl-cyclohexyl)-4-fluorophenyl]ethan-1-one (10b):

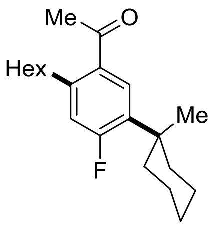

The general procedure **A** was followed using $[RuCl_2(p\text{-cymene})]_2$ (15.3 mg, 25.0 µmol), 1-$AdCO_2H$ (27 mg, 0.15 mmol), substrate **1a** (152 mg, 0.50 mmol), bromide **2a** (266 mg, 1.50 mmol) and K_2CO_3 (276 mg, 2.00 mmol). After 20 h, 1-bromohexane (248 mg, 1.50 mmol) was added to the reaction at ambient temperature and then the mixture was stirred at 120 °C for 20 h. At ambient temperature, HCl (2 N, 3.0 mL) was added, and the resulting mixture was stirred for 3 h and extracted with EtOAc (3 × 20 mL). The combined organic layers were dried over Na_2SO_4 and concentrated *in vacuo*. Purification by column chromatography (*n*-hexane/EtOAc 30:1) followed by HPLC separation yielded **10b** (89 mg, 56%) as a colorless oil.

^{1}H-NMR (400 MHz, $CDCl_3$): δ = 7.62 (d, J = 8.5 Hz, 1H), 6.87 (d, J = 13.9 Hz, 1H), 2.84–2.77 (m, 2H), 2.56 (s, 3H), 2.10–1.97 (m, 2H), 1.72–1.23 (m, 18H), 0.91–0.84 (m, 3H).

^{13}C-NMR (100 MHz, $CDCl_3$): δ = 201.1 (C_q), 163.5 (C_q, d, J = 255 Hz), 144.0 (C_q, d, J = 9.4 Hz), 133.7 (C_q, d, J = 2.8 Hz), 133.5 (C_q, d, J = 11.3 Hz), 130.2 (CH, d, J = 7.7 Hz), 119.1 (CH, d, J = 25.0 Hz), 37.7 (C_q, d, J = 3.5 Hz), 37.2 (CH_2, d, J = 4.0 Hz), 33.6 (CH_2, d, J = 1.2 Hz), 31.8 (CH_2), 31.4 (CH_2), 30.0 (CH_3), 29.5 (CH_2), 26.8 (CH_3), 26.4 (CH_2), 22.8 (CH_2), 22.7 (CH_2).

^{19}F-NMR (282 MHz, $CDCl_3$) δ = –104.1 (dd, J = 13.9, 8.5 Hz).

IR (ATR): $\tilde{\nu}$ = 2925, 2856, 1684, 1556, 1455, 1389, 1354, 1247, 1141, 899 cm^{-1}.
MS (EI) *m*/*z* (relative intensity): 318 (11) $[M]^+$, 303 (100) $[M–Me]^+$, 43 (46).
HR-MS (EI): *m*/*z* calcd for $C_{21}H_{31}FO^+$ $[M]^+$ 318.2359, found 318.2353.

3-(*tert*-Butyl)-4-fluorobenzoic acid (11)

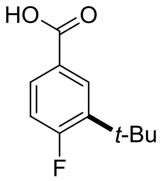

A mixture of phenone **3ab** (98 mg, 0.50 mmol) and anhydrous $Mn(OAc)_2$ (1.0 mg, 5.8 µmol) in acetic acid (0.5 mL) was stirred at 100 °C under an oxygen atmosphere for 15 h.[8] The reaction mixture was concentrated *in vacuo* to give a crude mixture. The crude mixture was dissolved with EtOAc and washed with HCl (1 N, 10 mL). The organic phase was dried over anhydrous Na_2SO_4. Filtration followed by evaporation gave a crude product. Purification of the residue by column chromatography (SiO_2, *n*-hexane/EtOAc 9:1 to 1:1) yielded the corresponding product **11** (87 mg, 89%) as a colorless solid.

^{1}H-NMR (500 MHz, $CDCl_3$): δ = 8.09 (dd, *J* = 8.0, 2.2 Hz, 1H), 7.96 (ddd, *J* = 8.5, 4.6, 2.2 Hz, 1H), 7.06 (dd, *J* = 12.0, 8.5 Hz, 1H), 1.40 (d, *J* = 1.0 Hz, 9H).
^{13}C-NMR (125 MHz, $CDCl_3$): δ = 171.4 (C_q), 165.6 (d, $^1J_{C\text{-}F}$ = 258 Hz, C_q), 137.6 (d, $^2J_{C\text{-}F}$ = 13 Hz, C_q), 130.4 (d, $^3J_{C\text{-}F}$ = 11 Hz, CH), 130.1 (d, $^3J_{C\text{-}F}$ = 8 Hz, CH), 124.9 (d, $^4J_{C\text{-}F}$ = 3 Hz, C_q), 116.7 (d, $^2J_{C\text{-}F}$ = 26 Hz, CH), 34.4 (d, $^3J_{C\text{-}F}$ = 3 Hz, C_q), 29.7 (d, $^4J_{C\text{-}F}$ = 3 Hz, CH_3).
^{19}F-NMR (470 MHz, $CDCl_3$): δ = (–100.4) – (–100.5) (m).
mp.: 154–155 °C.
IR (ATR): $\tilde{\nu}$ = 2964, 1683, 1428, 1294, 1258, 1217, 1089, 839, 772 cm^{-1}.
MS (EI) *m*/*z* (relative intensity): 196 (14) $[M]^+$, 181 (100) $[M–Me]^+$, 153 (79), 109 (15).
HR-MS (EI): *m*/*z* calcd for $C_{11}H_{13}FO_2^+$ $[M]^+$ 196.0894, found 196.0902.

2-(4-Fluoro-3-(*tert*-pentyl)phenyl)-1*H*-indole (12)

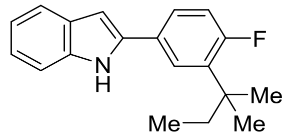

The indole was prepared by a modified literature procedure.[9] Phenone **3ac** (104 mg, 0.50 mmol), phenylhydrazine hydrochloride salt (147 mg, 1.0 mmol), and polyphosphoric acid (0.5 mL) in a microwave tube were stirred at 120 °C under microwave irradiation for 1 h. Then, sat. $NaHCO_{3(aq)}$ (20 mL) and CH_2Cl_2 (20 mL) were added. The layers were separated and the aqueous layer was extracted with CH_2Cl_2 (3 × 20 mL). The combined organic layers were dried over Na_2SO_4 and concentrated *in vacuo*. Purification by column chromatography (*n*-hexane/EtOAc 20:1 and Et_3N 1%) yielded aniline **12** (81 mg, 58%) as a pale yellow solid.

^{1}H-NMR (500 MHz, $CDCl_3$): δ = 8.27 (br s, 1H), 7.63 (d, J = 7.9 Hz, 1H), 7.54 (dd, J = 7.7, 2.4 Hz, 1H), 7.46 (ddd, J = 8.3, 4.4, 2.4 Hz, 1H), 7.41 (dd, J = 8.1, 1.1 Hz, 1H), 7.20 (ddd, J = 8.1, 7.1, 1.2 Hz, 1H), 7.13 (ddd, J = 7.9, 7.1, 1.1 Hz, 1H), 7.07 (dd, J = 12.3, 8.3 Hz, 1H), 6.76 (dd, J = 2.2, 0.9 Hz, 1H), 1.84 (qd, J = 7.5, 1.5 Hz, 2H), 1.42 (d, J = 1.1 Hz, 6H), 0.74 (t, J = 7.5 Hz, 3H).

^{13}C-NMR (125 MHz, $CDCl_3$): δ = 161.6 (d, $^{1}J_{C\text{-}F}$ = 250 Hz, C_q), 137.7 (C_q), 136.7 (C_q), 136.2 (d, $^{2}J_{C\text{-}F}$ = 12 Hz, C_q), 129.3 (C_q), 128.2 (d, $^{4}J_{C\text{-}F}$ = 3 Hz, C_q), 125.6 (d, $^{3}J_{C\text{-}F}$ = 6 Hz, CH), 124.4 (d, $^{3}J_{C\text{-}F}$ = 9 Hz, CH), 122.2 (CH), 120.5 (CH), 120.3 (CH), 116.9 (d, $^{2}J_{C\text{-}F}$ = 26 Hz, CH), 110.8 (CH), 99.7 (d, $^{6}J_{C\text{-}F}$ = 1 Hz, CH), 38.1 (d, $^{3}J_{C\text{-}F}$ = 3 Hz, C_q), 34.1 (d, $^{4}J_{C\text{-}F}$ = 4 Hz, CH_2), 27.7 (d, $^{4}J_{C\text{-}F}$ = 3 Hz, CH_3), 9.5 (CH_3).

^{19}F-NMR (470 MHz, $CDCl_3$): δ = (–109.9) – (–110.1) (m).

IR (ATR): $\tilde{\nu}$ = 3423, 2967, 1480, 1454, 1230, 797, 747 cm^{-1}.

m.p.: 142–144 °C.

MS (ESI) *m*/*z* (relative intensity): 561 (25), 490 (45), 381 (77), 312 (41), 282 (74) $[M+H]^+$, 118 (100).

HR-MS (ESI): *m*/*z* calcd for $C_{19}H_{21}FN^+$ $[M+H]^+$ 282.1653, found 282.1642.

3-(*tert*-Butyl)phenol (13a)

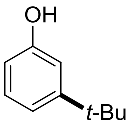

The general procedure **C** was followed using phenone **3bb** (88.1 mg, 0.50 mmol) and *m*-CPBA (370 mg, 70%wt purity, 1.50 mmol). Purification by column chromatography (*n*-pentane/Et_2O 10:1→1:1) yielded **13a** (60 mg, 80%) as an orange oil.

^{1}H-NMR (300 MHz, $CDCl_3$): δ = 7.19 (td, J = 7.9, 0.4 Hz, 1H), 6.99 (ddd, J = 7.9, 1.8, 1.0 Hz, 1H), 6.91–6.89 (m, 1H), 6.67 (ddd, J = 7.9, 2.6, 1.0 Hz, 1H), 5.24 (br s, 1H), 1.31 (s, 9H).

^{13}C-NMR (125 MHz, $CDCl_3$): δ = 155.1 (C_q), 153.2 (C_q), 129.1 (CH), 117.8 (CH), 112.5 (CH), 112.2 (CH), 34.7 (C_q), 31.3 (CH_3).

IR (ATR): $\tilde{\nu}$ = 3315, 2961, 1588, 1486, 1450, 1281, 1218, 915, 781, 699 cm^{-1}.

MS (EI) *m*/*z* (relative intensity): 150 (39) $[M]^+$, 135 (100) $[M–Me]^+$, 107 (56), 58 (41).

HR-MS (EI): *m*/*z* calcd for $C_{10}H_{14}O^+$ $[M]^+$ 150.1039, found 150.1045.

The spectral data are in accordance with those reported in the literature.[10]

3-(5-Chloro-2-methylpentan-2-yl)-4-fluorophenol (13b)

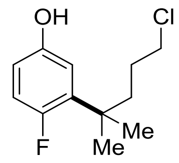

The general procedure **C** was followed using phenone **3ae** (27.3 mg, 0.11 mmol) and *m*-CPBA (78.9 mg, 70%wt purity, 0.33 mmol). Purification by column chromatography (*n*-pentane/Et_2O 10:1→1:1) yielded **13b** (23 mg, 92%) as an orange oil.

^{1}H-NMR (400 MHz, $CDCl_3$): δ = 6.85 (dd, J = 12.0, 8.7 Hz, 1H), 6.70 (dd, J = 6.6, 3.2 Hz, 1H), 6.62 (ddd, J = 8.7, 3.5, 3.2 Hz, 1H), 3.44 (t, J = 6.8 Hz, 2H), 1.89–1.81 (m, 2H), 1.58–1.48 (m, 2H), 1.34 (d, J = 1.0 Hz, 6H).

^{13}C-NMR (100 MHz, $CDCl_3$): δ = 156.1 (d, $^1J_{C\text{-}F}$ = 240 Hz, C_q), 151.1 (d, $^4J_{C\text{-}F}$ = 2 Hz, C_q), 136.1 (d, $^2J_{C\text{-}F}$ = 13 Hz, C_q), 116.8 (d, $^2J_{C\text{-}F}$ = 27 Hz, CH), 115.1 (d, $^3J_{C\text{-}F}$ = 6 Hz, CH), 113.6 (d, $^3J_{C\text{-}F}$ = 9 Hz, CH), 45.6 (CH_2), 38.9 (d, $^4J_{C\text{-}F}$ = 5 Hz, CH_2), 37.4 (d, $^3J_{C\text{-}F}$ = 3 Hz, C_q), 28.7 (CH_2), 28.1 (d, $^4J_{C\text{-}F}$ = 3 Hz, CH_3).

^{19}F-NMR (376 MHz, $CDCl_3$): δ = (–120.4) – (–120.5) (m).

IR (ATR): $\tilde{\nu}$ = 3349, 2961, 1484, 1439, 1196, 813, 765 cm^{-1}.

MS (ESI) *m*/*z* (relative intensity): 495 (18), 459 (100), 263 (19), 229 (99) $[M-H]^-$.

HR-MS (ESI): *m*/*z* calcd for $C_{12}H_{15}ClFO^-$ $[M-H]^-$ 229.0801, found 229.0806.

4-Fluoro-3-(*tert*-pentyl)phenol (13c)

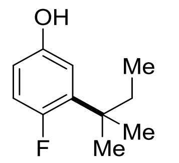

The general procedure **C** was followed using phenone **3ac** (104 mg, 0.50 mmol) and *m*-CPBA (370 mg, 70%wt purity, 1.50 mmol). Purification by column chromatography (*n*-pentane/Et_2O 10:1→1:1) yielded **13c** (85 mg, 93%) as an orange oil.

^{1}H-NMR (300 MHz, $CDCl_3$): δ = 6.84 (dd, J = 12.0, 8.6 Hz, 1H), 6.73 (dd, J = 6.6, 3.2 Hz, 1H), 6.62 (ddd, J = 8.6, 3.3, 3.2 Hz, 1H), 1.75 (qd, J = 7.5, 1.4 Hz, 2H), 1.30 (d, J = 1.0 Hz, 6H), 0.69 (td, J = 7.5, 0.6 Hz, 3H).

^{13}C-NMR (125 MHz, $CDCl_3$): δ = 156.2 (d, $^1J_{C\text{-}F}$ = 240 Hz, C_q), 150.8 (d, $^4J_{C\text{-}F}$ = 2 Hz, C_q), 136.7 (d, $^2J_{C\text{-}F}$ = 13 Hz, C_q), 116.6 (d, $^2J_{C\text{-}F}$ = 27 Hz, CH), 115.3 (d, $^3J_{C\text{-}F}$ = 6 Hz, CH), 113.3 (d, $^3J_{C\text{-}F}$ = 9 Hz, CH), 38.0 (d, $^3J_{C\text{-}F}$ = 3 Hz, C_q), 34.1 (d, $^4J_{C\text{-}F}$ = 4 Hz, CH_2), 27.6 (d, $^4J_{C\text{-}F}$ = 3 Hz, CH_3), 9.4 (CH_3).

^{19}F-NMR (282 MHz, $CDCl_3$): δ = (–120.2) – (–120.4) (m).

IR (ATR): $\tilde{\nu}$ = 3315, 2965, 1482, 1440, 1194, 811, 761, 738 cm^{-1}.

MS (EI) *m*/*z* (relative intensity): 182 (36) $[M]^+$, 153 (100) $[M–Et]^+$, 125 (81).

HR-MS (EI): *m*/*z* calcd for $C_{11}H_{15}FO^+$ $[M]^+$ 182.1101, found 182.1103.

4-Fluoro-3-(*tert*-pentyl)aniline (14)

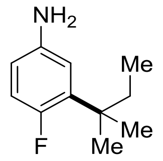

The title aniline was prepared by a modified literature procedure.[11] Phenone **3ac** (104 mg, 0.50 mmol), $NH_2OH{\cdot}HCl$ (52.1 mg, 0.75 mmol) and $FeCl_3{\cdot}6H_2O$ were placed in a pre-dried 10 mL pressure. The mixture was stirred at 130 °C for 2 h. Then, at ambient temperature, sat. $NaHCO_{3(aq)}$ (20 mL) and CH_2Cl_2 (20 mL) were added. The layers were separated and the aqueous later was extracted with CH_2Cl_2 (3 × 20 mL). The combined organic layers were concentrated *in vacuo*. To the crude mixture was added NaOH(aq) (2 M, 2 mL) and EtOH/water (3:1, 4 mL). The mixture was stirred at 60 °C for 3 h. Then, sat. $NaHCO_{3(aq)}$ (20 mL) and CH_2Cl_2 (20 mL) added. The layers were separated and the aqueous layer was extracted with CH_2Cl_2 (3 × 20 mL). The combined organic layers were dried over Na_2SO_4 and concentrated *in vacuo*. Purification by column chromatography (*n*-pentane/Et_2O 10:1→1:1) yielded aniline **14** (55 mg, 61%) as a colorless oil.

^{1}H-NMR (400 MHz, $CDCl_3$): δ = 6.77 (dd, J = 12.4, 8.5 Hz, 1H), 6.55 (dd, J = 6.9, 2.9 Hz, 1H), 6.47 (ddd, J = 8.5, 3.7, 2.9 Hz, 1H), 3.48 (br s, 2H), 1.74 (qd, J = 7.5, 1.4 Hz, 2H), 1.30 (d, J = 1.0 Hz, 6H), 0.69 (td, J = 7.5, 0.6 Hz, 3H).

^{13}C-NMR (100 MHz, $CDCl_3$): δ = 155.5 (d, $^1J_{C\text{-}F}$ = 238 Hz, C_q), 141.8 (d, $^4J_{C\text{-}F}$ = 2 Hz, C_q), 136.0 (d, $^2J_{C\text{-}F}$ = 13 Hz, C_q), 116.4 (d, $^2J_{C\text{-}F}$ = 26 Hz, CH), 115.3 (d, $^3J_{C\text{-}F}$ = 6 Hz, CH), 113.4 (d, $^3J_{C\text{-}F}$ = 9 Hz, CH), 37.8 (d, $^3J_{C\text{-}F}$ = 3 Hz, C_q), 34.1 (d, $^4J_{C\text{-}F}$ = 5 Hz, CH_2), 27.6 (d, $^4J_{C\text{-}F}$ = 3 Hz, CH_3), 9.4 (CH_3).

^{19}F-NMR (376 MHz, $CDCl_3$): δ = (–122.6) – (–122.8) (m).

IR (ATR): $\tilde{\nu}$ = 3361, 2964, 2874, 1493, 1436, 1203, 863, 810 cm^{-1}.

MS (ESI) *m*/*z* (relative intensity): 182 (100) $[M+H]^+$.

HR-MS (ESI): *m*/*z* calcd for $C_{11}H_{17}FN^+$ $[M+H]^+$ 182.1340, found 182.1340.

Synthesis of Ruthenium(II) complex (7)

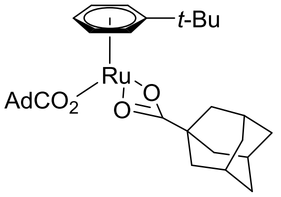

A suspension of $[RuCl_2(t\text{-}BuPh)]_2$ (153 mg, 0.25 mmol), 1-$AdCO_2H$ (270 mg, 1.5 mmol) and K_2CO_3 (346 mg, 2.5 mmol) in toluene (20 mL) was stirred at 23 °C for 42 h. Removal of the solvent, dissolution in CH_2Cl_2 (10 mL), filtration over celite and evaporation of the filtrate yielded **7** (294 mg, 99%) as an orange solid.

^{1}H-NMR (300 MHz, $CDCl_3$): δ = 5.92–5.85 (m, 1H), 5.82–5.72 (m, 4H), 1.99–1.54 (m, 30H), 1.42 (s, 9H).

^{13}C-NMR (125 MHz, $CDCl_3$): δ = 191.4 (C_q), 104.6 (C_q), 78.4 (CH), 78.0 (CH), 75.5 (CH), 42.2 (C_q), 39.4 (CH_2), 36.9 (CH_2), 34.6 (C_q), 30.3 (CH_3), 28.4 (CH).

mp.: 187 °C.

IR (ATR): $\tilde{\nu}$ = 2901, 2849, 1611, 1482, 1449, 1335, 1295, 813, 679, 488 cm^{-1}.

MS (LIFDI) *m/z* calcd for $[C_{32}H_{44}O_4Ru]^+$ 594.2, found 594.1.

Elemental analysis: calcd. C 64.73%, H 7.47%, found C 64.78%, H 7.37%.

Competition Experiments

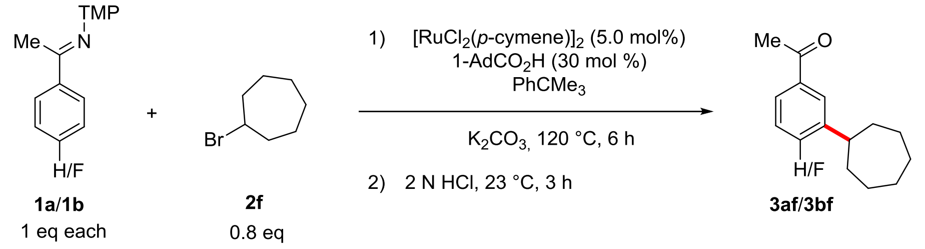

^{1}H NMR (400 MHz); before quenching H:F = 1.00 : 5.30

^{1}H NMR (300 MHz); after quenching H:F = 1.00 : 5.00

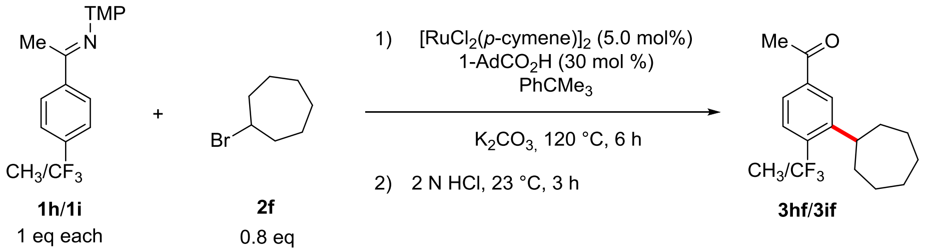

^{1}H NMR (400 MHz); before quenching CH_3:CF_3 = 1.00 : 1.45

^{1}H NMR (300 MHz); after quenching CH_3:CF_3 = 1.00 : 1.54

Supplementary Figure 1: Intermolecular competition experiments.

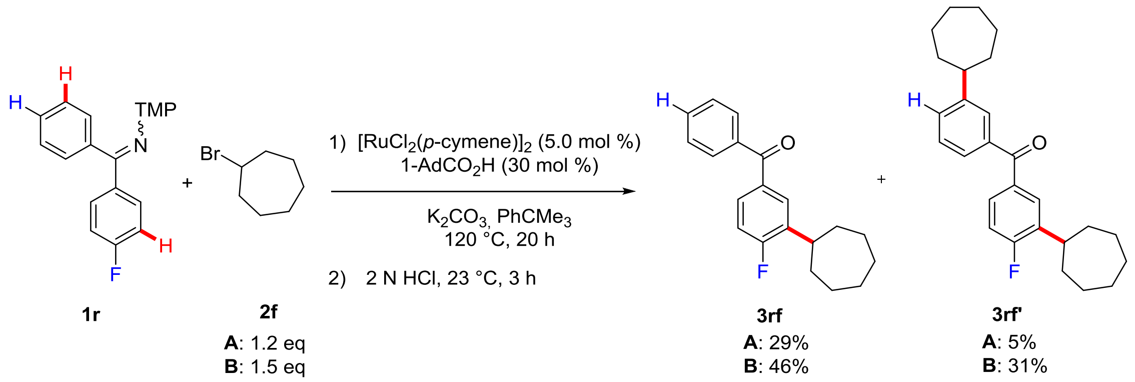

Supplementary Figure 2: Intramolecular competition experiments.

Alkyl bromide 2f (1.2 equiv)

The general procedure **A** was followed using substrate **1r** (183 mg, 0.50 mmol) and bromide **2f** (107 mg, 0.60 mmol). After 20 h, purification by column chromatography (*n*-hexane/EtOAc 100:1) followed by HPLC yielded monoalkylated **3rf** (43 mg, 29%) as a colorless oil as well as dialkylated **3rf’** (9 mg, 5%) as a colorless oil and recovered unreacted ketone (43 mg, 43%) as a colorless oil.

Alkyl bromide 2f (1.5 equiv)

The general procedure **A** was followed using substrate **1r** (183 mg, 0.50 mmol) and bromide **2f** (133 mg, 0.75 mmol). After 20 h, purification by column chromatography (*n*-hexane/EtOAc 100:1) followed by HPLC yielded monoalkylated **3rf** (68 mg, 46%) as a colorless oil as well as dialkylated **3rf'** (62 mg, 31%) as a colorless oil and recovered unreacted ketone (8 mg, 8%) as a colorless oil.

(3-Cycloheptyl-4-fluorophenyl)(phenyl)methanone (3rf)

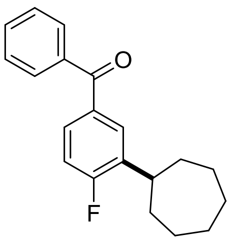

^{1}H-NMR (600 MHz, $CDCl_3$): δ = 7.78–7.75 (m, 3H), 7.61–7.57 (m, 2H), 7.51–7.47 (m, 2H), 7.07 (dd, J = 9.9, 8.4 Hz, 1H), 3.05 (tt, J = 10.7, 3.5 Hz, 1H), 1.94–1.88 (m, 2H), 1.84–1.77 (m, 2H), 1.75–1.65 (m, 4H), 1.63–1.53 (m, 4H).

^{13}C-NMR (125 MHz, $CDCl_3$): δ = 195.7 (C_q), 162.8 (d, $^{1}J_{C\text{-}F}$ = 253 Hz, C_q), 137.7 (C_q), 136.7 (d, $^{2}J_{C\text{-}F}$ = 16 Hz, C_q), 133.6 (d, $^{4}J_{C\text{-}F}$ = 3 Hz, C_q), 132.3 (CH), 130.5 (d, $^{3}J_{C\text{-}F}$ = 7 Hz, CH), 129.9 (CH), 129.7 (d, $^{3}J_{C\text{-}F}$ = 10 Hz, CH), 128.3 (CH), 115.2 (d, $^{2}J_{C\text{-}F}$ = 24 Hz, CH), 39.5 (CH), 35.2 (CH_2), 27.7 (CH_2), 27.3 (CH_2).

^{19}F-NMR (470 MHz, $CDCl_3$): δ = (–111.7) – (–111.8) (m).

IR (ATR): $\tilde{\nu}$ = 2924, 2855, 1657, 1599, 1490, 1446, 1281, 1092, 713 cm^{-1}.

MS (EI) *m/z* (relative intensity): 296 (92) $[M]^+$, 226 (68), 149 (53), 105 (100).

HR-MS (ESI): *m/z* calcd for $C_{20}H_{22}FO^+$ $[M+H]^+$ 297.1649, found 297.1654.

(3-Cycloheptyl-4-fluorophenyl)(3-cycloheptylphenyl)methanone (**3rf'**)

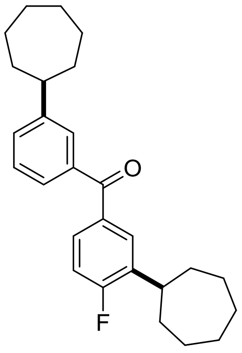

^{1}H-NMR (600 MHz, $CDCl_3$): δ = 7.74 (dd, J = 7.3, 2.3 Hz, 1H), 7.62 (ddd, J = 8.5, 5.0, 2.3 Hz, 1H), 7.60–7.59 (m, 1H), 7.56 (ddd, J = 7.4, 1.6, 1.6 Hz, 1H), 7.42 (ddd, J = 7.6, 1.6, 1.6 Hz, 1H), 7.38 (dd, J = 7.6, 7.4 Hz, 1H), 7.08 (dd, J = 9.9, 8.5 Hz, 1H), 3.06 (tt, J = 10.7, 3.5 Hz, 1H), 2.74 (tt, J = 10.7, 3.6 Hz, 1H), 1.97–1.89 (m, 4H), 1.85–1.77 (m, 4H), 1.74–1.65 (m, 8H), 1.63–1.51 (m, 8H).

^{13}C-NMR (100 MHz, $CDCl_3$): δ = 195.9 (C_q), 162.7 (d, $^{1}J_{C\text{-}F}$ = 252 Hz, C_q), 150.1 (C_q), 137.7 (C_q), 136.5 (d, $^{2}J_{C\text{-}F}$ = 16 Hz, C_q), 133.8 (d, $^{4}J_{C\text{-}F}$ = 3 Hz, C_q), 130.9 (CH), 130.6 (d, $^{3}J_{C\text{-}F}$ = 7 Hz, CH), 129.6 (d, $^{3}J_{C\text{-}F}$ = 10 Hz, CH), 128.3 (CH), 128.2 (CH), 127.3 (CH), 115.2 (d, $^{2}J_{C\text{-}F}$ = 24 Hz, CH), 46.9 (CH), 39.3 (CH), 36.8 (CH_2), 35.3 (CH_2), 27.8 (CH_2), 27.8 (CH_2), 27.3 (CH_2), 27.2 (CH_2).

^{19}F-NMR (282 MHz, $CDCl_3$): δ = (–112.1) – (–112.2) (m).

IR (ATR): $\tilde{\nu}$ = 2919, 1657, 1583, 1284, 1246, 1170, 1091, 833, 806, 755 cm^{-1}.

MS (EI) *m/z* (relative intensity): 392 (100) [M]$^+$, 310 (22), 219 (57), 201 (22), 149 (24), 55 (19).

HR-MS (EI): *m/z* calcd for $C_{27}H_{33}FO^+$ [M]$^+$ 392.2510, found 392.2523.

The interconversion of geometric isomers 1r

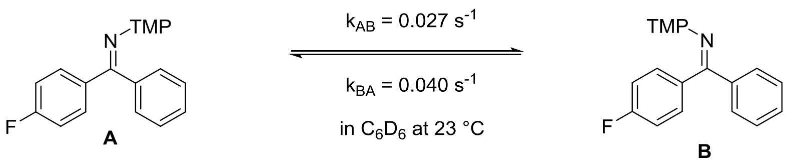

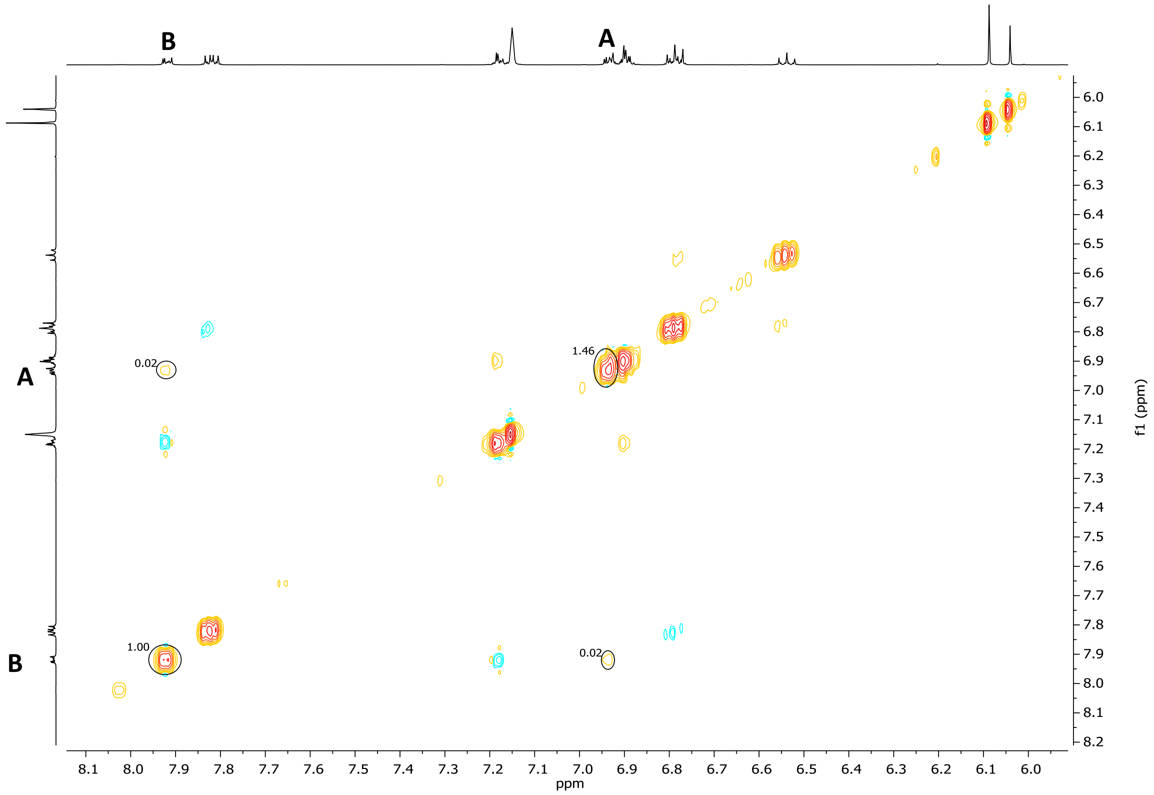

Supplementary Figure 3: NOESY-NMR of interconversion of geometric isomers **1r**.

meta-C–H Alkylation in the presence of radical scavengers

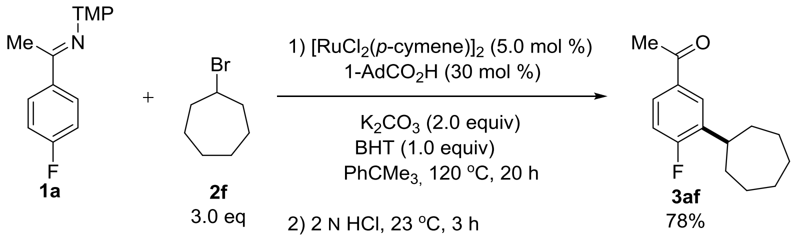

Figure 4(*i*): *meta*-C–H Alkylation in the presence of BHT.

Ketimine **1a** (152 mg, 0.50 mmol), [RuCl$_2$(*p*-cymene)]$_2$ (15.3 mg, 25.0 µmol, 5.0 mol %), 1-AdCO$_2$H (**4**) (27.3 mg, 0.15 mmol, 30 mol %), K_2CO_3 (138 mg, 1.00 mmol) and BHT (110.2 mg, 0.50 mmol) were placed in a pre-dried 25 mL pressure tube. The tube was evacuated and purged with N_2 for three times. Alkyl bromide **2f** (266 mg, 1.50 mmol) and PhCMe$_3$ (2.0 mL) were then added and the mixture was stirred at 120 °C for 20 h. At ambient temperature, HCl (2 N, 3.0 mL) was added, and the resulting mixture was stirred for additional 3 h, and then extracted with Et$_2$O (3 × 20 mL). The combined organic layers were dried over Na_2SO_4 and concentrated *in vacuo*. Purification of the residue by column chromatography (*n*-pentane/Et$_2$O 50:1) yielded adduct **3af** (91 mg, 78%) as a colorless oil.

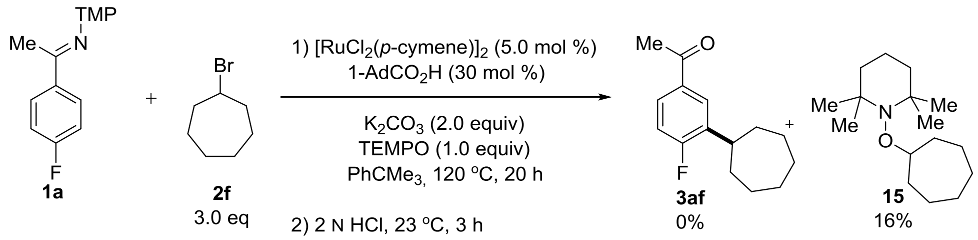

Figure 4(*i*): *meta*-C–H Alkylation in the presence of TEMPO.

Ketimine **1a** (152 mg, 0.50 mmol), [RuCl$_2$(*p*-cymene)]$_2$ (15.3 mg, 25.0 µmol, 5.0 mol %), 1-AdCO$_2$H (**4**) (27.3 mg, 0.15 mmol, 30 mol %), K_2CO_3 (138 mg, 1.00 mmol) and TEMPO (78.5 mg, 0.50 mmol) were placed in a pre-dried 25 mL pressure tube. The tube was evacuated and purged with N_2 for three times. Alkyl bromide **2f** (1.50 mmol) and PhCMe$_3$ (2.0 mL) were then added and the mixture was stirred at 120 °C for 20 h. At ambient temperature, HCl (2 N, 3.0 mL) was added, and the resulting mixture was stirred for additional 3 h, and then extracted with Et$_2$O (3 × 20 mL). The combined organic layers were dried over Na_2SO_4 and concentrated *in vacuo*. Purification of the residue by column chromatography (*n*-pentane/Et$_2$O 50:1) yielded TEMPO-adduct **15** (20 mg, 16%) as a colorless oil.

1-(Cycloheptyloxy)-2,2,6,6-tetramethylpiperidine (15)

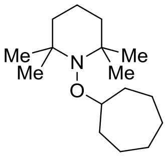

^{1}H-NMR (300 MHz, $CDCl_3$): δ = 3.82 (tt, J = 8.5, 4.4 Hz, 1H), 2.07–1.95 (m, 2H), 1.71–1.41 (m, 13H), 1.40–1.22 (m, 3H), 1.11 (s, 12H).

^{13}C-NMR (75 MHz, $CDCl_3$): δ = 83.8 (CH), 59.7 (C_q), 40.3 (CH_2), 34.4 (CH_3), 33.5 (CH_2), 28.6 (CH_2), 23.4 (CH_2), 20.4 (CH_3), 17.3 (CH_2).

IR (ATR): $\tilde{\nu}$ = 2924, 2856, 1458, 1359, 1132, 1006, 973 cm^{-1}.

MS (ESI) m/z (relative intensity): 254 (100) $[M+H]^+$, 126 (33).

HR-MS (ESI): m/z calcd for $C_{16}H_{32}NO^+$ $[M+H]^+$ 254.2478, found 254.2479.

The spectral data are in accordance with those reported in the literature.[12]

meta-C–H Alkylation with enantiomerically enriched alkyl bromide 2m

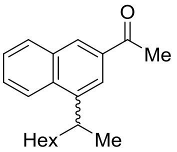

The general procedure **A** was followed using substrate **1l** (34 mg, 0.10 mmol) and bromide (*S*)-**2m** (84% ee, 58 mg, 0.30 mmol). After 20 h, purification by column chromatography (*n*-pentane/Et_2O 20:1) yielded *rac*-**3lm** (0% ee, 23 mg, 78%) as a colorless oil.

HPLC (*n*-hexane/*i*-PrOH 99:1): R_{t1} = 10.33 min (area: 49.99%), R_{t2} = 11.97 min (area: 50.01%).

meta-C–H Alkylation with diastereomerically pure alkyl bromides 2p

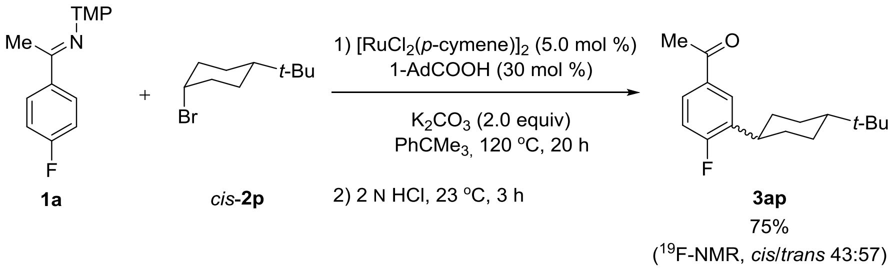

Figure 4(*iii*): *meta*-C–H Alkylation with *cis*-**2p**.

The general procedure **A** was followed using substrate **1a** (152 mg, 0.50 mmol) and bromide *cis*-**2p** (329 mg, 1.50 mmol). After 20 h, purification by column chromatography (*n*-pentane/Et_2O 50:1) yielded **3ap** as a mixture of *cis*- and *trans*-isomers (*cis*-**3ap**/*trans*-**3ap** 43:57, 104 mg, 75%).

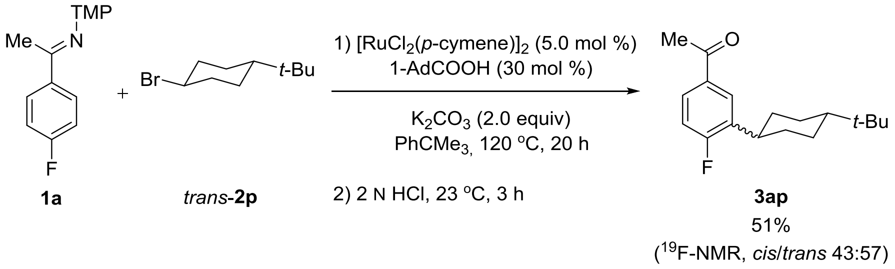

Figure 4(*iii*): *meta*-C–H Alkylation with *trans*-**2p**.

The general procedure **A** was followed using substrate **1a** (152 mg, 0.50 mmol) and bromide *trans*-**2p** (329 mg, 1.50 mmol). After 20 h, purification by column chromatography (*n*-pentane/Et_2O 50:1) yielded **3ap** as a mixture of *cis*- and *trans*-isomers (*cis*-**3ap**/*trans*-**3ap** 43:57, 71 mg, 51%).

1-{3-[*trans*-4-(*tert*-Butyl)cyclohexyl]-4-fluorophenyl}ethan-1-one (*trans*-3ap)

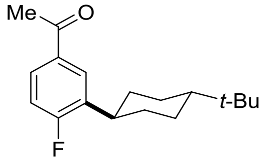

White solid.

^{1}H-NMR (300 MHz, $CDCl_3$): δ = 7.88 (dd, J = 7.1, 2.3 Hz, 1H), 7.77 (ddd, J = 8.5, 4.9, 2.3 Hz, 1H), 7.05 (dd, J = 9.9, 8.5 Hz, 1H), 2.83 (tt, J = 12.2, 3.4 Hz, 1H), 2.58 (s, 3H), 2.00–1.86 (m, 4H), 1.60–1.42 (m, 2H), 1.28–1.05 (m, 3H), 0.89 (s, 9H).

^{13}C-NMR (125 MHz, $CDCl_3$): δ = 196.7 (C_q), 163.7 (d, $^1J_{C\text{-}F}$ = 253 Hz, C_q), 134.7 (d, $^2J_{C\text{-}F}$ = 16 Hz, C_q), 133.5 (d, $^4J_{C\text{-}F}$ = 3 Hz, C_q), 128.3 (d, $^3J_{C\text{-}F}$ = 7 Hz, CH), 128.0 (d, $^3J_{C\text{-}F}$ = 10 Hz, CH), 115.4 (d, $^2J_{C\text{-}F}$ = 24 Hz, CH), 47.7 (CH), 37.2 (d, $^3J_{C\text{-}F}$ = 2 Hz, CH), 33.3 (CH_2), 32.5 (C_q), 27.6 (CH_3), 27.6 (CH_2), 26.6 (CH_3).

^{19}F-NMR (282 MHz, $CDCl_3$): δ = –111.5 (ddd, J = 9.9, 7.1, 4.9 Hz).

IR (ATR): $\tilde{\nu}$ = 2940, 2859, 1681, 1606, 1491, 1354, 1281, 1116, 825, 570 cm^{-1}.

m.p.: 54–55 °C.

MS (EI) *m*/*z* (relative intensity): 276 (32) $[M]^+$, 261 (11) $[M–Me]^+$, 220 (71) $[M–Bu]^+$, 205 (100), 177 (25), 151 (34), 57 (94), 43 (96).

HR-MS (EI): *m*/*z* calcd for $C_{18}H_{25}FO^+$ $[M]^+$ 276.1884, found 276.1896.

1-{3-[*cis*-4-(*tert*-Butyl)cyclohexyl]-4-fluorophenyl}ethan-1-one (*cis*-3ap)

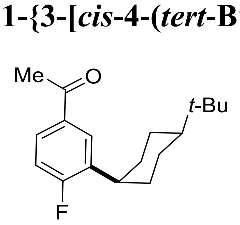

Colorless oil.

^{1}H-NMR (300 MHz, $CDCl_3$): δ = 8.11 (ddd, J = 7.6, 2.3, 0.8 Hz, 1H), 7.79 (dddd, J = 8.5, 4.8, 2.3, 0.5 Hz, 1H), 7.05 (dd, J = 11.0, 8.5 Hz, 1H), 3.35–3.26 (m, 1H), 2.59 (s, 3H), 2.17–2.07 (m, 2H), 1.88–1.73 (m, 2H),

1.72–1.60 (m, 2H), 1.26 (qd, J = 12.1, 3.5 Hz, 2H), 1.14 (tt, J = 11.2, 3.0 Hz, 1H), 0.85 (s, 9H).

^{13}C-NMR (125 MHz, $CDCl_3$): δ = 196.7 (C_q), 164.2 (d, $^1J_{C\text{-}F}$ = 254 Hz, C_q), 133.8 (d, $^2J_{C\text{-}F}$ = 14 Hz, C_q), 132.9 (d, $^4J_{C\text{-}F}$ = 3 Hz, C_q), 129.7 (d, $^3J_{C\text{-}F}$ = 6 Hz, CH), 128.0 (d, $^3J_{C\text{-}F}$ = 10 Hz, CH), 115.4 (d, $^2J_{C\text{-}F}$ = 24 Hz, CH), 47.4 (CH), 32.7 (C_q), 31.1 (CH), 29.9 (CH_2), 29.9 (CH_2), 27.6 (CH_3), 26.6 (CH_3), 23.3 (CH_2).

^{19}F-NMR (282 MHz, $CDCl_3$): δ = –107.9 (ddd, J = 11.0, 7.6, 4.8 Hz).

IR (ATR): $\tilde{\nu}$ = 2941, 2866, 1687, 1584, 1491, 1357, 1254, 1112, 828, 575 cm^{-1}.

MS (EI) *m*/*z* (relative intensity): 276 (2) $[M]^+$, 261 (3) $[M–Me]^+$, 220 (29) $[M–Bu]^+$, 205 (23), 177 (14), 149 (10), 57 (26), 43 (100).

HR-MS (EI): *m*/*z* calcd for $C_{18}H_{25}FO^+$ $[M]^+$ 276.1884, found 276.1884.

Kinetic Analysis (Figure 5)

Order with respect to [Ru(*p*-cymene)($O_2CAd)_2$] (6)

The reaction order with respect to [Ru(*p*-cymene)($O_2CAd)_2$] (**6**) was examined using the initial rate method.[13] Inside a glovebox, a Schlenk-tube was charged with (*E*)-1-(4-fluorophenyl)-*N*-(3,4,5-trimethoxyphenyl)ethan-1-imine (**1a**) (303 mg, 1.00 mmol), 1-bromo-1-methylcyclohexane (**2a**) (531 mg, 3.00 mmol), K_2CO_3 (276 mg, 2.00 mmol) and 1-fluorononane (37 mg, 0.25 mmol). A solution of [Ru(*p*-cymene)($O_2CAd)_2$] (**6**) (2.5, 3.5, 7.5, 10.0 mol %) in $PhCMe_3$ (4.0 mL) was added and the mixture was stirred at 92 °C. Periodically aliquots (0.1 mL) were removed via a syringe, diluted with PhMe-d_8 (0.25 mL) and analyzed by $^{19}F\{^1H\}$-NMR spectroscopy.

**6** / mol %	Δ[**8aa**] Δt^{-1} / 10^{-8} mol L^{-1} s^{-1}	log(*c* / mol L^{-1})	log(Δ[**8aa**] Δt^{-1} / mol L^{-1} s^{-1})
2.5	0.945	–2.204	–8.024
3.5	1.515	–2.058	–7.820
7.5	3.377	–1.727	–7.471
10.0	5.041	–1.602	–7.298

Order with respect to the concentration of ketimine (1a)

The reaction order with respect to the concentration of ketimine (**1a**) was examined using the initial rate method.[13] Inside a glovebox, a Schlenk-tube was charged with (*E*)-1-(4-fluorophenyl)-*N*-(3,4,5-trimethoxyphenyl)ethan-1-imine (**1a**) (0.50, 0.75, 1.00, 1.50, 2.00 mmol), 1-bromo-1-methylcyclohexane (**2a**) (531 mg, 3.00 mmol), K_2CO_3 (276 mg, 2.00 mmol), 1-fluorononane (37 mg, 0.25 mmol) and MS (4 Å, 25 mg). A solution of [Ru(*p*-cymene)($O_2CAd)_2$] (**6**) (44.5 mg, 7.5 mol %) in $PhCMe_3$ (4.0 mL) was added and the mixture was stirred at 92 °C. Periodically aliquots (0.1 mL) were removed via a syringe, diluted with PhMe-d_8 (0.25 mL) and analyzed by $^{19}F\{^1H\}$-NMR spectroscopy.

**6** / mol %	Δ[**8aa**] Δt^{-1} / 10^{-8} mol L^{-1} s^{-1}	log(*c* / mol L^{-1})	log(Δ[**8aa**] Δt^{-1} / mol L^{-1} s^{-1})
0.50	1.883	–0.903	–7.725
0.75	2.384	–0.727	–7.623
1.00	3.775	–0.602	–7.423
1.50	4.909	–0.426	–7.309
2.00	6.600	–0.301	–7.180

Order with respect to the concentration of alkyl bromide (2a)

The reaction order with respect to the concentration of alkyl bromide (**2a**) was examined using the initial rate method.[13] Inside a glovebox, a Schlenk-tube was charged with (*E*)-1-(4-fluorophenyl)-*N*-(3,4,5-trimethoxyphenyl)ethan-1-imine (**1a**) (1.00 mmol), 1-bromo-1-methylcyclohexane (**2a**) (1.00, 1.50, 3.00, 4.50 mmol), K_2CO_3 (276 mg, 2.00 mmol) and 1-fluorononane (37 mg, 0.25 mmol). A solution of [Ru(*p*-cymene)($O_2CAd)_2$] (**6**) (44.5 mg, 7.5 mol %) in $PhCMe_3$ (4.0 mL) was added and the mixture was stirred at 92 °C. Periodically aliquots (0.1 mL) were removed via a syringe, diluted with PhMe-d_8 (0.25 mL) and analyzed by $^{19}F\{^1H\}$-NMR spectroscopy.

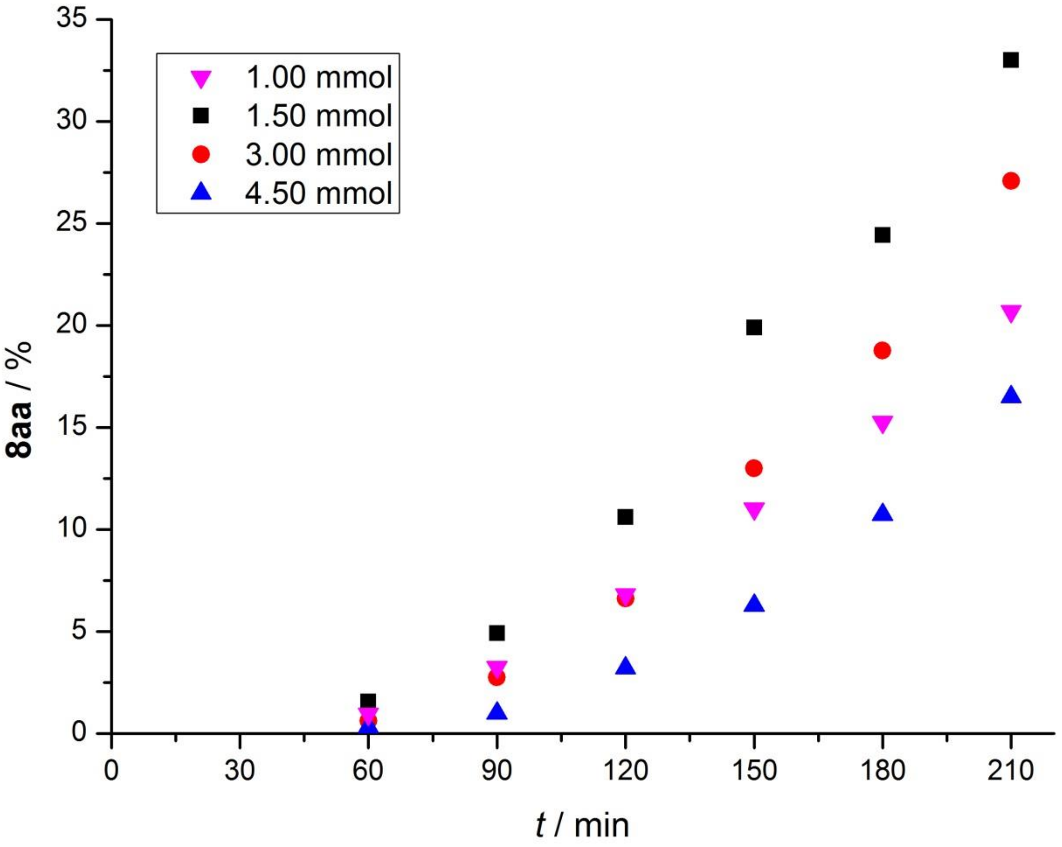

Supplementary Figure 4: Variation of concentration of alkyl bromide **2a**.

Determination of the activation energy

The activation energy was determined by an Arrhenius plot analysis. Inside a glovebox, a Schlenk-tube was charged with (*E*)-1-(4-fluorophenyl)-*N*-(3,4,5-trimethoxyphenyl)ethan-1-imine (**1a**) (303 mg, 1.00 mmol), 1-bromo-1-methylcyclohexane (**2a**) (531 mg, 3.00 mmol), K_2CO_3 (276 mg, 2.00 mmol) and 1-fluorononane (37 mg, 0.25 mmol). A solution of [Ru(*p*-cymene)($O_2CAd)_2$] (44.5 mg, 7.5 mol %) in $PhCMe_3$ (4.0 mL) was added and the mixture was stirred at the given temperatures (84, 92, 98, 103, 112 °C). Periodically aliquots

(0.1 mL) were removed via a syringe, diluted with PhMe-d_8 (0.25 mL) and analyzed by $^{19}F\{^1H\}$-NMR spectroscopy.

T / K	Δ**[8aa]** Δt^{-1} / 10^{-8} mol L^{-1} s^{-1}	T^{-1} / 10^{-3} K^{-1}	ln(Δ**[8aa]** Δt^{-1} / mol L^{-1} s^{-1})
357	1.303	2.80	–18.16
365	3.775	2.74	–17.09
371	7.277	2.69	–16.44
376	8.986	2.66	–16.23
385	15.02	2.60	–15.71

Reaction with Well-Defined Ruthenium (II)-complexes

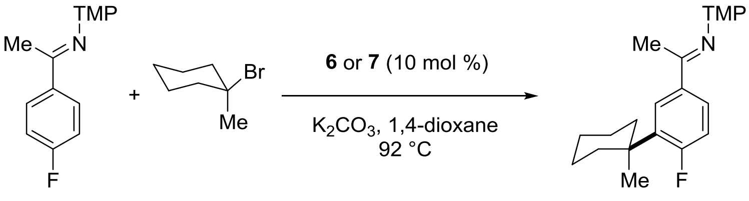

The reaction was set up in a glovebox, using ketimine **1a** (303 mg, 1.00 mmol, 1.0 equiv), [Ru(O_2CAd)$_2$(t-BuC_6H_5)] (**7**) (59.5 mg, 0.10 mmol, 10 mol %), 1-bromo-1-methylcyclohexane (**2a**) (531 mg, 3.00 mmol, 3.0 equiv), MS (4 Å, 25 mg) and 1-fluornonane (45 µL, 0.25 mmol). Periodically aliquots (0.1 mL) were removed via a syringe, diluted with PhMe-d_8 (0.25 mL) and analyzed by $^{19}F\{^1H\}$-NMR spectroscopy.

The reaction was set up in a glovebox, using ketimine **1a** (303 mg, 1.00 mmol, 1.0 equiv), [Ru(O_2CAd)$_2$(p-cymene)] (**6**) (59.4 mg, 0.10 mmol, 10 mol %) 1-bromo-1-methylcyclohexane (**7**) (531 mg, 3.00 mmol, 3.0 equiv), MS (4 Å, 25 mg) and 1-fluornonane (45 µL, 0.25 mmol). Periodically aliquots (0.1 mL) were removed via a syringe, diluted with PhMe-d_8 (0.25 mL) and analyzed by $^{19}F\{^1H\}$-NMR spectroscopy.

entry	t/h	t/min	**8aa** / %	**1a** / %	**8aa** / %	**1a** / %
			[Ru(O_2CAd)$_2$(t-BuC_6H_5)] (**7**)		[Ru(O_2CAd)$_2$(p-cymene)] (**6**)	
1	1	60	0	100	0	100
2	2	120	2.8	97	1.0	98
3	3	180	9.0	85	3.0	97
4	4	240	18	75	6.0	89
5	5	300	26	63	11	76
6	6	360	34	52	22	65
7	7	420	41	43	30	56

8	8	480	46	36	37	49
9	9	540	53	31	42	44
10	10	600	55	27	46	37
11	11	660	58	25	48	30
12	12	720	59	23	47	29
13	13	780	58	21	51	26
14	14	870	57	18	54	24
15	24	1440	58	19	54	19

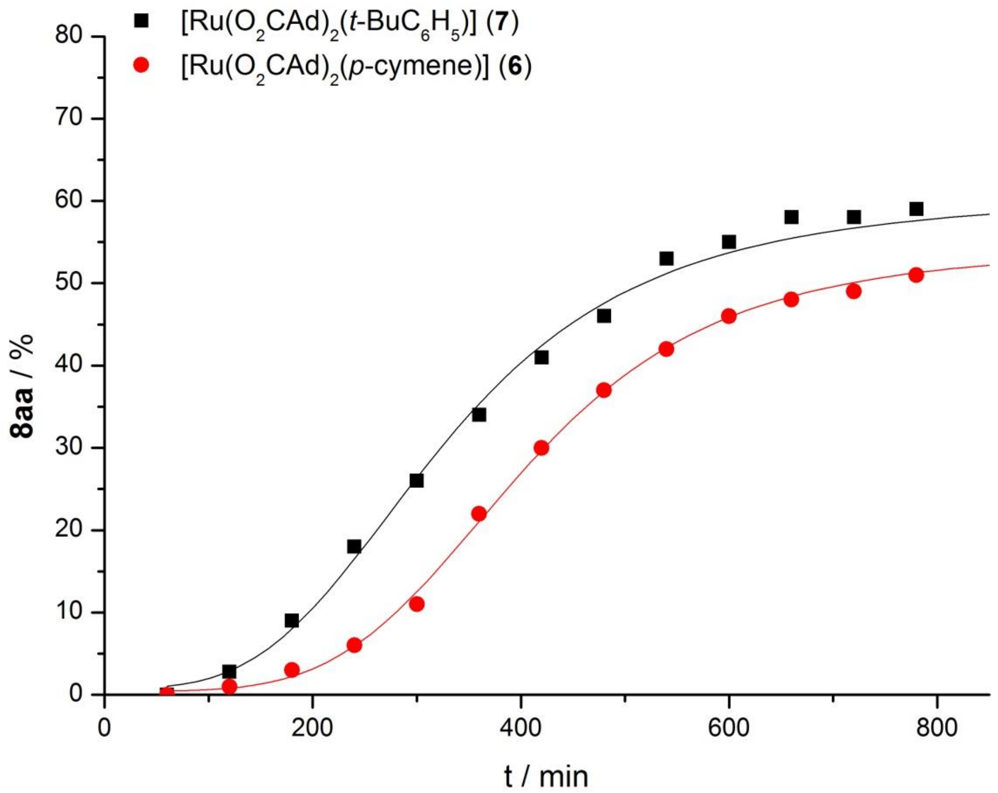

Figure 5(*iv*): Conversion to **8aa** with well-defined ruthenium (II)-complexes.

Supplementary Table 3: Robustness Test

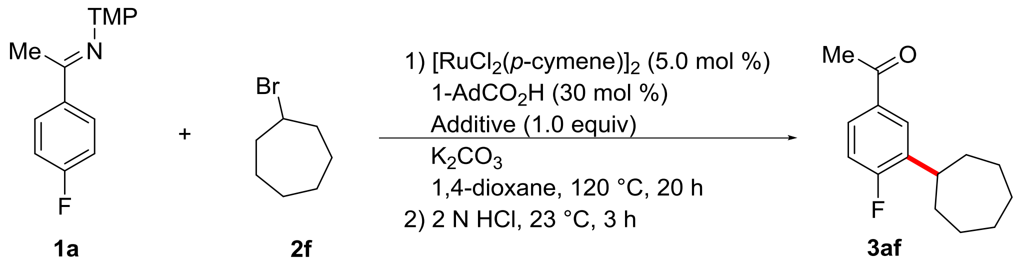

Entry	Additive	Yield of 3af[b]	Remaining additive[b]	Yield of Ketone[b]
1	none	97%		0%
2	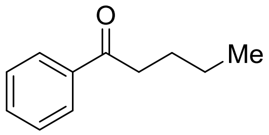	96%	93%	0%
3	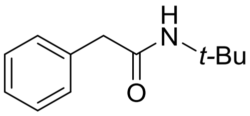	52%	45%	0%
4	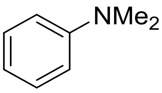	78%	0%	0%
5	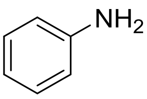	64%	0%	0%
6	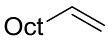	71%	nd[c]	0%
7	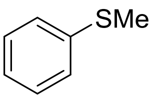	79%	0%	0%
8	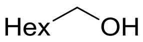	41%	77%	22%
9	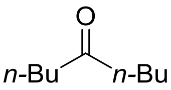	74%	96%	0%
10	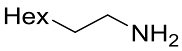	48%	0%	0%
11	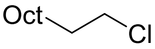	72%	100%	0%

12	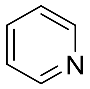	61%	nd[c]	0%
13	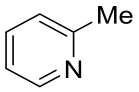	71%	0%	0%
14	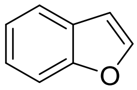	96%	91%	0%
15	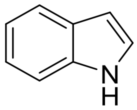	86%	41%	0%
16	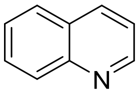	81%	0%	0%
17	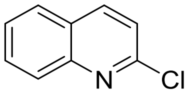	10%	0%	0%
18	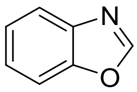	2%	10%	48%
19	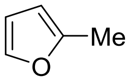	64%	nd[c]	0%
20	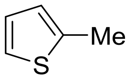	63%	88%	0%
21	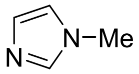	3%	0%	44%

[a] Reaction conditions: a) **1a** (0.5 mmol), **2f** (1.5 mmol), $[RuCl_2(p\text{-cymene})]_2$ (5.0 mol %), 1-AdCO$_2$H (30 mol %), additive (1.0 equiv), K_2CO_3 (1.0 mmol), PhCMe$_3$ (2 mL), 20 h, under N_2; b) HCl (2 N, 2.0 mL), 23 °C, 3 h. [b] Determined by GC with *n*-dodecane (40 µL) as the internal standard. [c] Not detected due to overlap with solvent signal.

Supplementary References

1. Masada, H., Murotani, Y. A Convenient Method for the Preparation of Highly Pure *t*-Alkyl Bromides and Iodides. *Bull. Chem. Soc. Jpn.* **53**, 1181–1182 (1980).
2. Shi, B.-F., Maugel, N., Zhang, Y.-H., Yu, J.-Q. Pd^{II}-Catalyzed Enantioselective Activation of C(sp^2)–H and C(sp^3)–H Bonds Using Monoprotected Amino Acids as Chiral Ligands. *Angew. Chem. Int. Ed.* **47**, 4882–4886 (2008).
3. Periasamy, M., Srinivas, G., Bharathi, P. Conversion of Aryl Methyl Ketimines to 2,5-Diarylpyrroles Using $TiCl_4/Et_3N$. *J. Org. Chem.* **64**, 4204–4205 (1999).
4. Rabideau, P. W., Marcinow, Z. The Birch Reduction of Aromatic Compounds. In *Organic Reactions.* Vol. **42** (Ed.: L. A. Paquette) (John Wiley & Sons, Inc., 2004)
5. Hodson, E., Simpson, S. J. Synthesis and characterisation of [(η^6-cymene)Ru(L)X_2] compounds: single crystal X-ray structure of [(η^6-cymene)Ru(P{OPh}$_3$)Cl_2] at 203 K. *Polyhedron* **23**, 2695–2707 (2004).
6. Berger, S. The t-butyl group as sensor group of the ortho effect. *Tetrahedron* **32**, 2451–2455 (1976).
7. Charpentier, B., Bernardon, J.-M., Eustache, J., Millois, C., Martin, B., Michel, S., Shroot, B. Synthesis, Structure-Affinity Relationships, and Biological Activities of Ligands Binding to Retinoic Acid Receptor Subtypes. *J. Med. Chem.* **38**, 4993–5006 (1995).
8. Nakamura, R., Obora, Y., Ishii, Y. Selective Oxidation of Acetophenones Bearing Various Functional Groups to Benzoic Acid Derivatives with Molecular Oxygen. *Adv. Synth. Catal.* **351**, 1677–1684 (2009).
9. Shen, C., Liu, R.-R., Fan, R.-J., Li, Y.-L., Xu, T.-F., Gao, J.-R., Jia, Y.-X. Enantioselective Arylative Dearomatization of Indoles via Pd-Catalyzed Intramolecular Reductive Heck Reactions. *J. Am. Chem. Soc.* **137**, 4936–4939 (2015).
10. Kaeding, W. W. The Halogenation of *meta-tert*-Butylphenol. *J. Org. Chem.* **26**, 4851–4855 (1961).
11. Mahajan, S., Sharma, B., Kapoor, K. K. A solvent-free one step conversion of ketones to amides via Beckmann rearrangement catalysed by $FeCl_3 \cdot 6H_2O$ in presence of hydroxylamine hydrochloride. *Tetrahedron Lett.* **56**, 1915–1918 (2015).
12. Root, K. S., Hill, C. L., Lawrence, L. M., Whitesides, G. M. The mechanism of formation of Grignard reagents: trapping of free alkyl radical intermediates by reaction with tetramethylpiperidine-N-oxyl. *J. Am. Chem. Soc.* **111**, 5405–5412 (1989).

13. Espenson, J. H., *Chemical Kinetics and Reaction Mechanisms*, *2nd ed*., McGraw-Hill, New York, **1995**.

Supplementary Figures

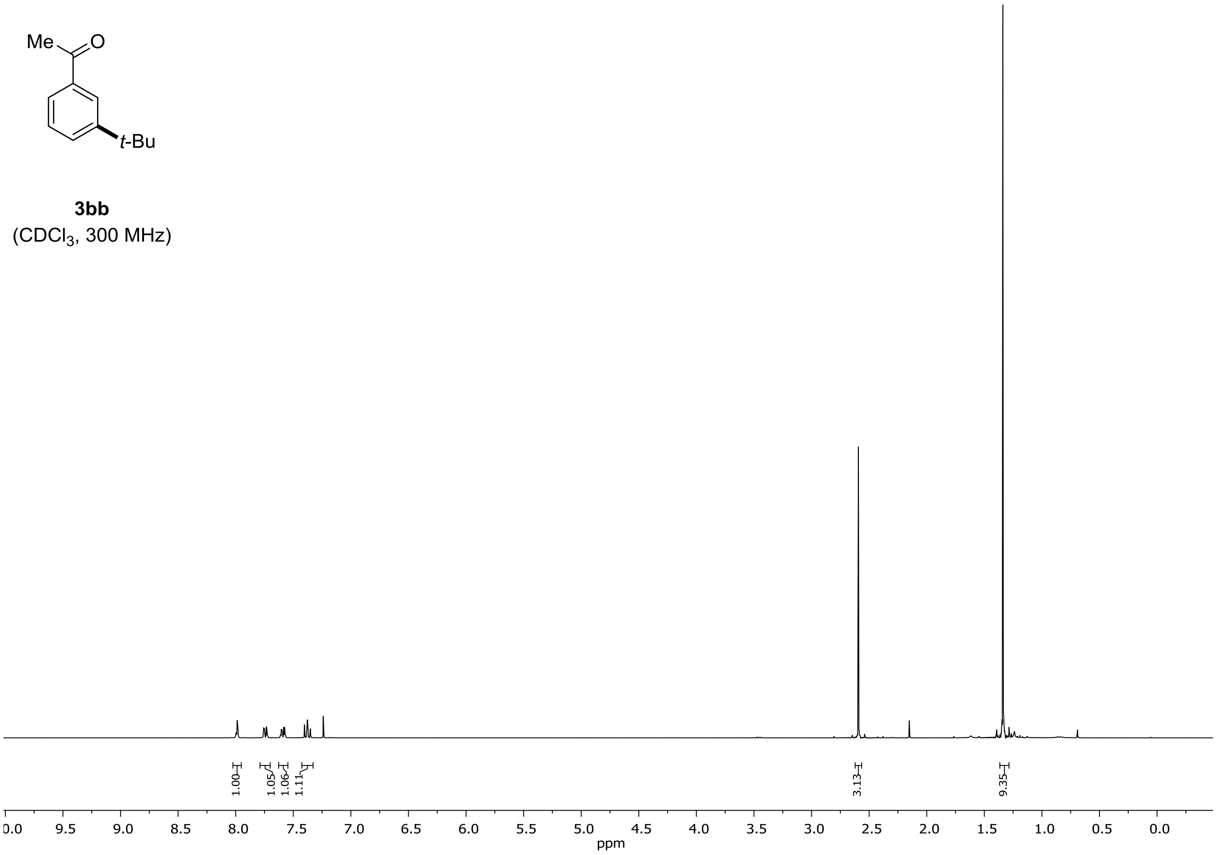

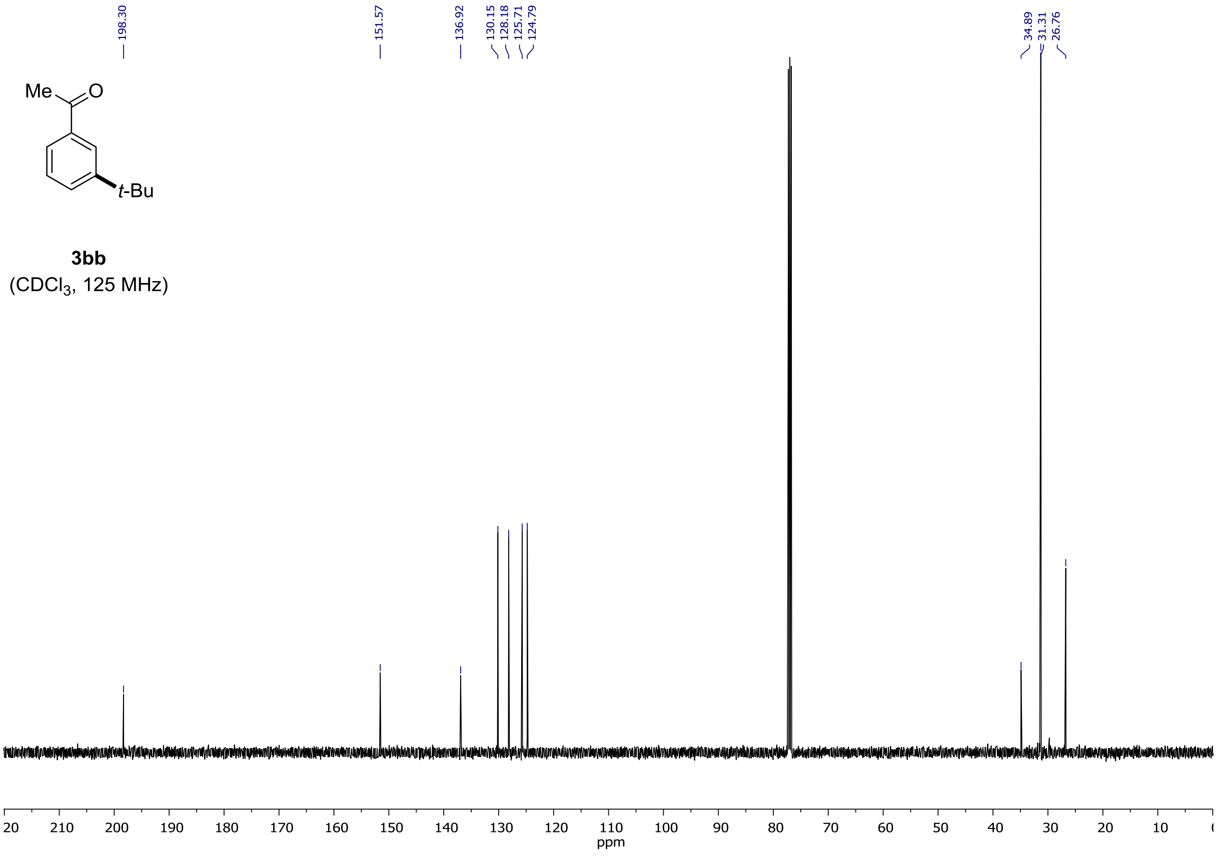

Supplementary Figure 5: ^{1}H and ^{13}C-NMR of Compound 3bb.

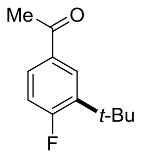

3ab
($CDCl_3$, 300 MHz)

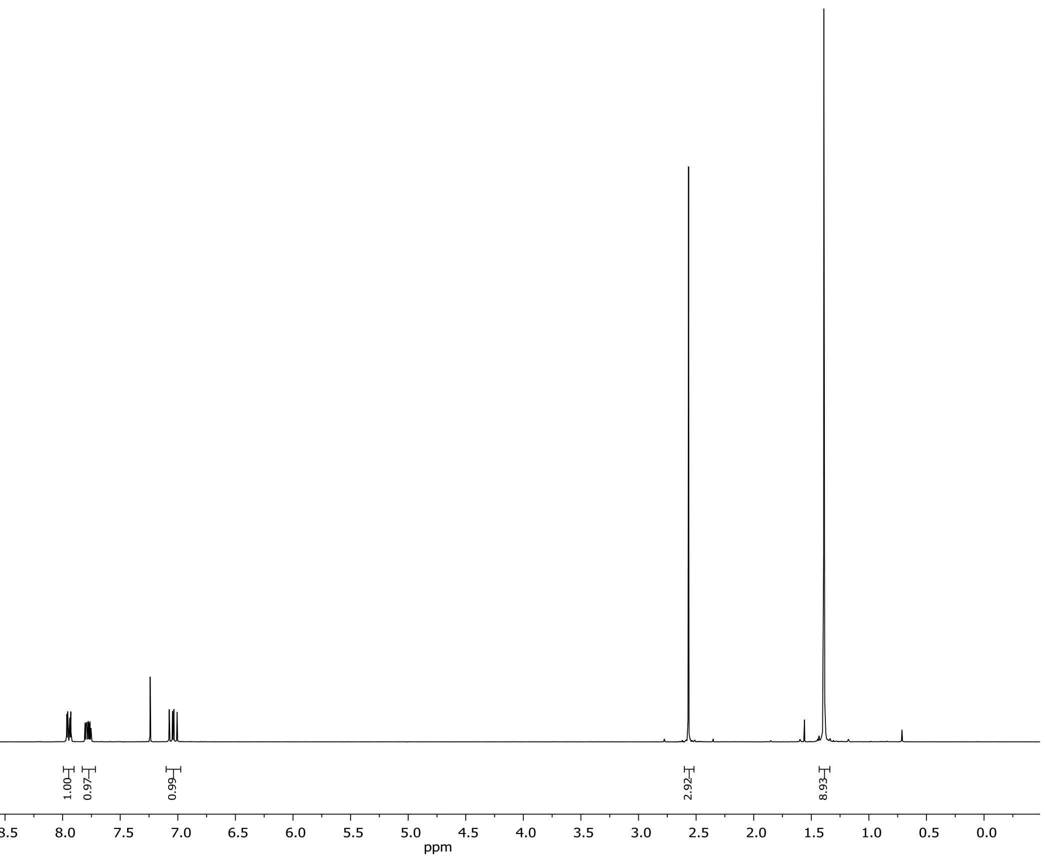

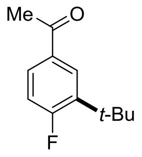

3ab
($CDCl_3$, 125 MHz)

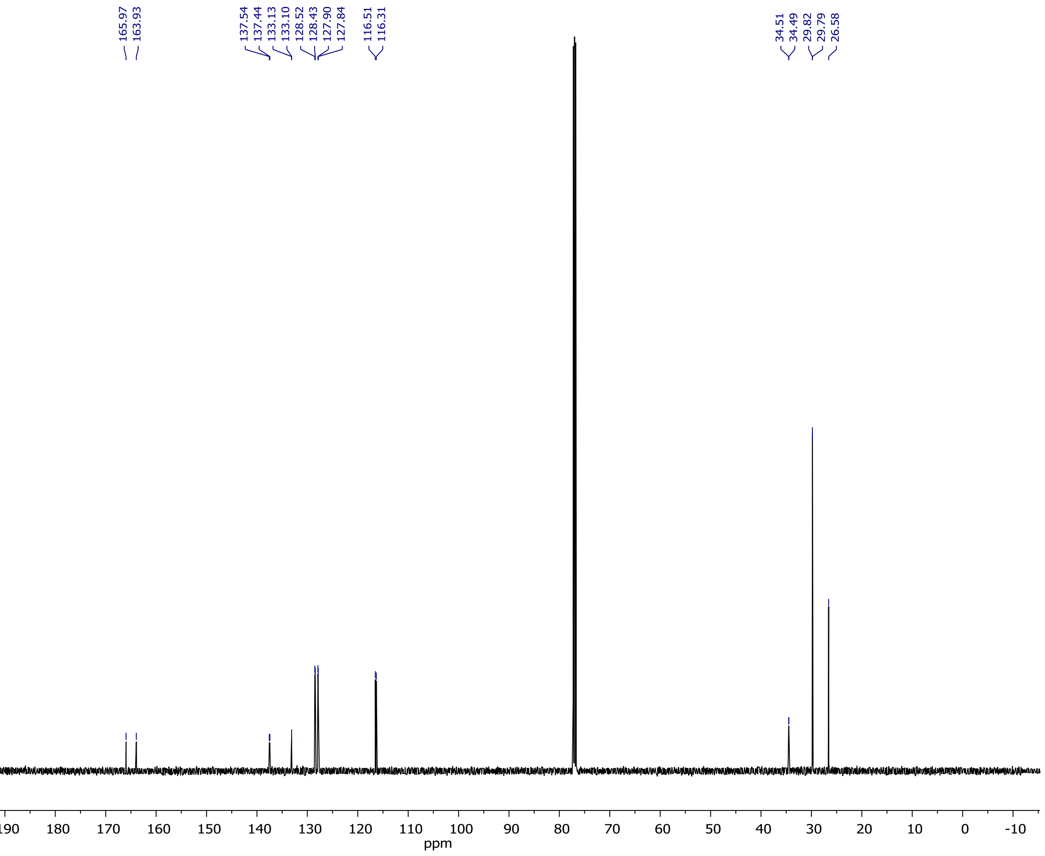

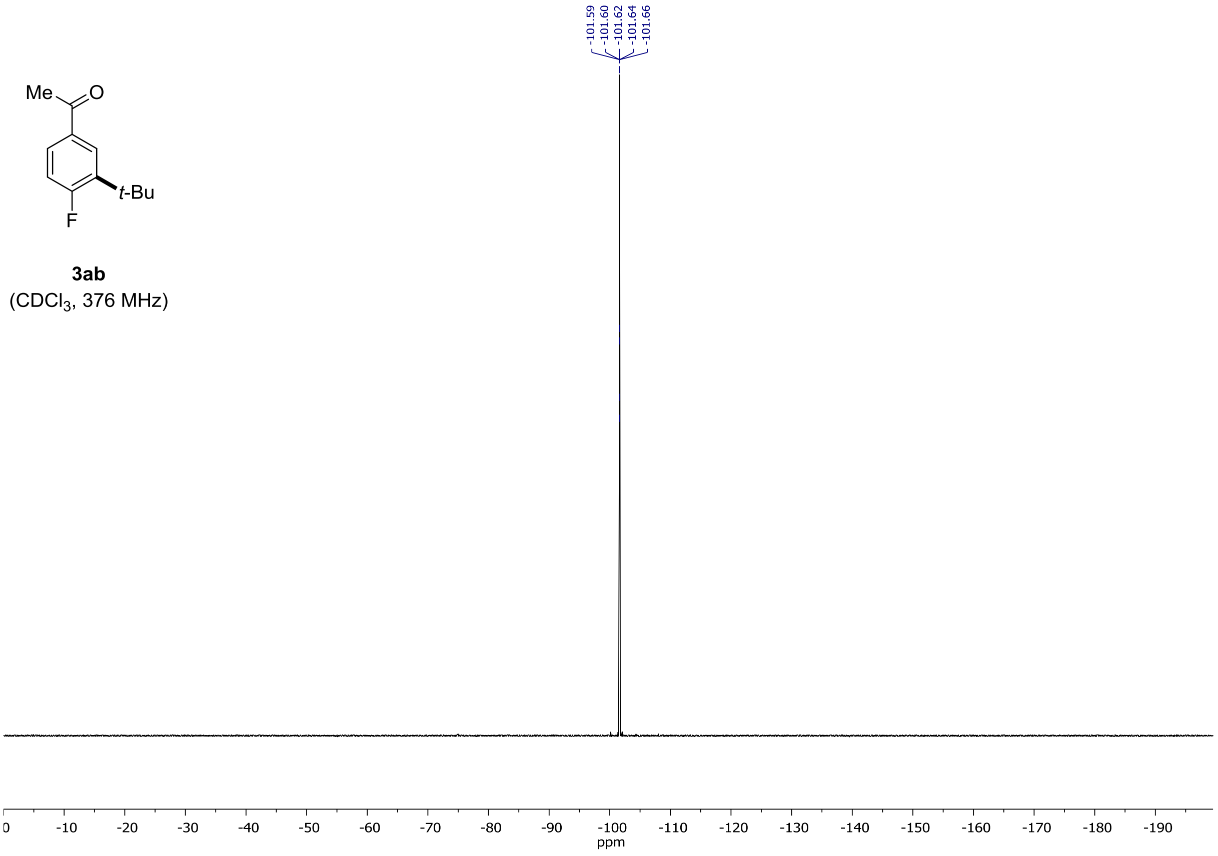
-101.59
-101.60
-101.62
-101.64
-101.66
Me
O
t-Bu
F
3ab
(CDCl3, 376 MHz)
0
-10
-20
-30
-40
-50
-60
-70
-80
-90
-100
-110
-120
-130
-140
-150
-160
-170
-180
-190
ppm

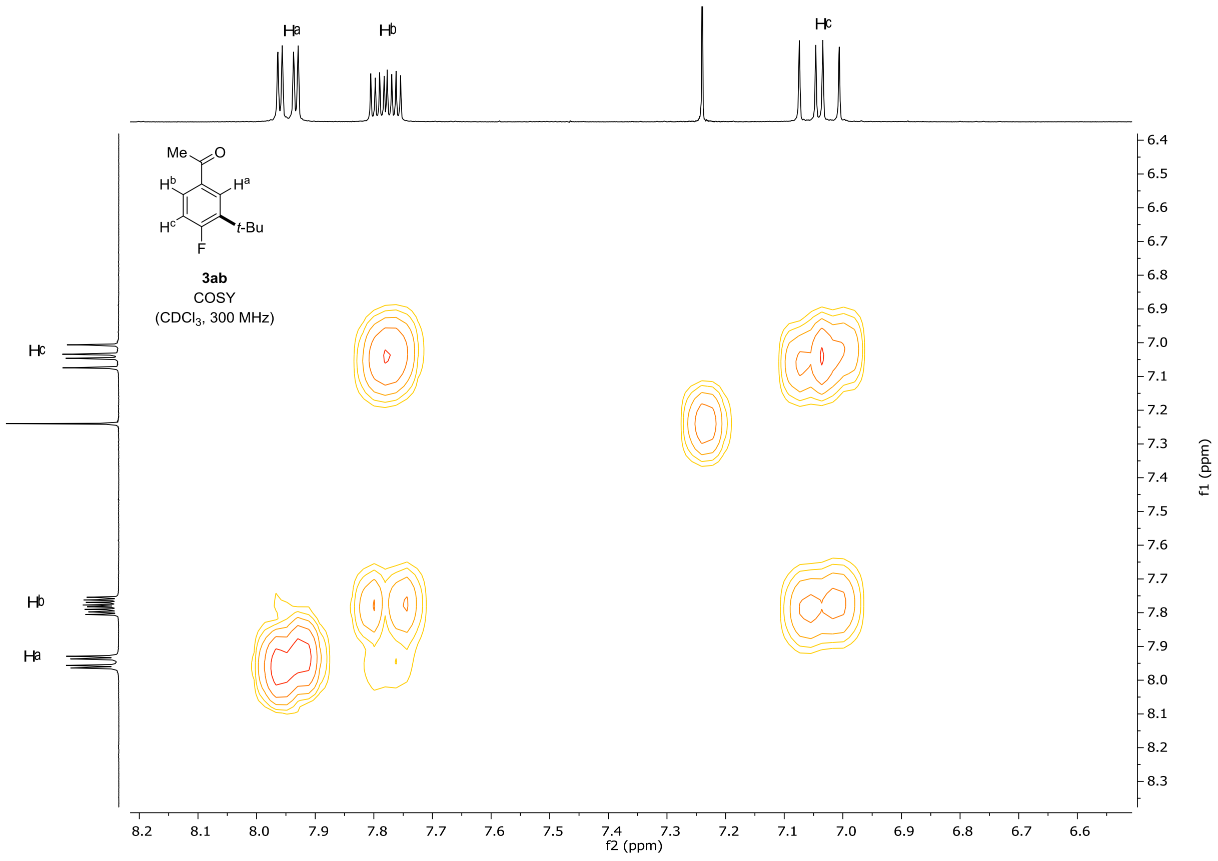
Ha
Hb
Hc
Me
O
Hb
Ha
Hc
t-Bu
F
3ab
COSY
(CDCl3, 300 MHz)
f1 (ppm)
f2 (ppm)

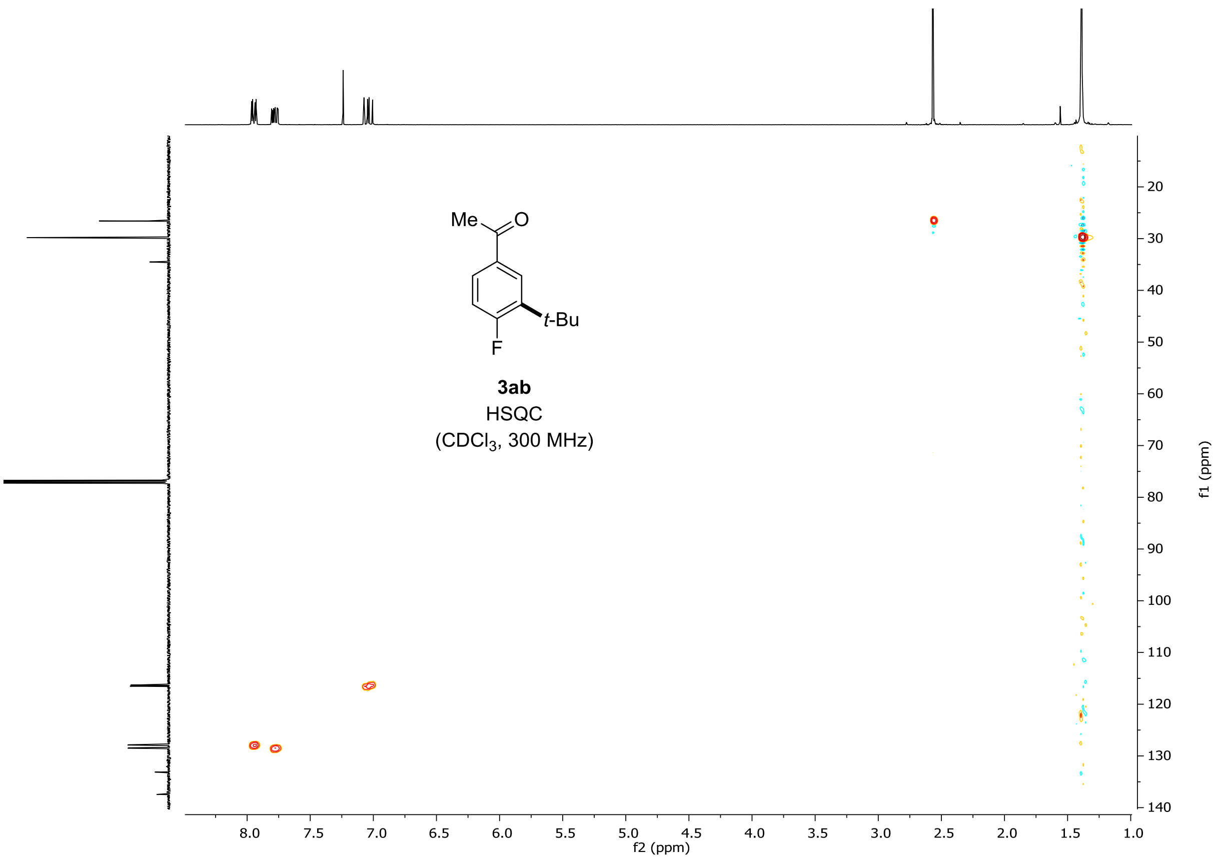

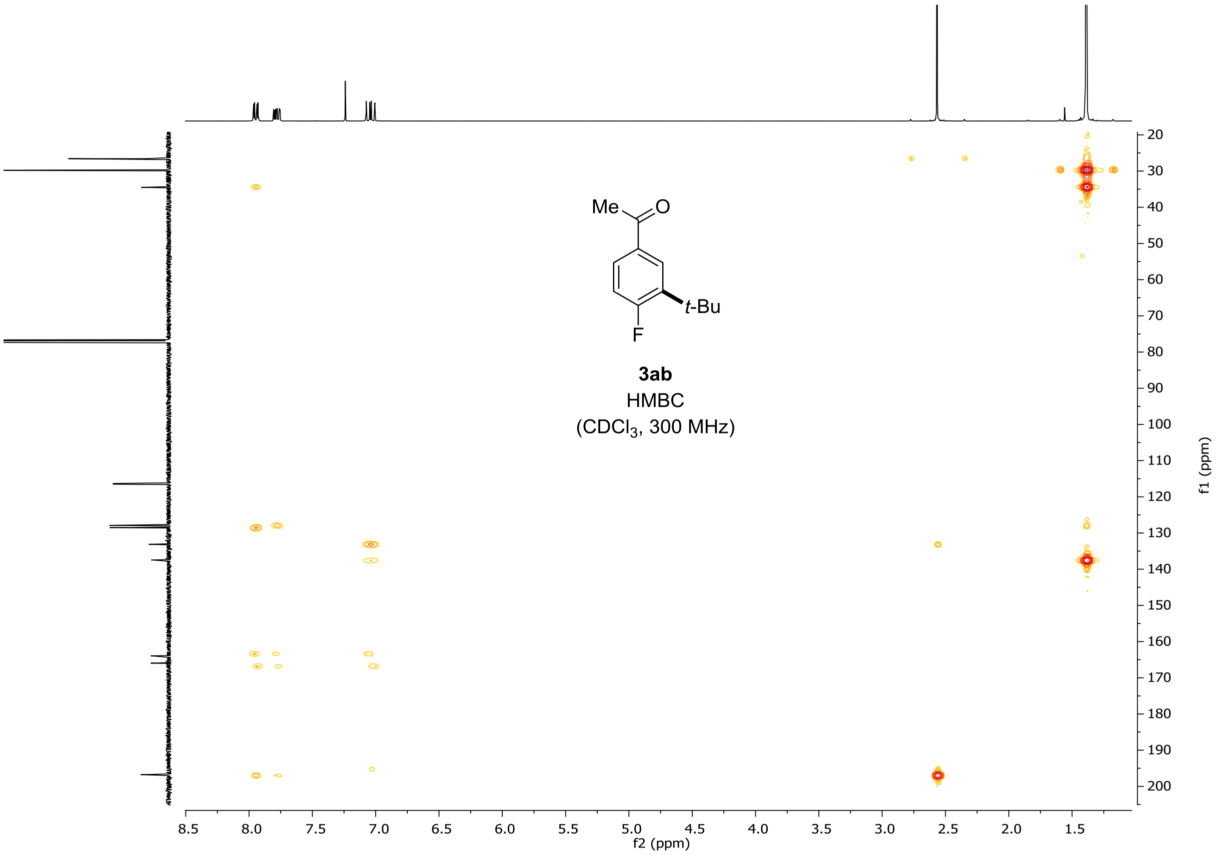

Supplementary Figure 6: ^{1}H, ^{13}C, ^{19}F, COSY, HSQC and HMBC-NMR of Compound 3ab.

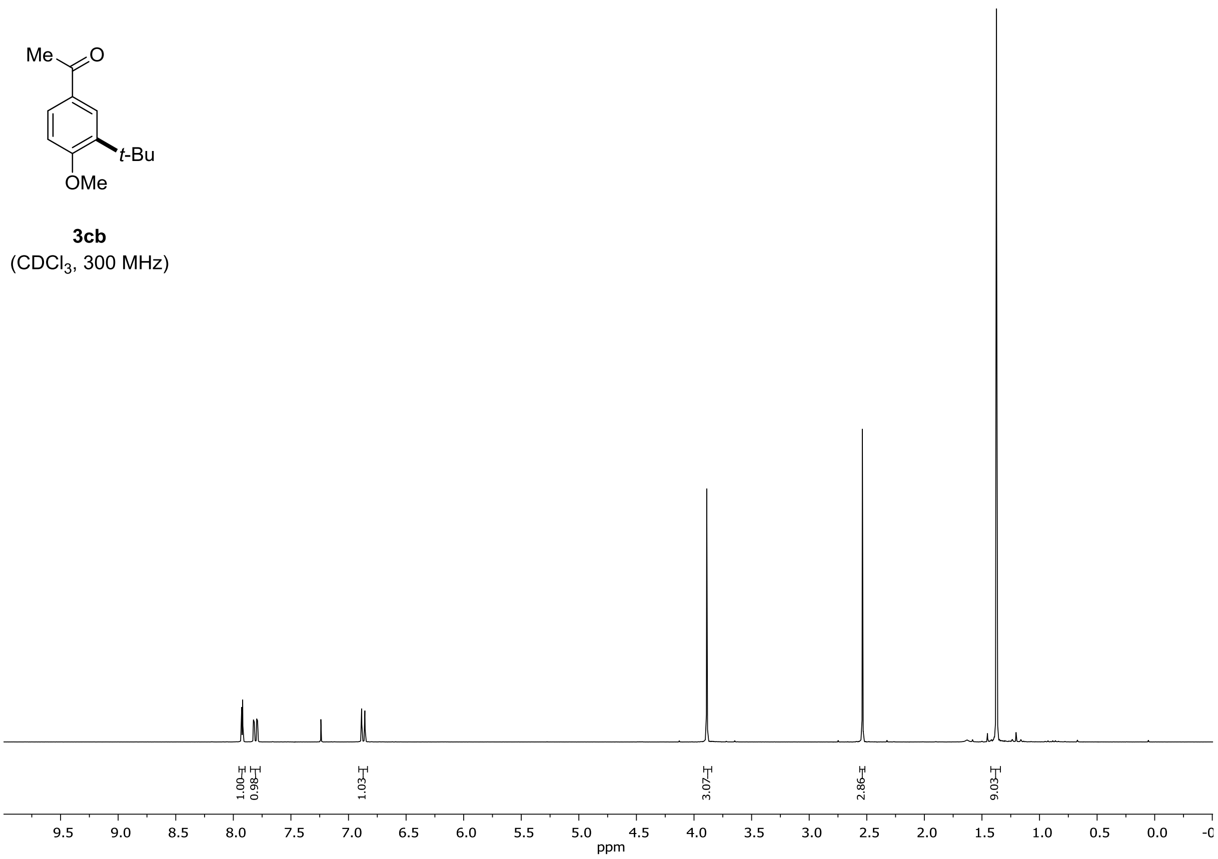

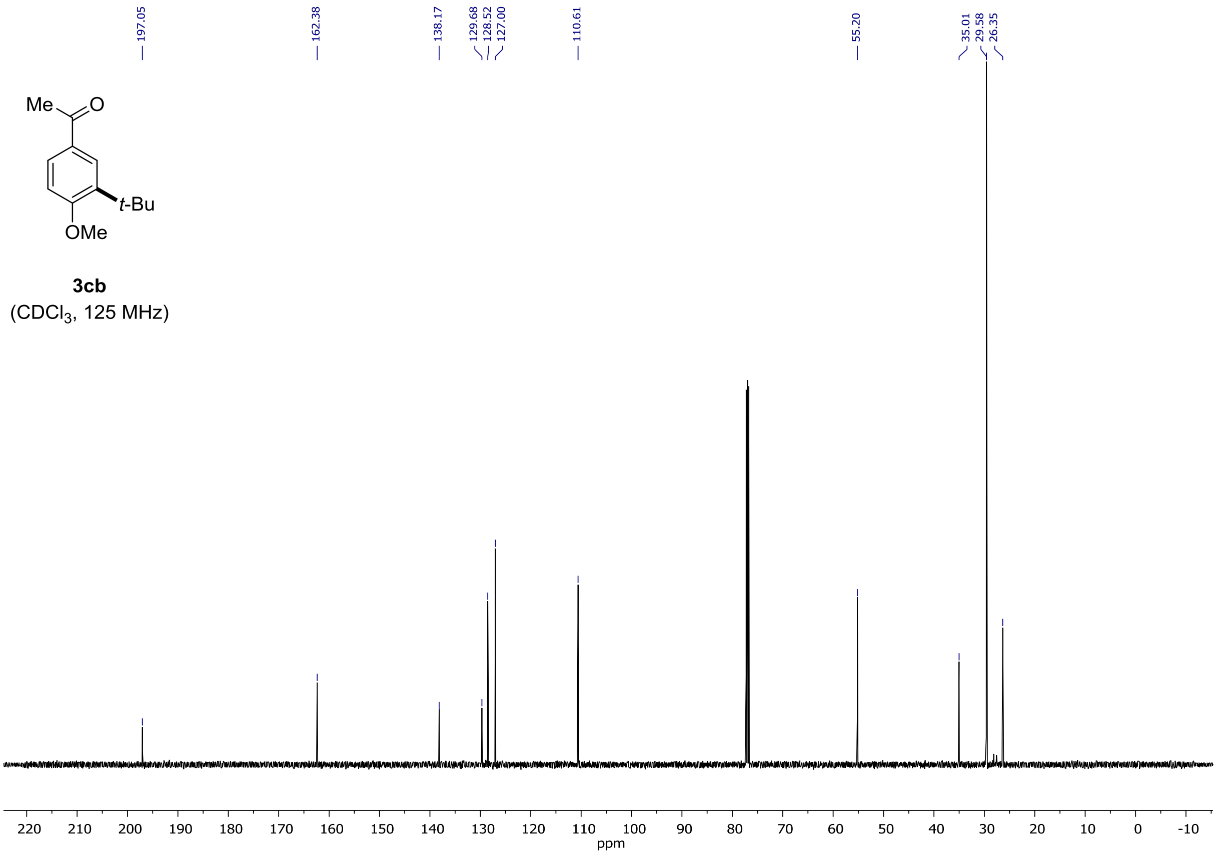

Supplementary Figure 7: ^{1}H and ^{13}C-NMR of Compound 3cb.

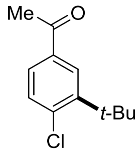

3db
($CDCl_3$, 300 MHz)

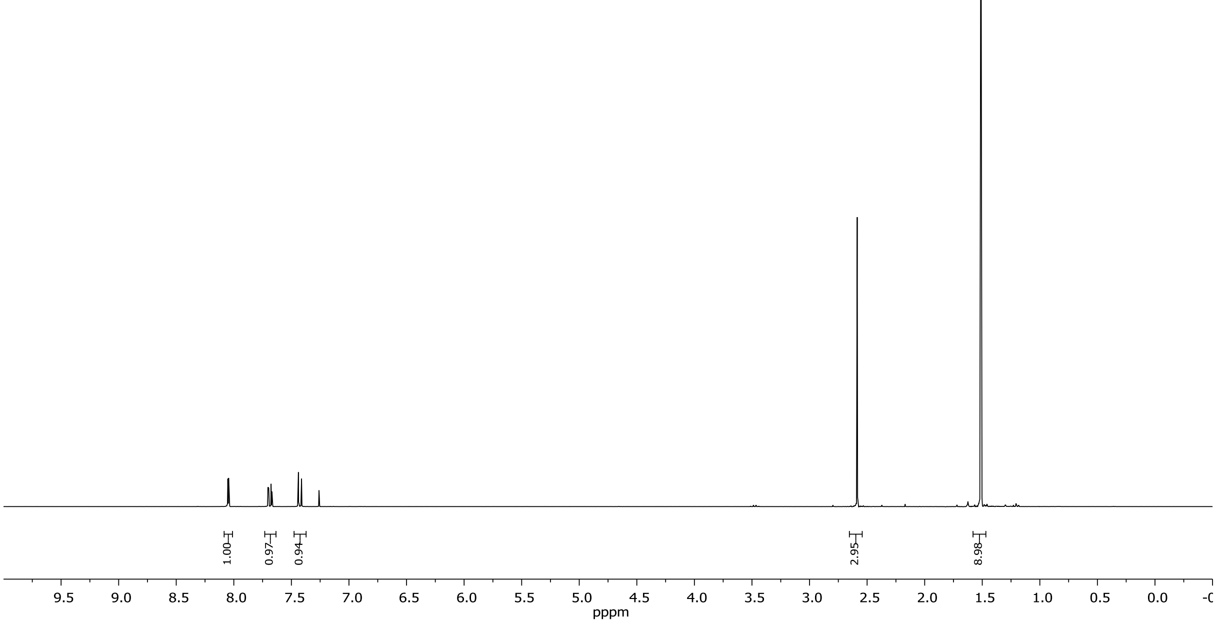

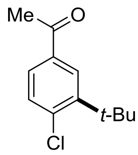

3db
($CDCl_3$, 125 MHz)

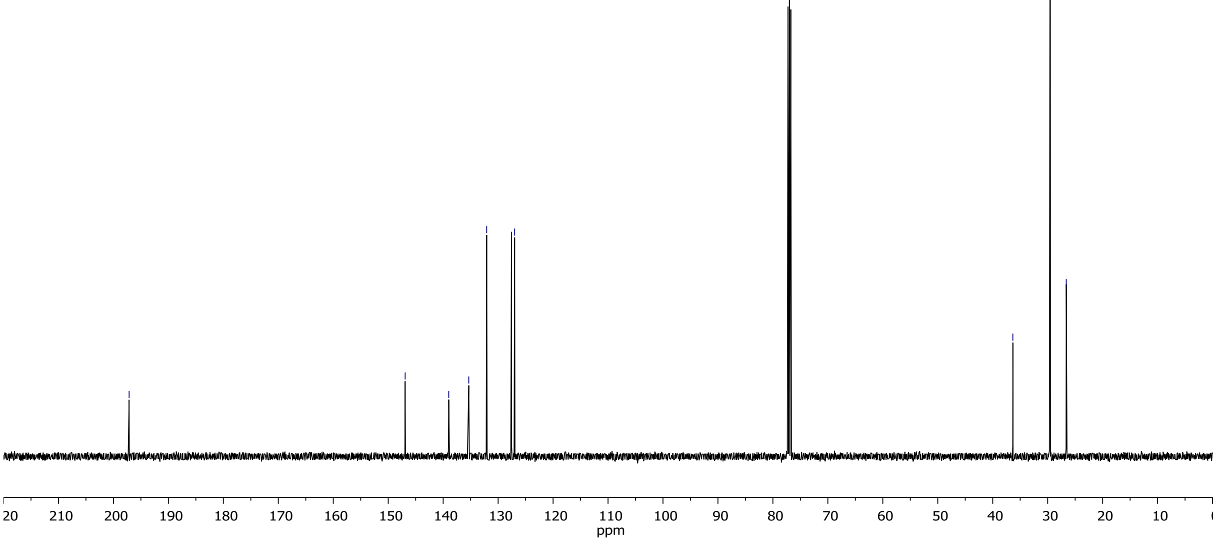

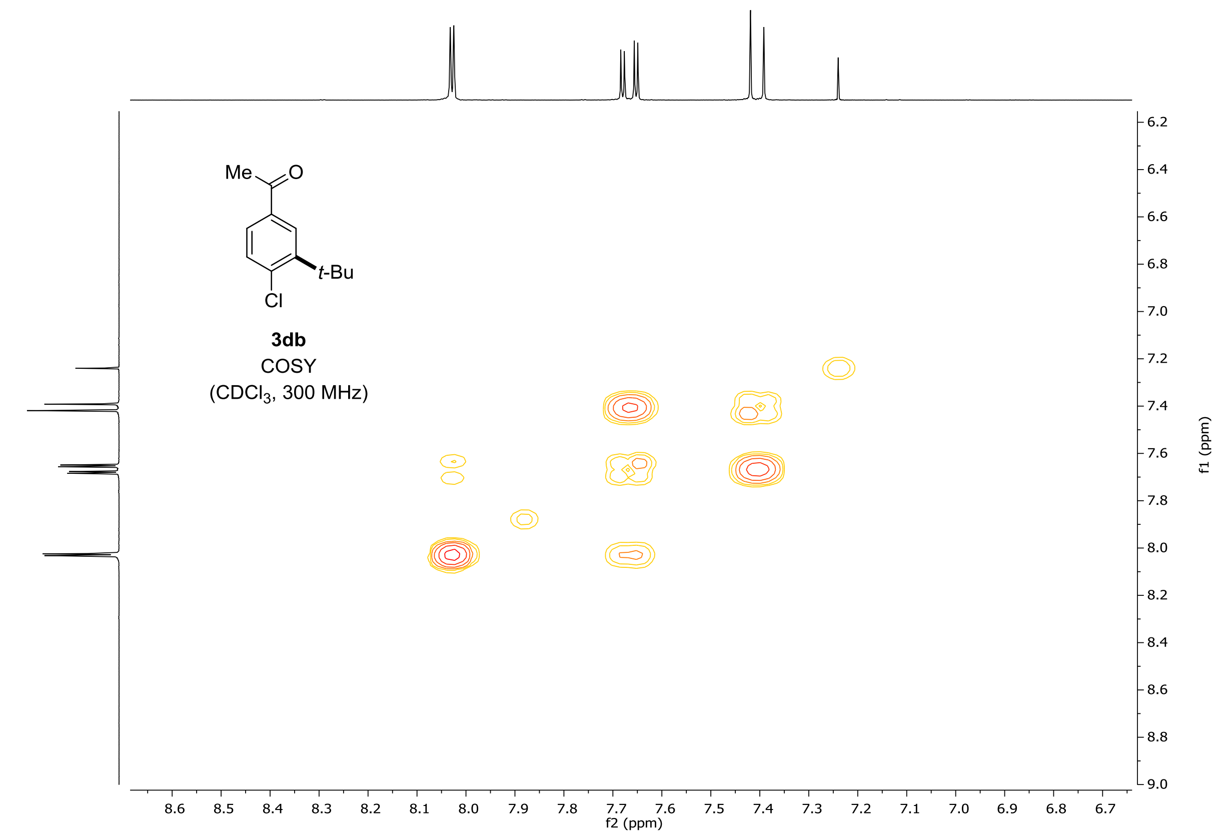
Me
O
t-Bu
Cl
3db
COSY
(CDCl3, 300 MHz)
f2 (ppm)
f1 (ppm)

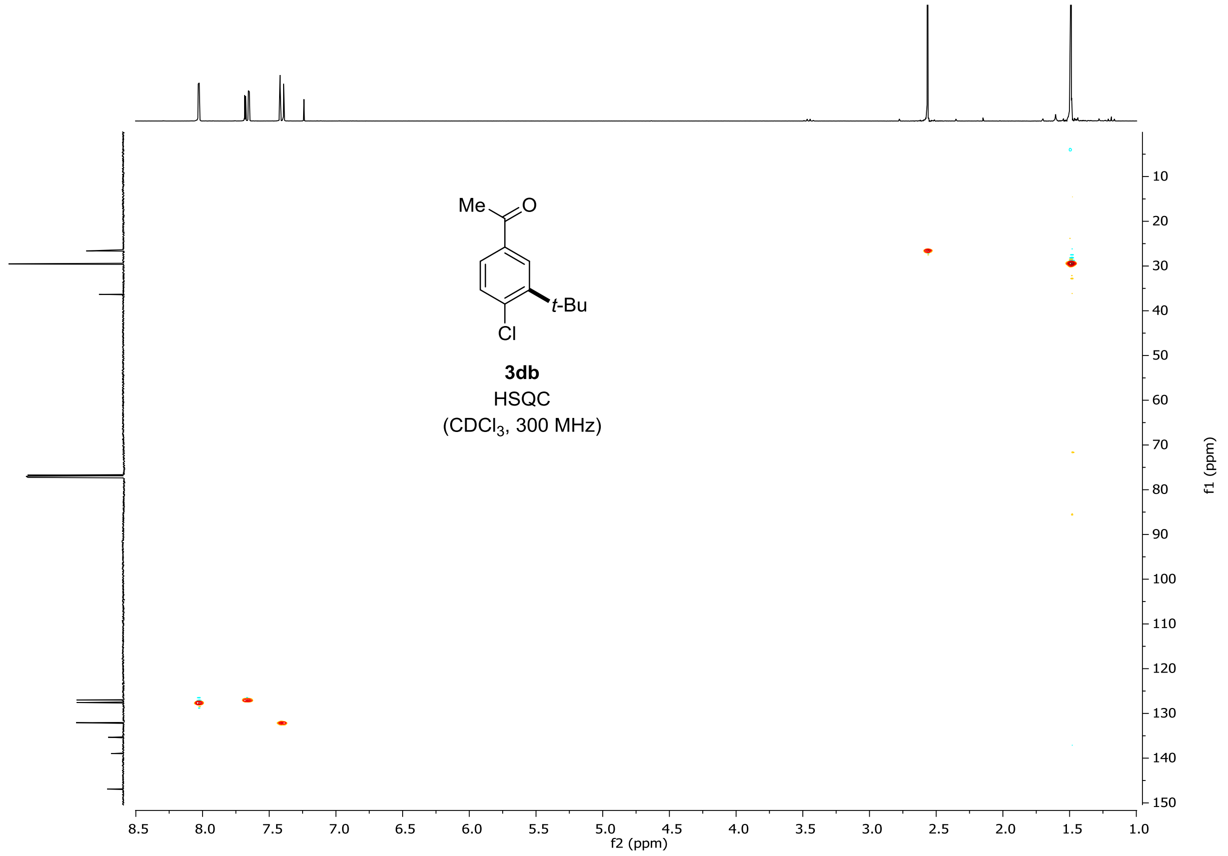
Me
O
t-Bu
Cl
3db
HSQC
(CDCl3, 300 MHz)
f2 (ppm)
f1 (ppm)

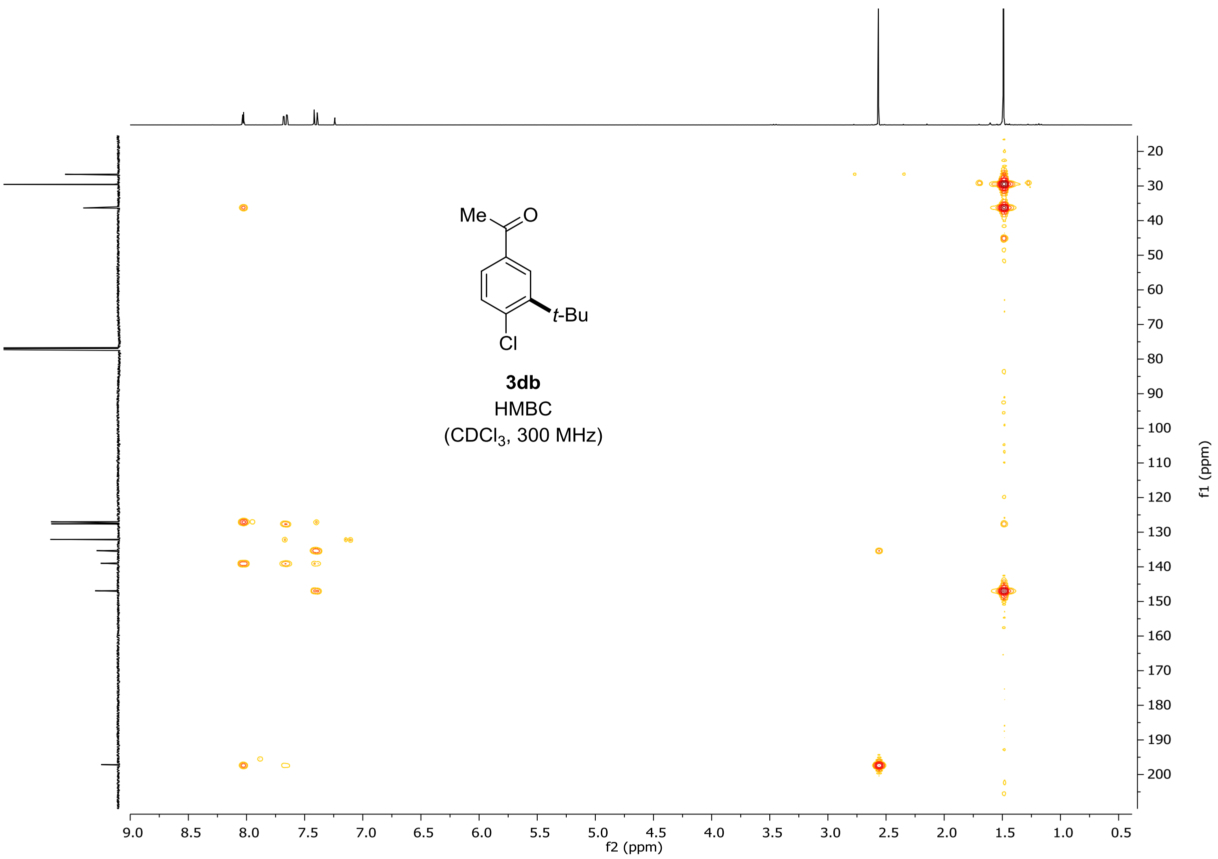

Supplementary Figure 8: ^{1}H, ^{13}C, COSY, HSQC and HMBC-NMR of Compound 3db.

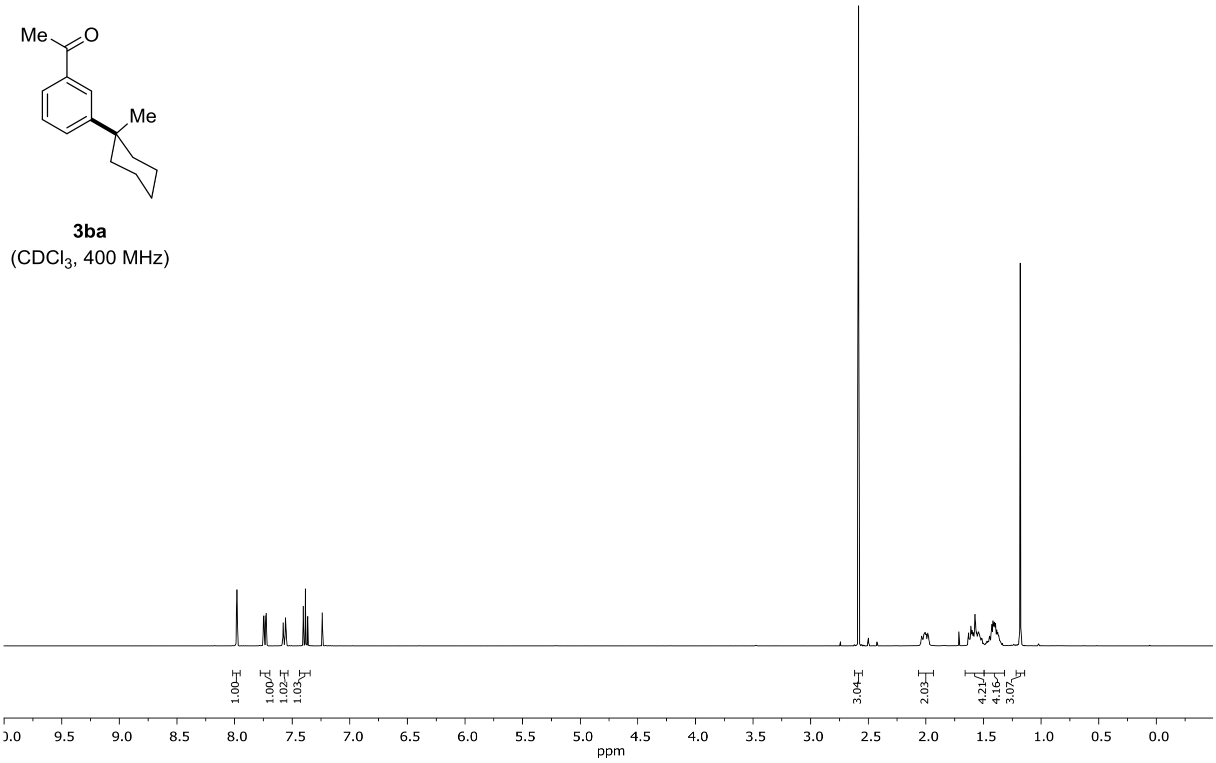

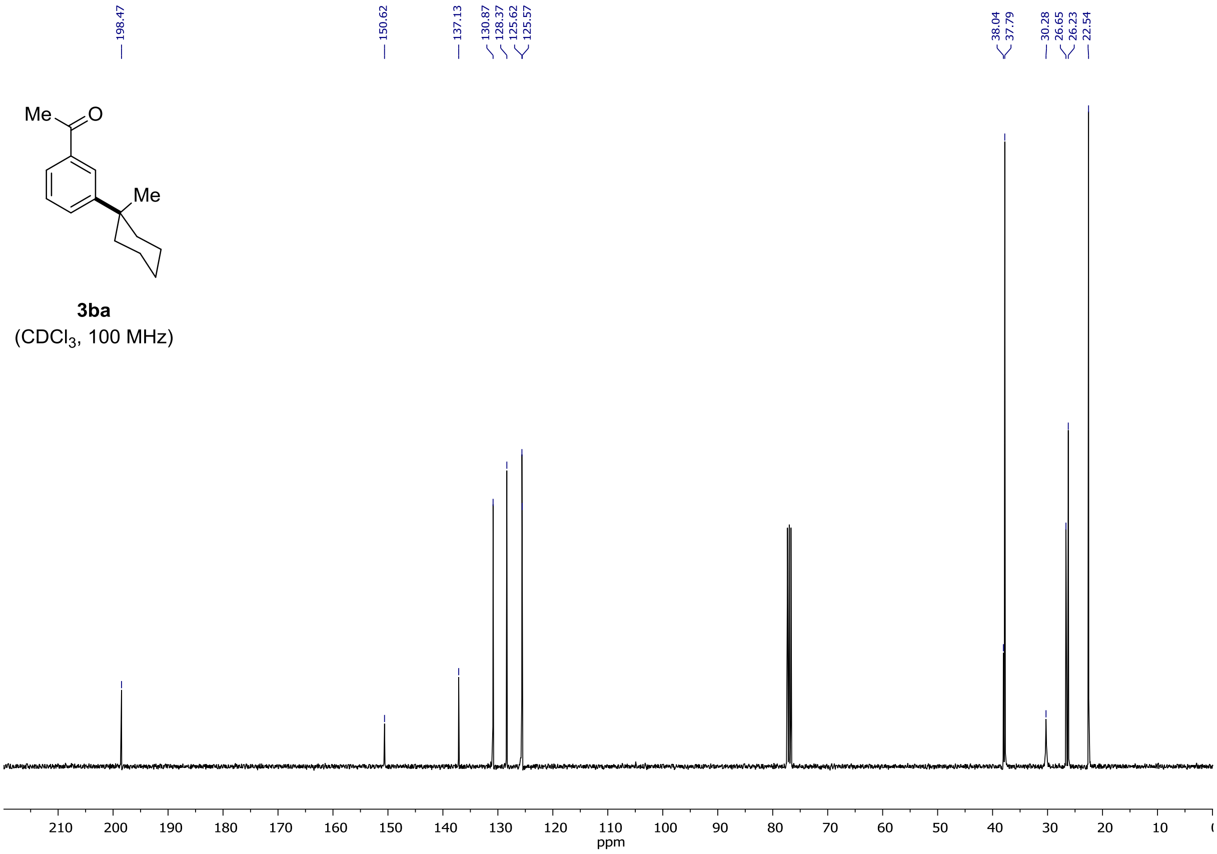

Supplementary Figure 9: ^{1}H and ^{13}C-NMR of Compound 3ba.

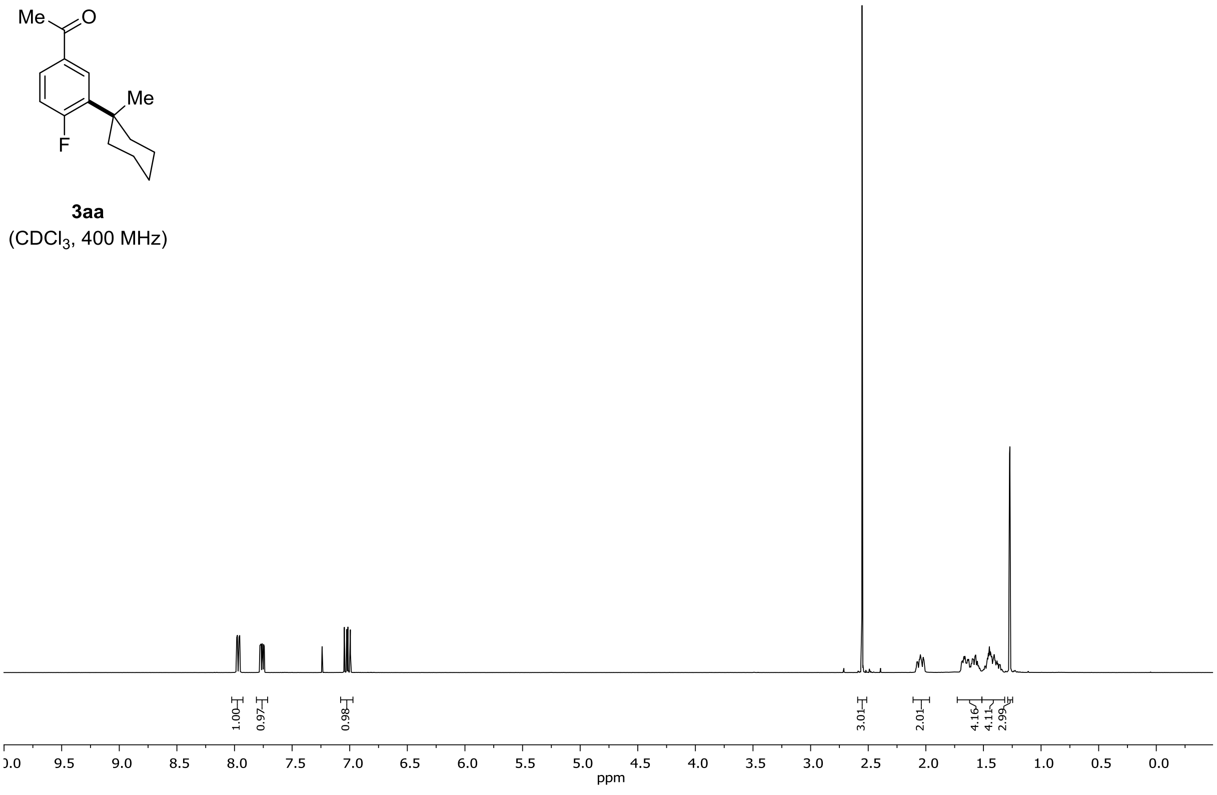

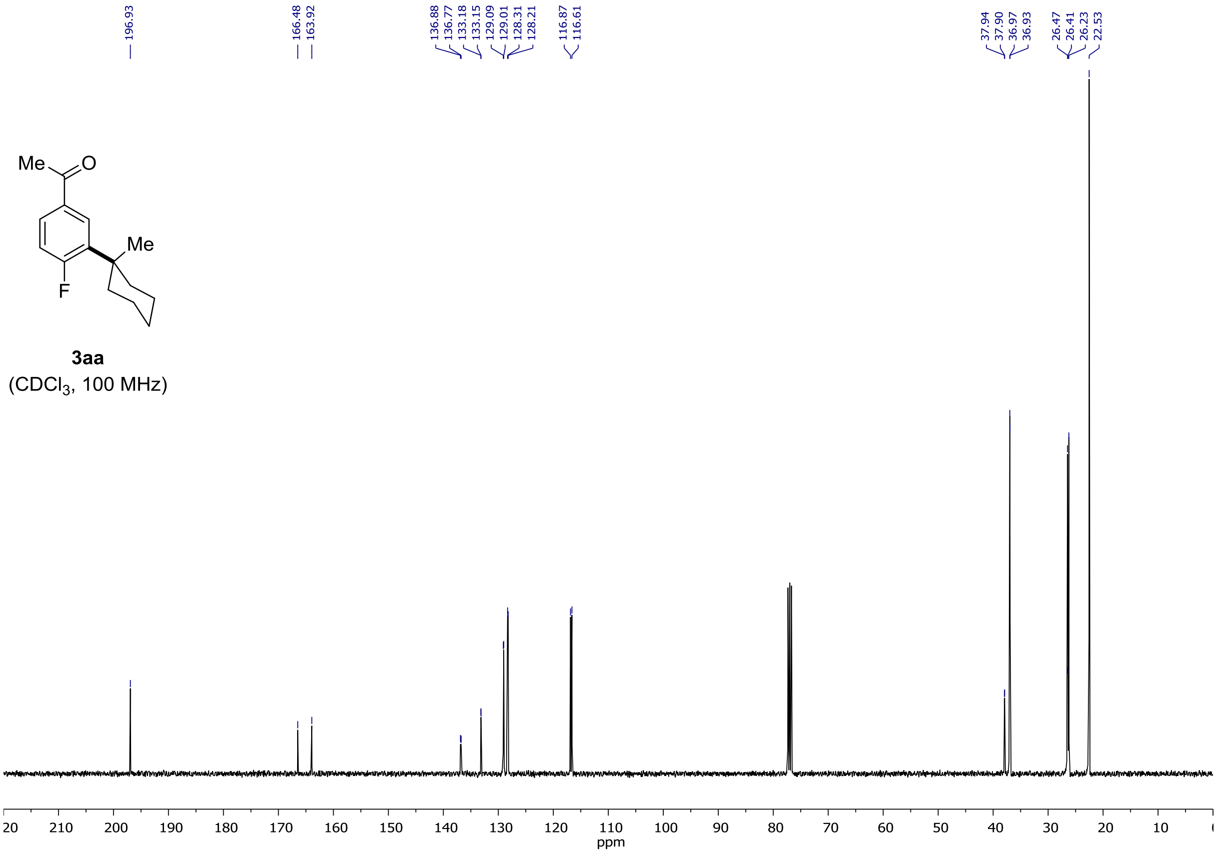

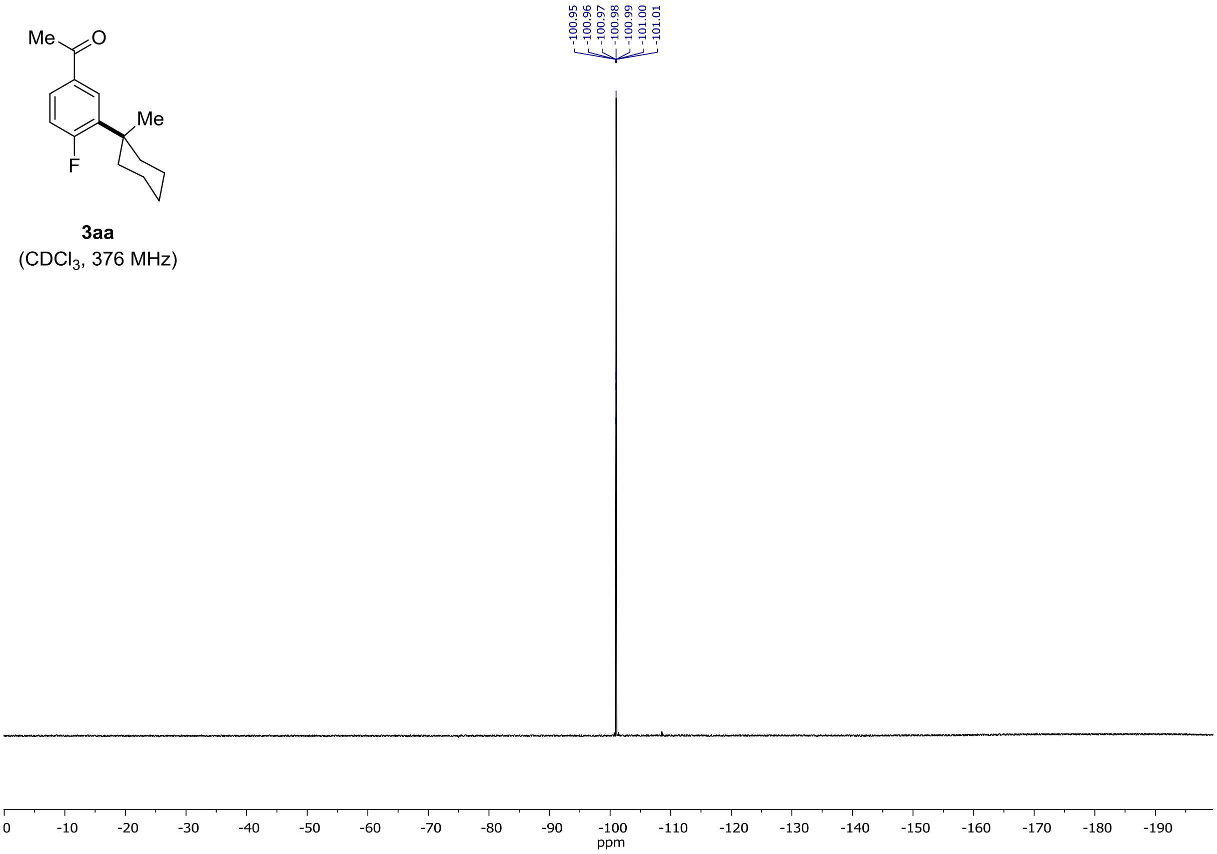

Supplementary Figure 10: ^{1}H, ^{13}C and ^{19}F-NMR of Compound 3aa.

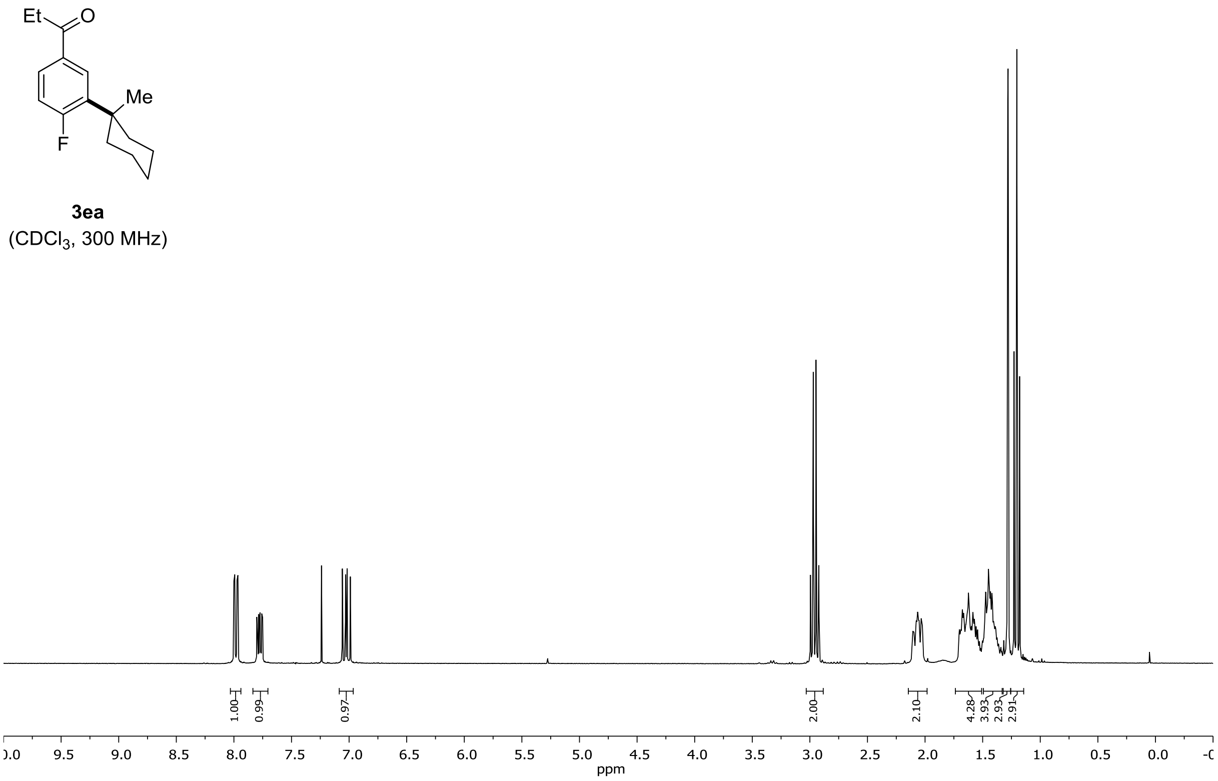
Et
O
Me
F
3ea
(CDCl3, 300 MHz)
1.00
0.99
0.97
2.00
2.10
4.28
3.93
2.93
2.91
9.5 9.0 8.5 8.0 7.5 7.0 6.5 6.0 5.5 5.0 4.5 4.0 3.5 3.0 2.5 2.0 1.5 1.0 0.5 0.0
ppm

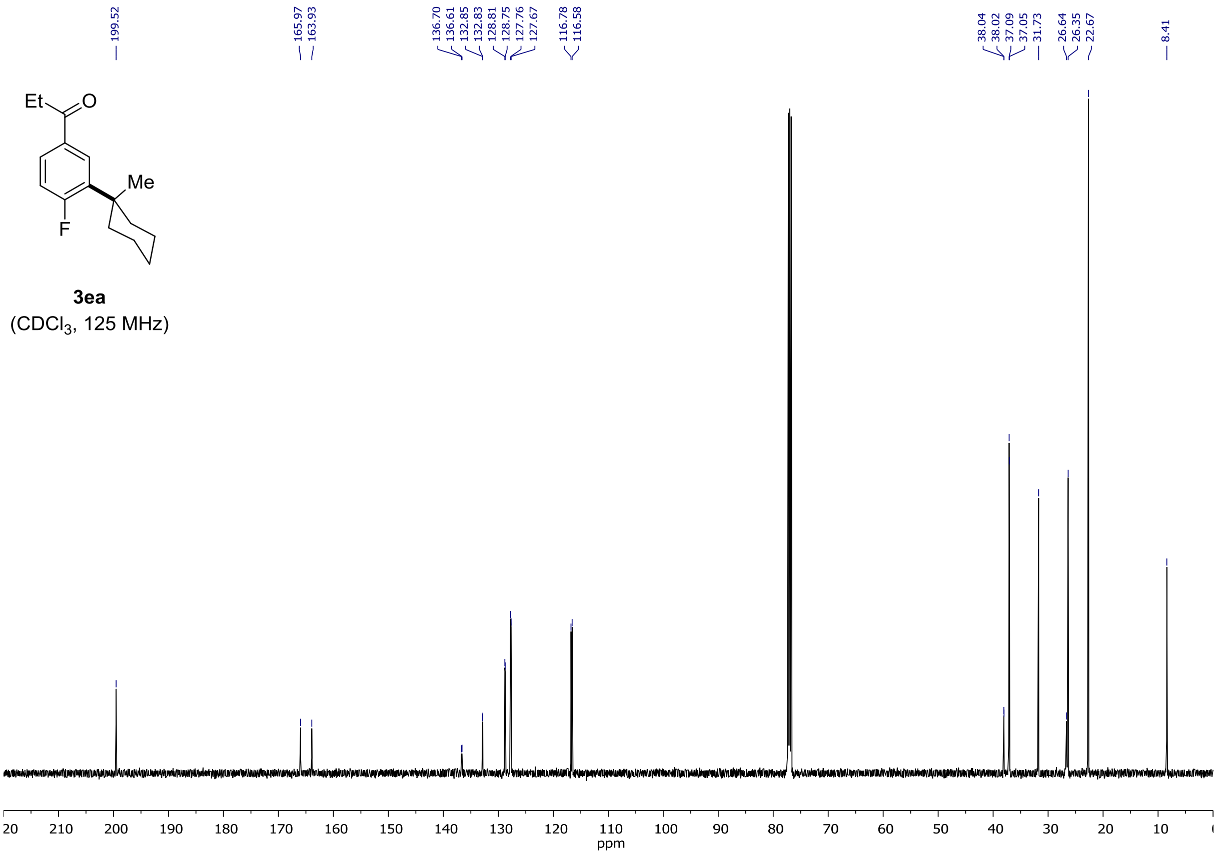
199.52
165.97
163.93
136.70
136.61
132.85
132.83
128.81
128.75
127.76
127.67
116.78
116.58
38.04
38.02
37.09
37.05
31.73
26.64
26.35
22.67
8.41
Et
O
Me
F
3ea
(CDCl3, 125 MHz)
210 200 190 180 170 160 150 140 130 120 110 100 90 80 70 60 50 40 30 20 10
ppm

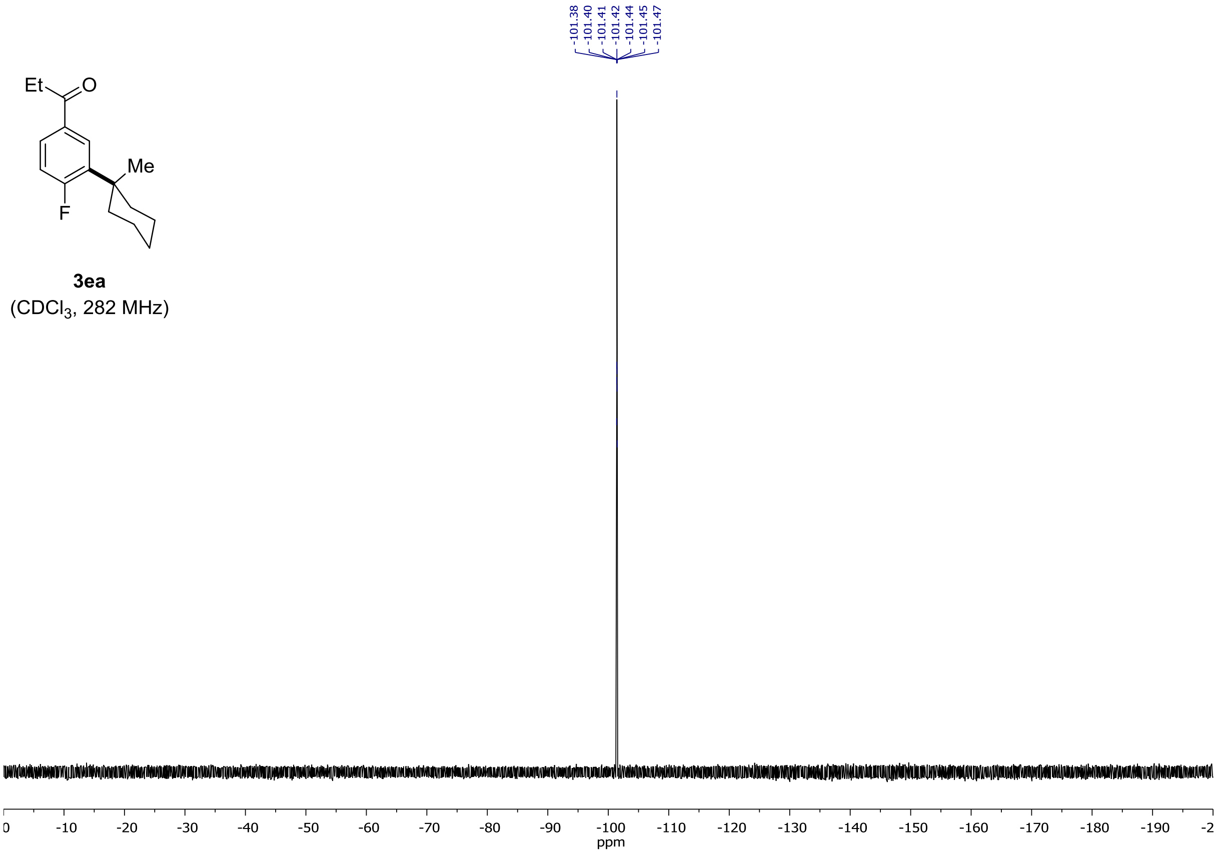

Supplementary Figure 11: ^{1}H, ^{13}C and ^{19}F-NMR of Compound 3ea.

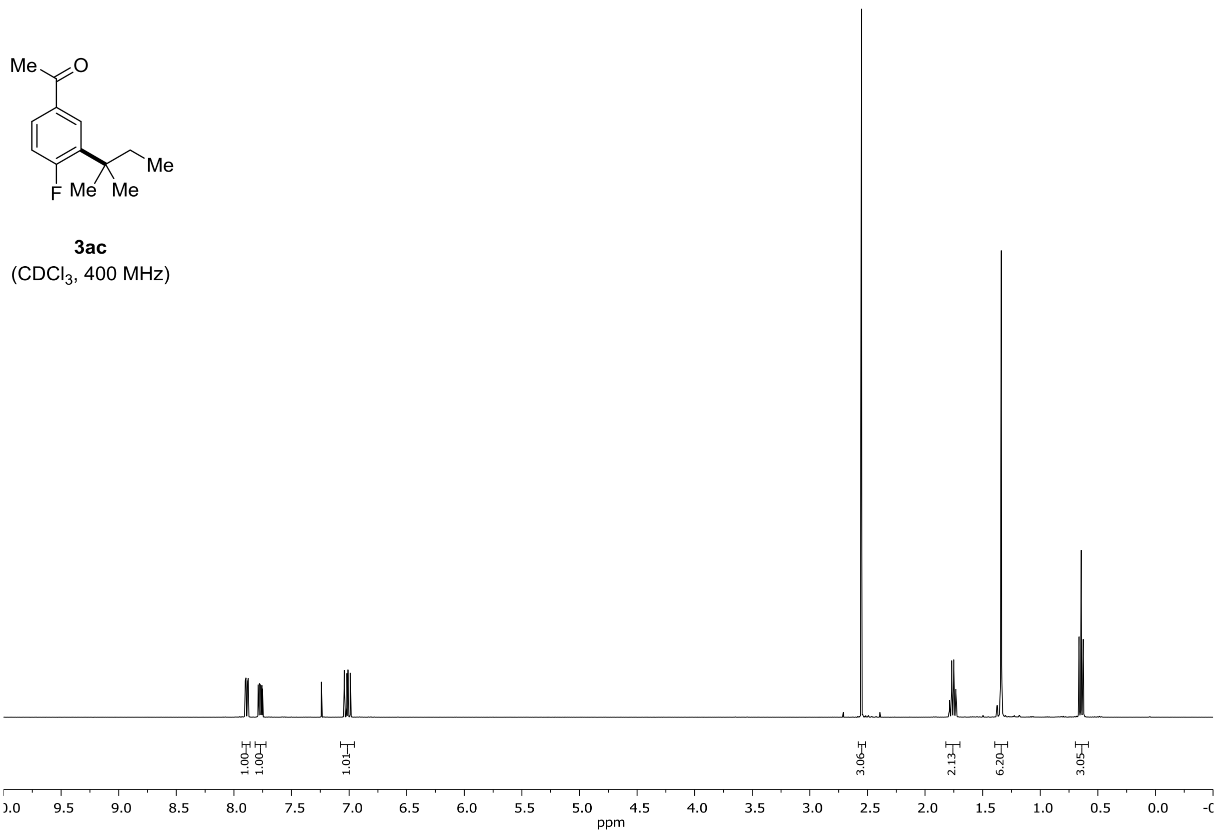

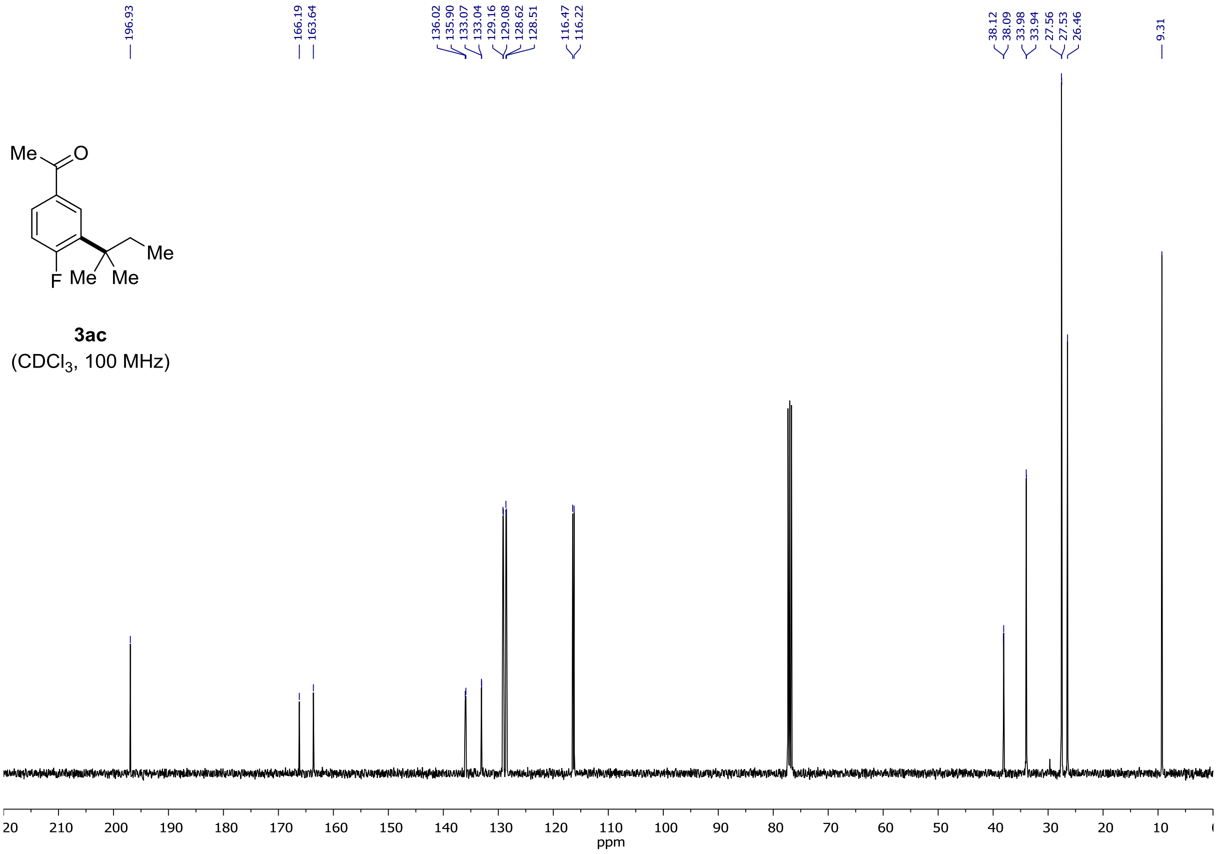

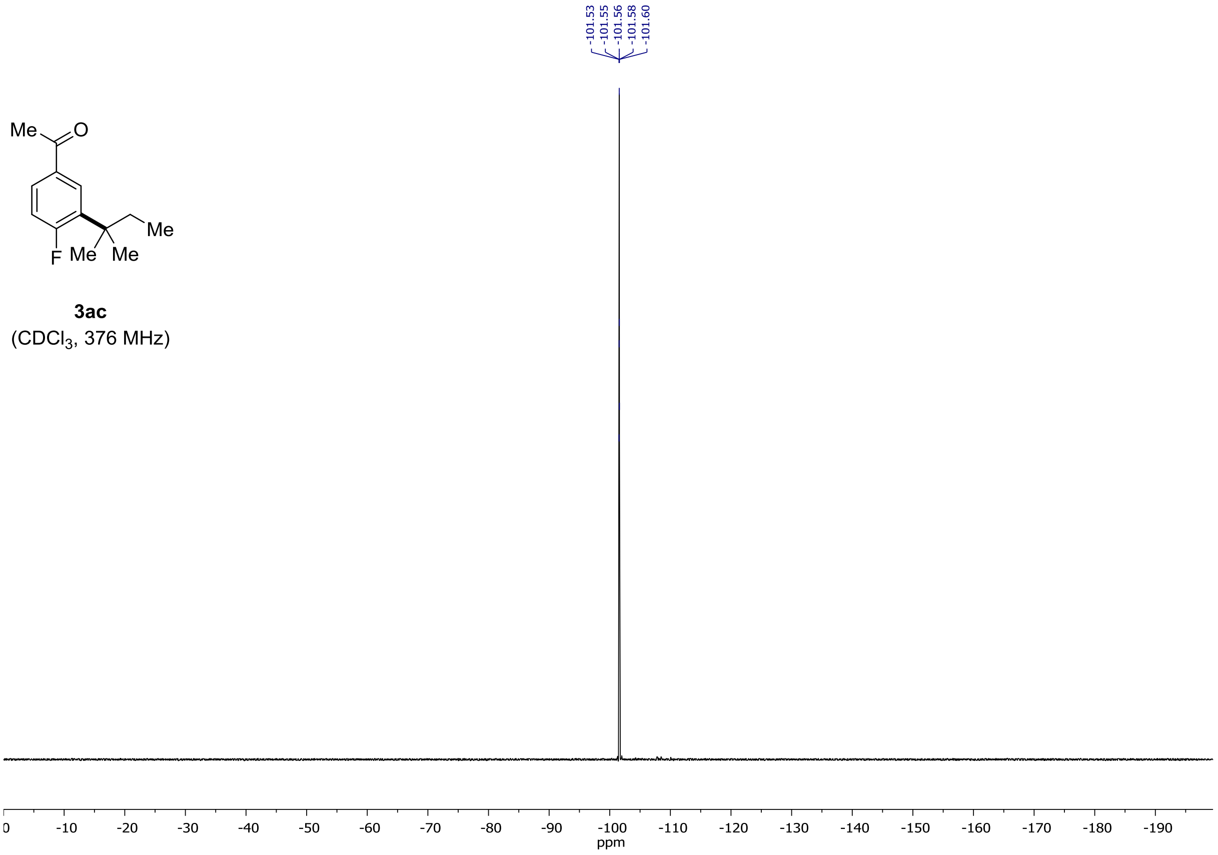

Supplementary Figure 12: ^{1}H, ^{13}C and ^{19}F-NMR of Compound 3ac.

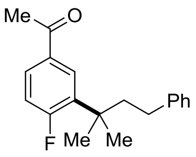

3ad
($CDCl_3$, 500 MHz)

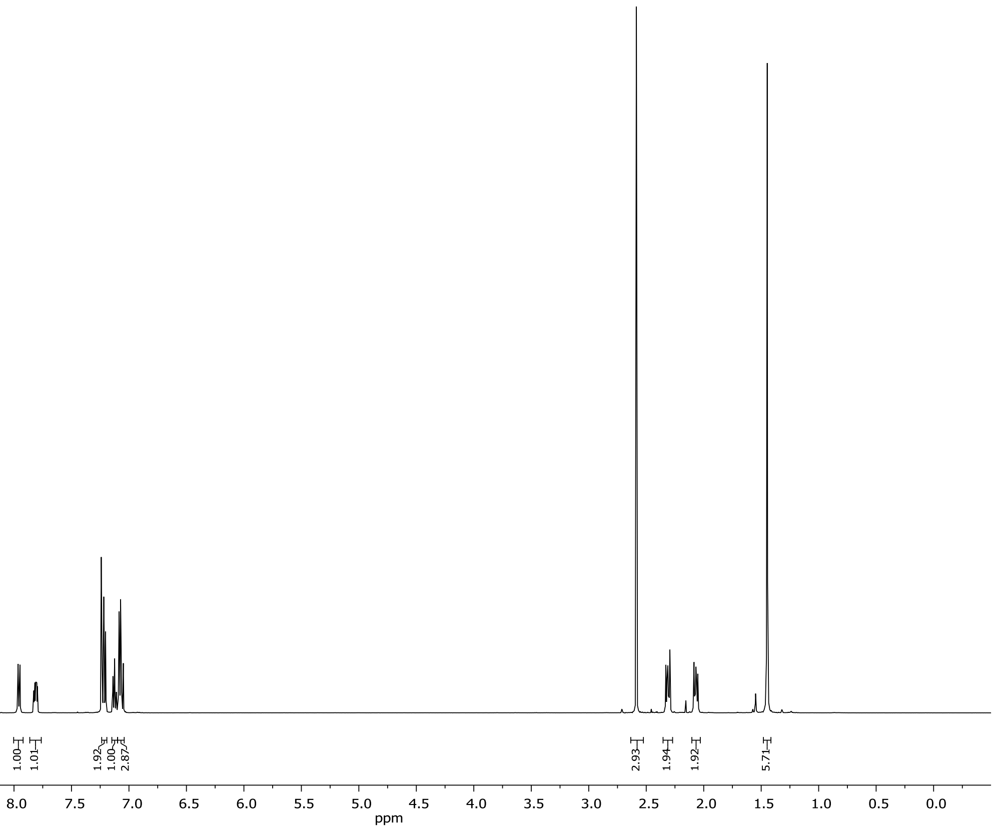

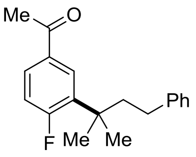

3ad
($CDCl_3$, 125 MHz)

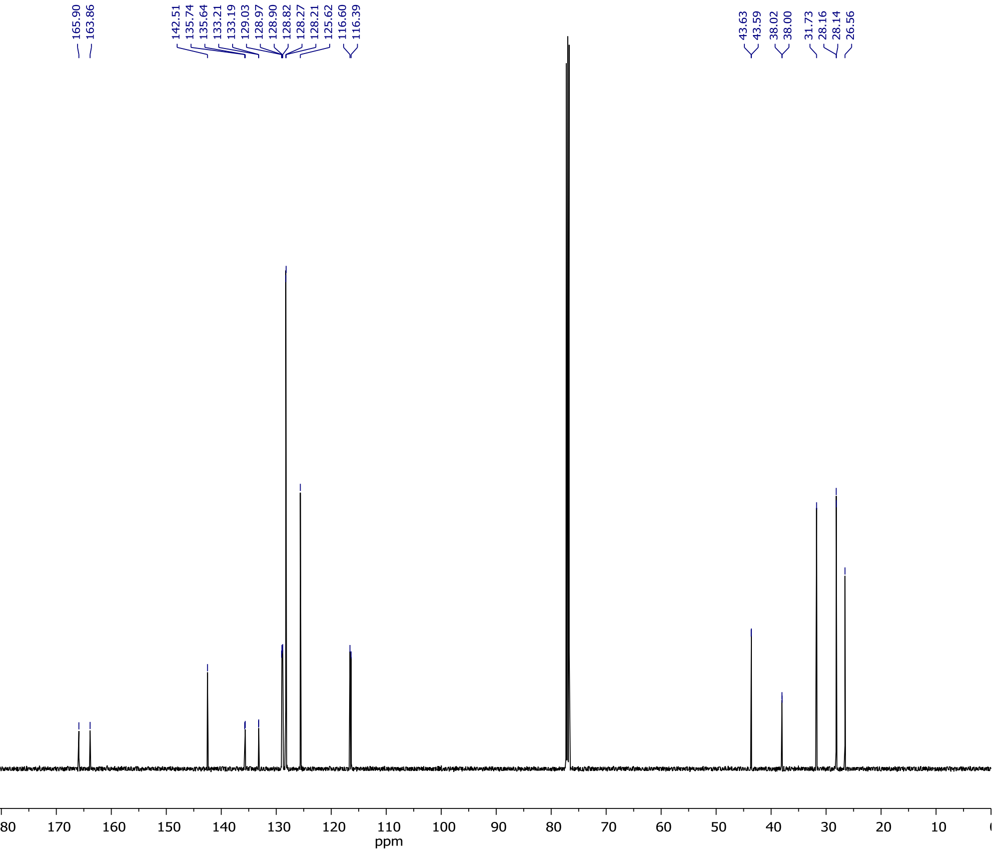

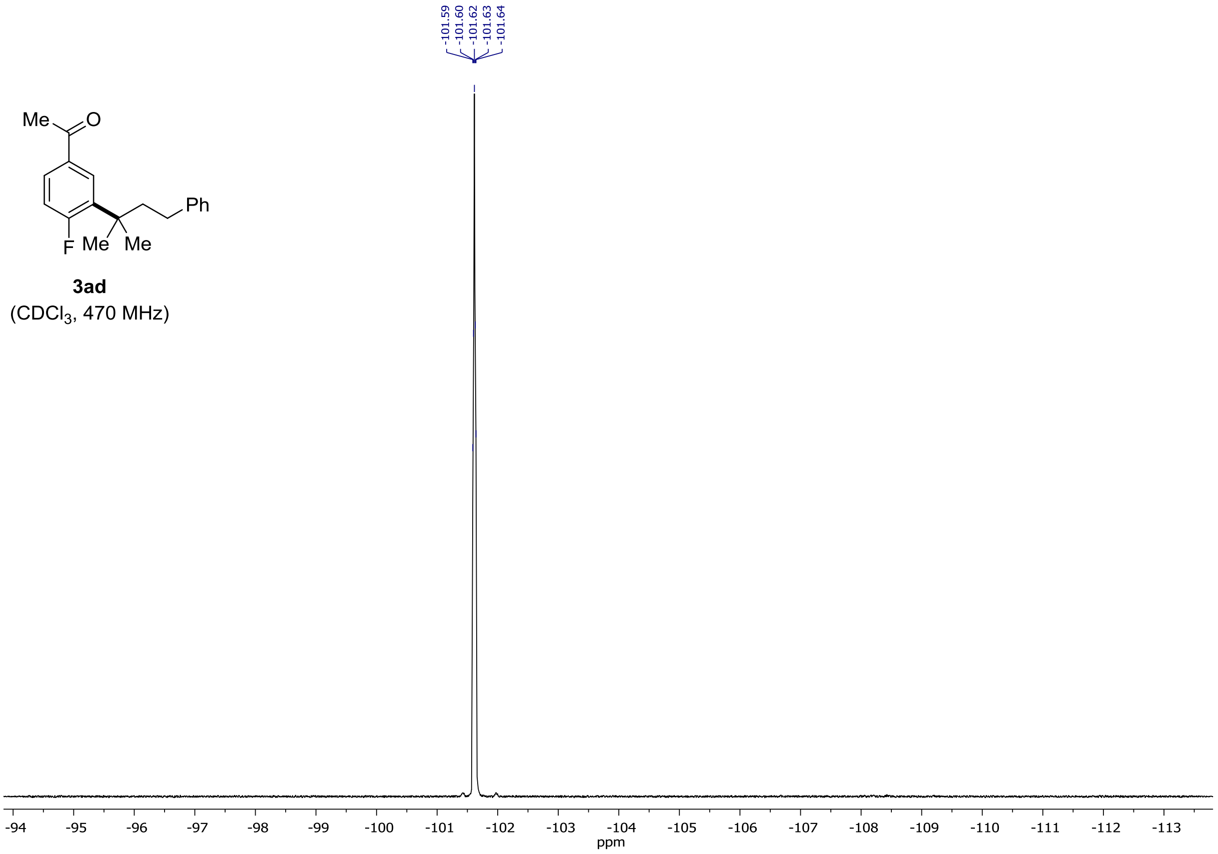

3ad
($CDCl_3$, 470 MHz)

Supplementary Figure 13: ^{1}H, ^{13}C and ^{19}F-NMR of Compound 3ad.

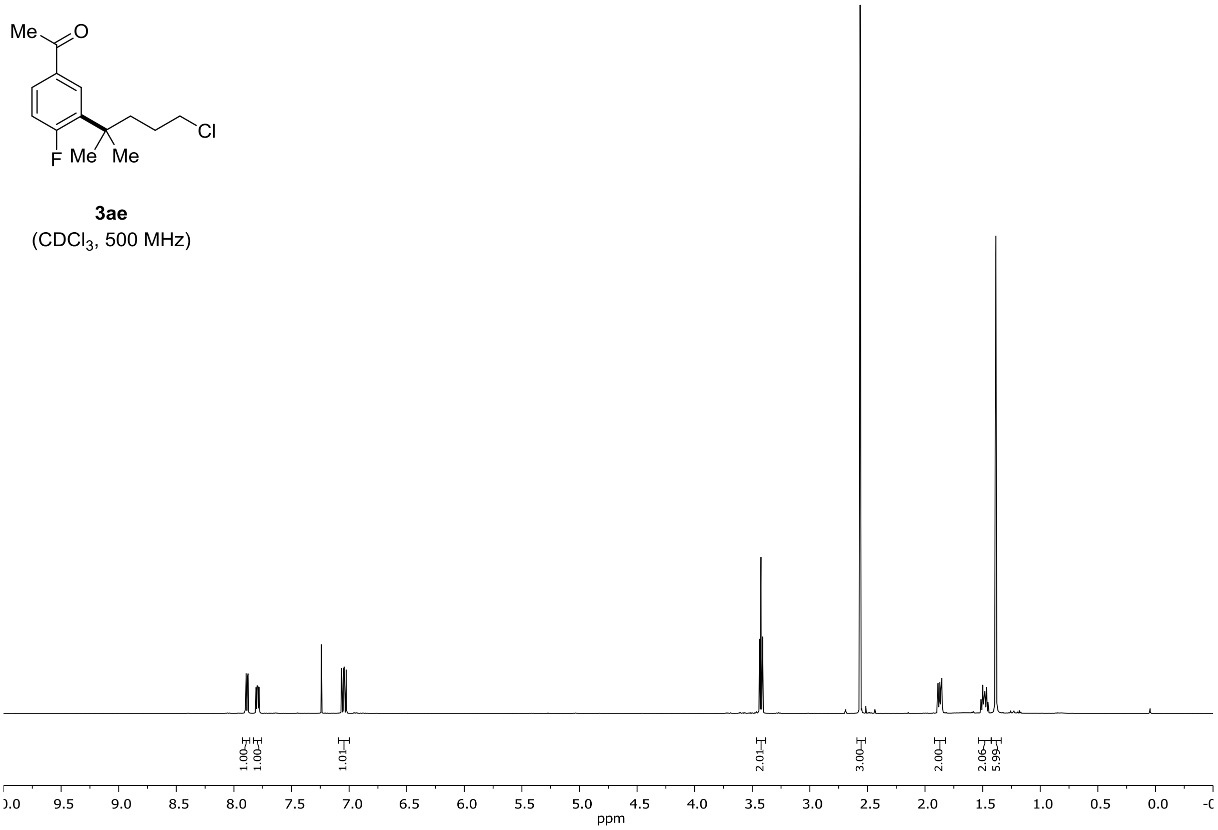

3ae
($CDCl_3$, 500 MHz)

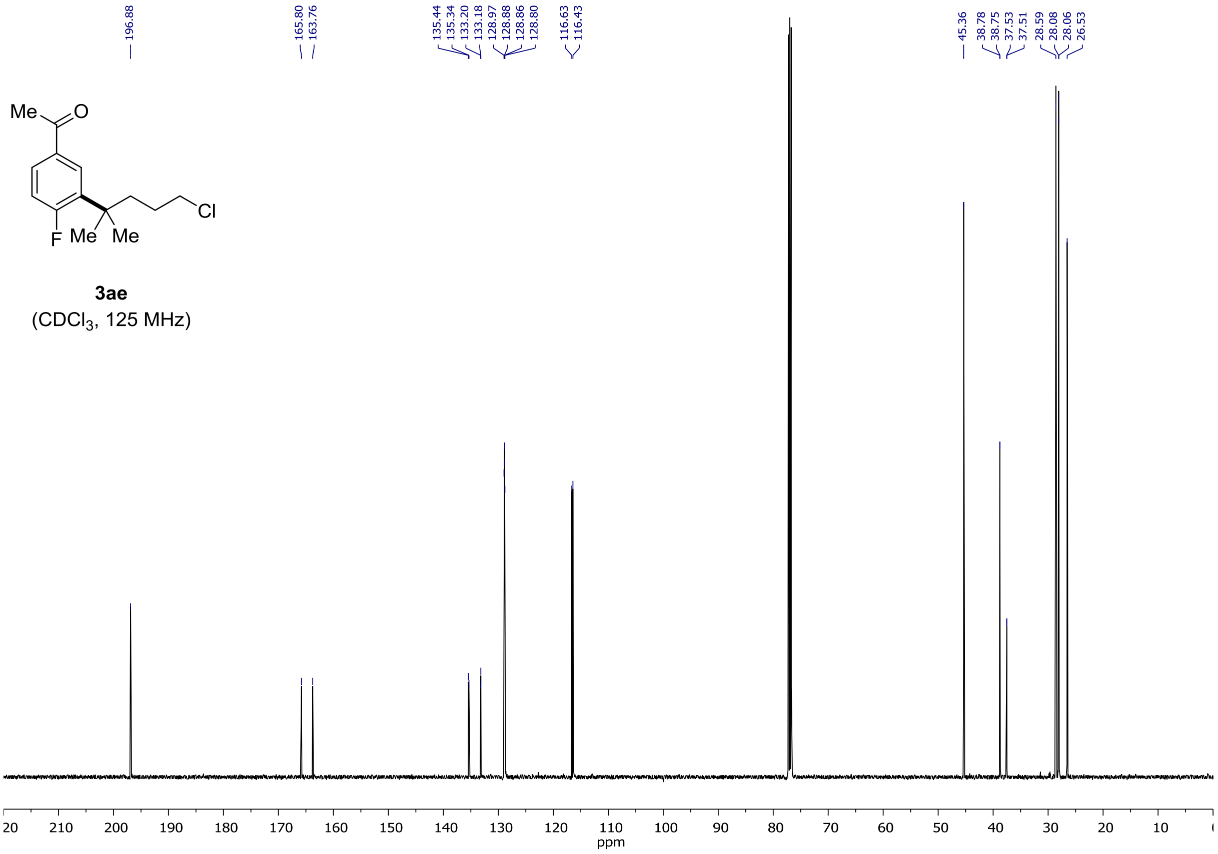

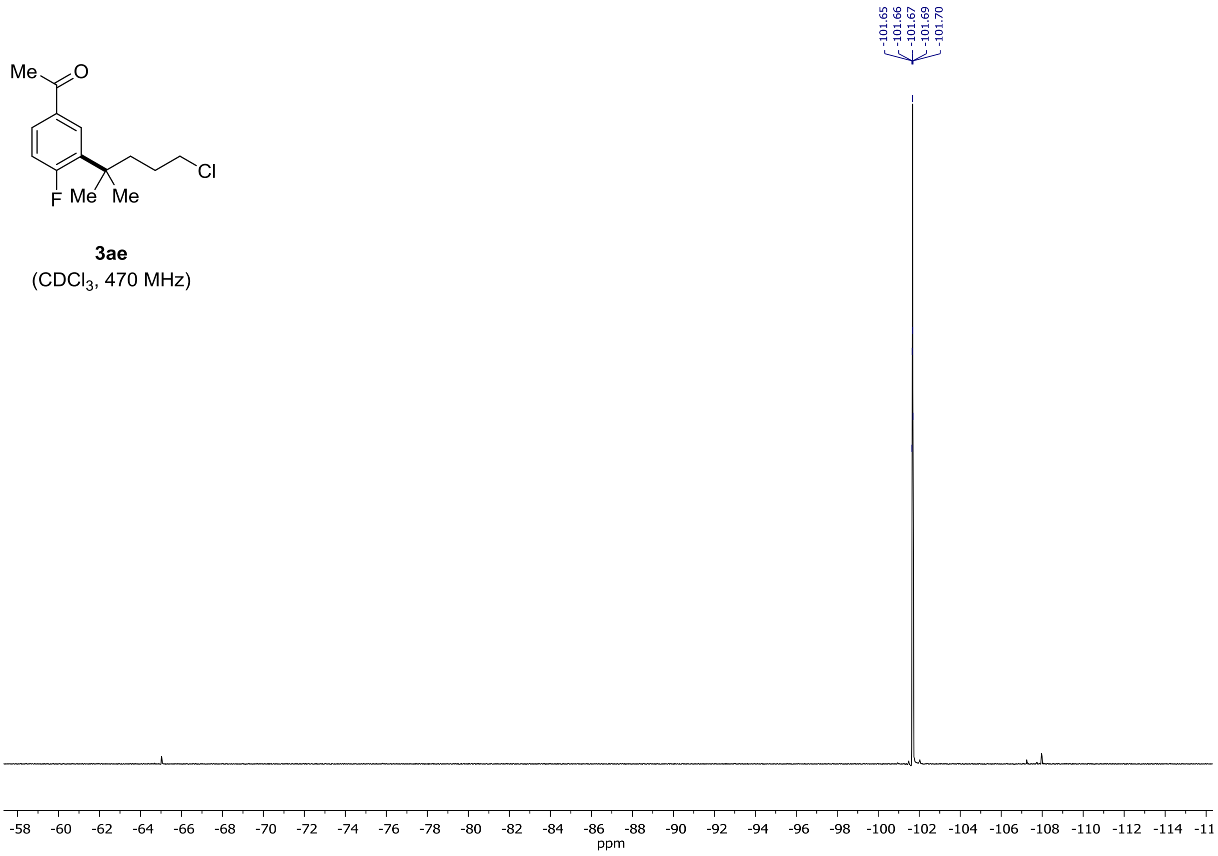

Supplementary Figure 14: ^{1}H, ^{13}C and ^{19}F-NMR of Compound 3ae.

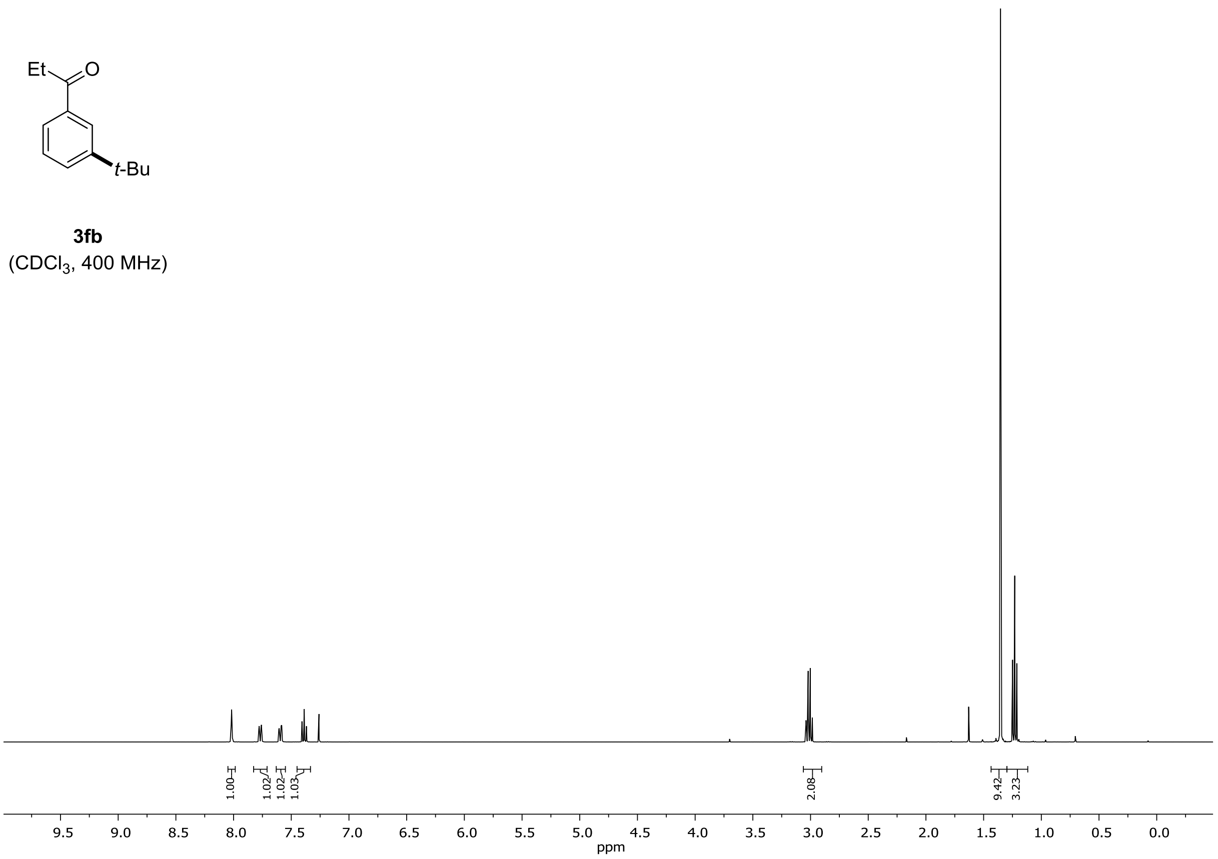

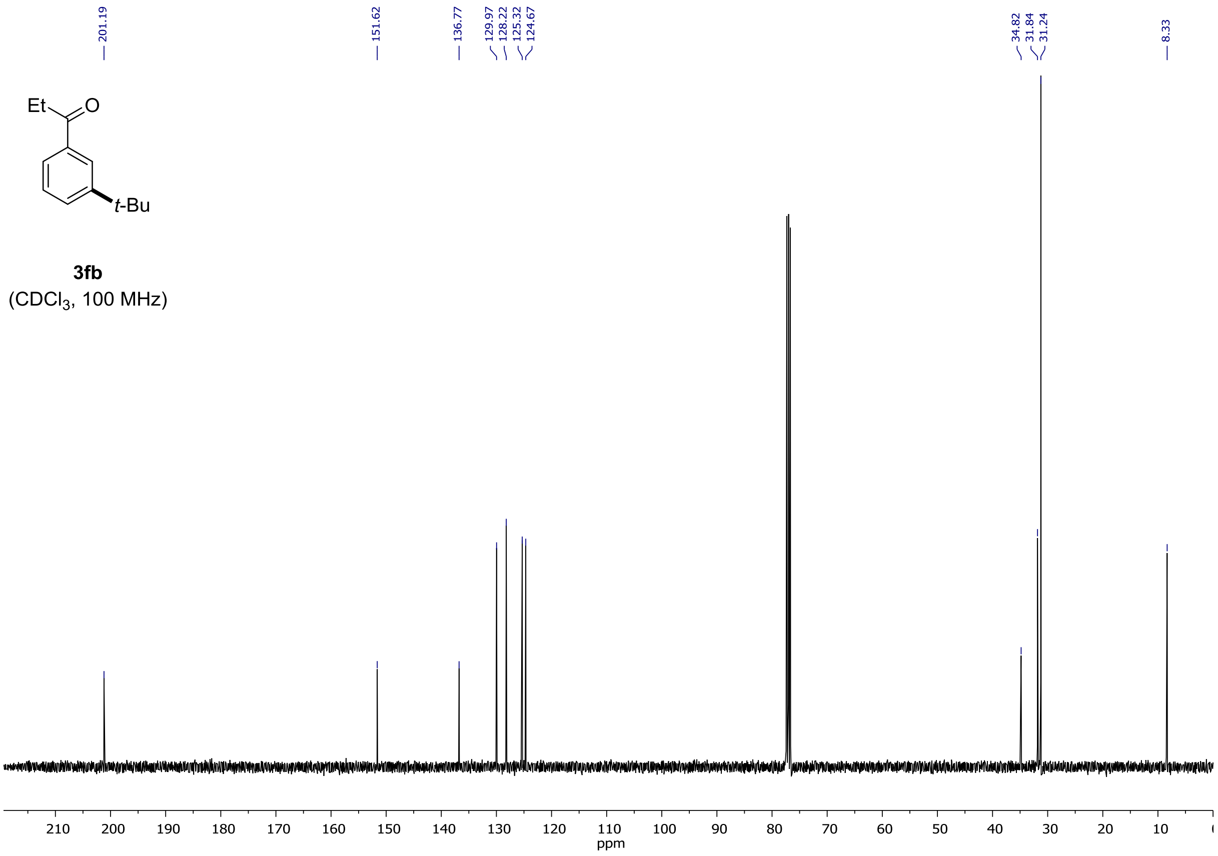

Supplementary Figure 15: ^{1}H and ^{13}C-NMR of Compound 3fb.

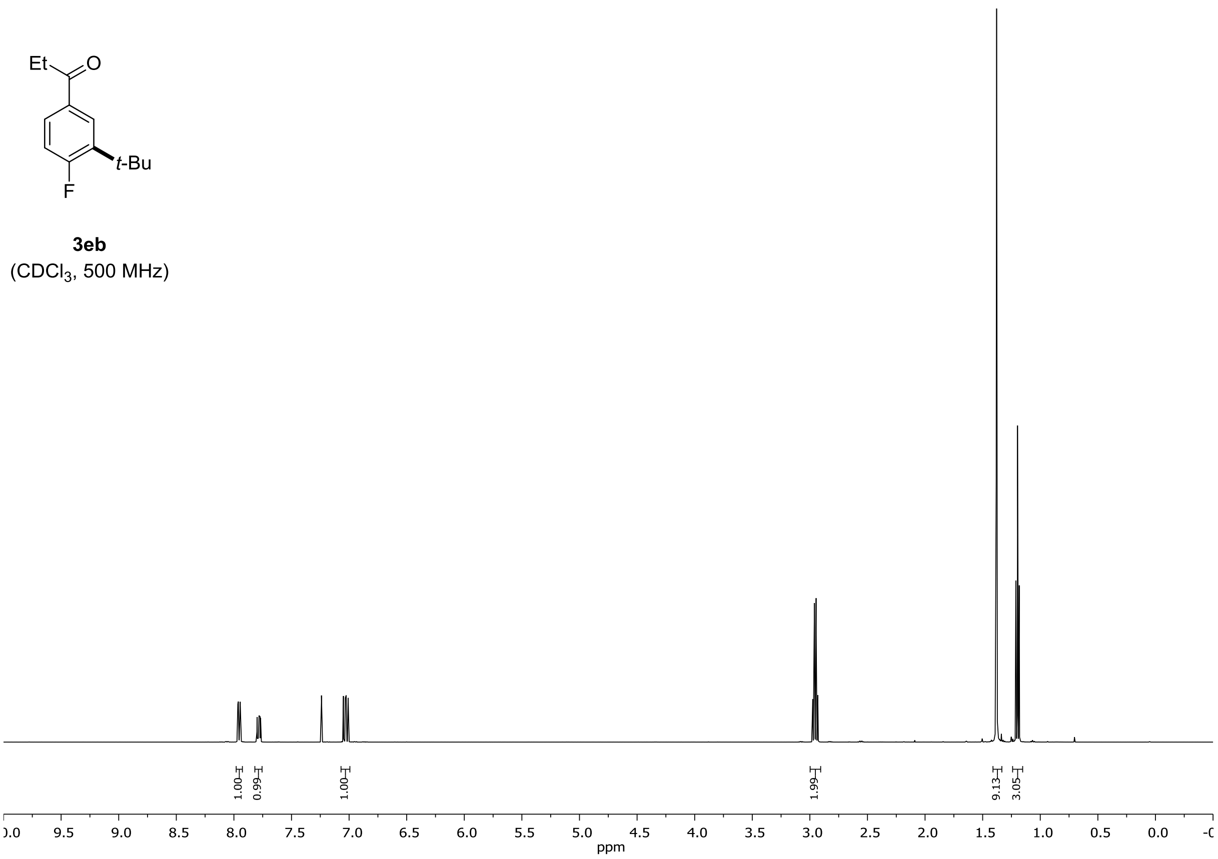

3eb
($CDCl_3$, 500 MHz)

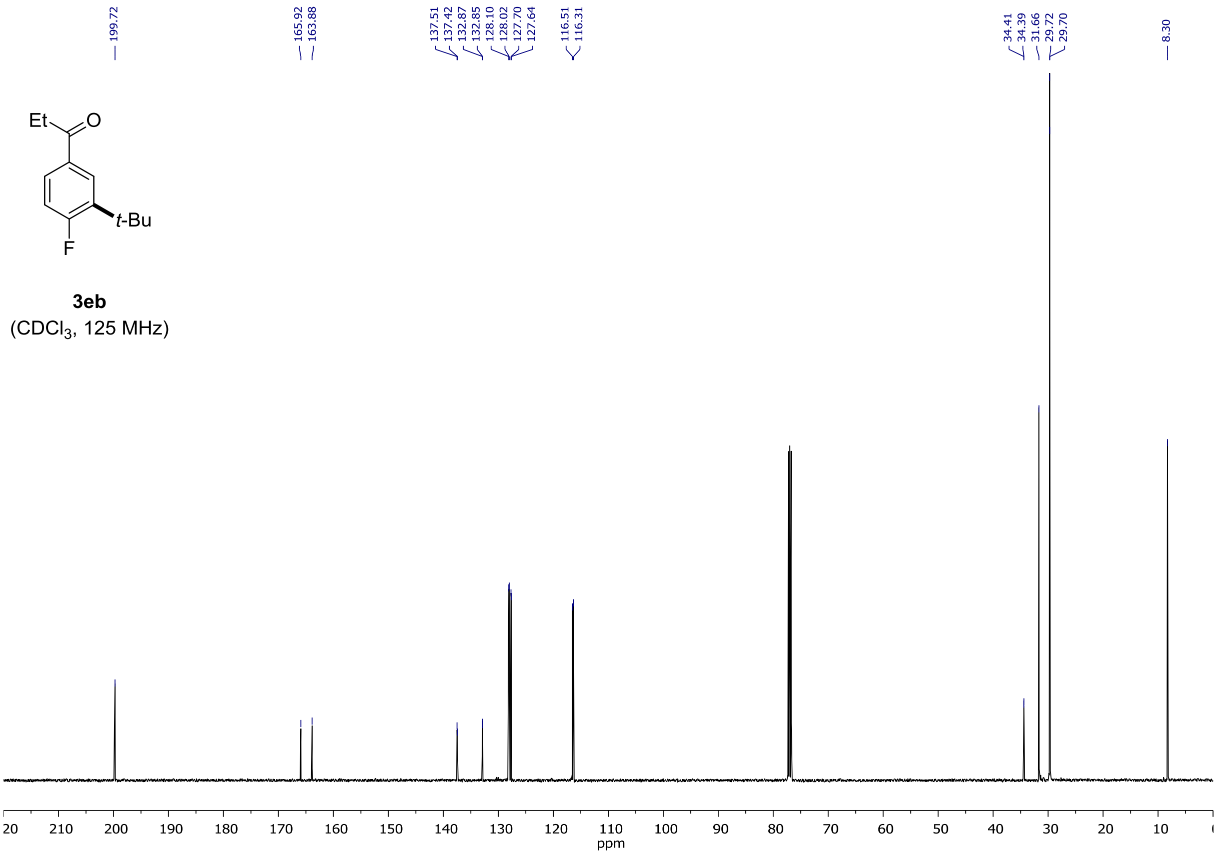

3eb
($CDCl_3$, 125 MHz)

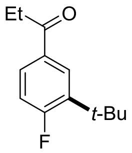

3eb
($CDCl_3$, 470 MHz)

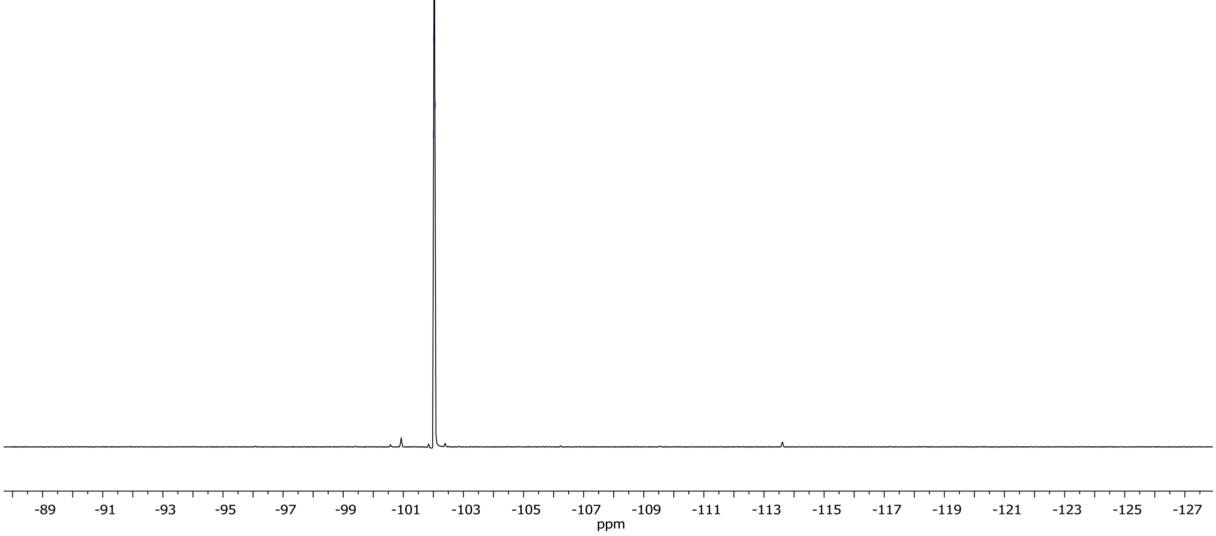

Supplementary Figure 16: ^{1}H, ^{13}C and ^{19}F-NMR of Compound 3eb.

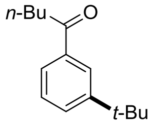

3gb
($CDCl_3$, 400 MHz)

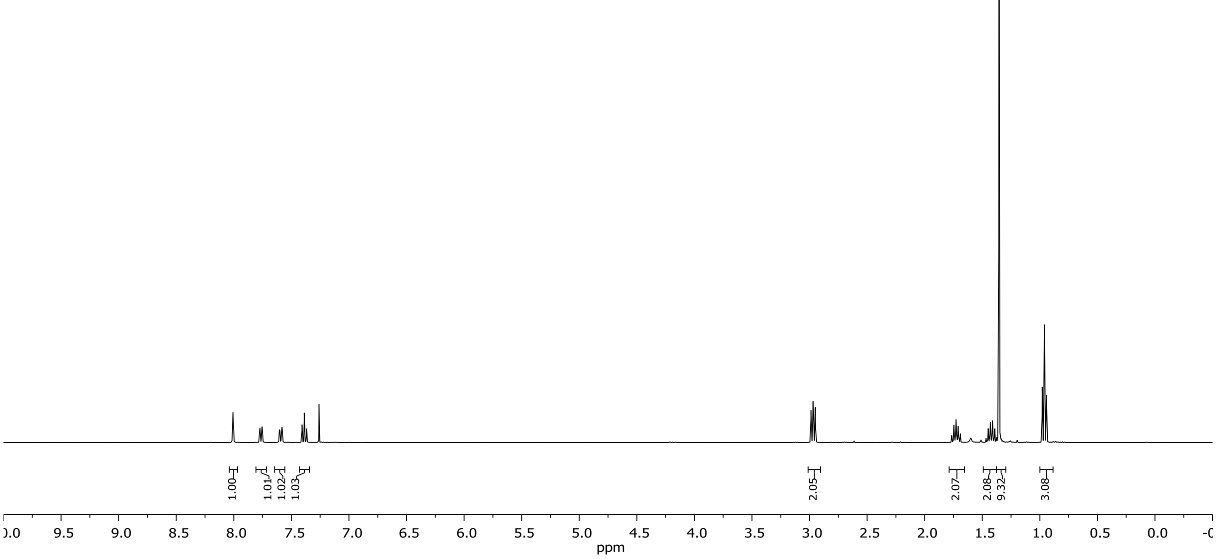

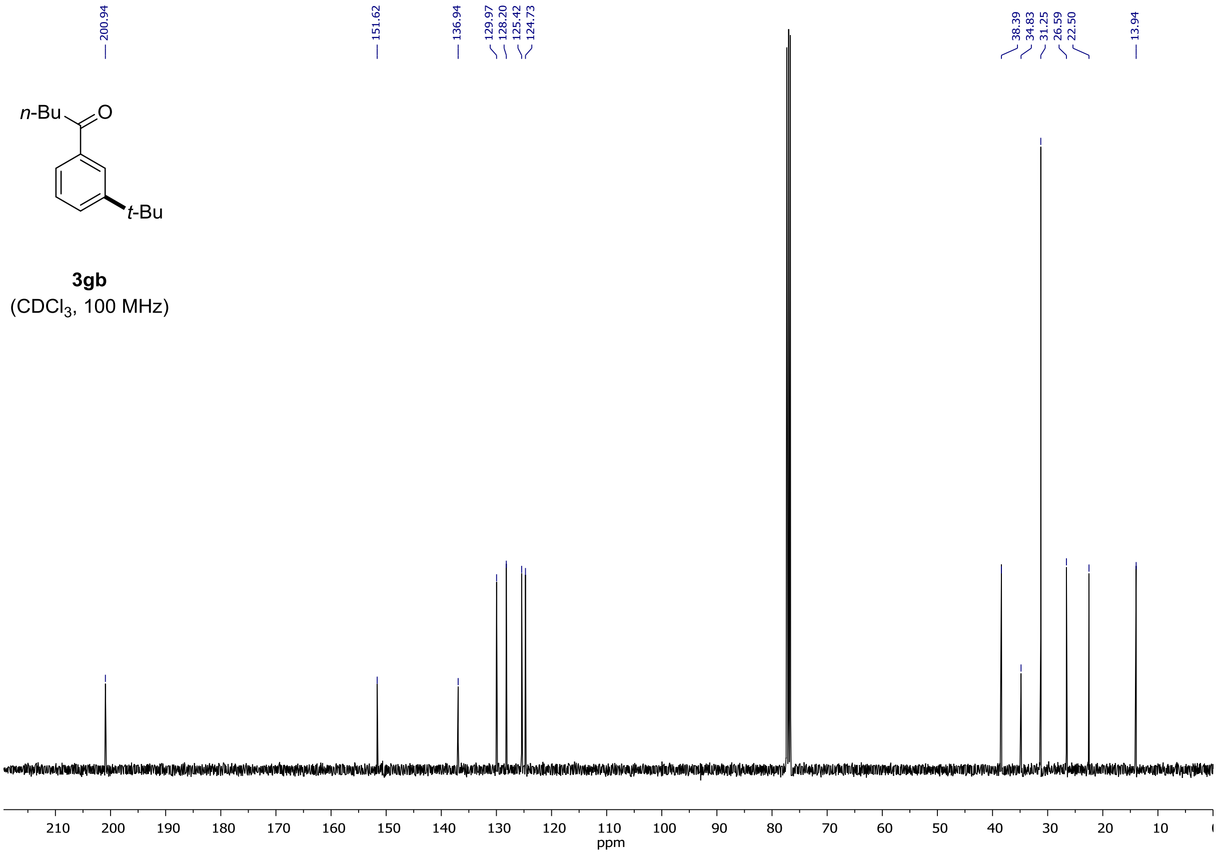

Supplementary Figure 17: ^{1}H and ^{13}C-NMR of Compound 3gb.

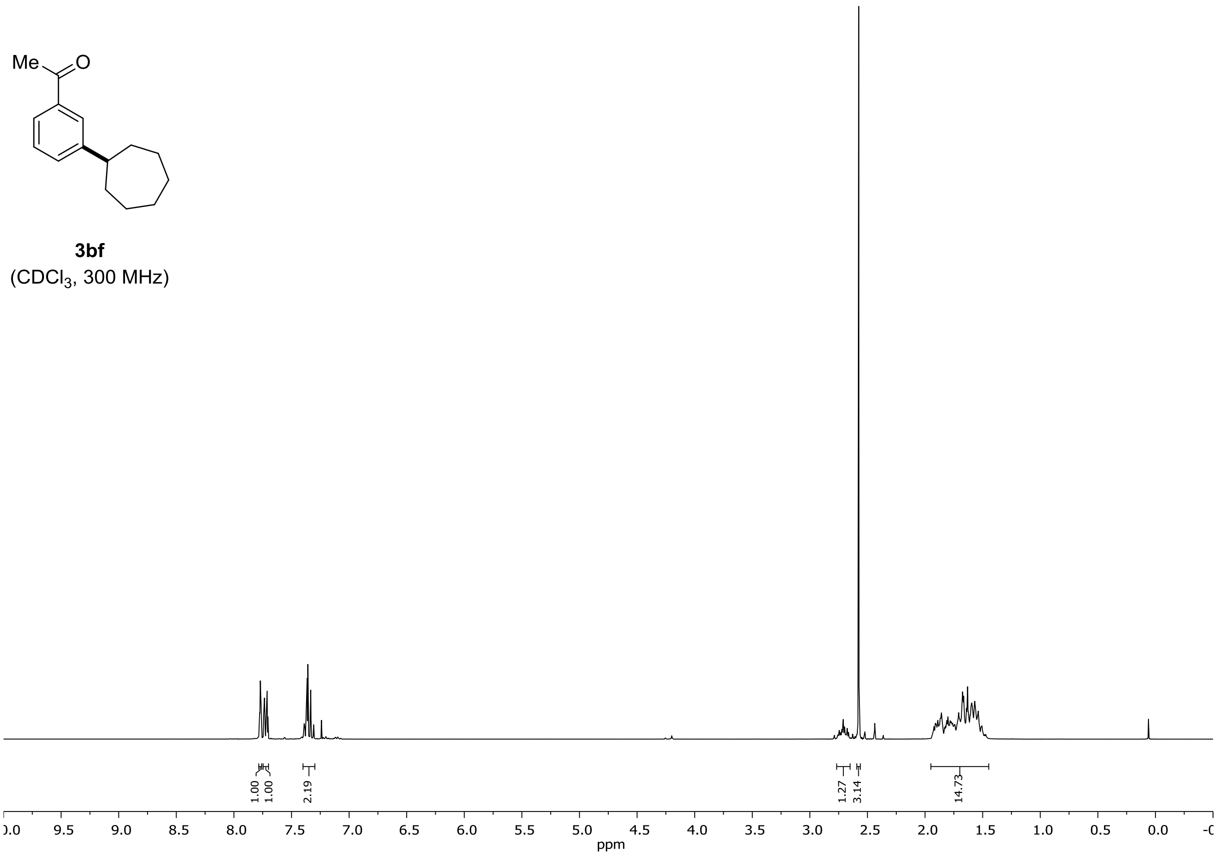

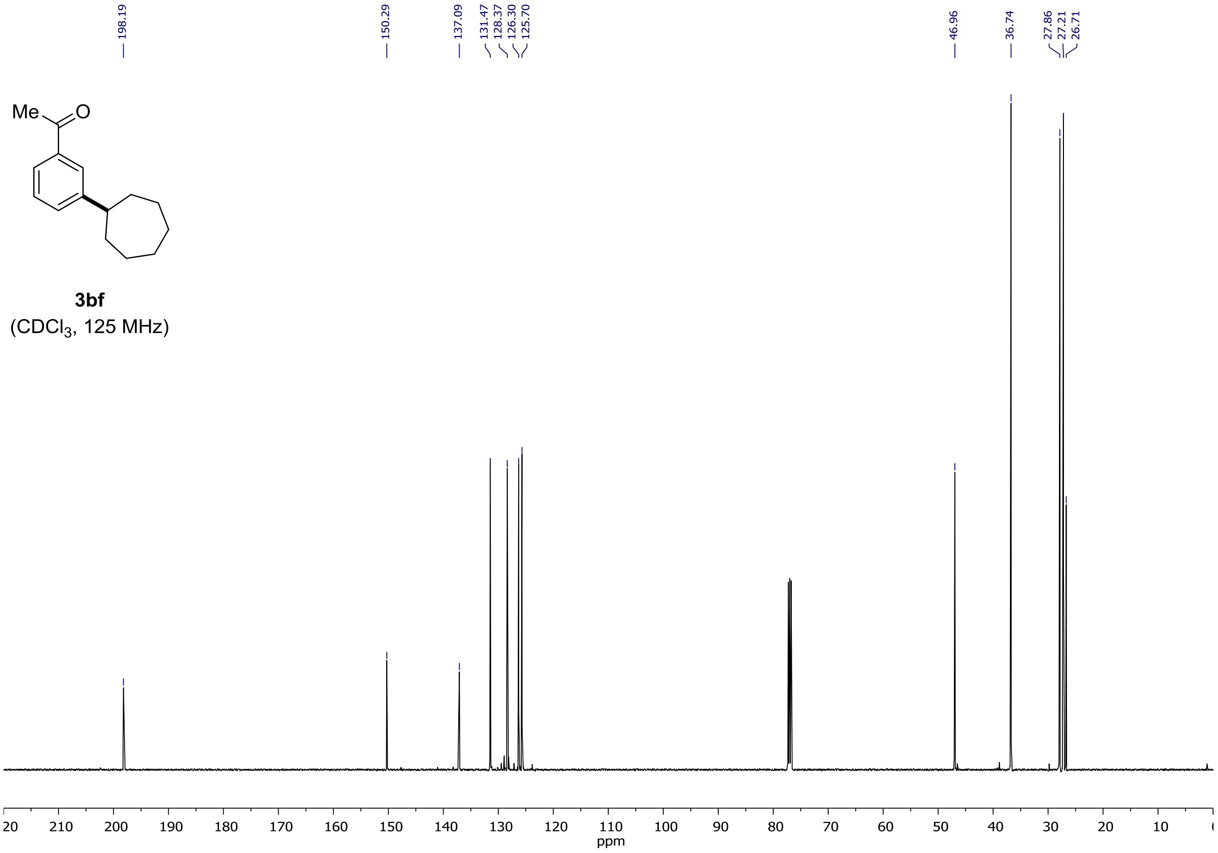

Supplementary Figure 18: ^{1}H and ^{13}C-NMR of Compound 3bf.

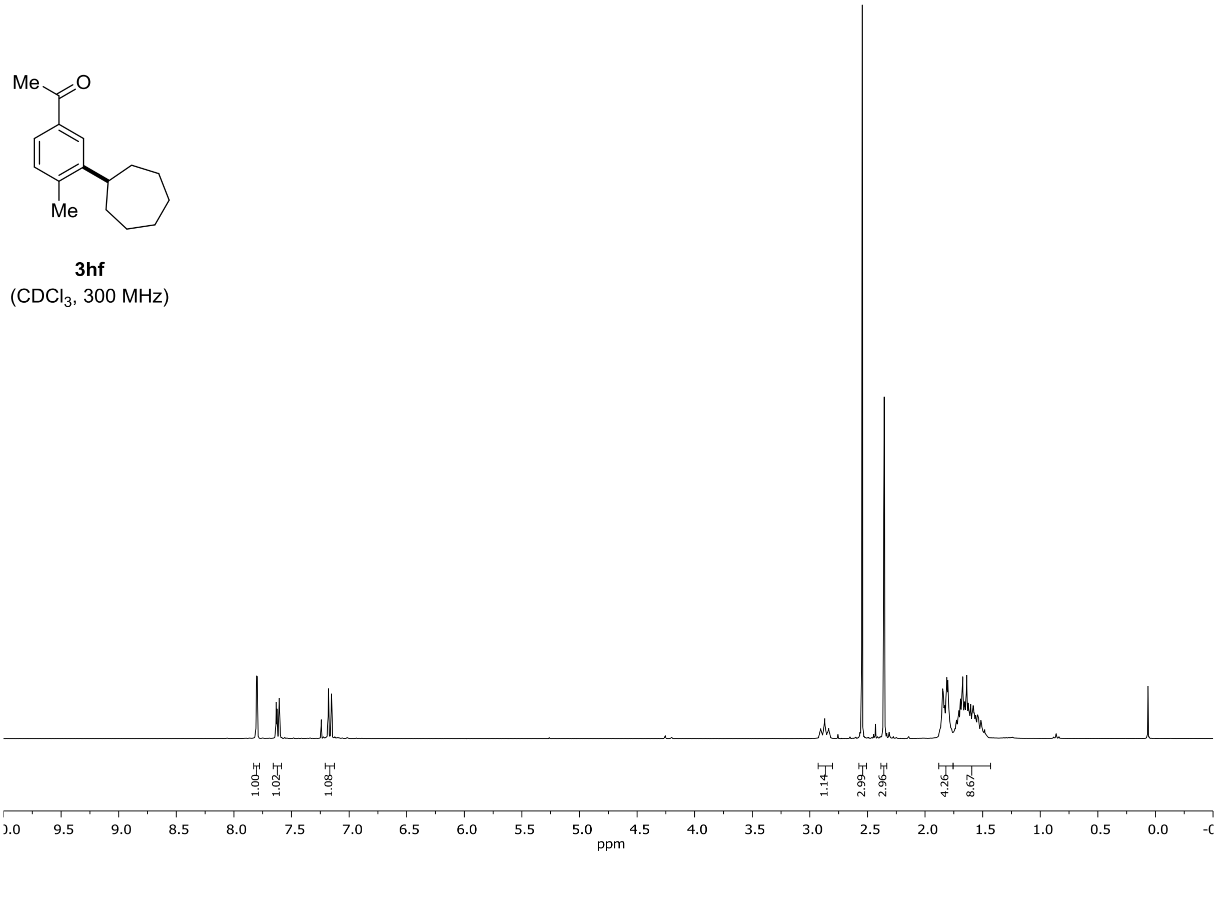

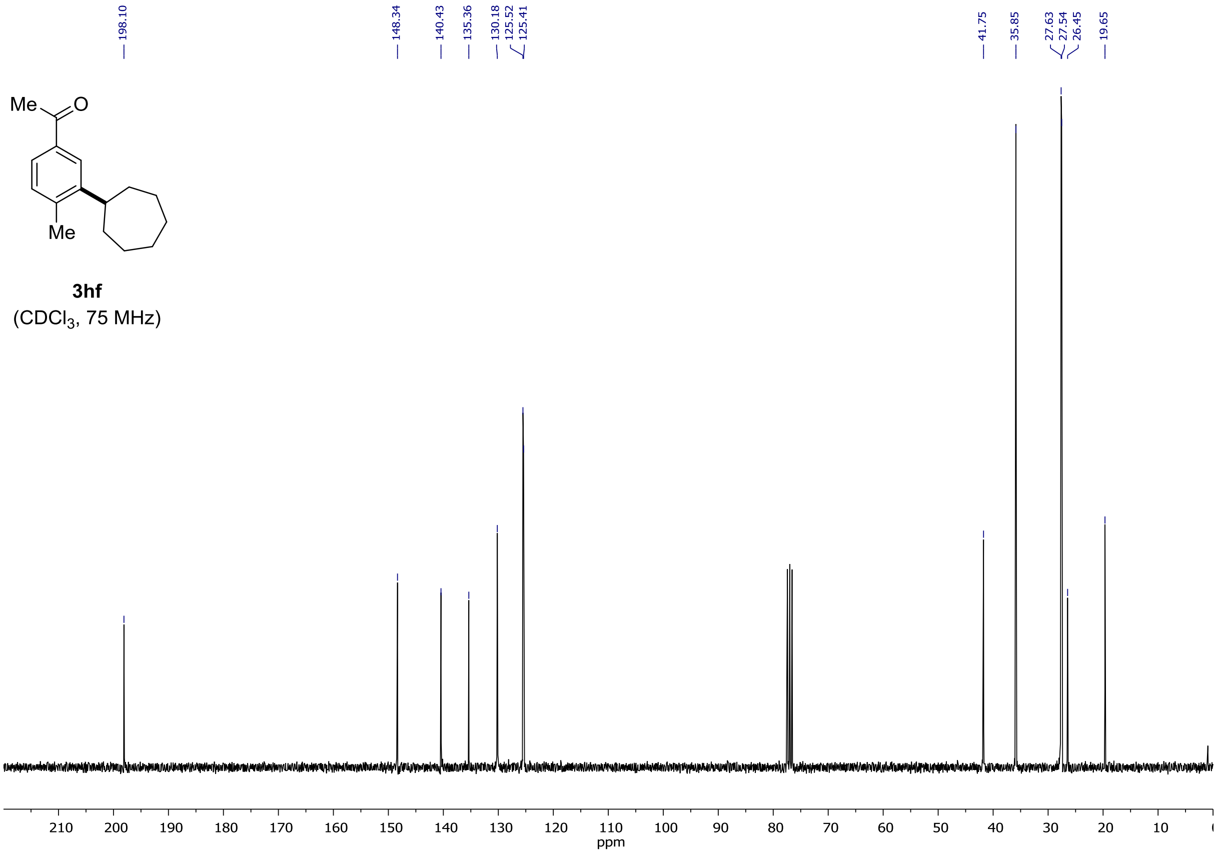

Supplementary Figure 19: ^{1}H and ^{13}C-NMR of Compound 3hf.

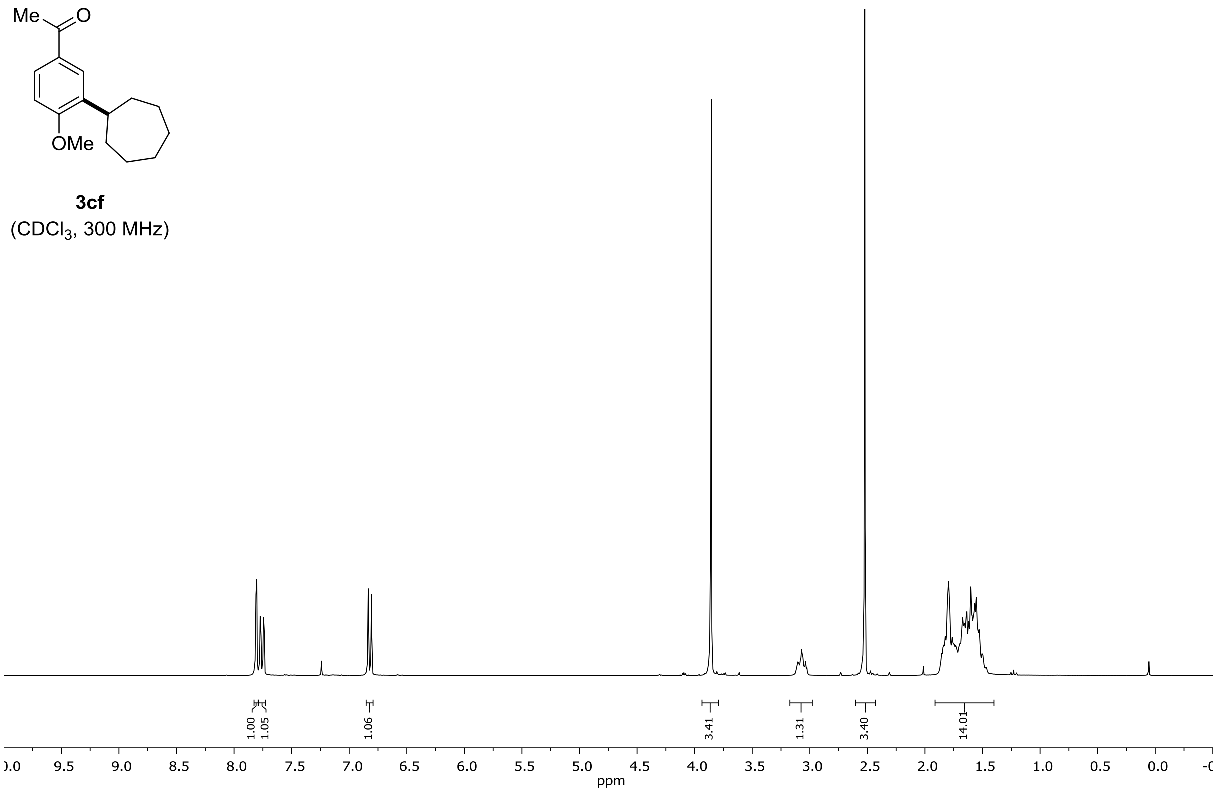

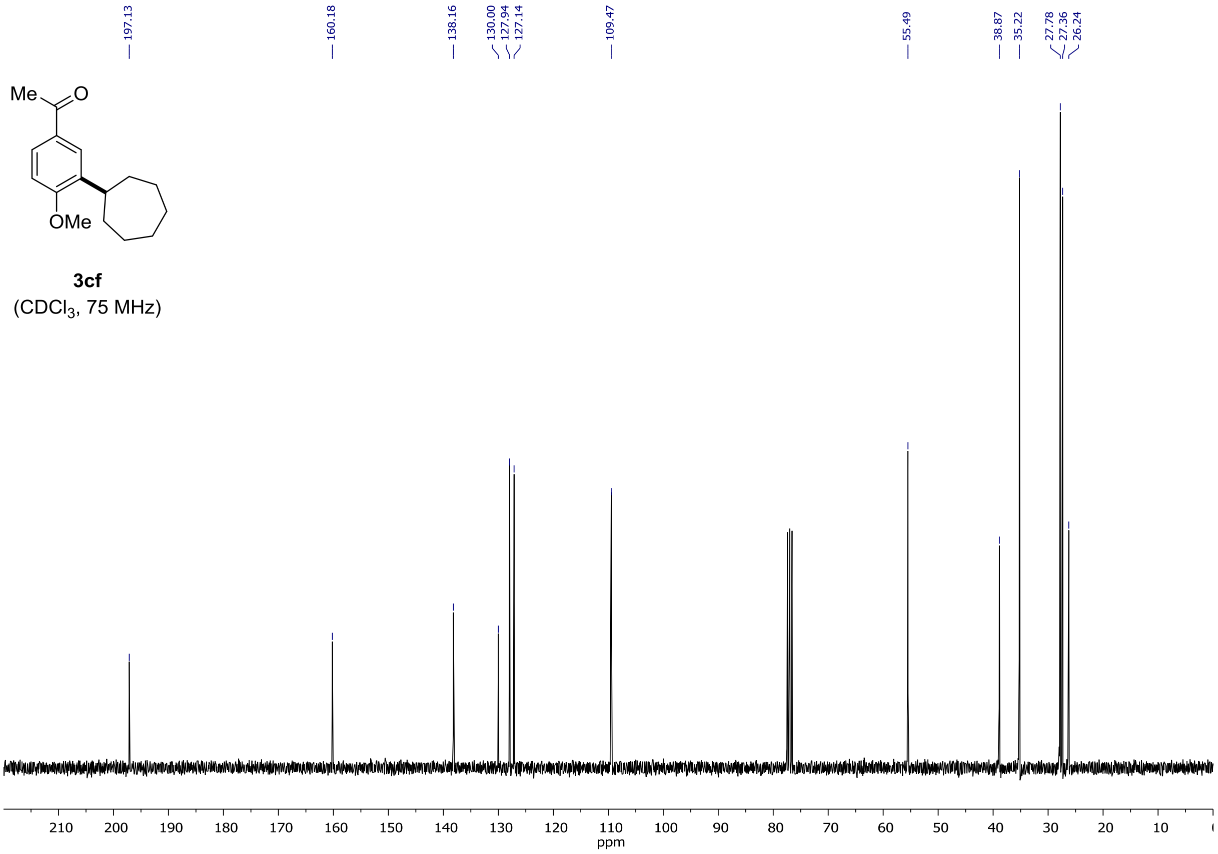

Supplementary Figure 20: ^{1}H and ^{13}C-NMR of Compound 3cf.

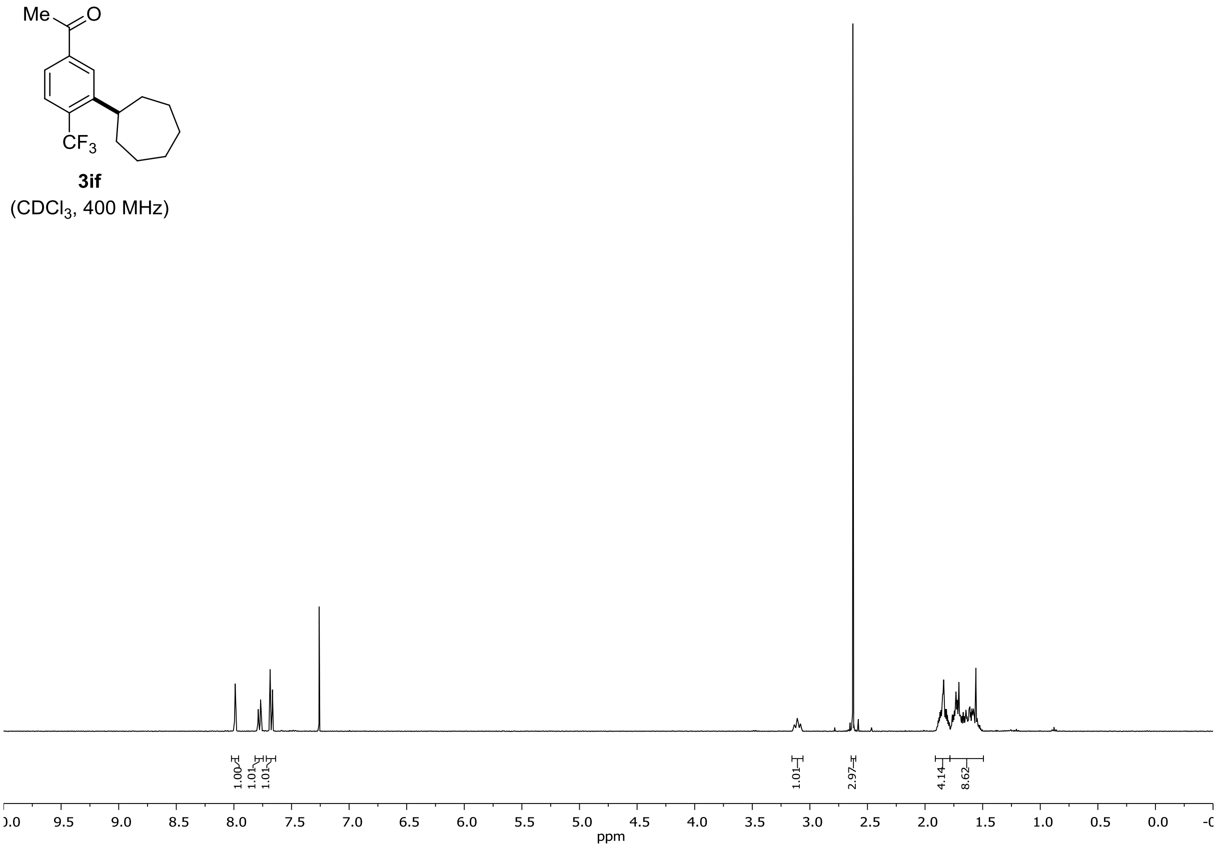

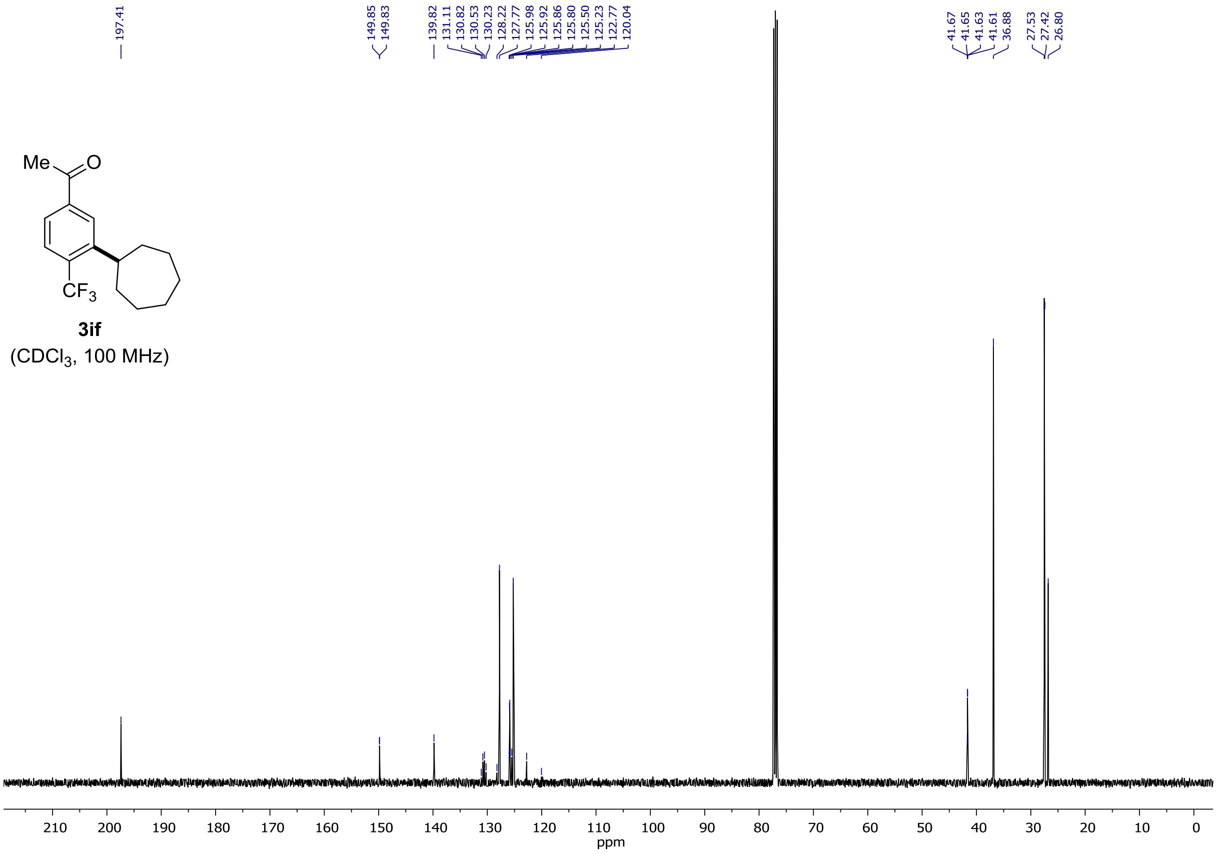

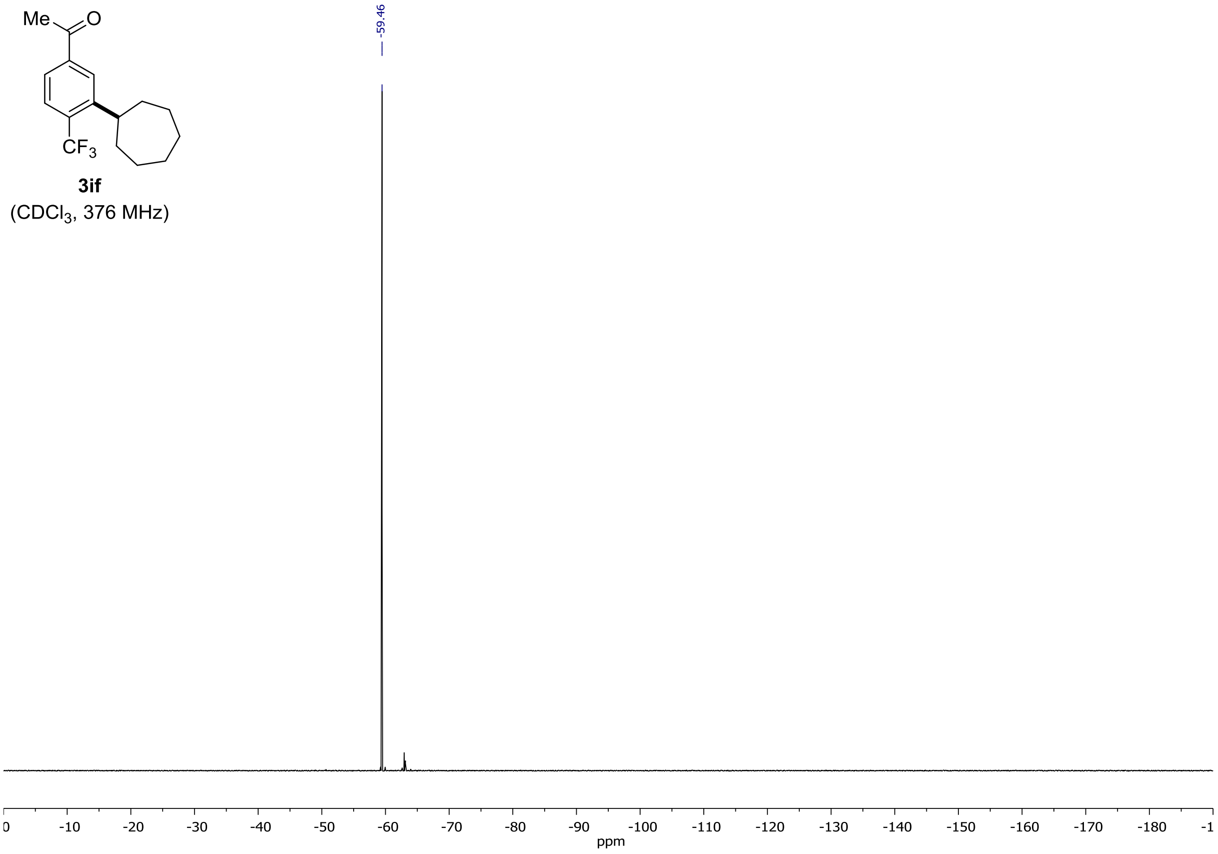

Supplementary Figure 21: ^{1}H, ^{13}C and ^{19}F-NMR of Compound 3if.

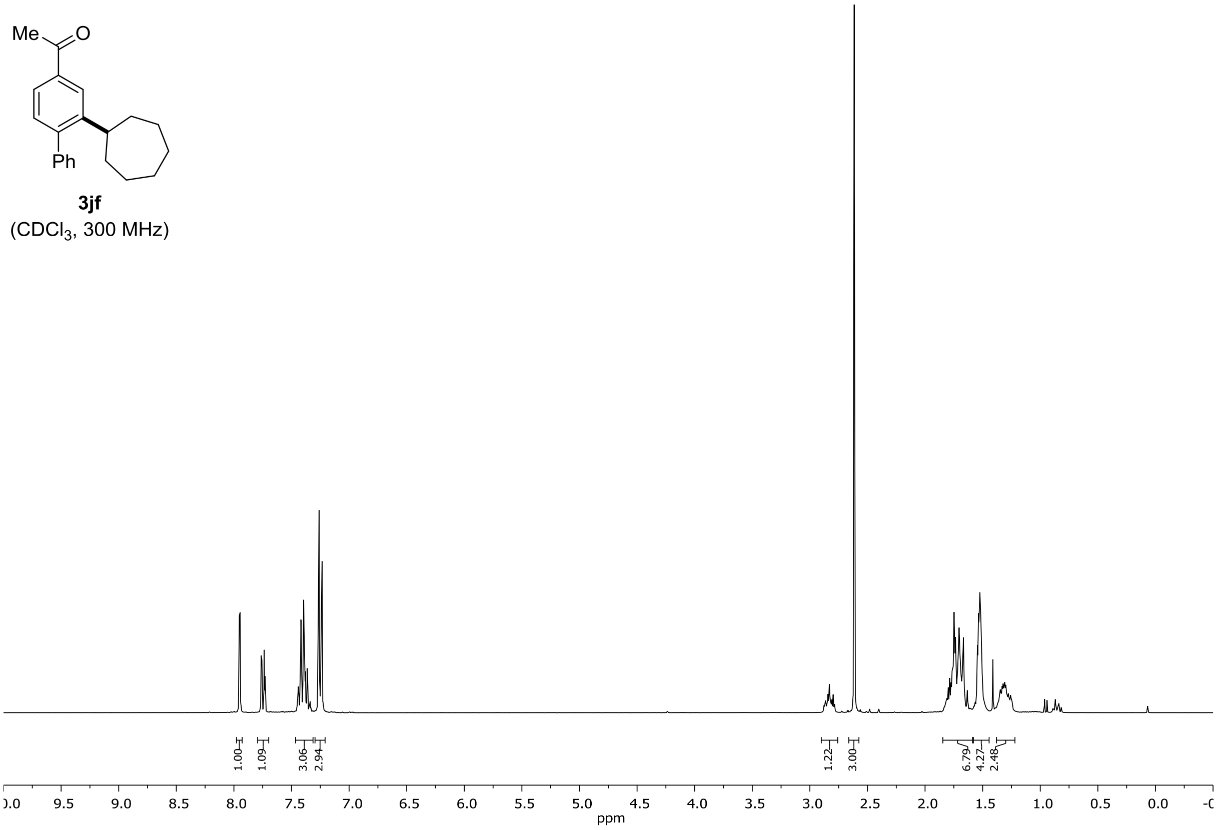

3jf
(CDCl$_3$, 300 MHz)

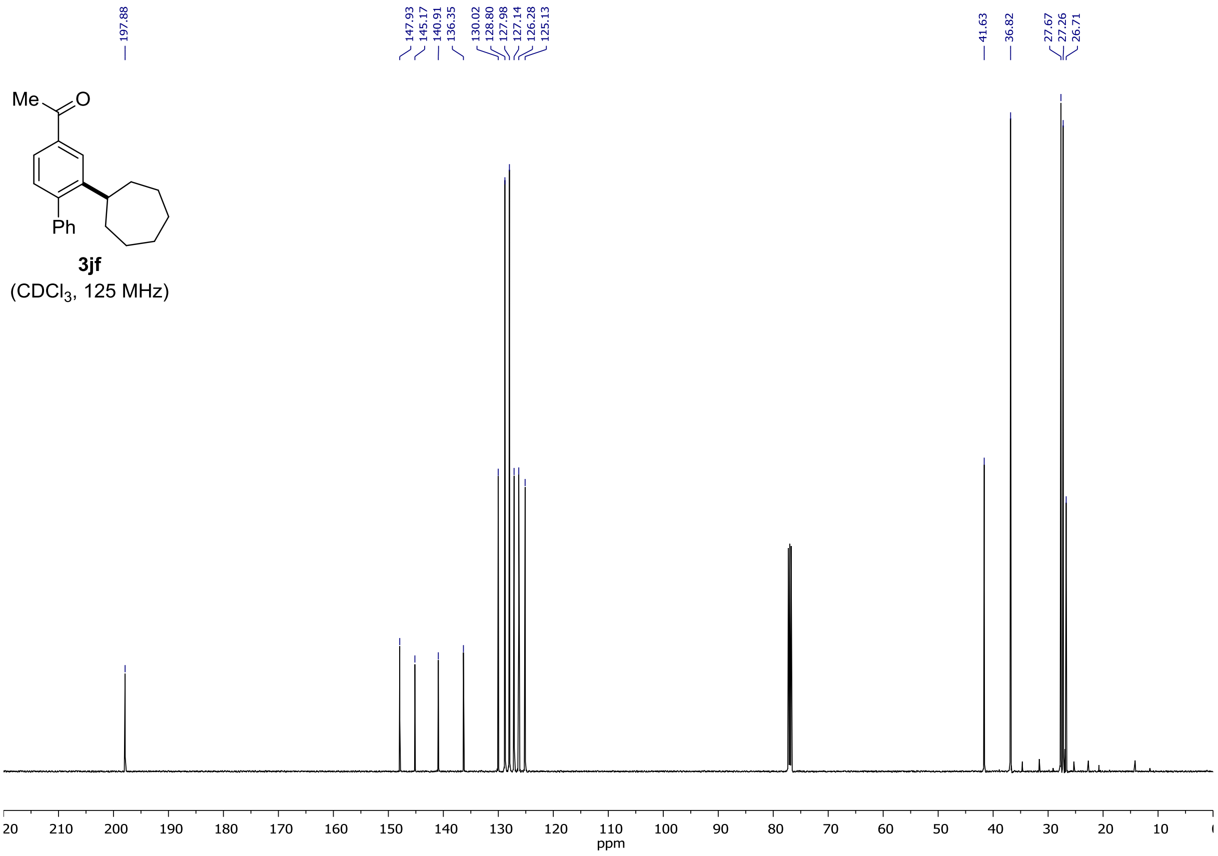

3jf
(CDCl$_3$, 125 MHz)

Supplementary Figure 22: ^{1}H and ^{13}C-NMR of Compound 3jf.

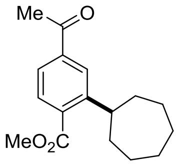

3kf
($CDCl_3$, 300 MHz)

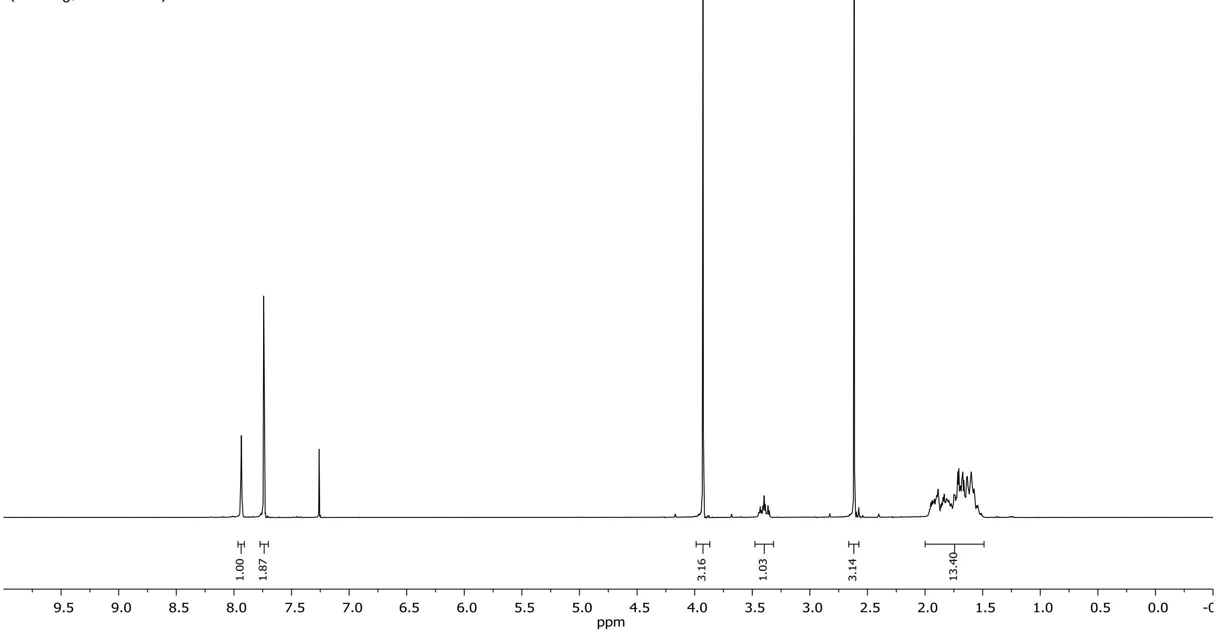

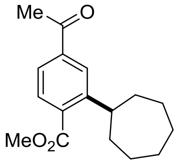

3kf
($CDCl_3$, 125 MHz)

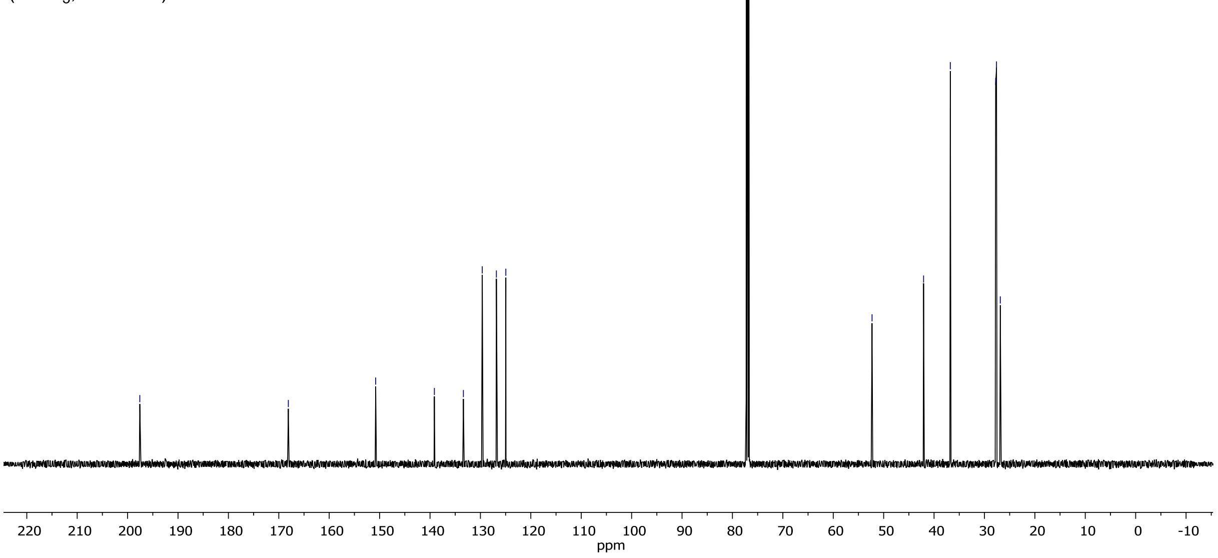

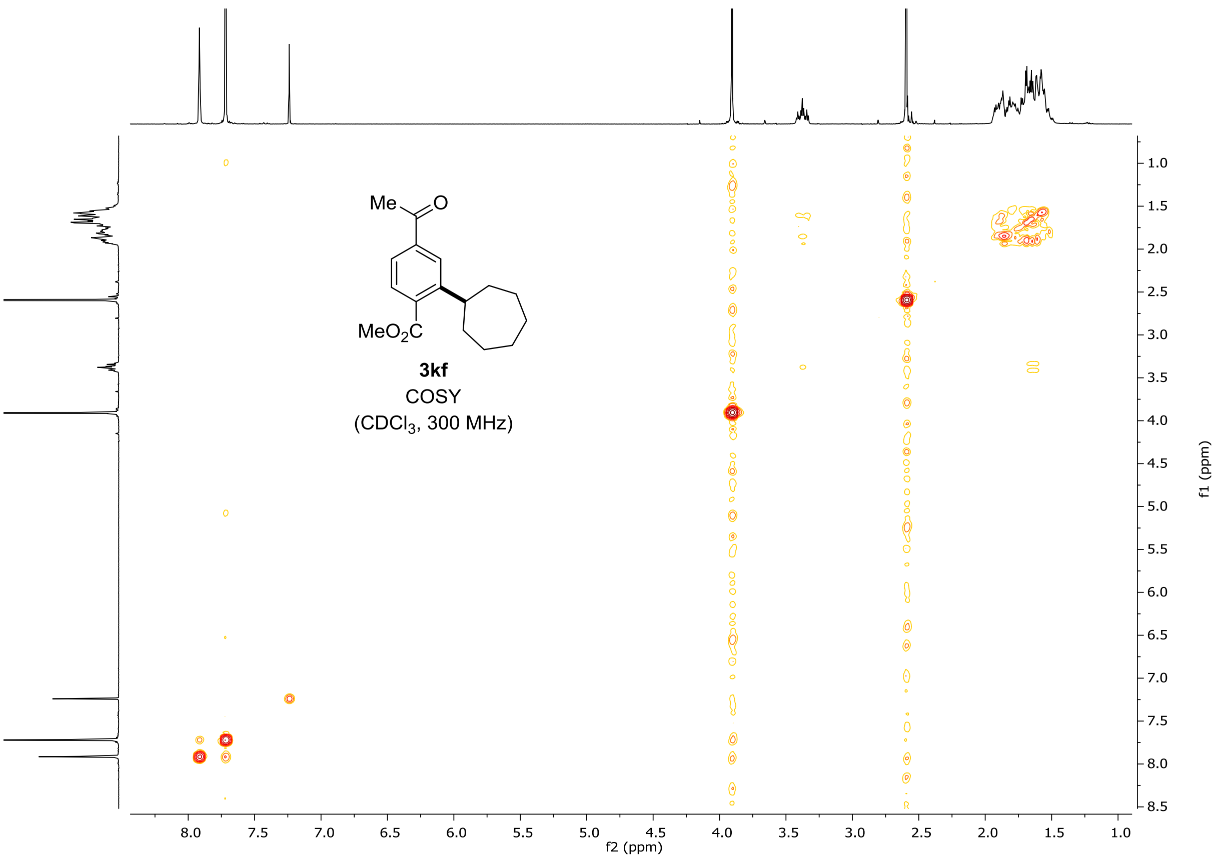
Me
O
MeO2C
3kf
COSY
(CDCl3, 300 MHz)
f2 (ppm)
f1 (ppm)

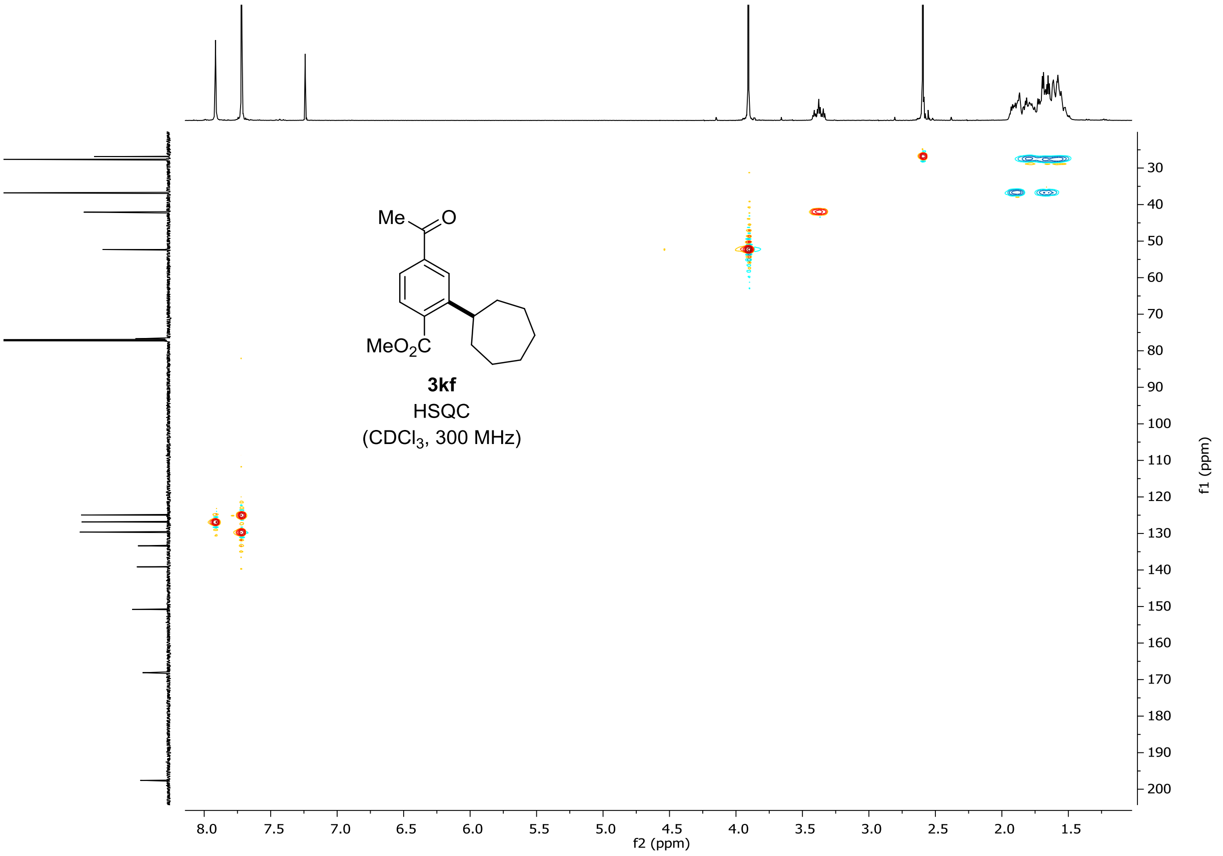
Me
O
MeO2C
3kf
HSQC
(CDCl3, 300 MHz)
f2 (ppm)
f1 (ppm)

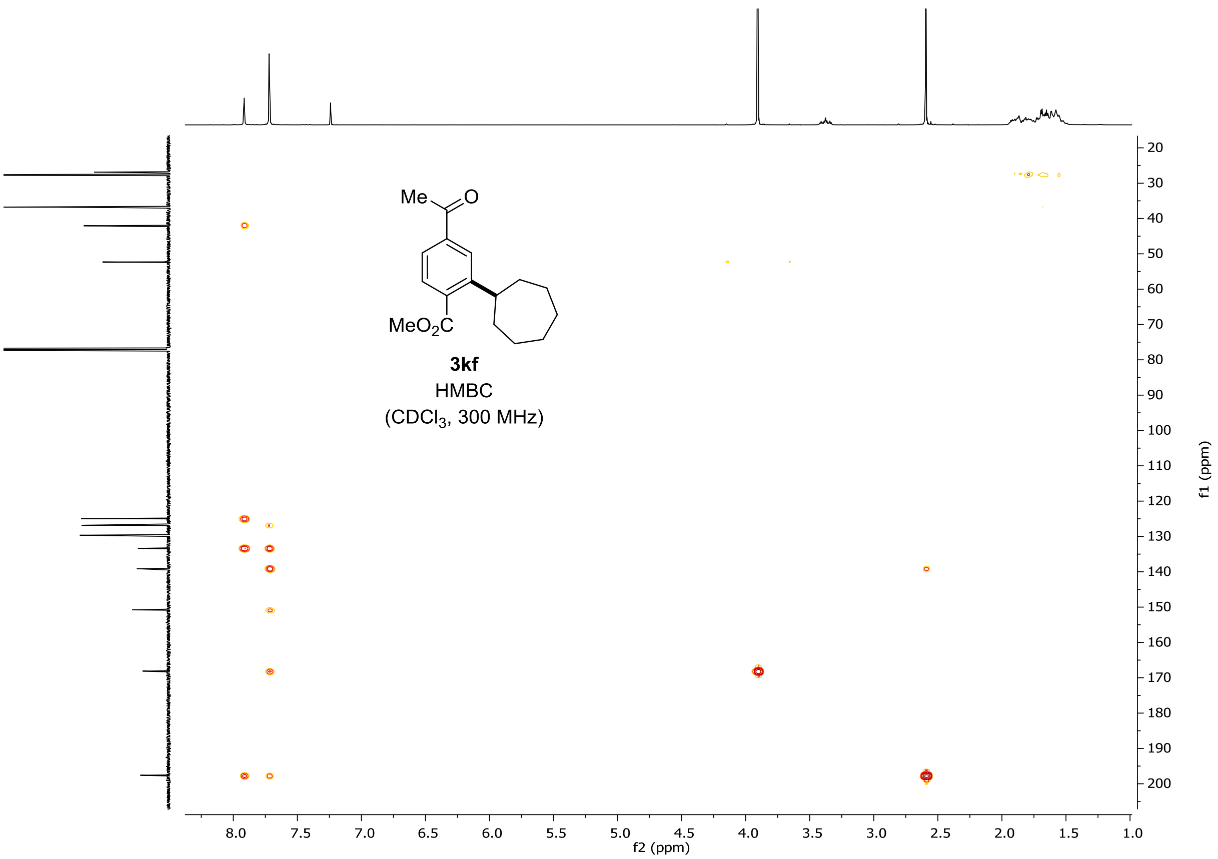

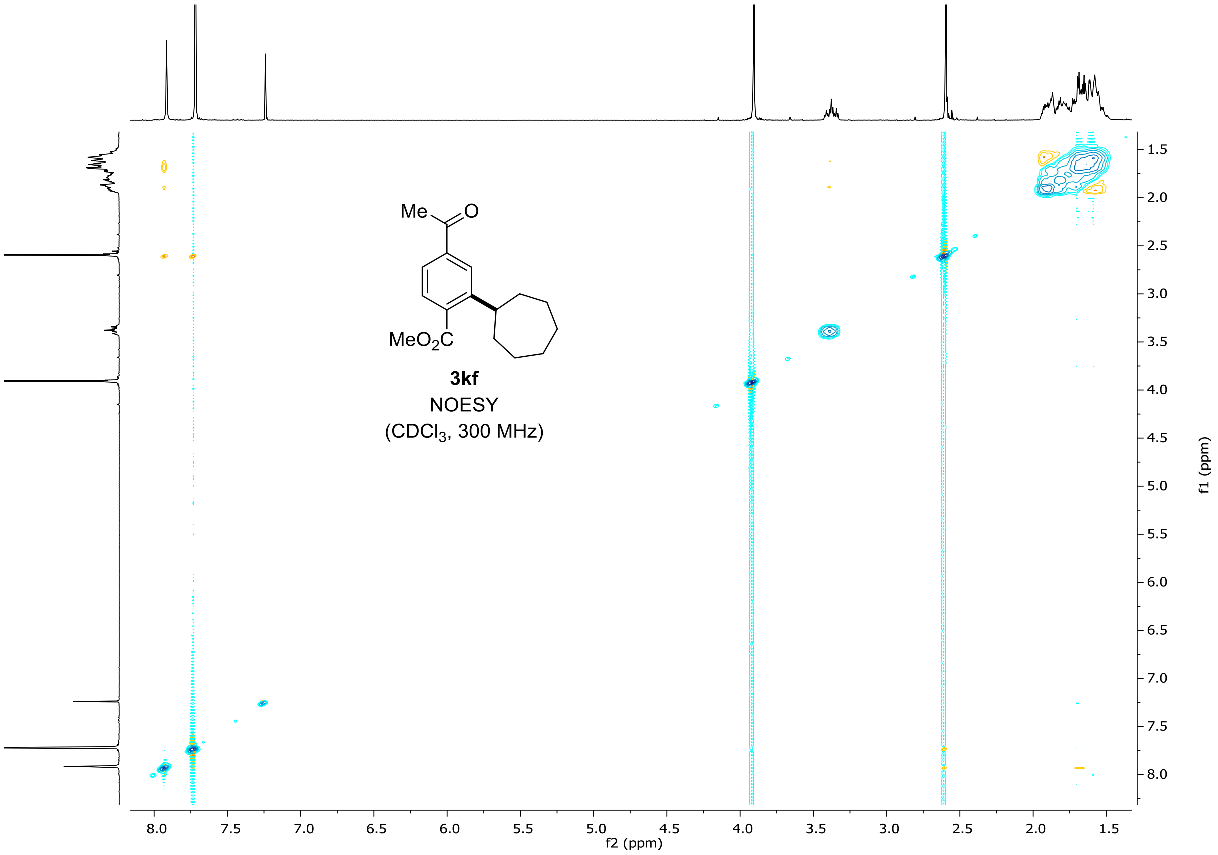

Supplementary Figure 23: ^{1}H, ^{13}C, COSY, HSQC, HMBC and NOESY-NMR of Compound 3kf.

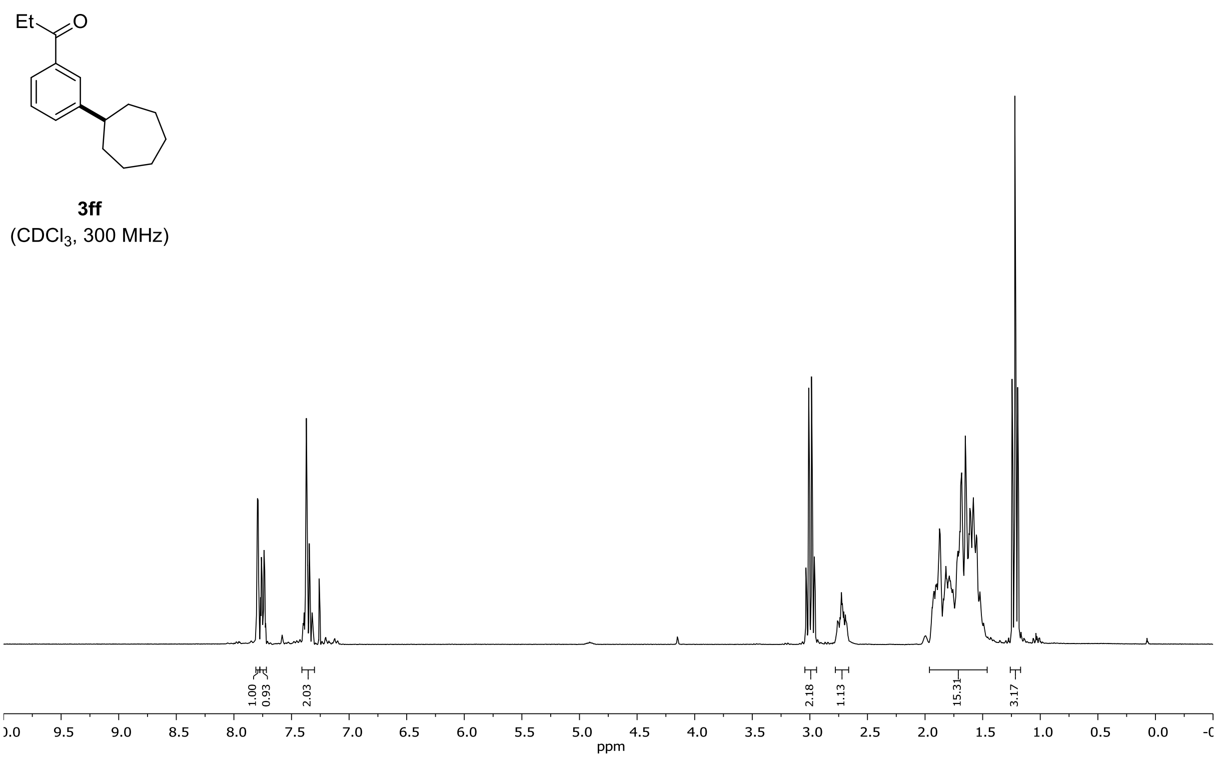

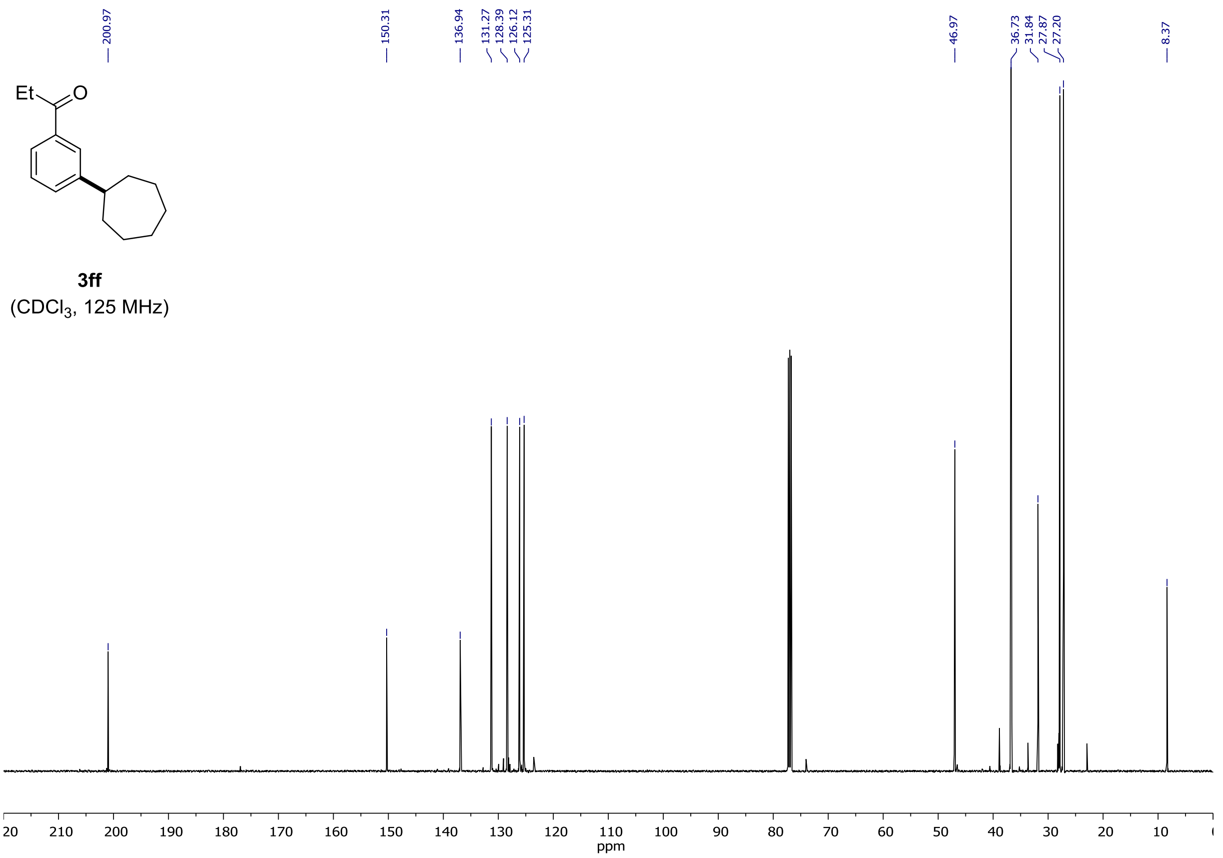

Supplementary Figure 24: ^{1}H and ^{13}C-NMR of Compound 3ff.

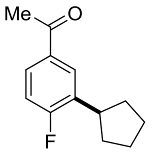

3ag
(CDCl$_3$, 300 MHz)

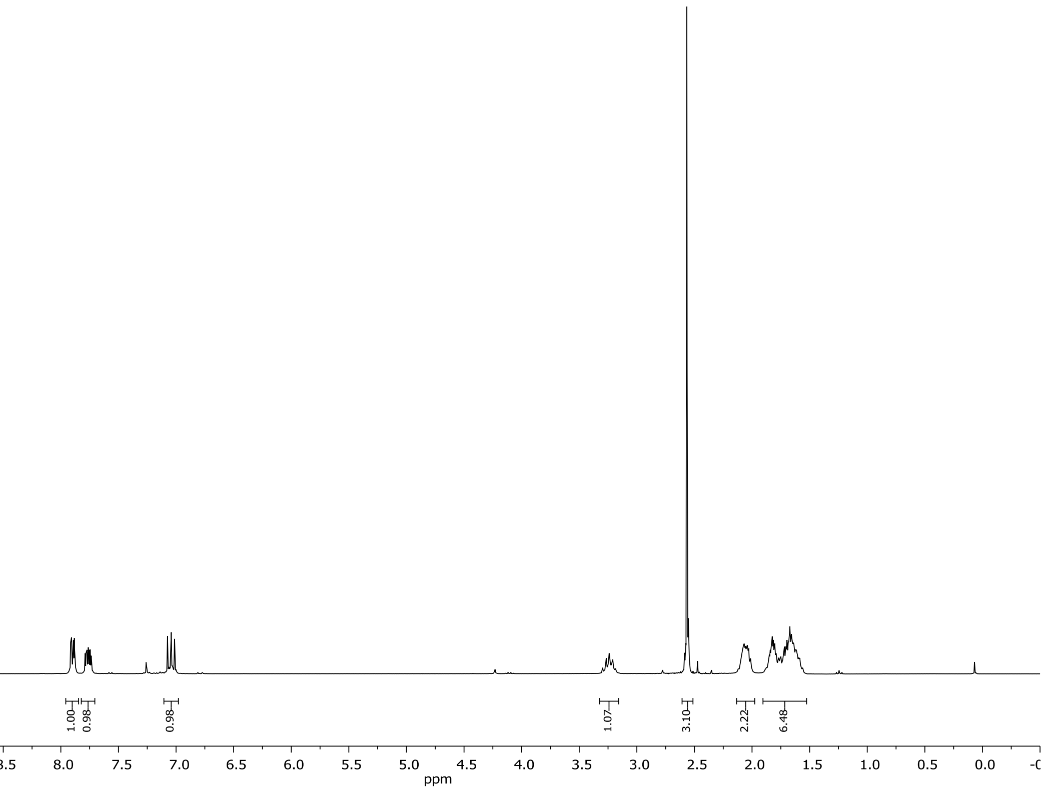

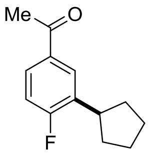

3ag
(CDCl$_3$, 125 MHz)

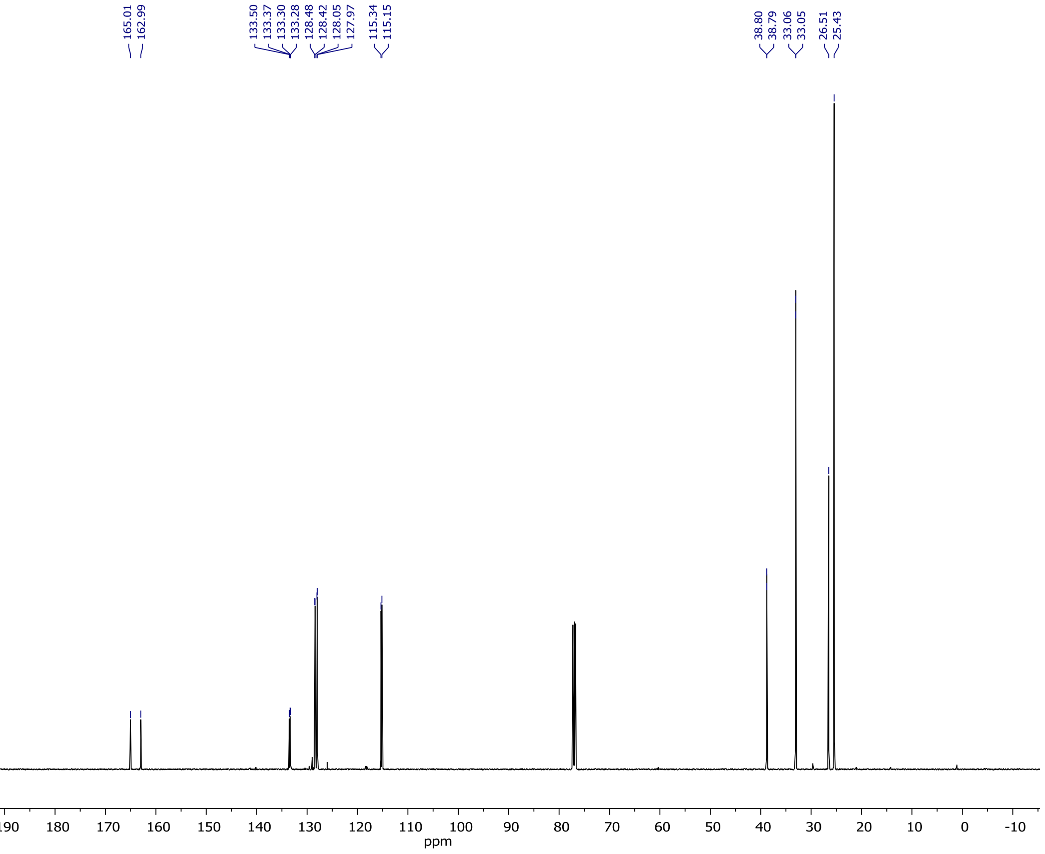

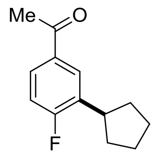

3ag
($CDCl_3$, 282 MHz)

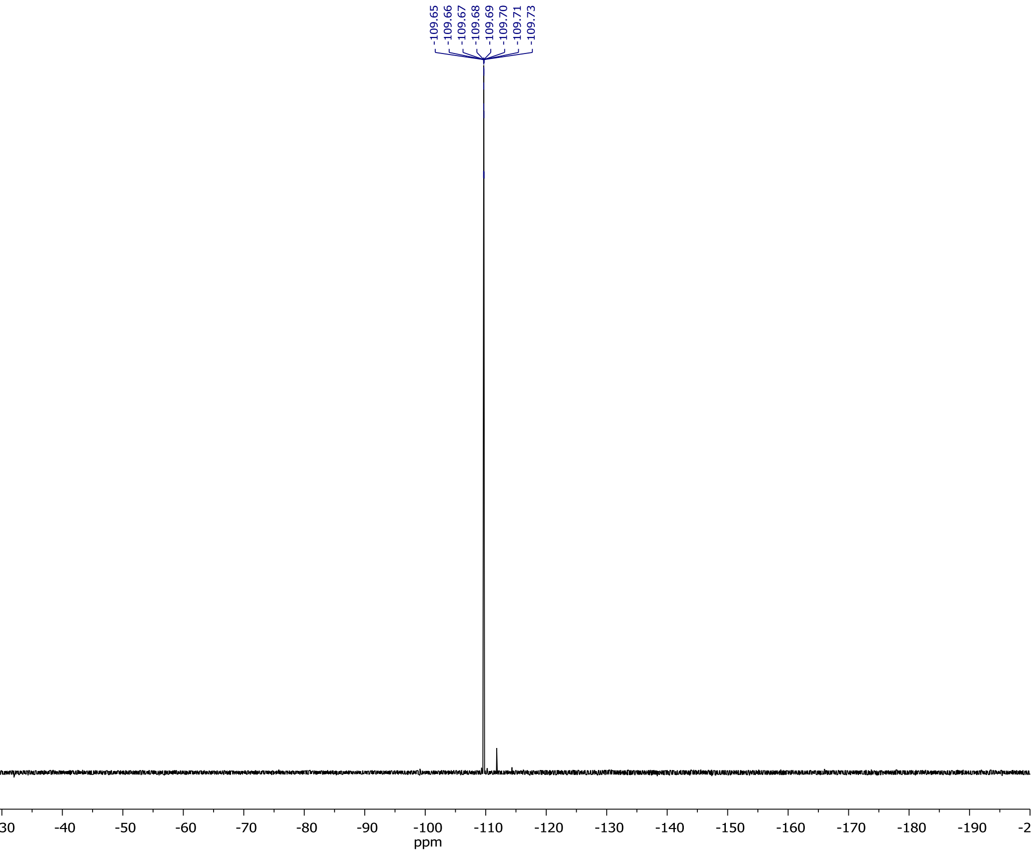

Supplementary Figure 25: ^{1}H, ^{13}C and ^{19}F-NMR of Compound 3ag.

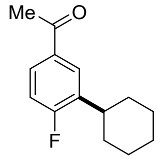

3ah
($CDCl_3$, 300 MHz)

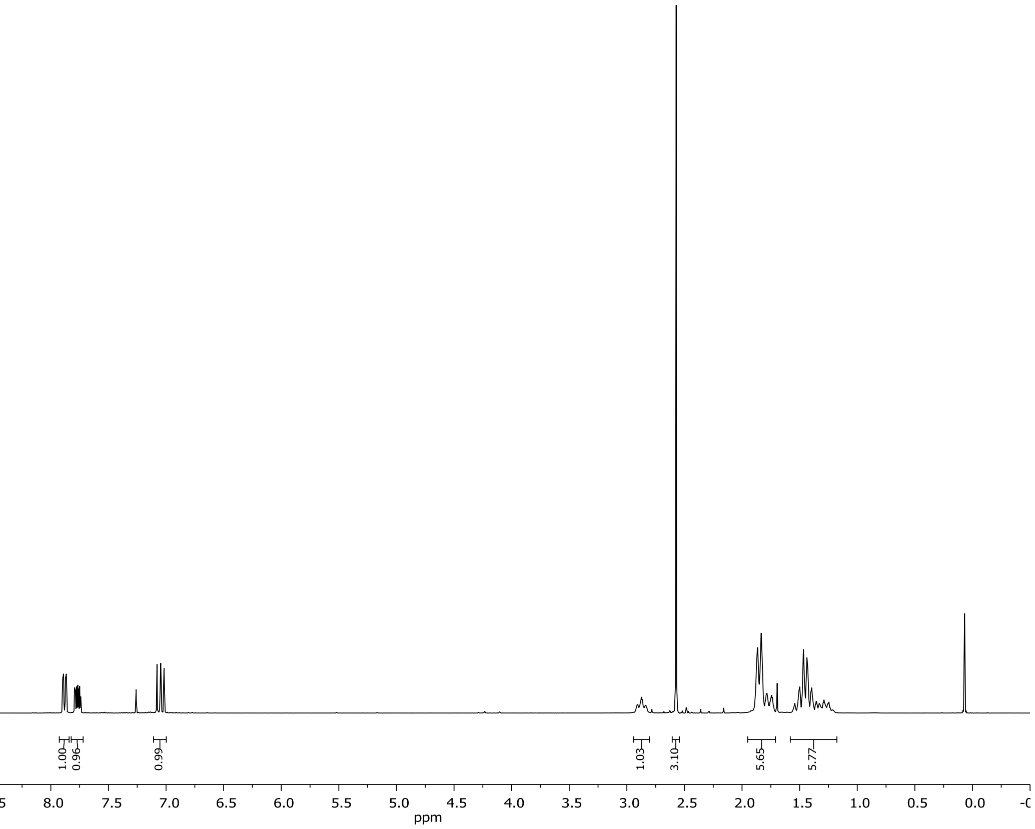

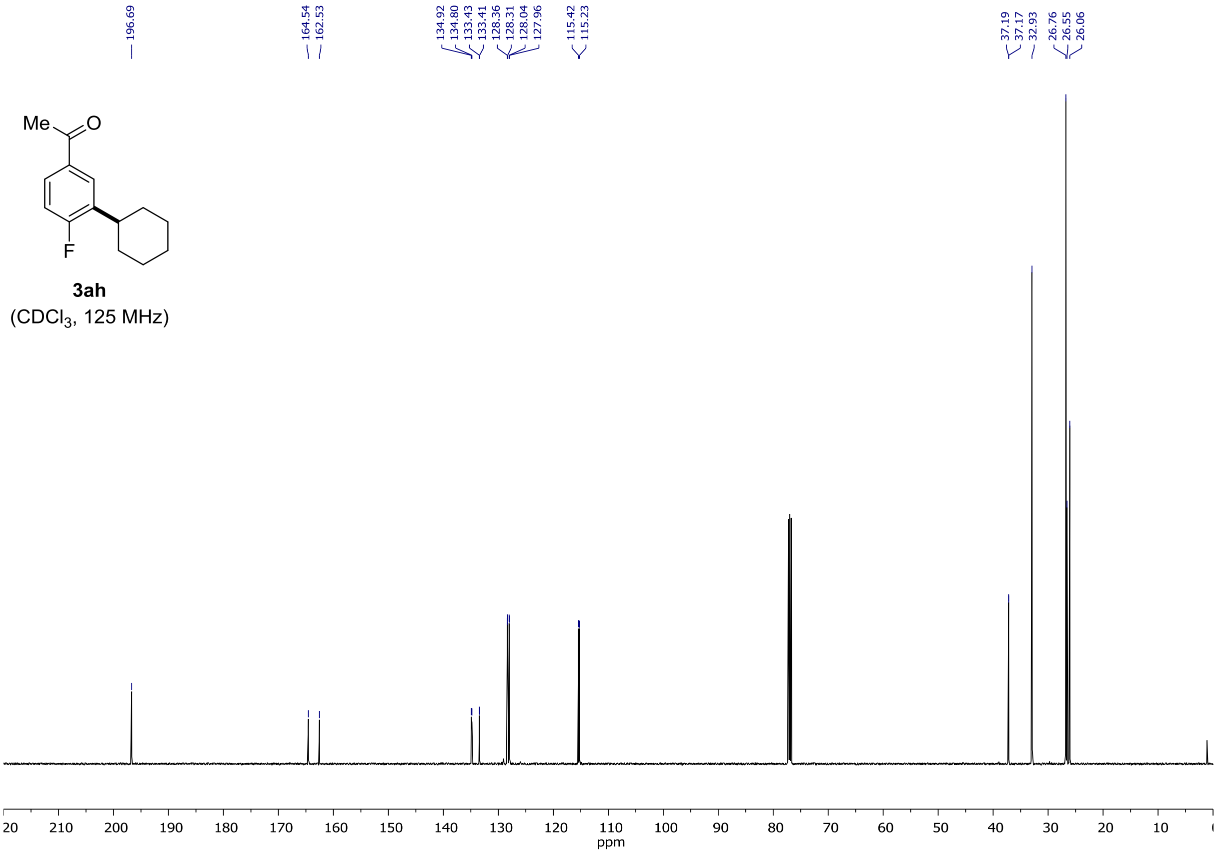

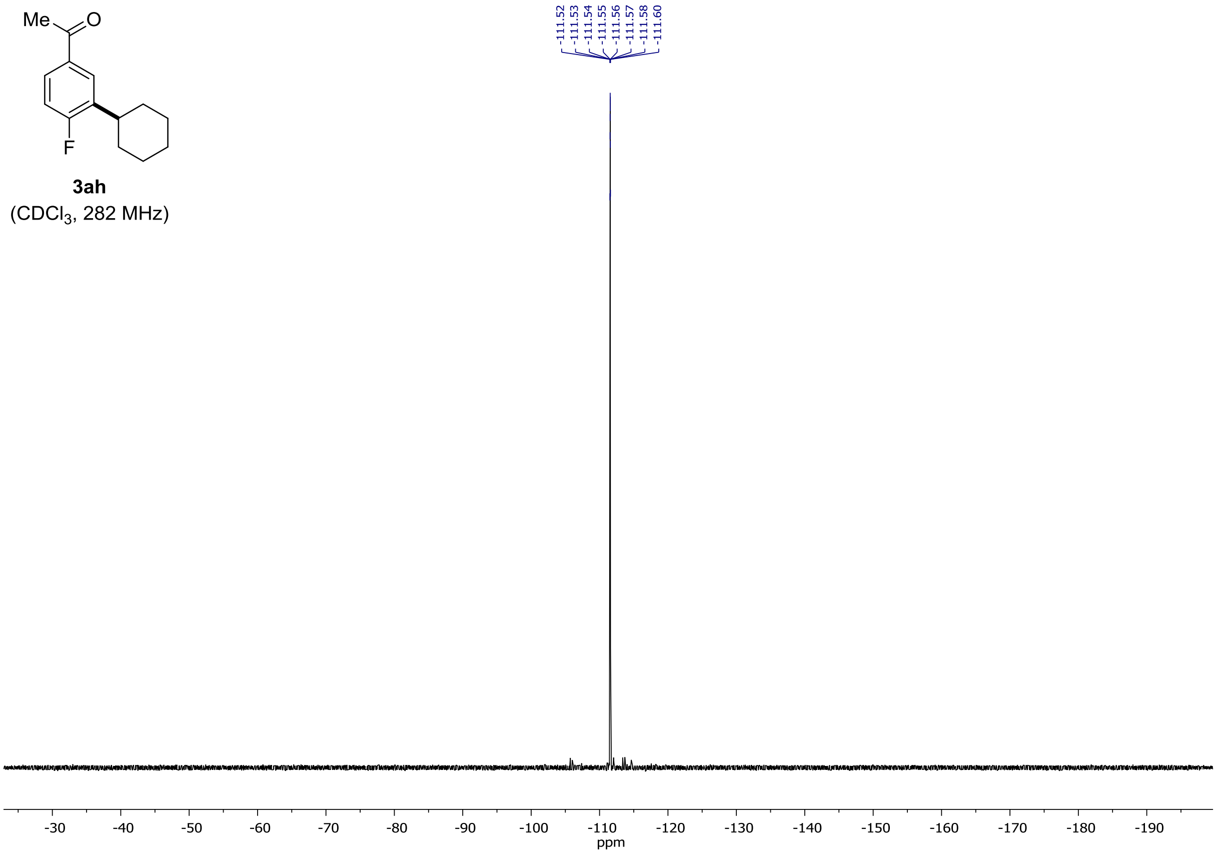

Supplementary Figure 26: ^{1}H, ^{13}C and ^{19}F-NMR of Compound 3ah.

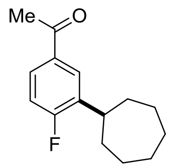

3af
($CDCl_3$, 300 MHz)

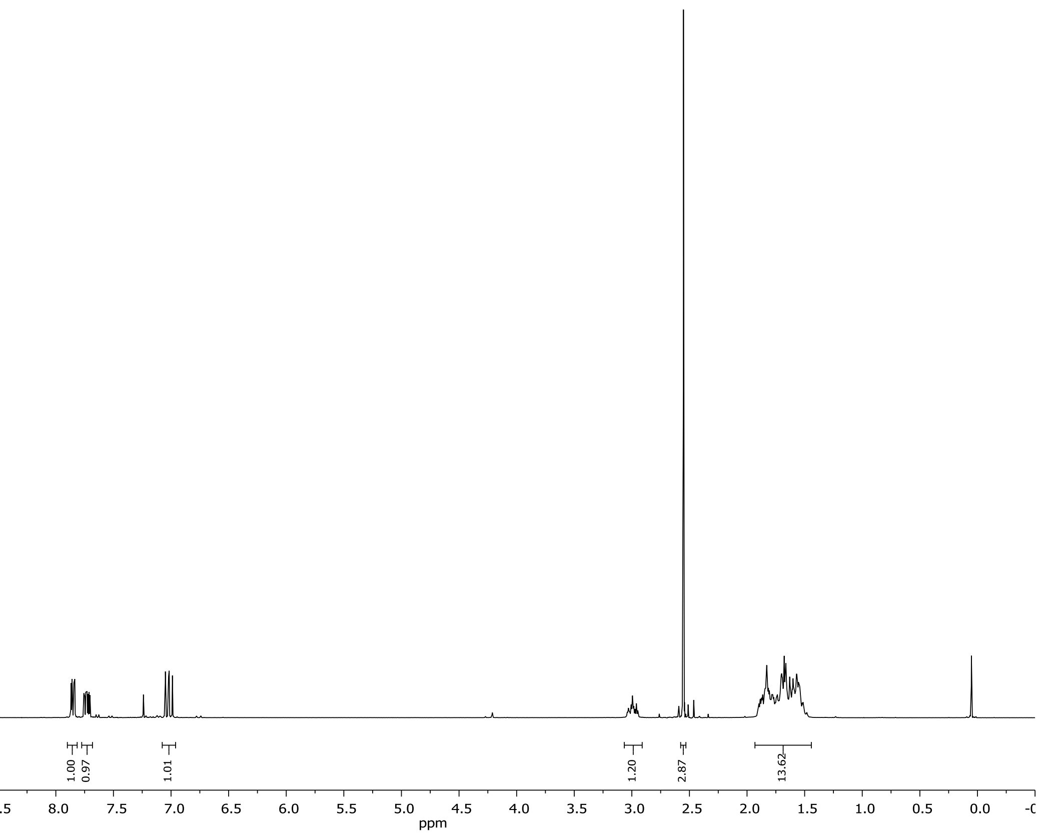

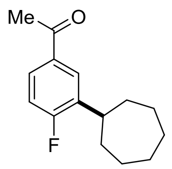

3af
($CDCl_3$, 75 MHz)

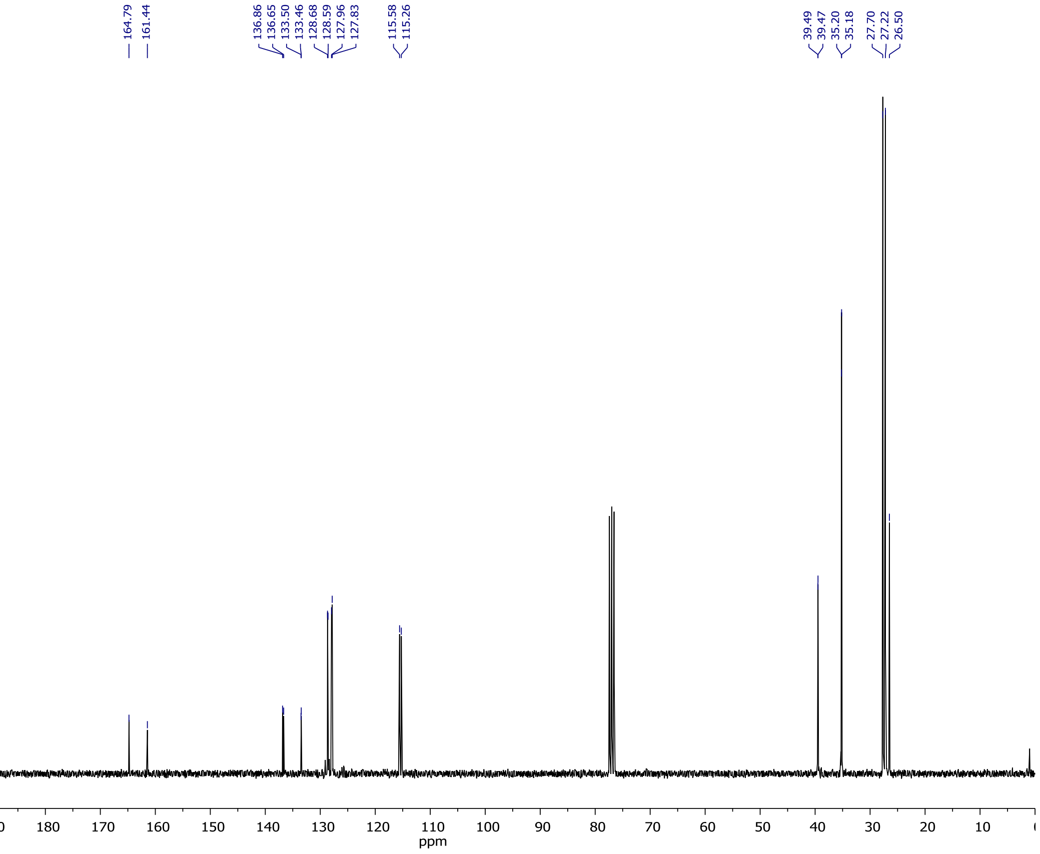

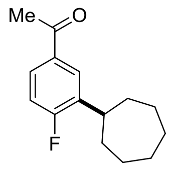

3af
($CDCl_3$, 282 MHz)

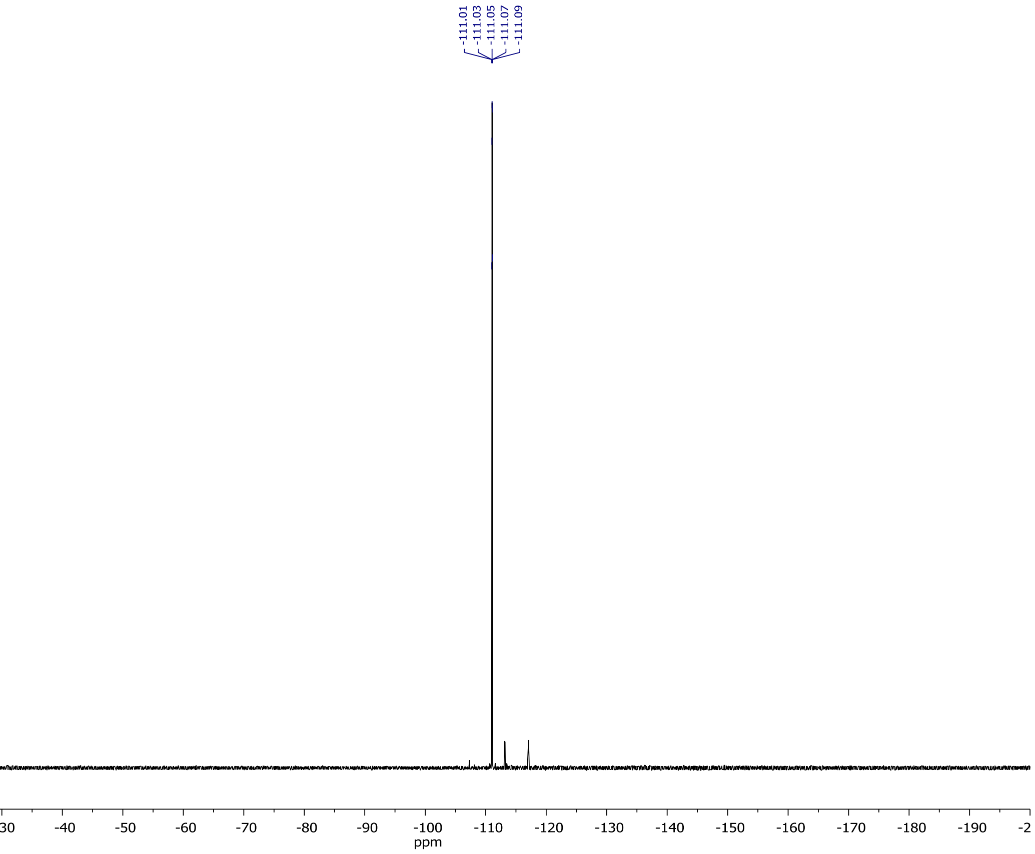

Supplementary Figure 27: ^{1}H, ^{13}C and ^{19}F-NMR of Compound 3af.

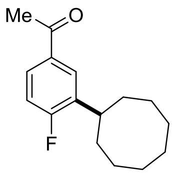

3ai
($CDCl_3$, 300 MHz)

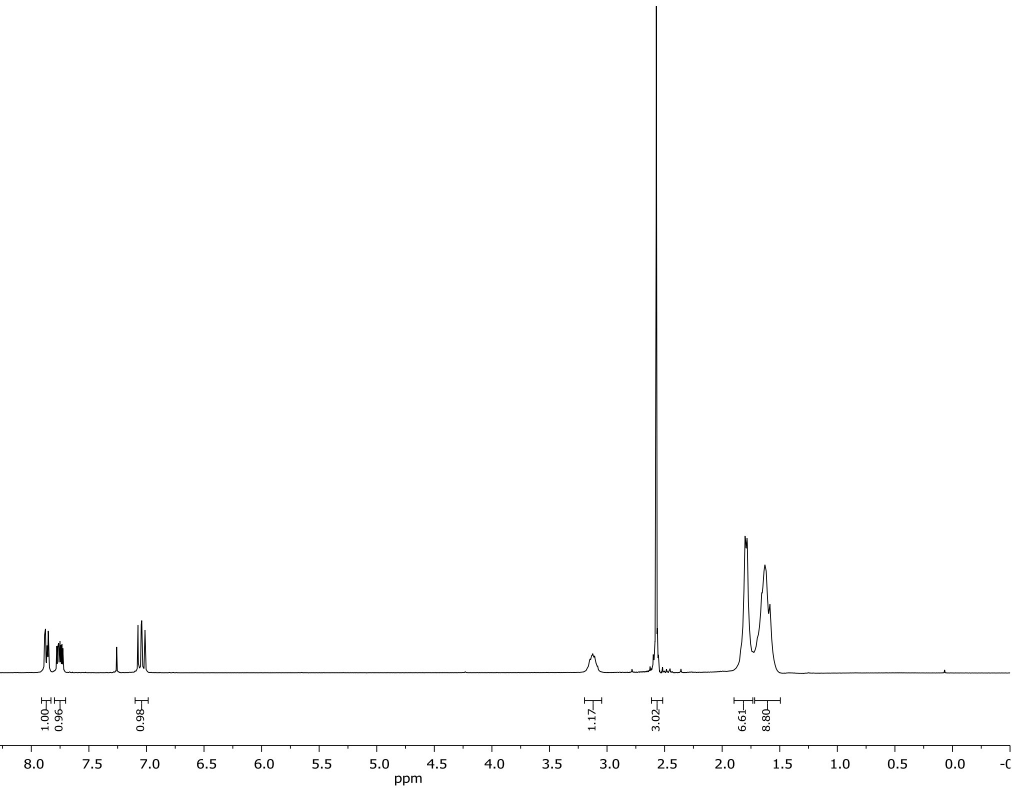

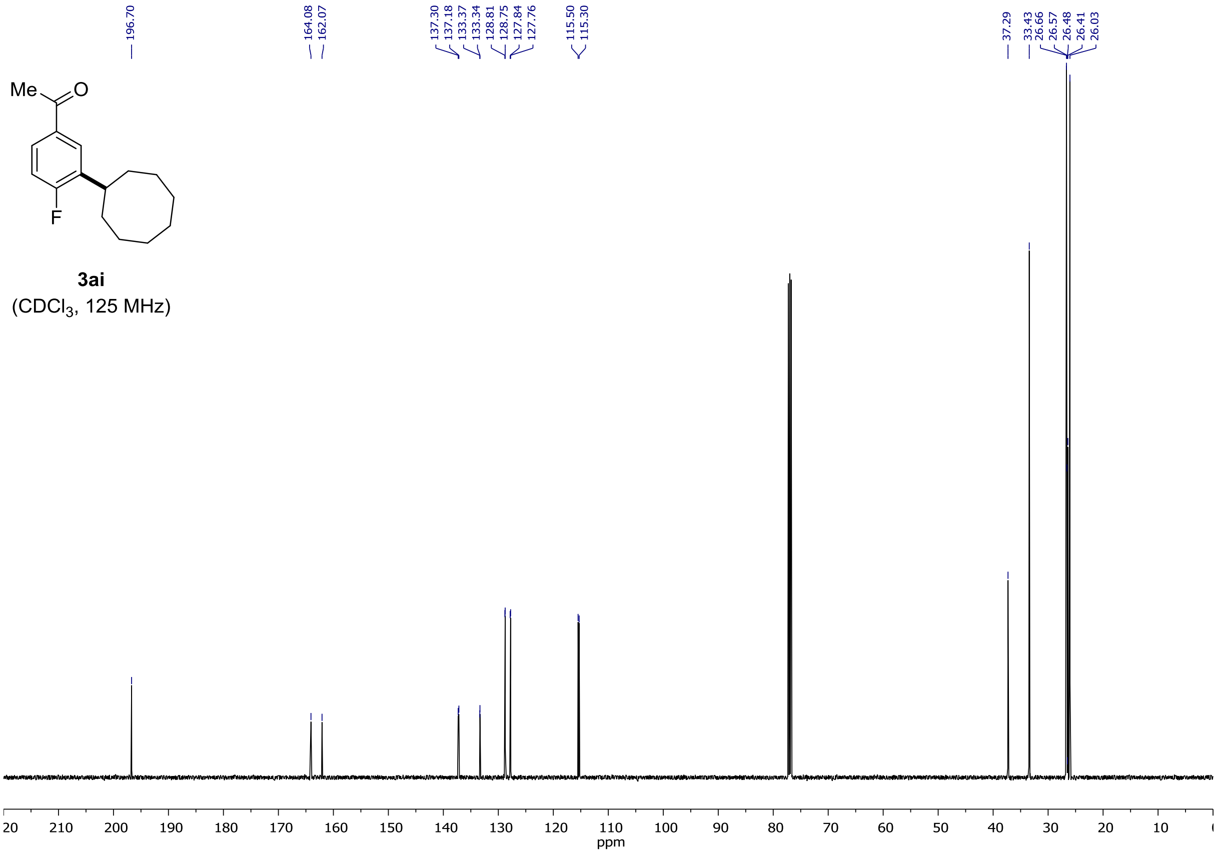

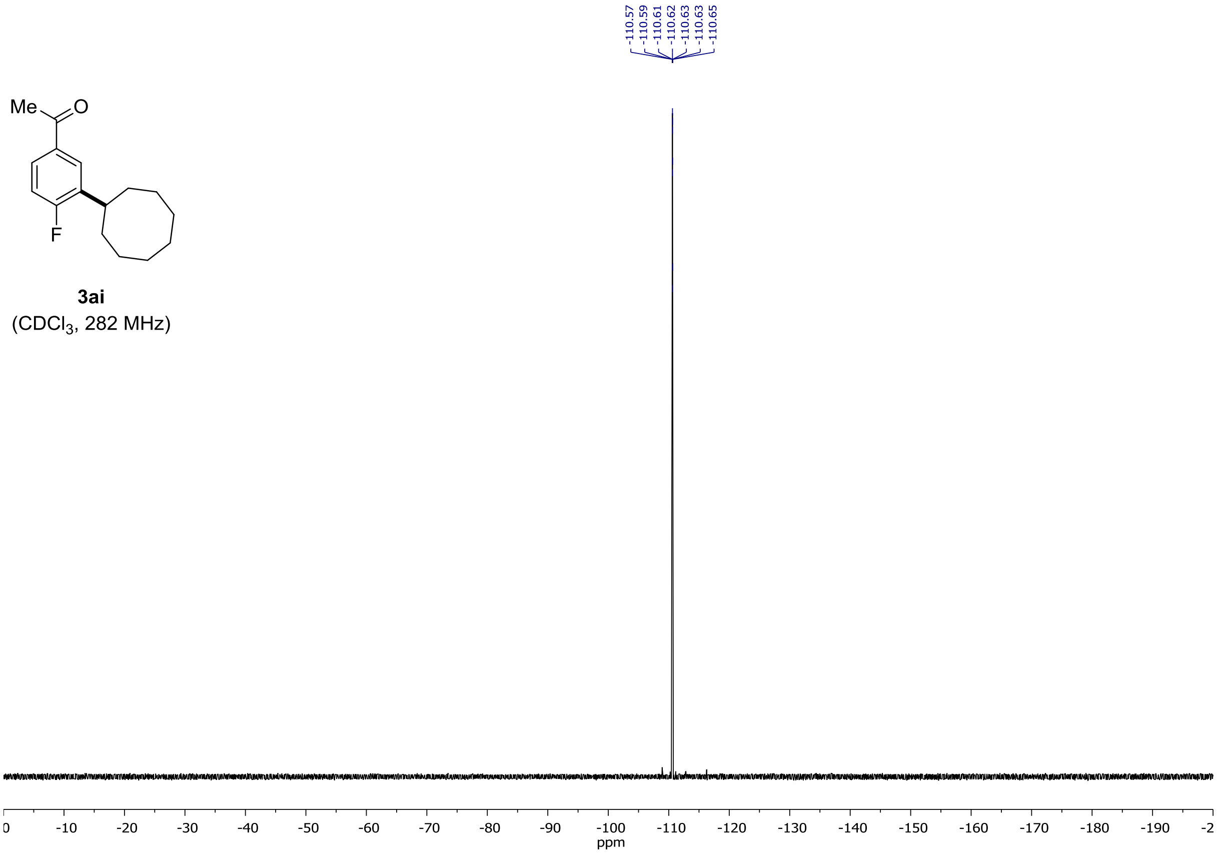

Supplementary Figure 28: ^{1}H, ^{13}C and ^{19}F-NMR of Compound 3ai.

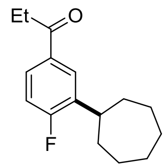

3ef
($CDCl_3$, 300 MHz)

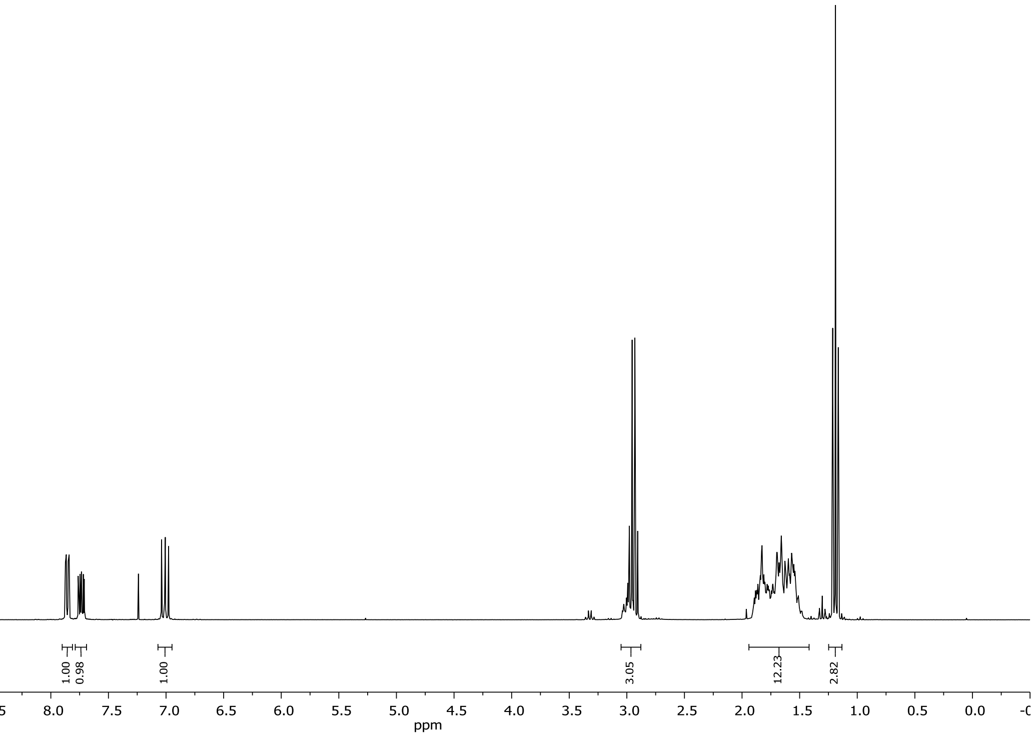

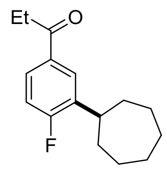

3ef
($CDCl_3$, 125 MHz)

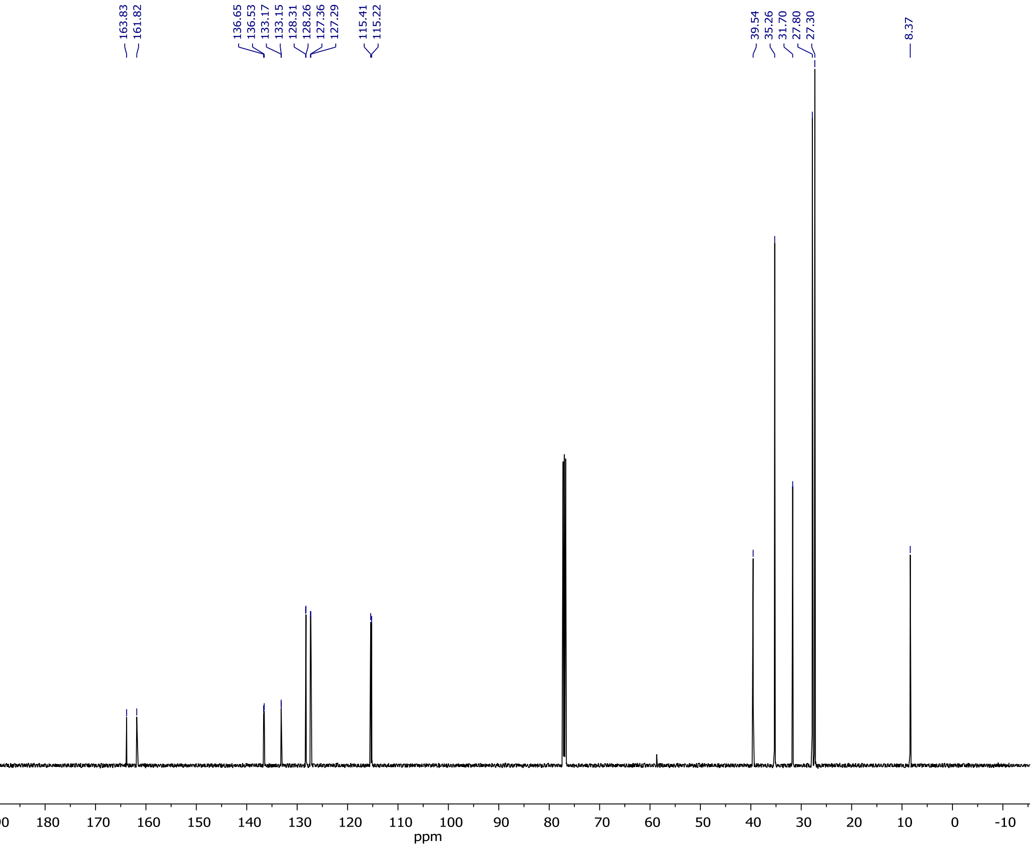

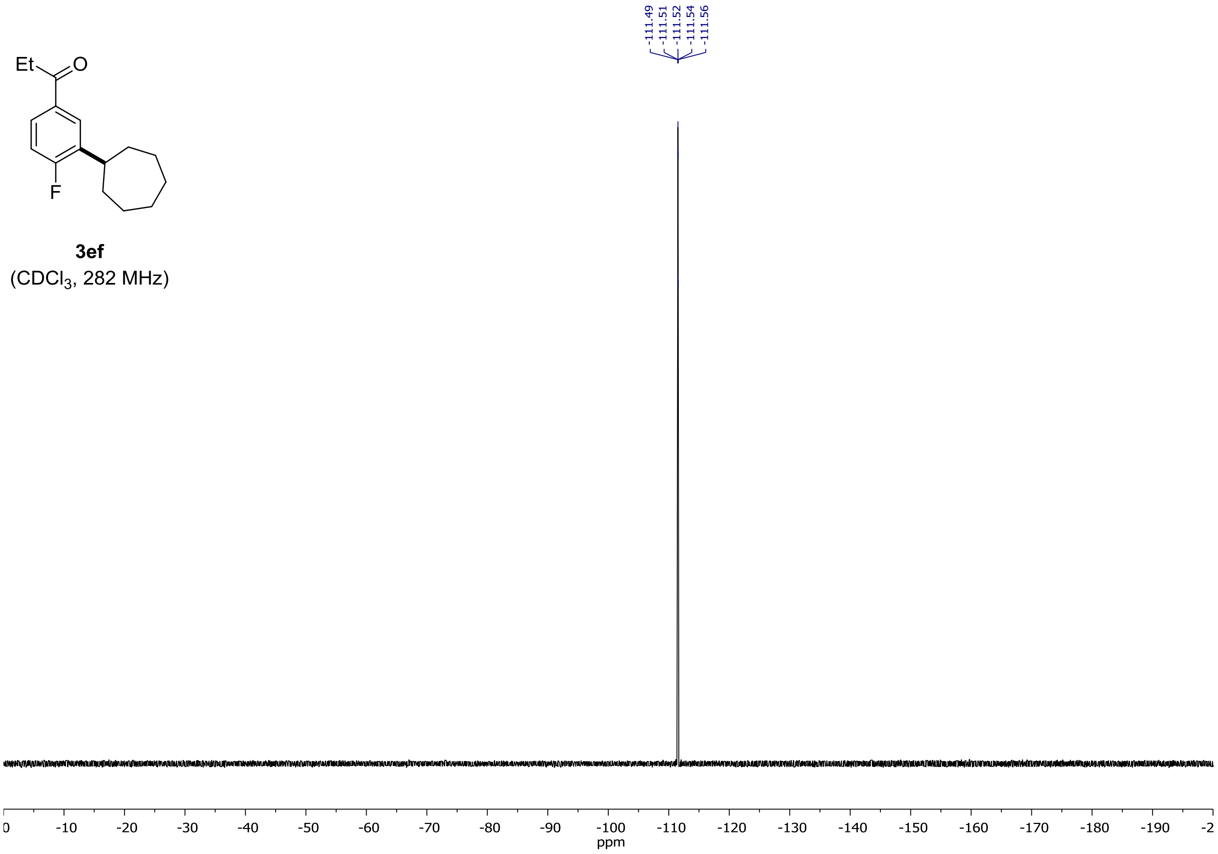

Supplementary Figure 29: ^{1}H, ^{13}C and ^{19}F-NMR of Compound 3ef.

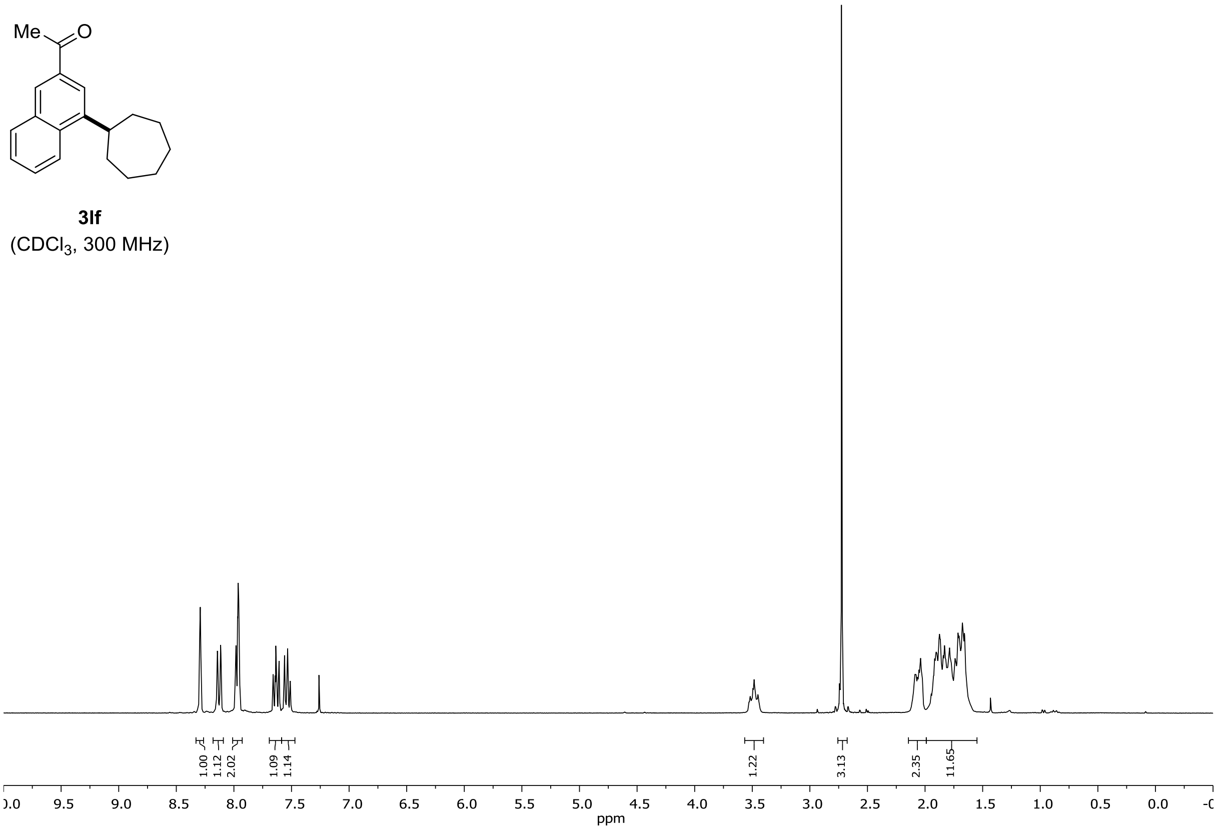

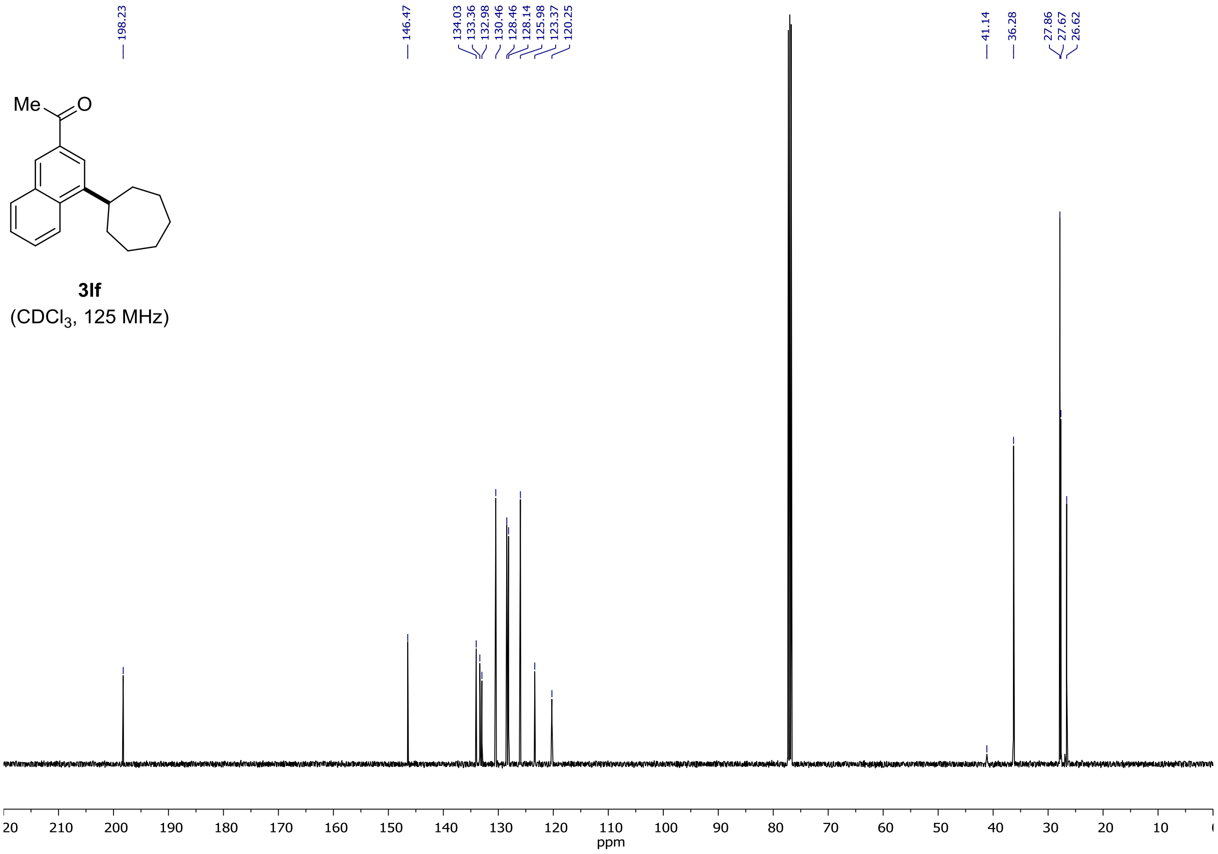

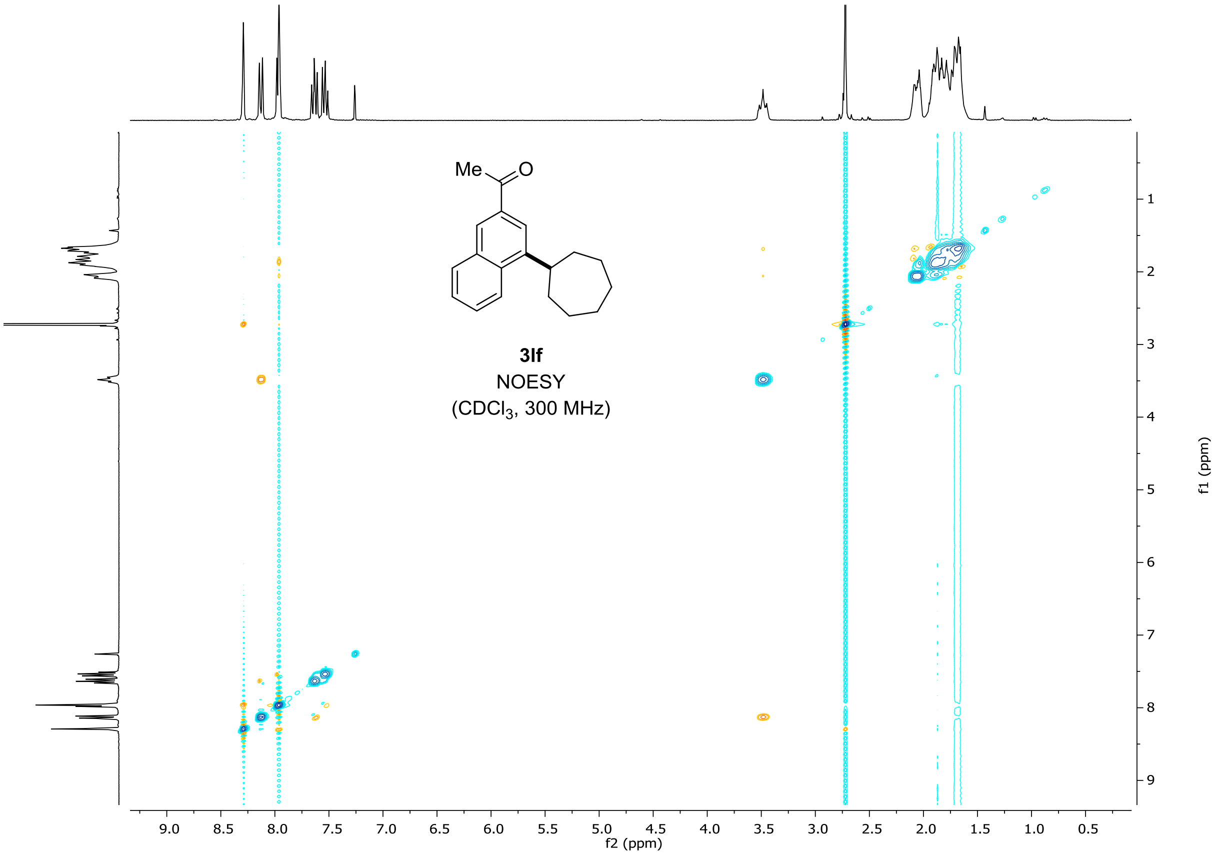

Supplementary Figure 30: ^{1}H, ^{13}C and NOESY-NMR of Compound 3lf.

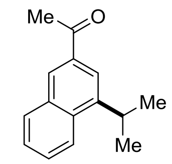

3lj
($CDCl_3$, 300 MHz)

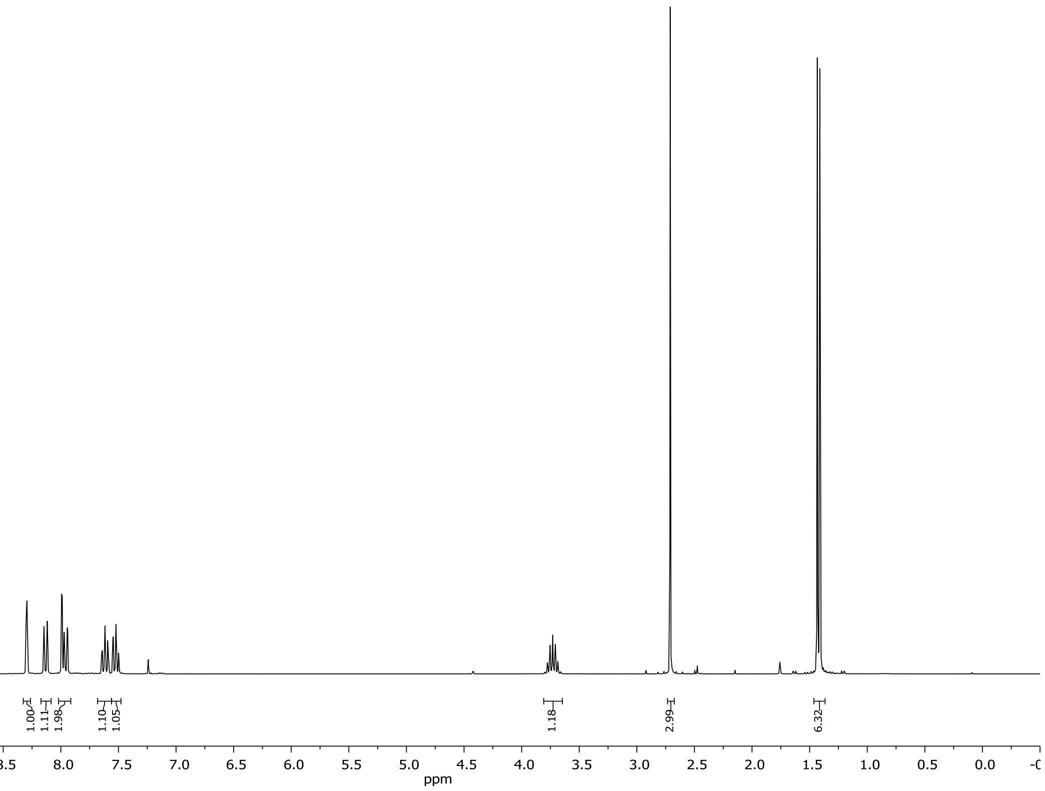

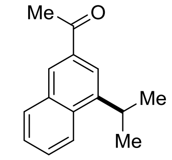

3lj
($CDCl_3$, 125 MHz)

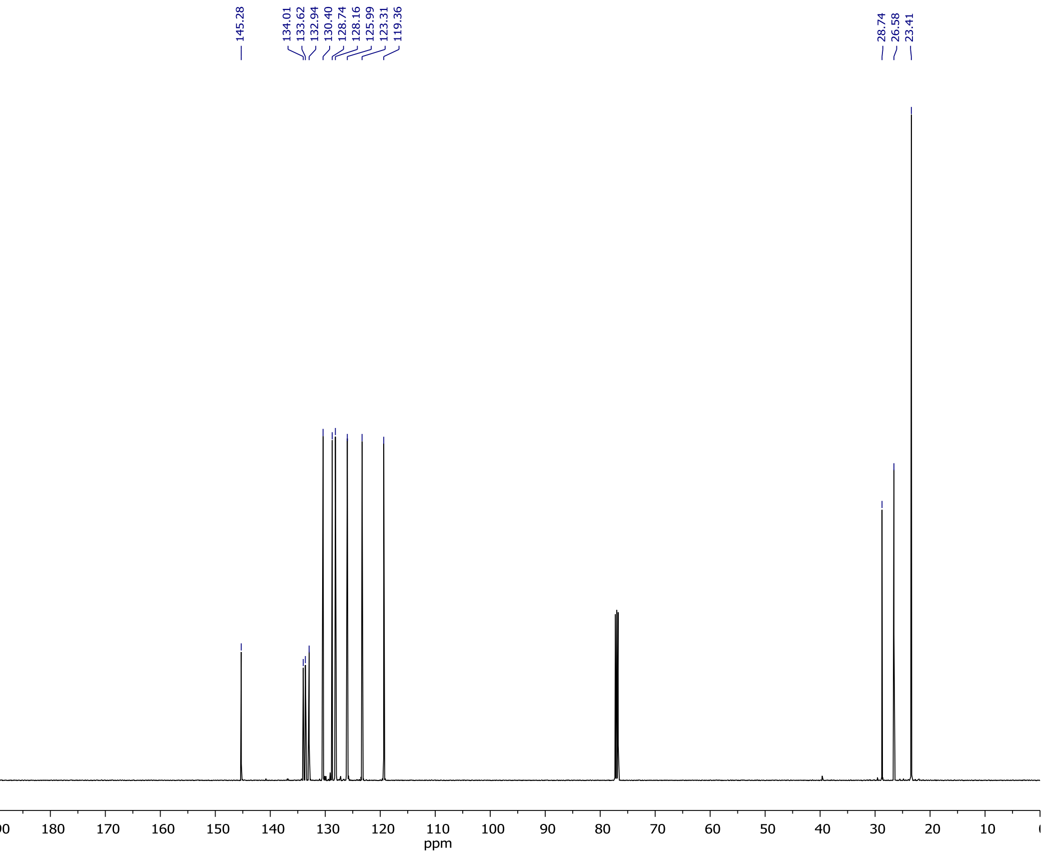

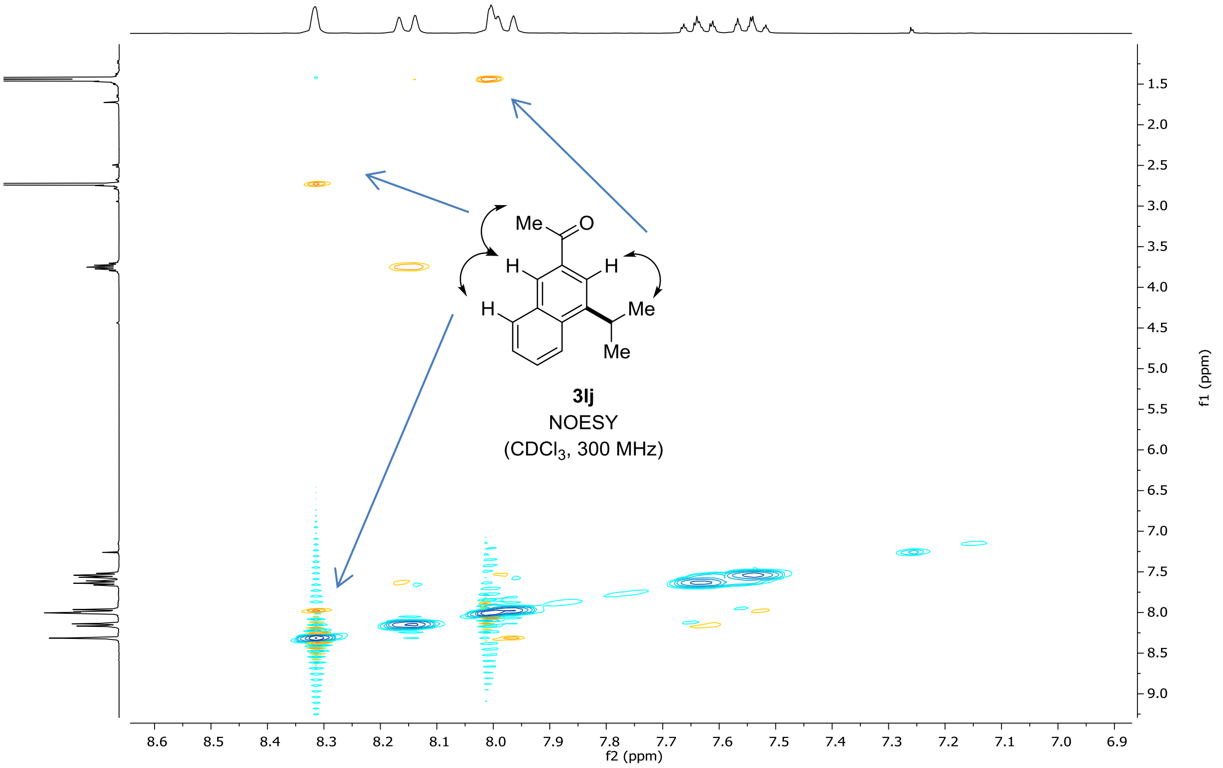

Supplementary Figure 31: ^{1}H, ^{13}C and NOESY-NMR of Compound 3lj.

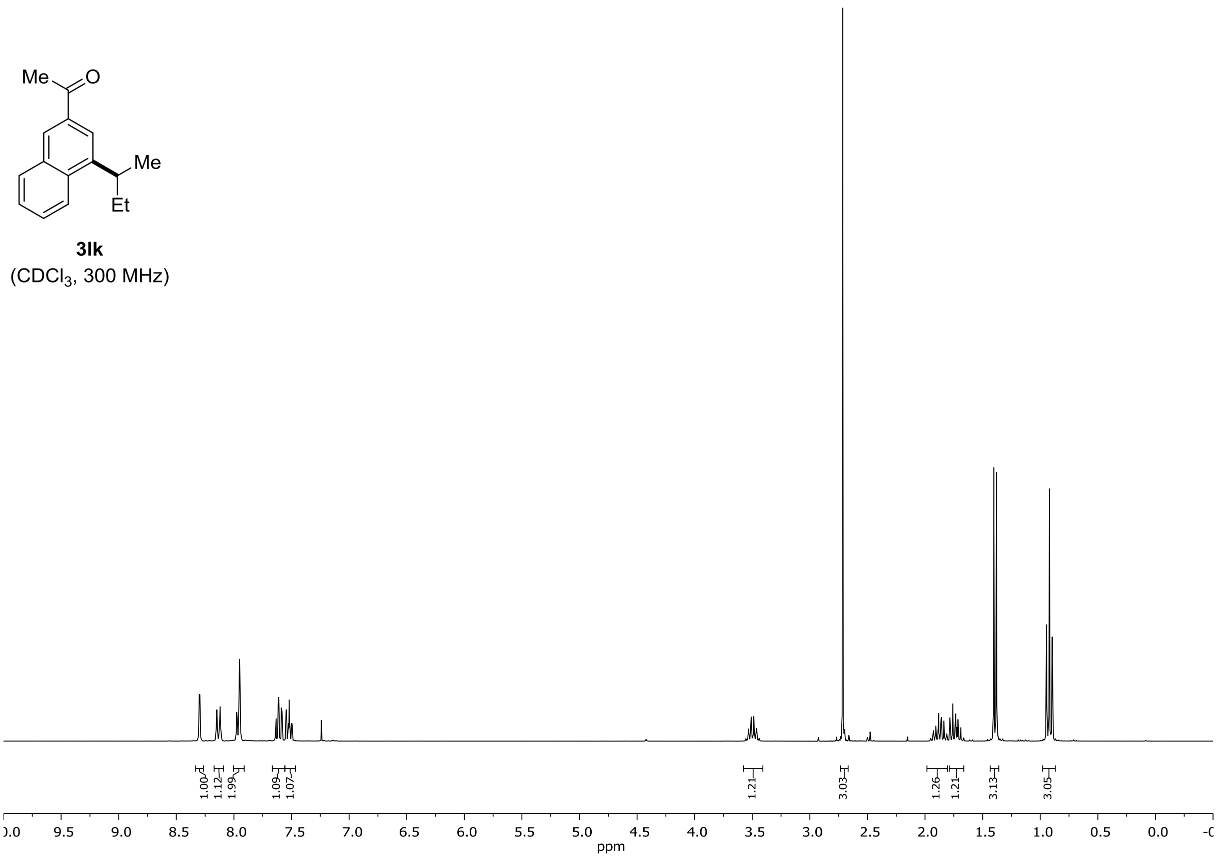

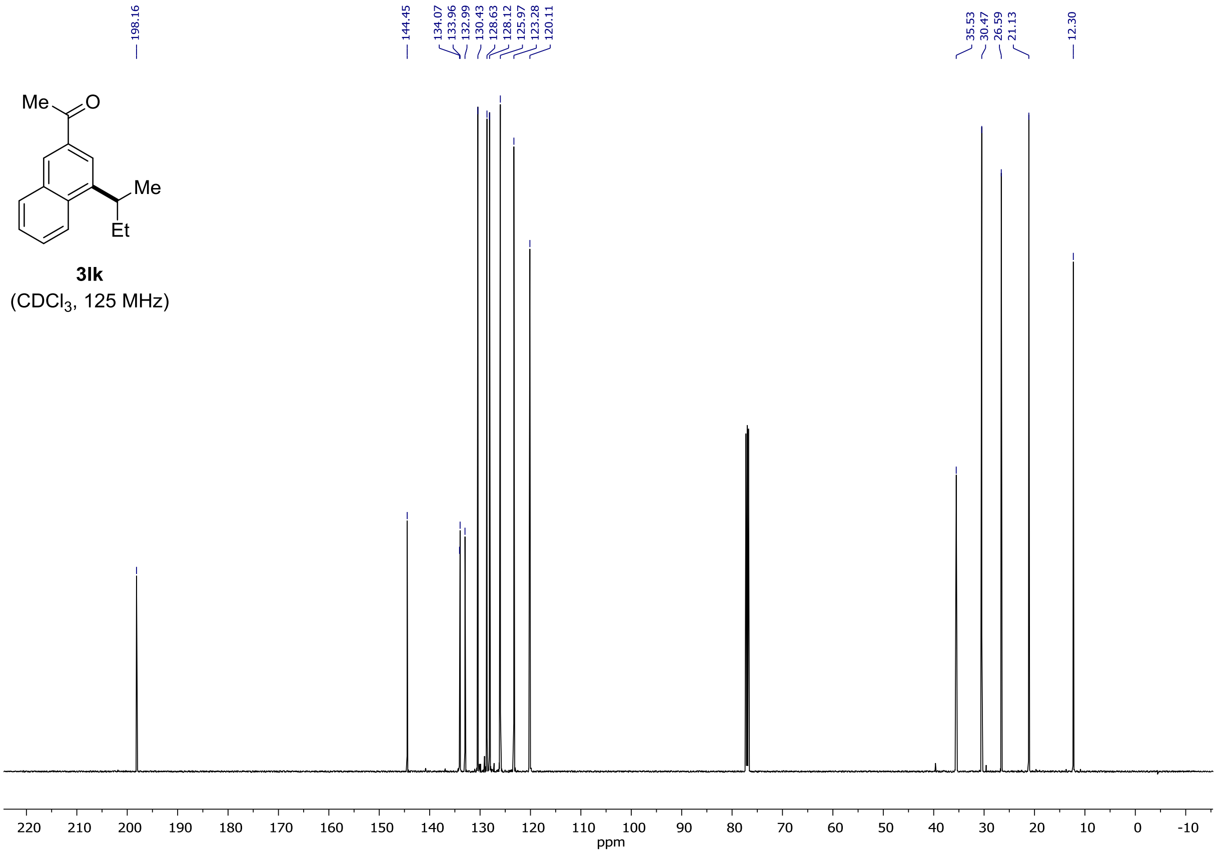

Supplementary Figure 32: ^{1}H and ^{13}C-NMR of Compound 3lk.

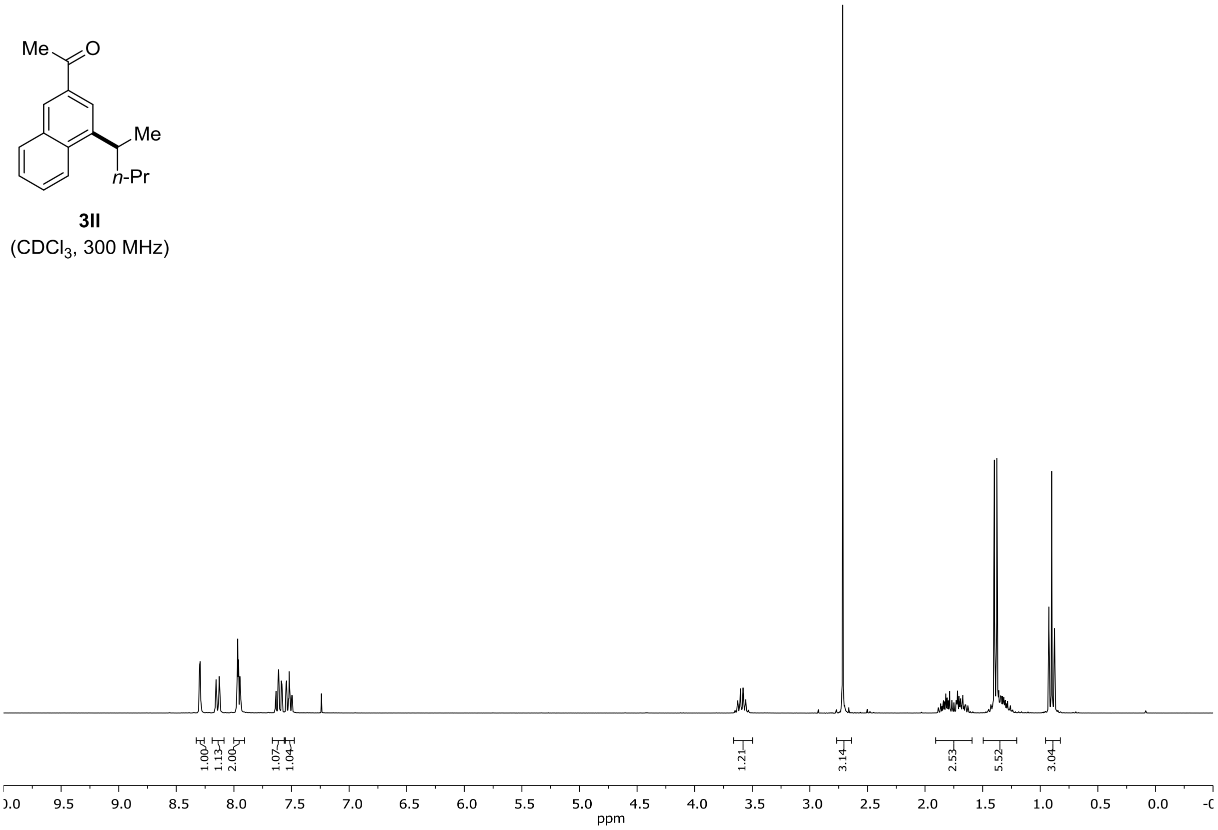

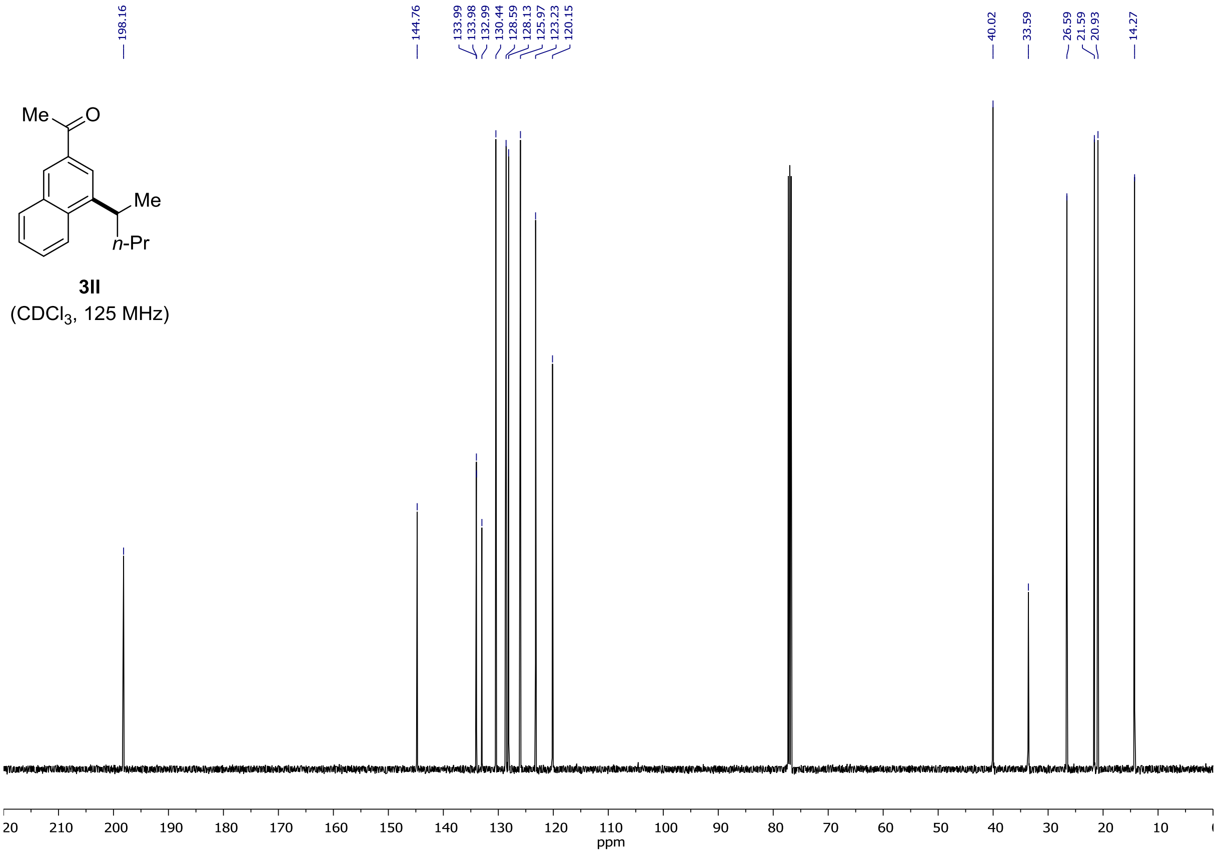

Supplementary Figure 33: ^{1}H and ^{13}C-NMR of Compound 3ll.

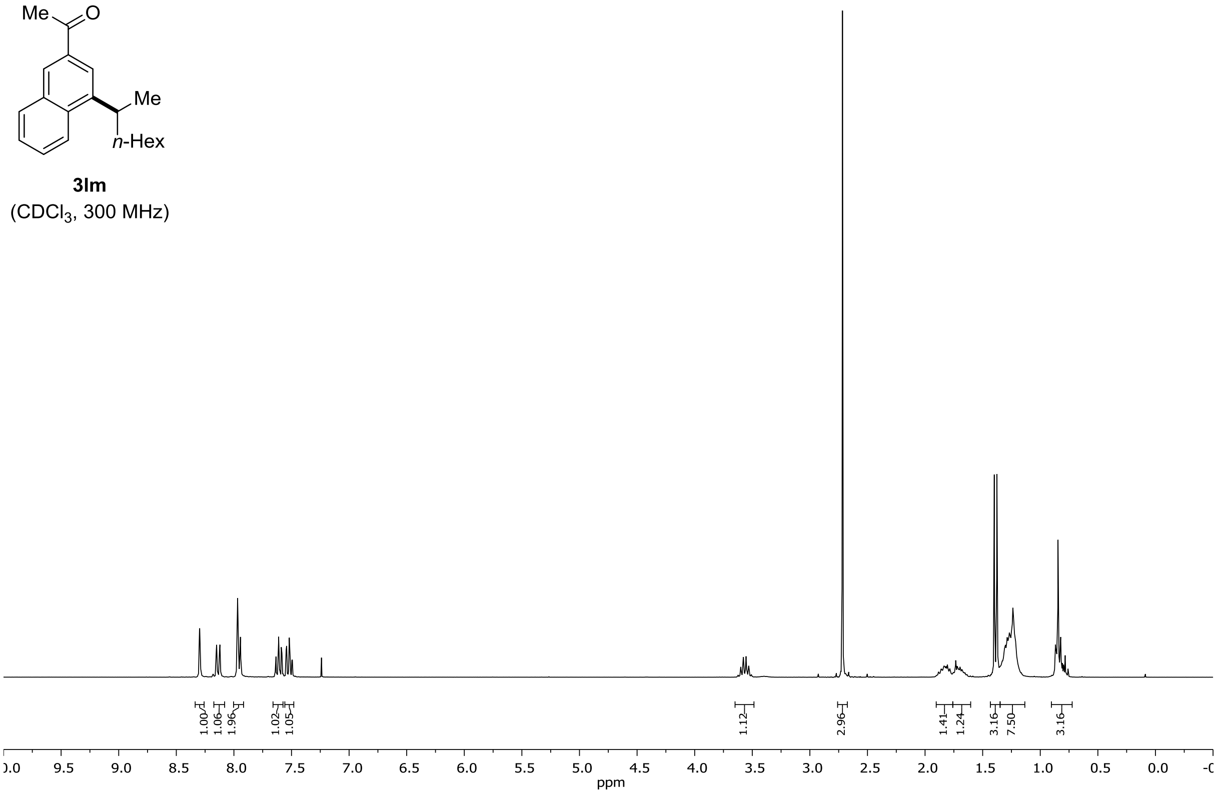

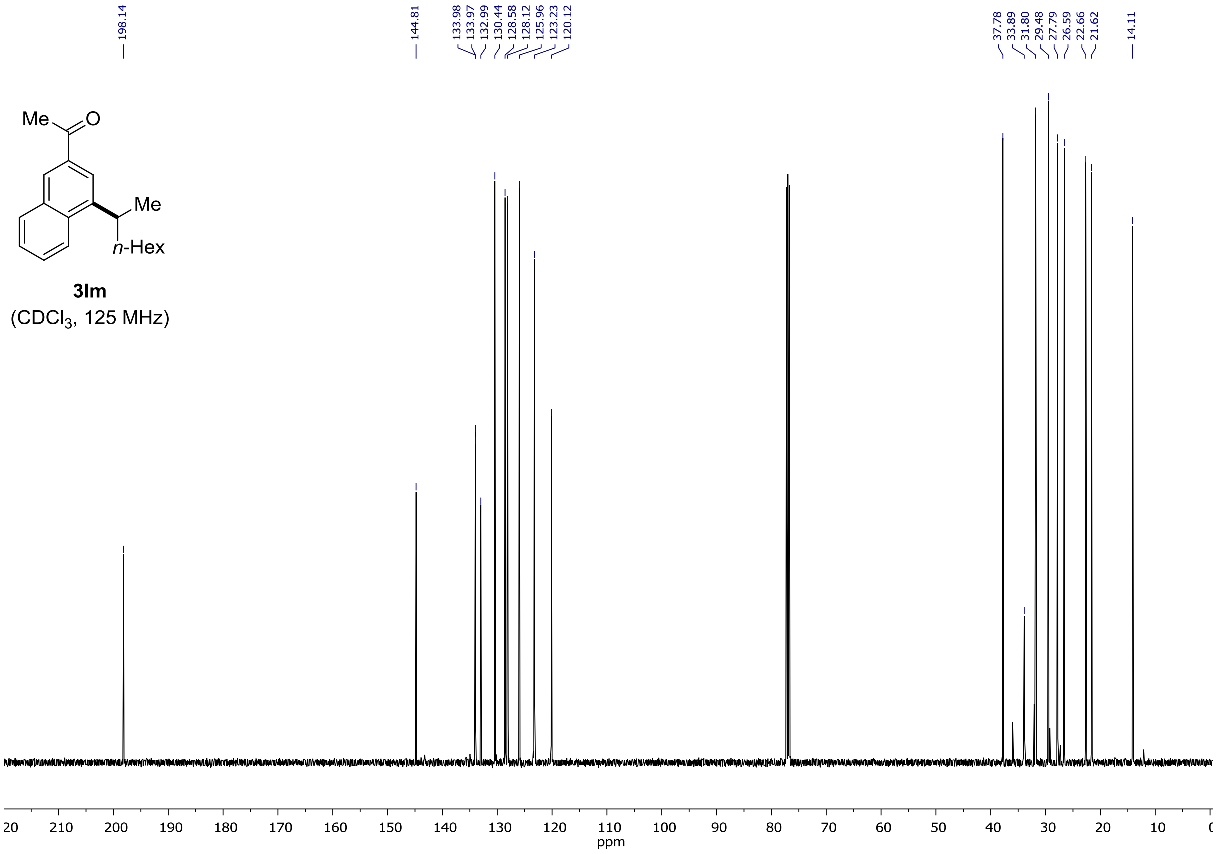

Supplementary Figure 34: ^{1}H and ^{13}C-NMR of Compound 3lm.

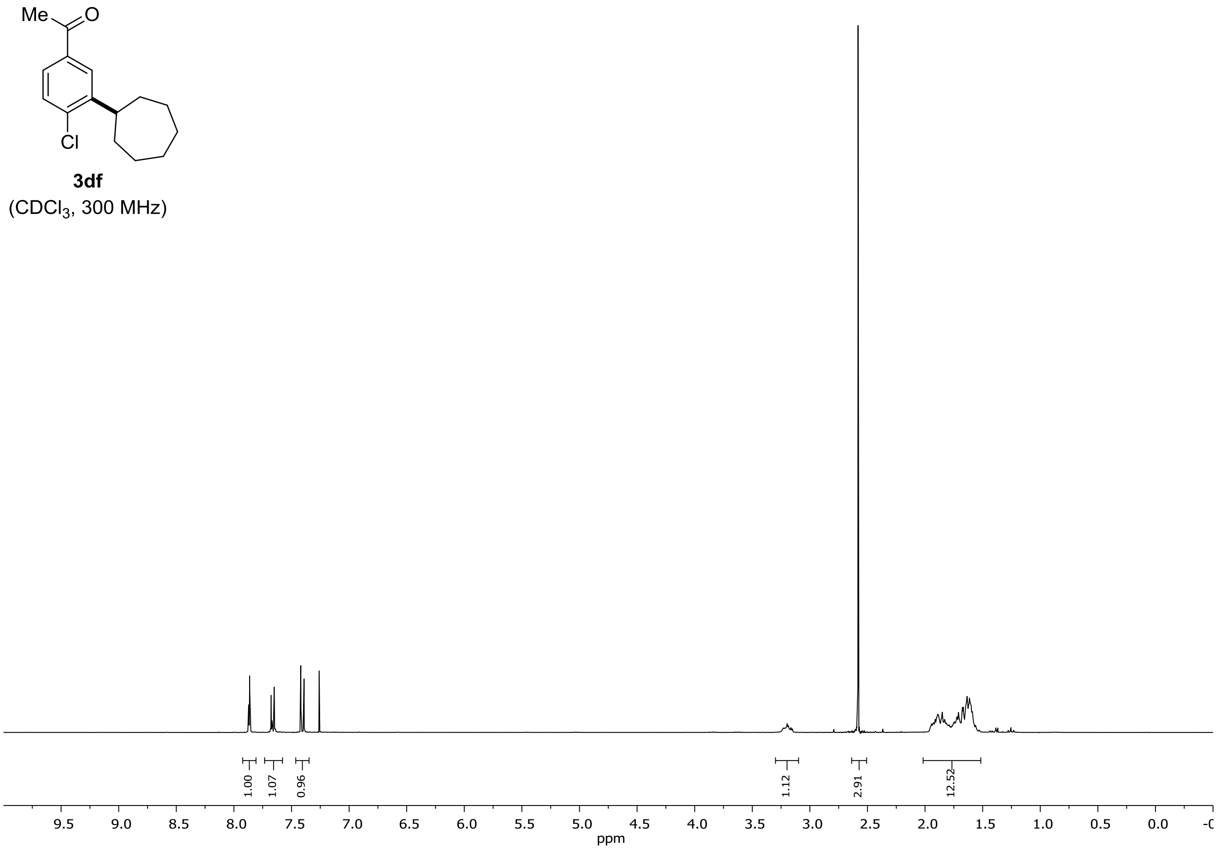

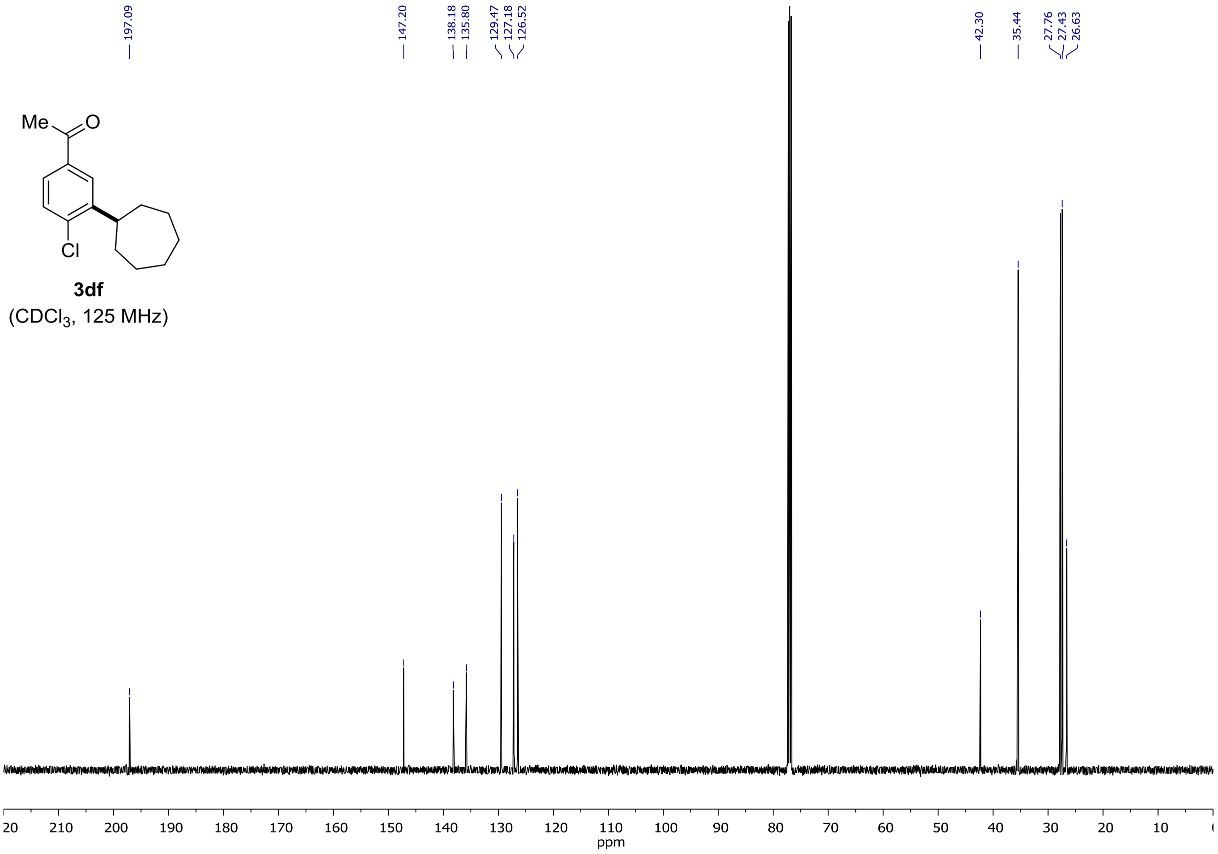

Supplementary Figure 35: ^{1}H and ^{13}C-NMR of Compound 3df.

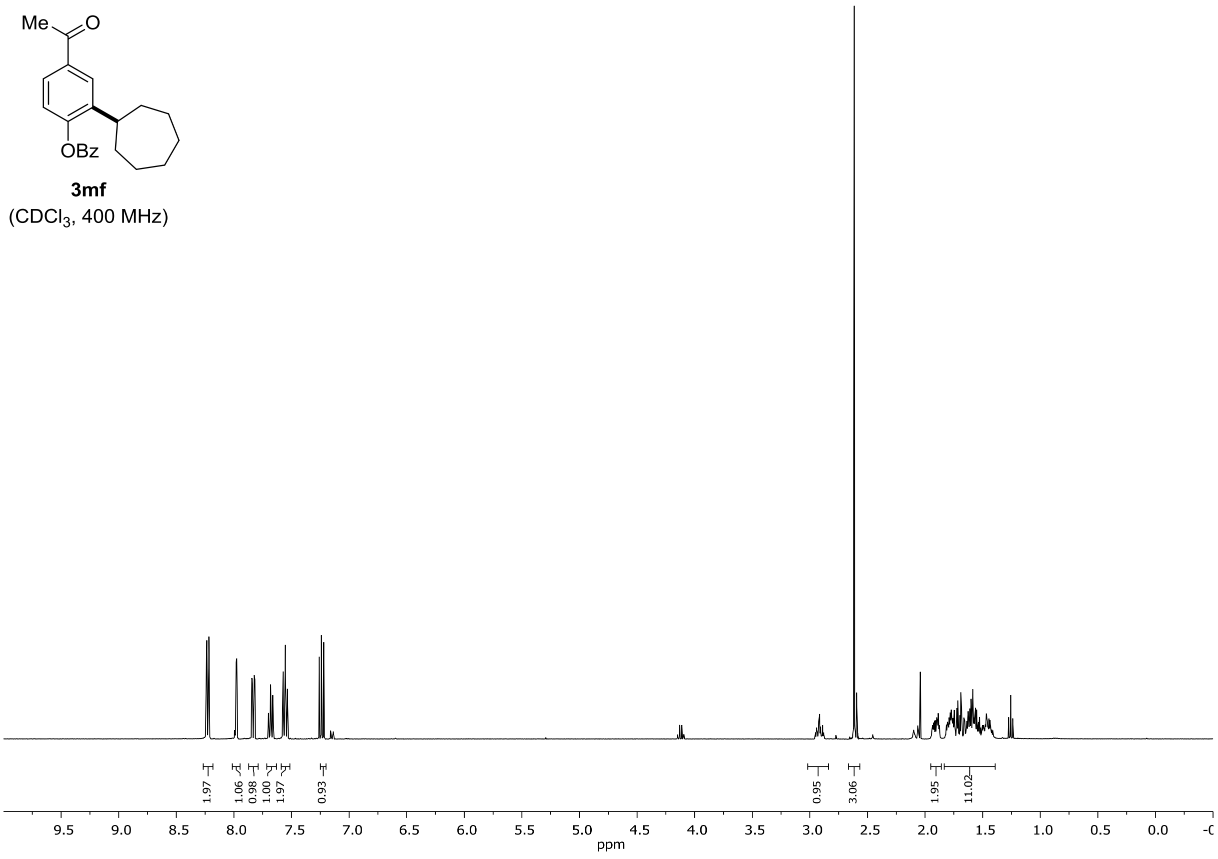

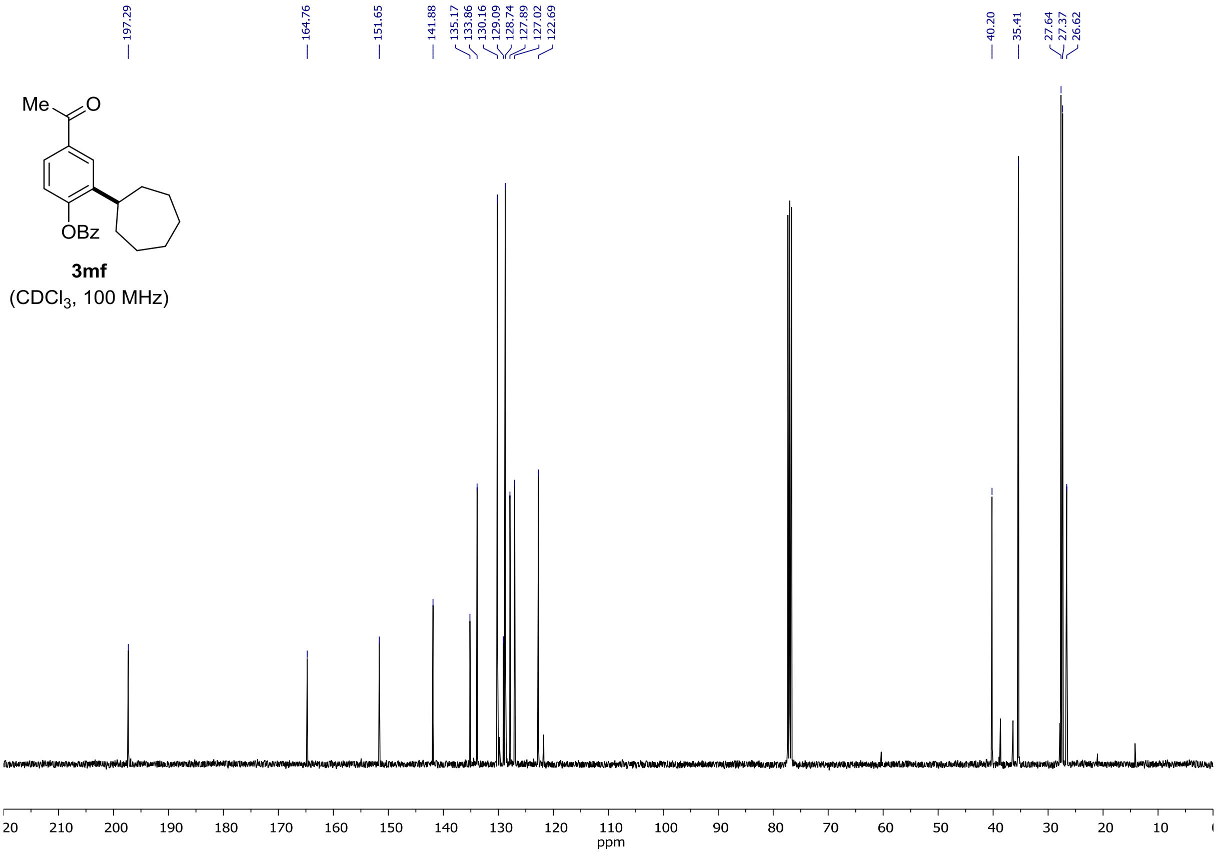

Supplementary Figure 36: ^{1}H and ^{13}C-NMR of Compound 3mf.

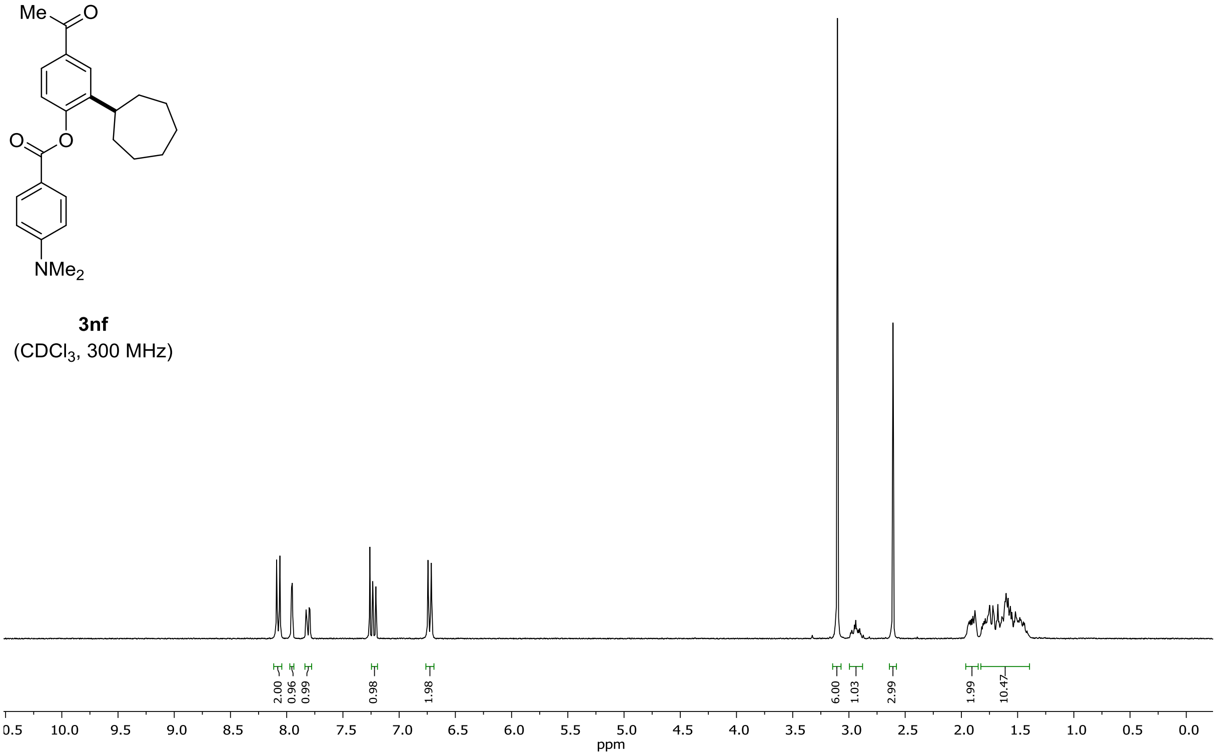

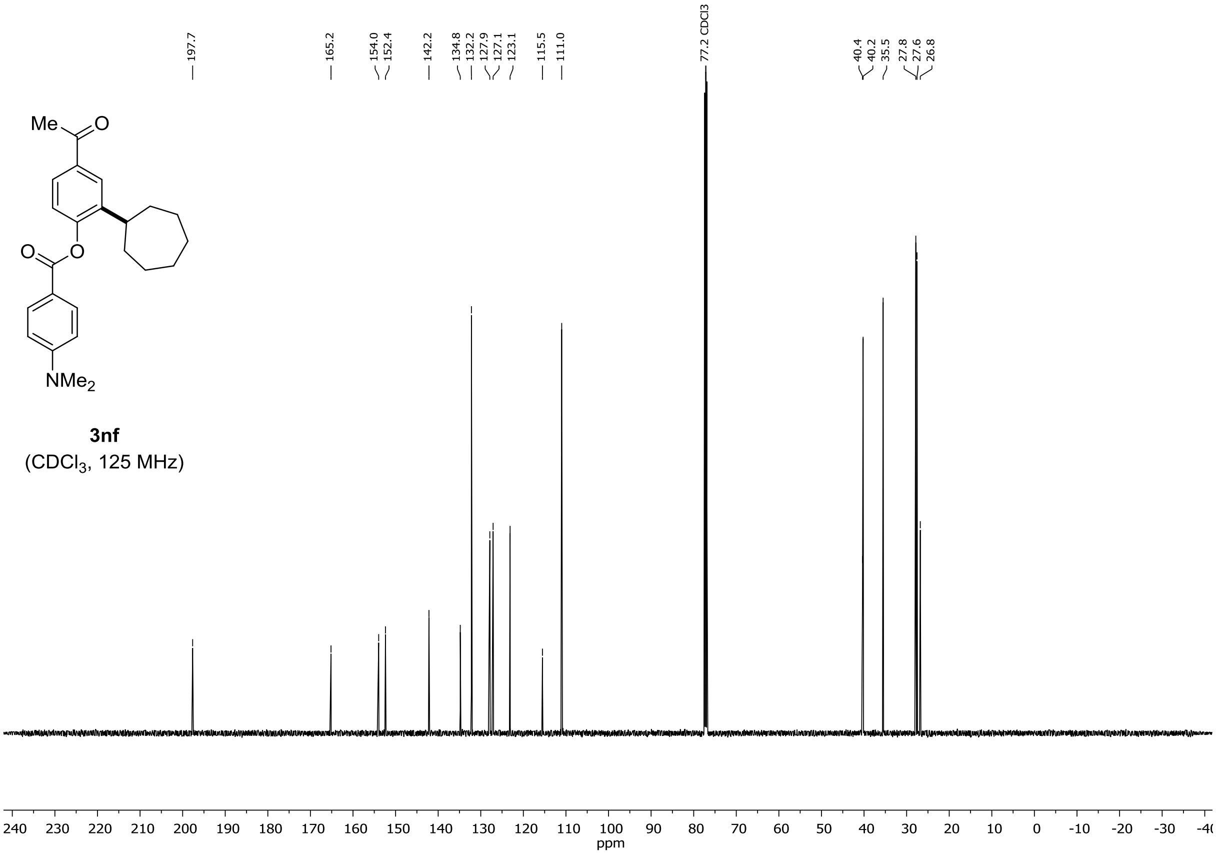

Supplementary Figure 37: ^{1}H and ^{13}C-NMR of Compound 3nf.

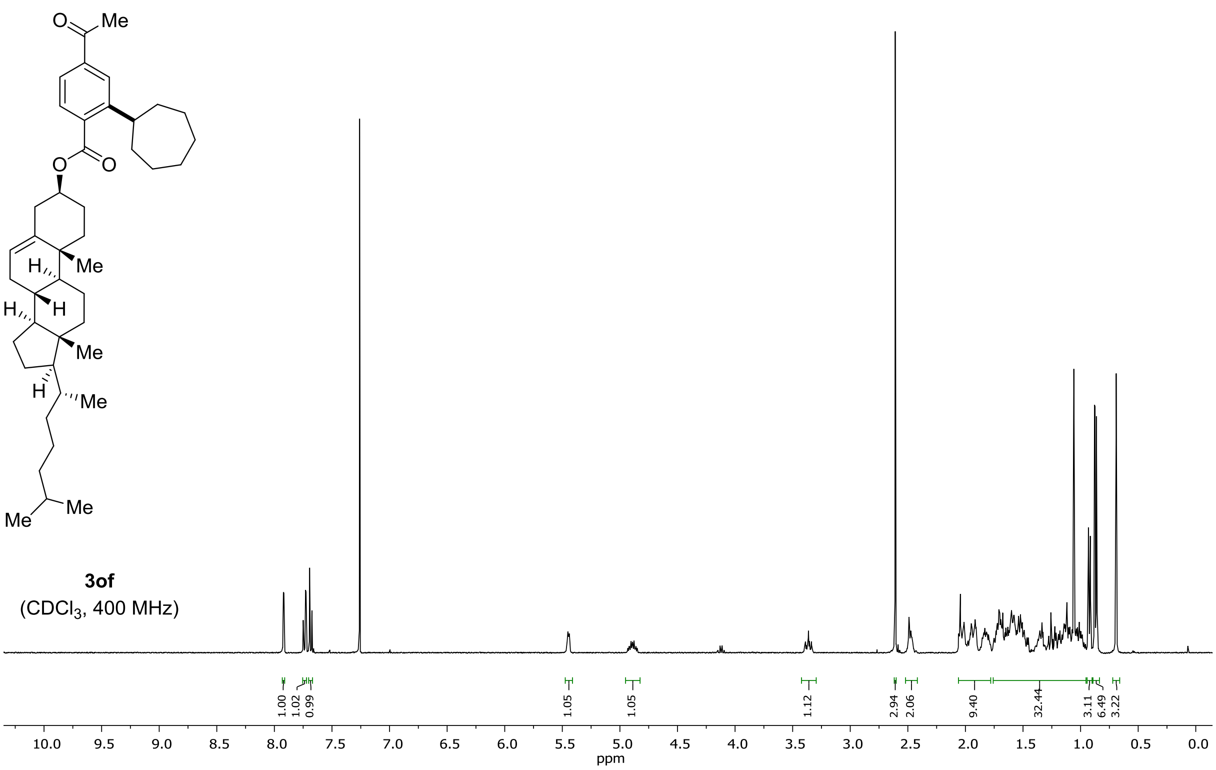

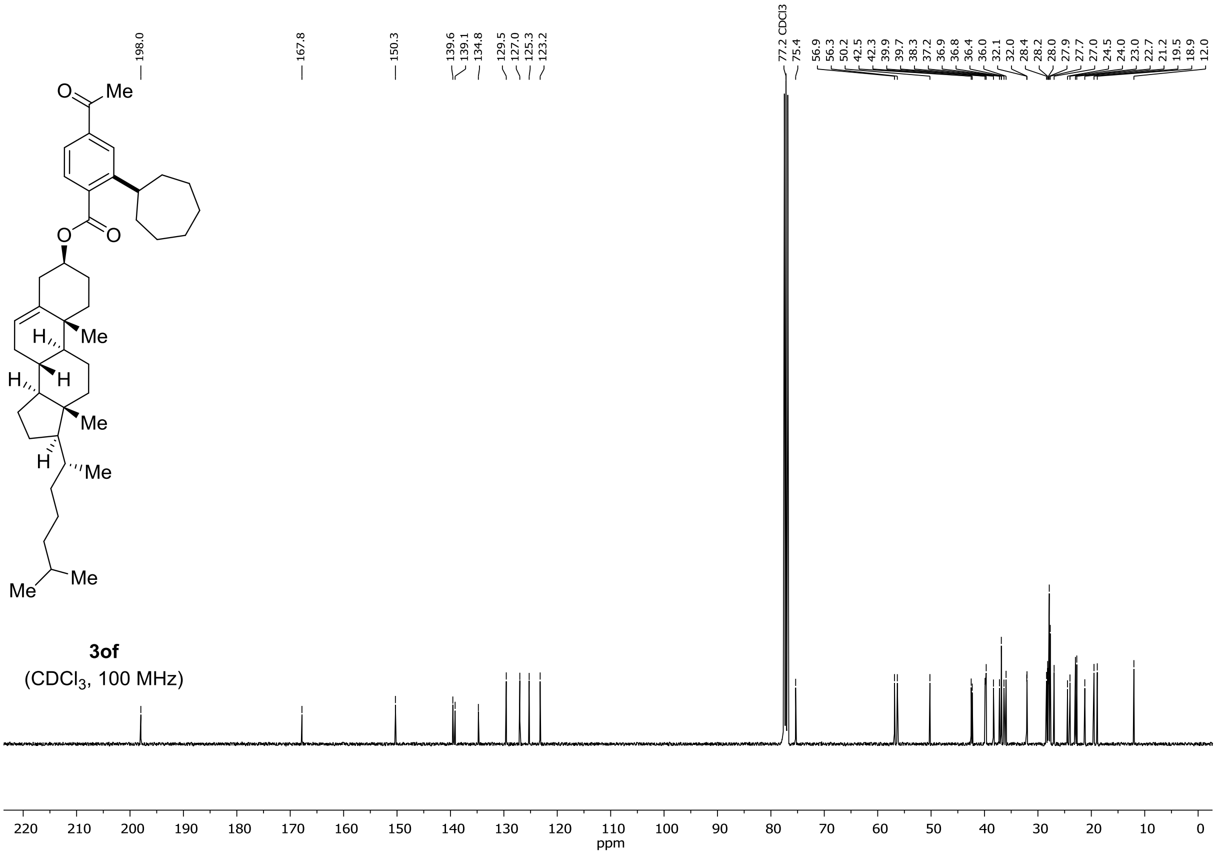

Supplementary Figure 38: ^{1}H and ^{13}C-NMR of Compound 3of.

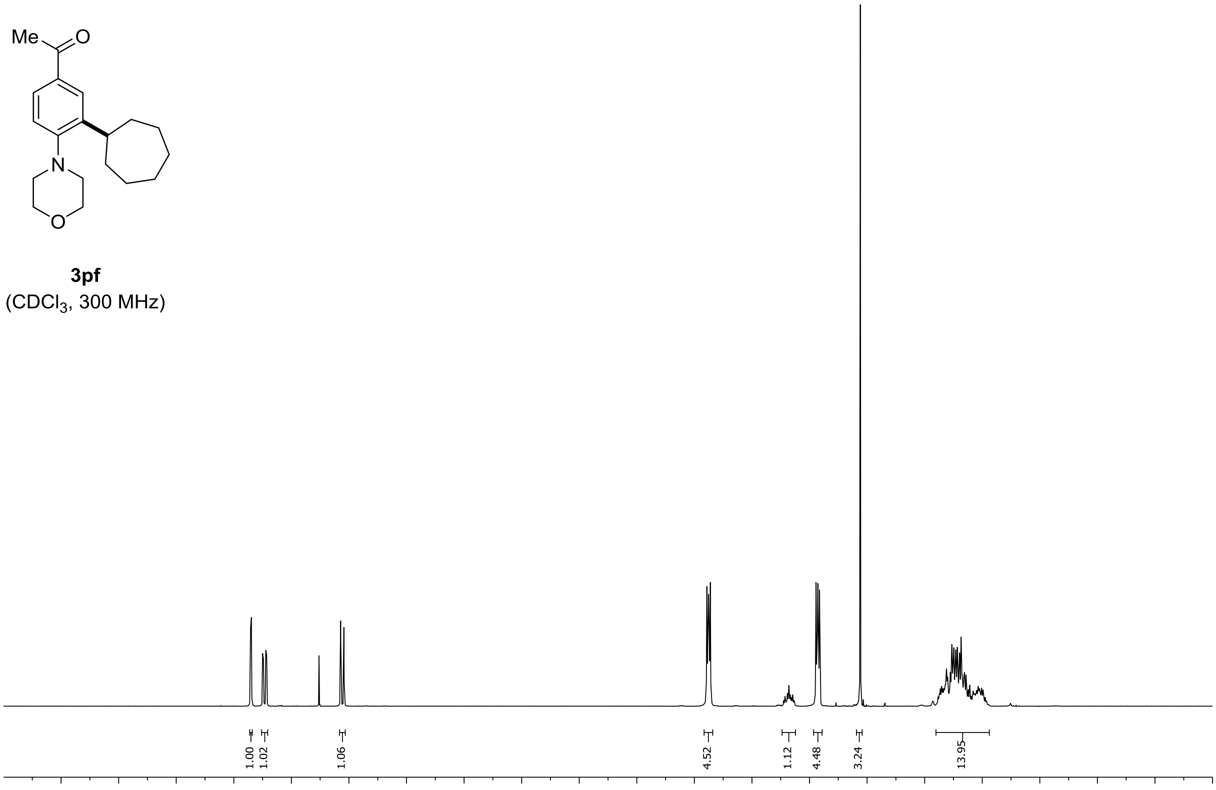

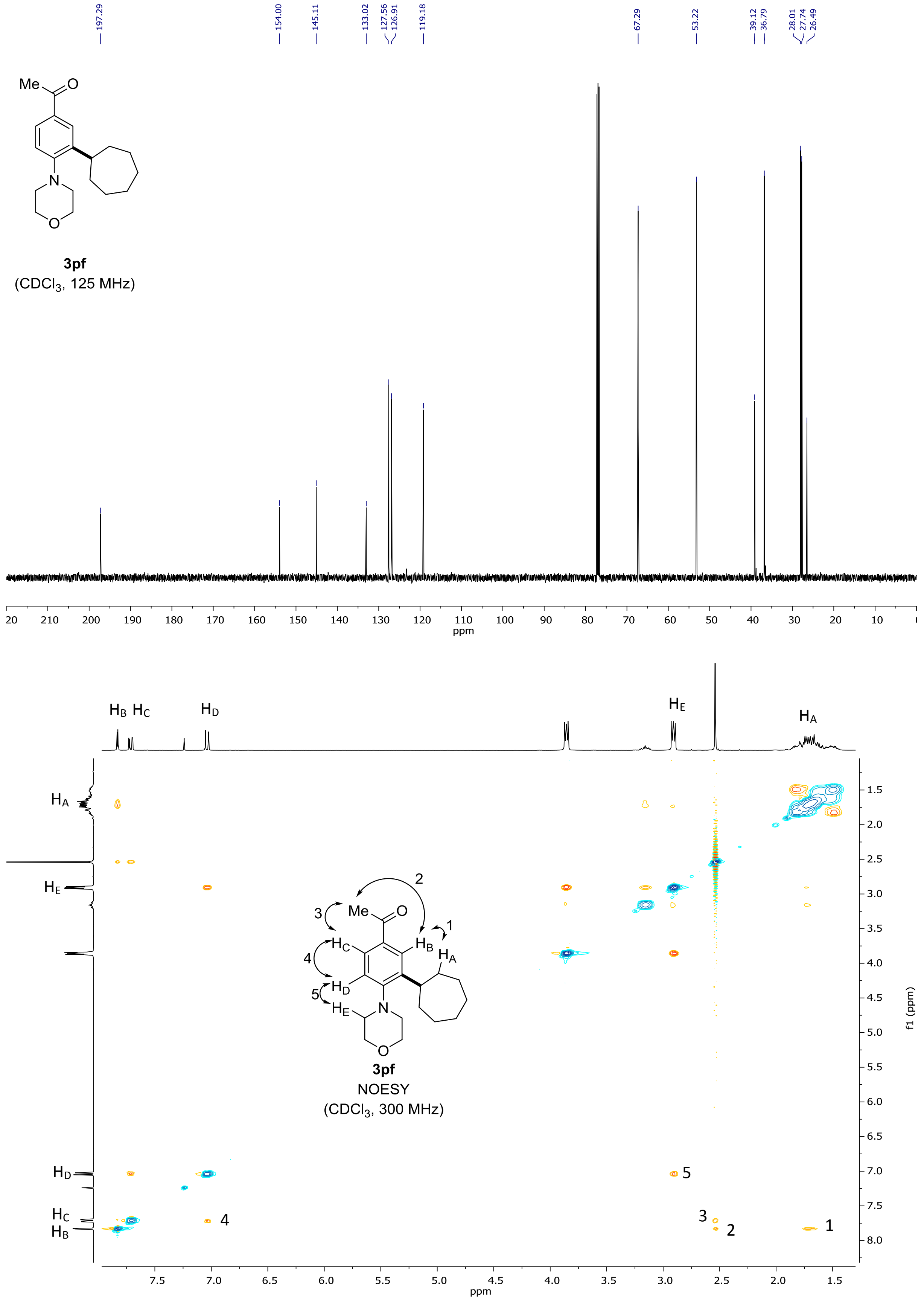

Supplementary Figure 39: ^{1}H, ^{13}C and NOESY-NMR of Compound 3pf.

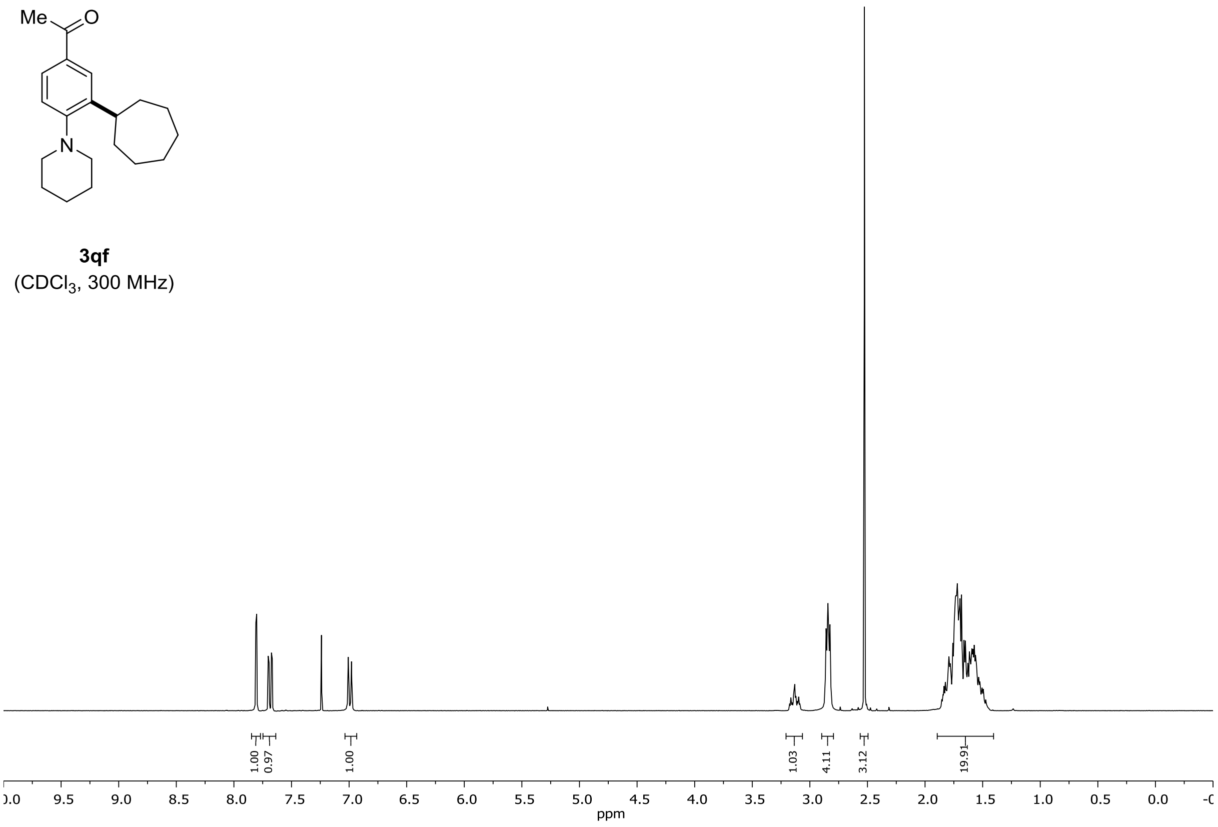

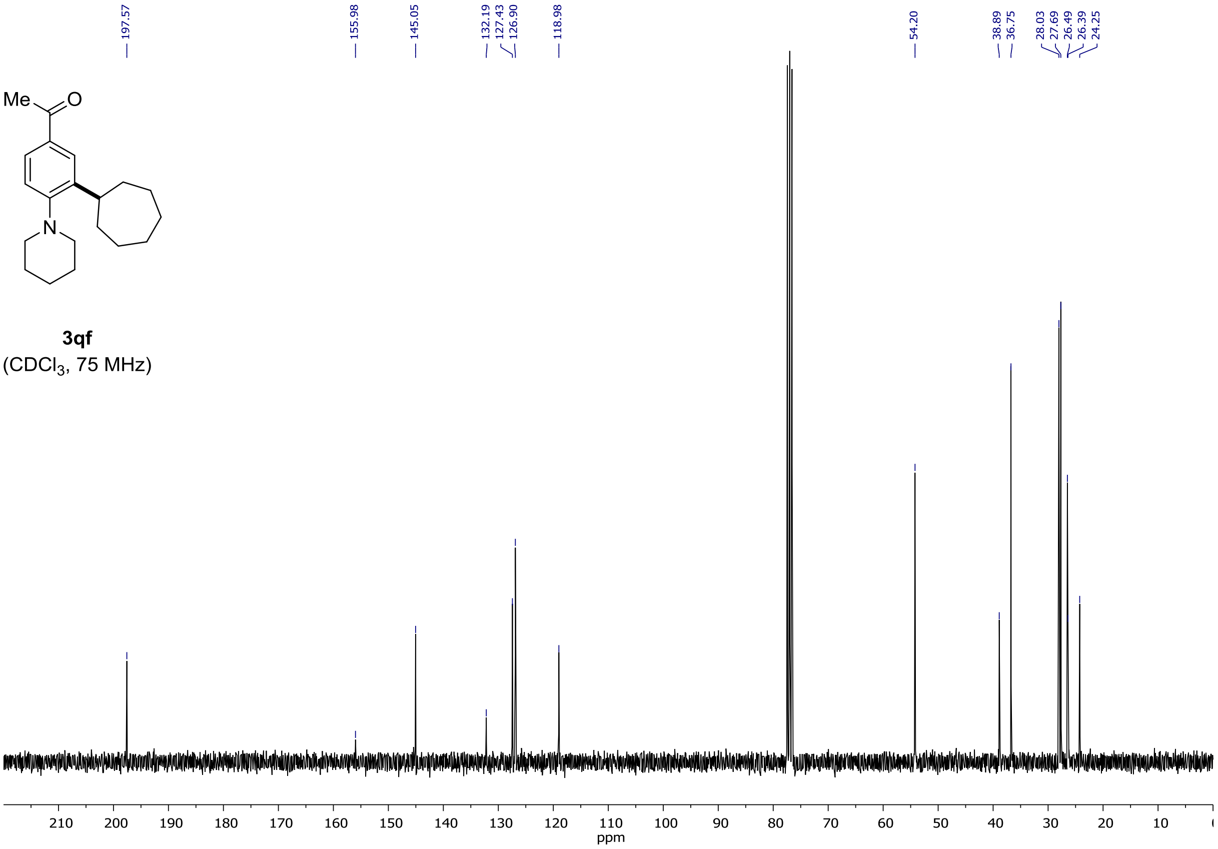

Supplementary Figure 40: ^{1}H and ^{13}C-NMR of Compound 3qf.

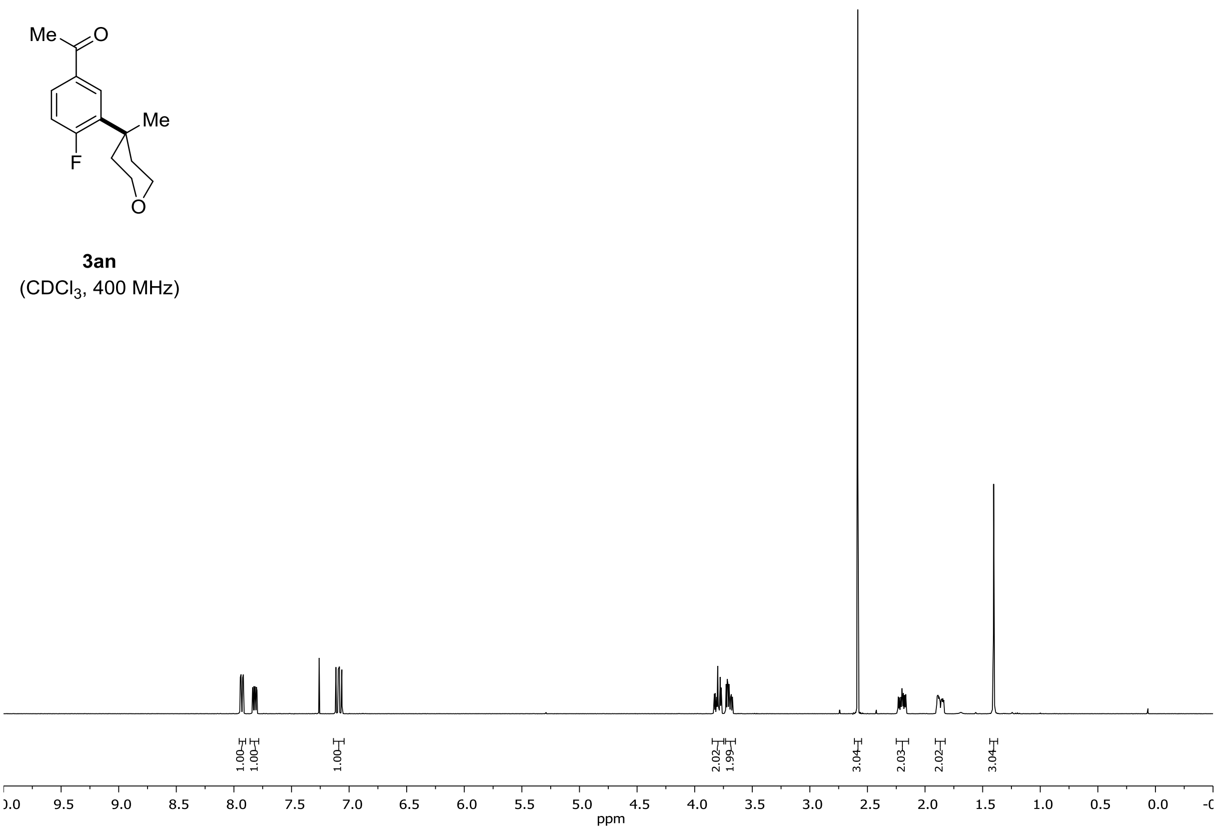
3an
(CDCl$_3$, 400 MHz)
1.00
1.00
1.00
2.02
1.99
3.04
2.03
2.02
3.04
ppm

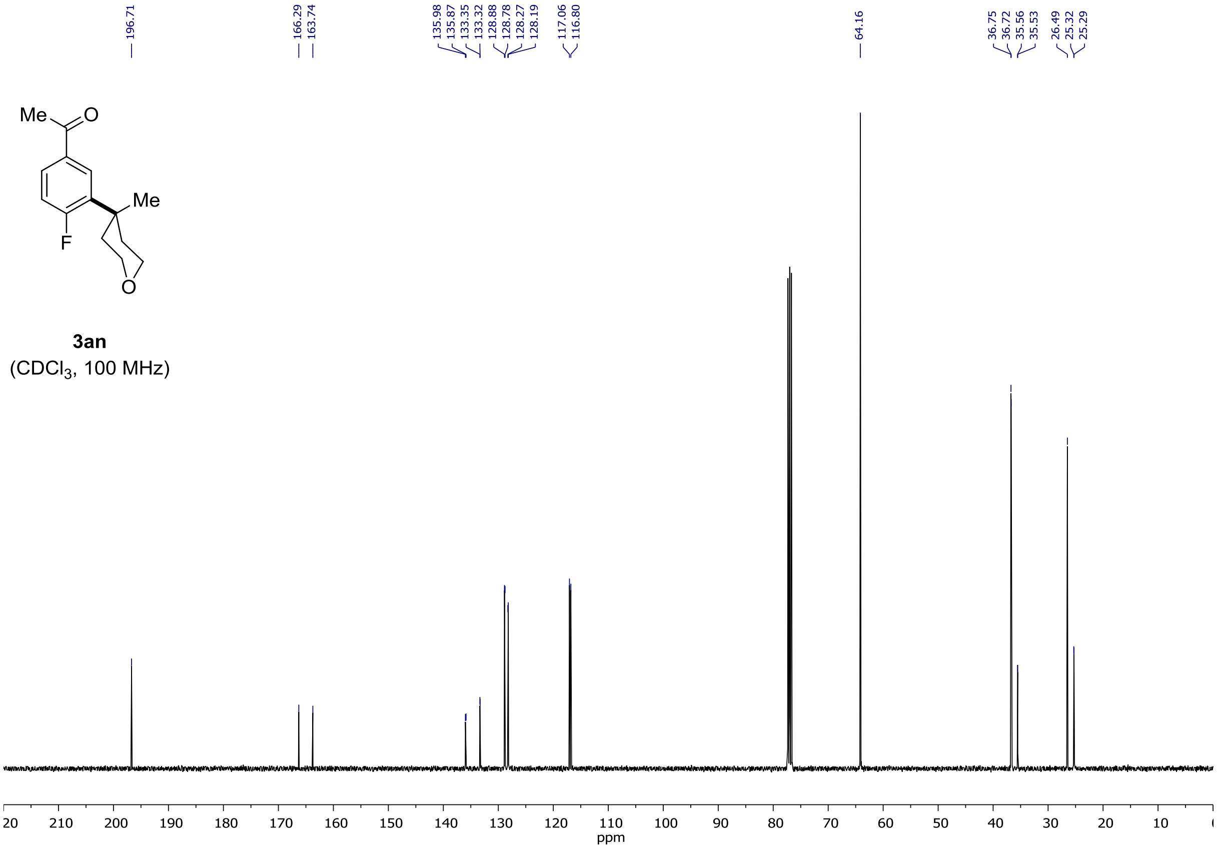
196.71
166.29
163.74
135.98
135.87
133.35
133.32
128.88
128.78
128.27
128.19
117.06
116.80
64.16
36.75
36.72
35.56
35.53
26.49
25.32
25.29
3an
(CDCl$_3$, 100 MHz)
ppm

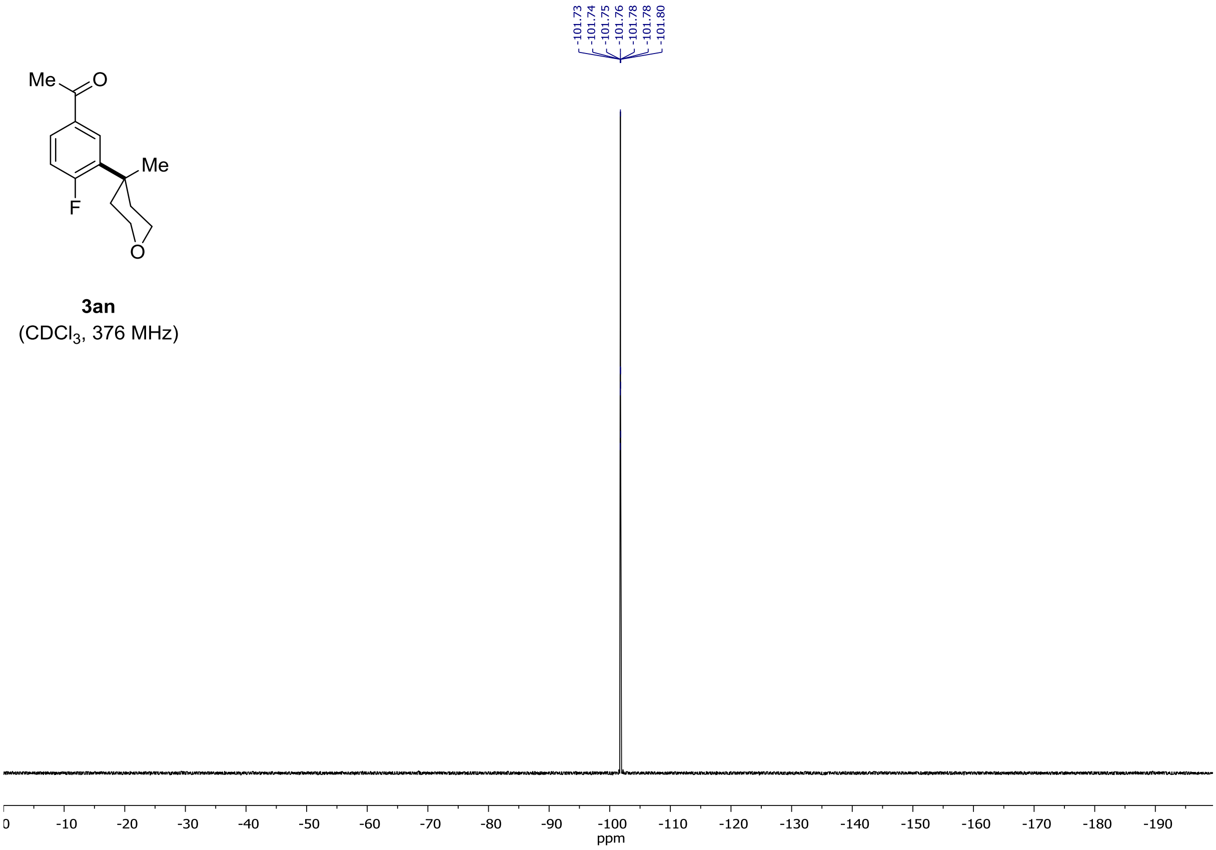

Supplementary Figure 41: ^{1}H, ^{13}C and ^{19}F-NMR of Compound 3an.

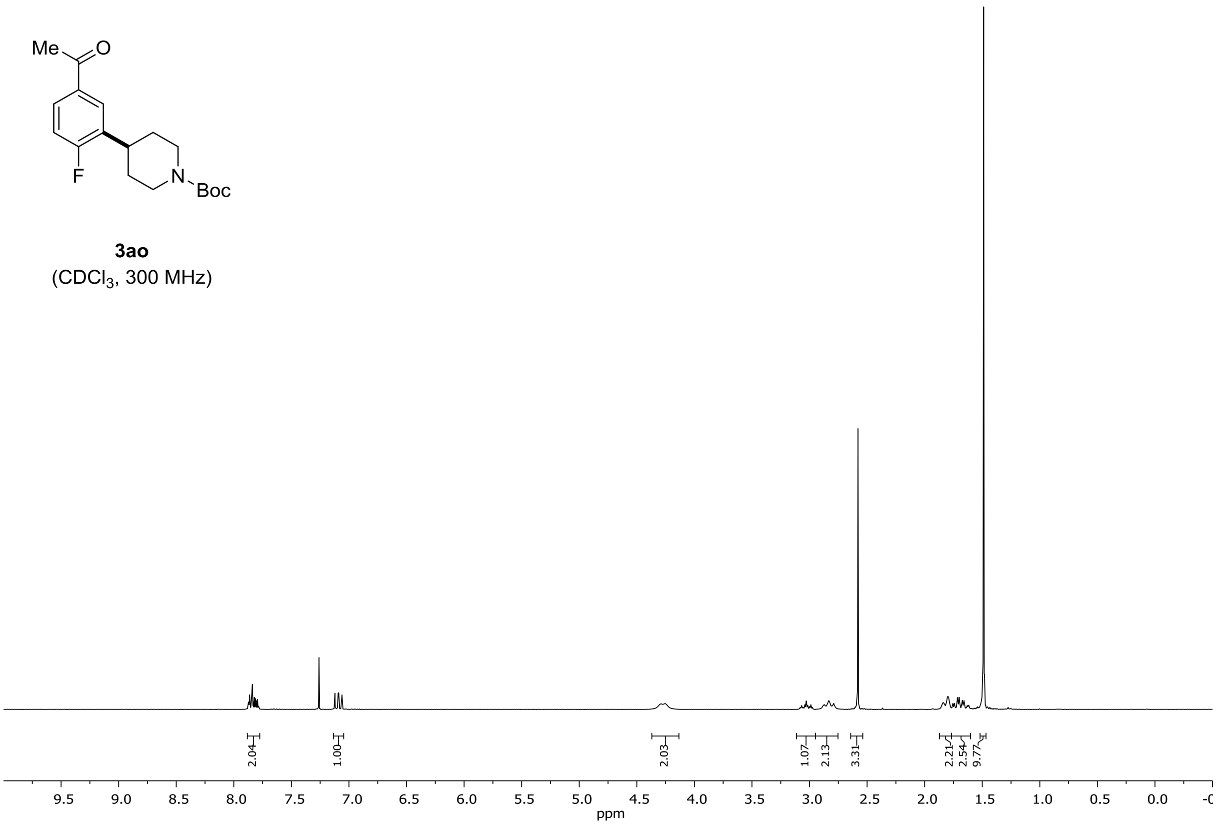

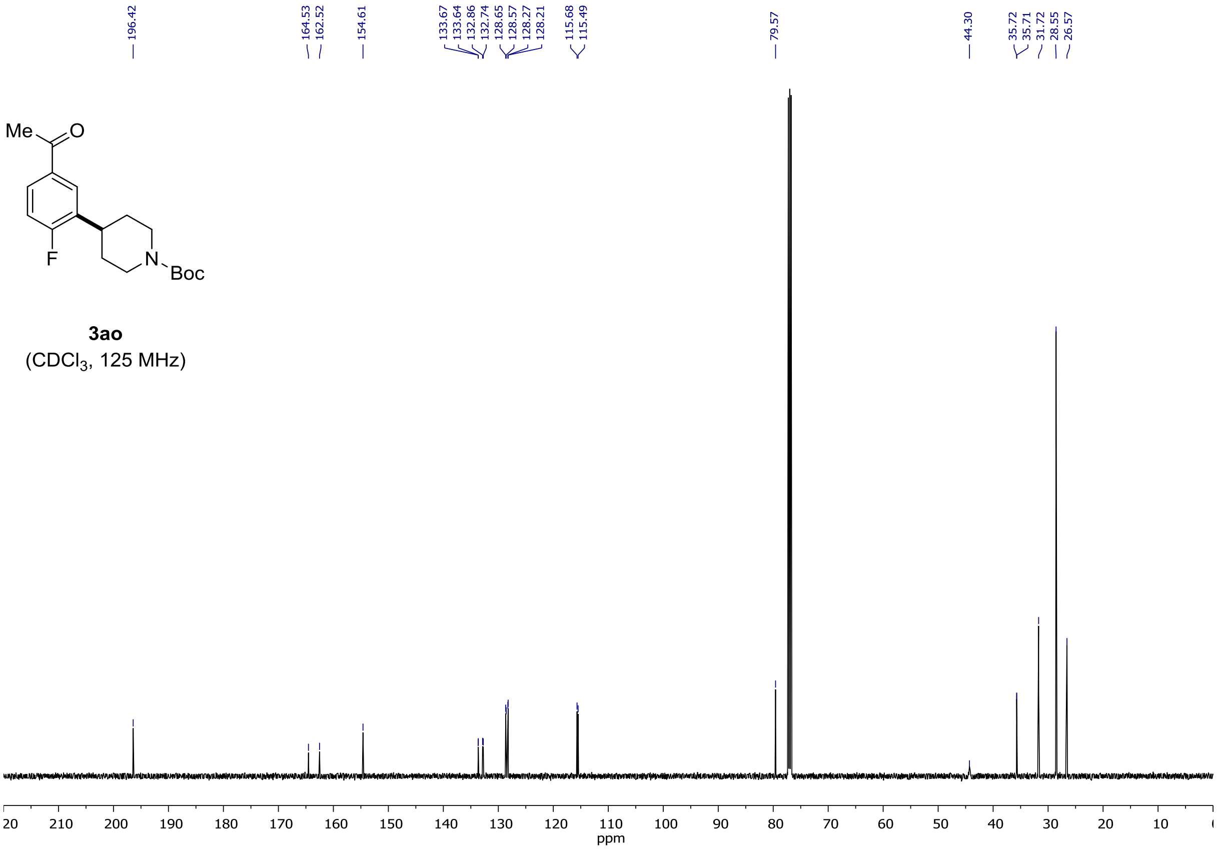

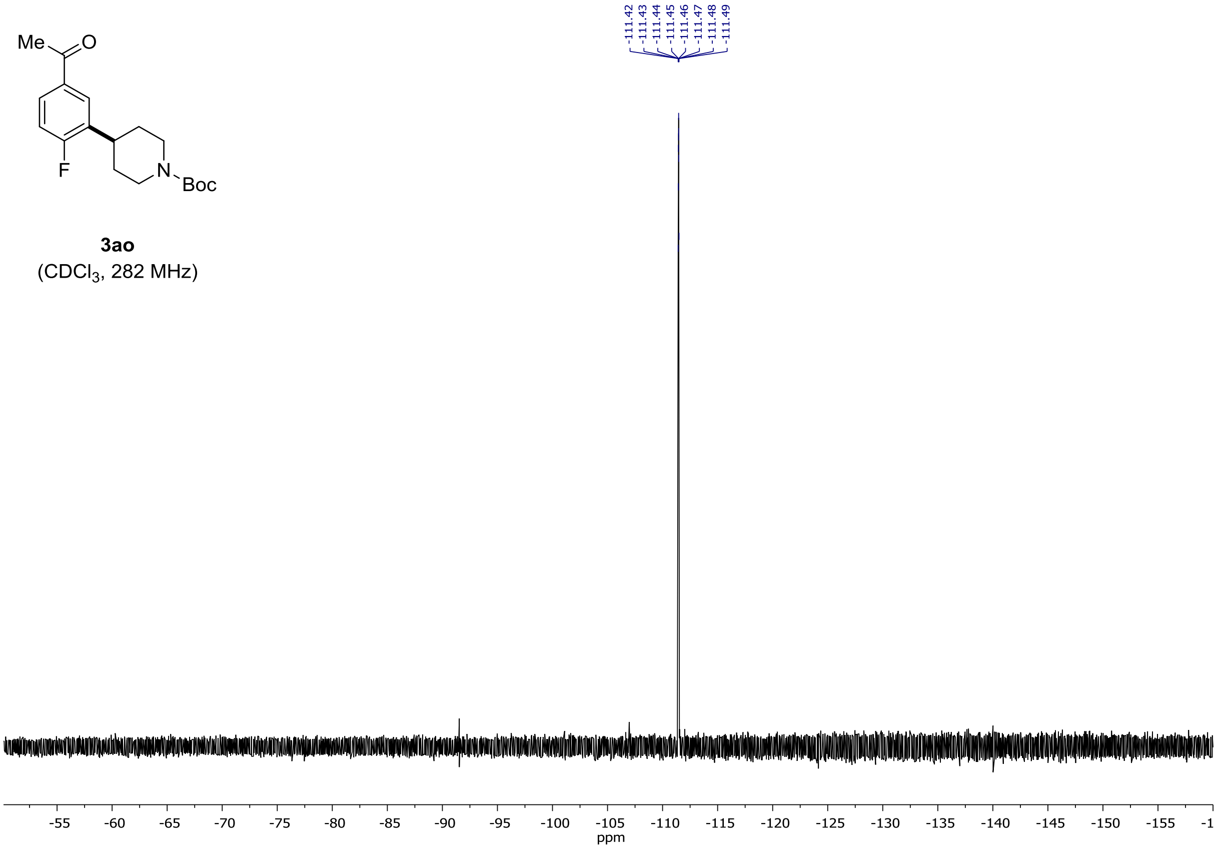

Supplementary Figure 42: ^{1}H, ^{13}C and ^{19}F-NMR of Compound 3ao.

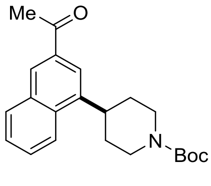

3lo
($CDCl_3$, 300 MHz)

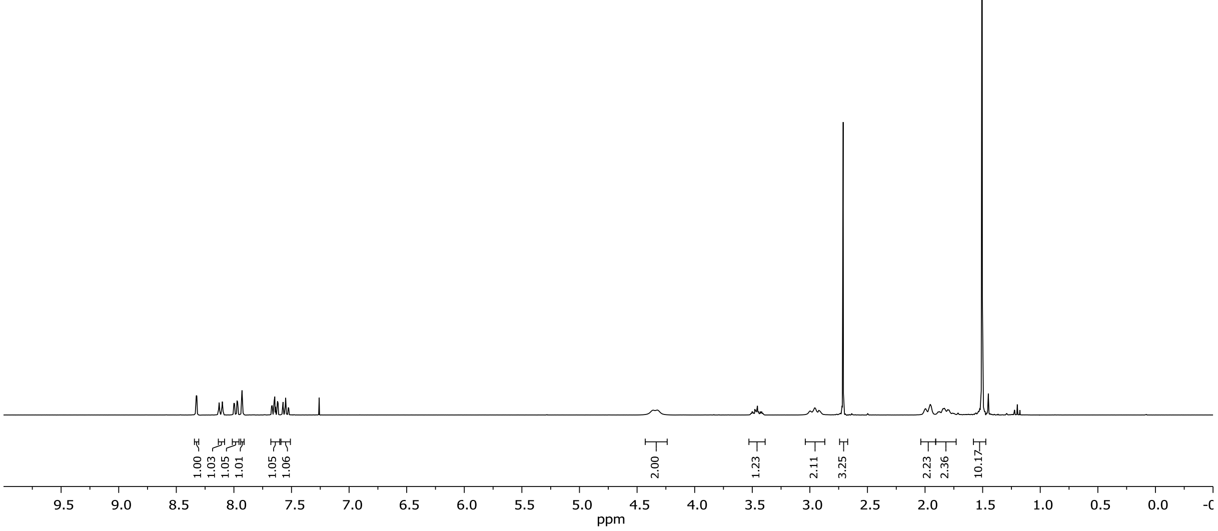

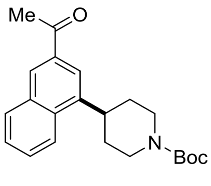

3lo
($CDCl_3$, 125 MHz)

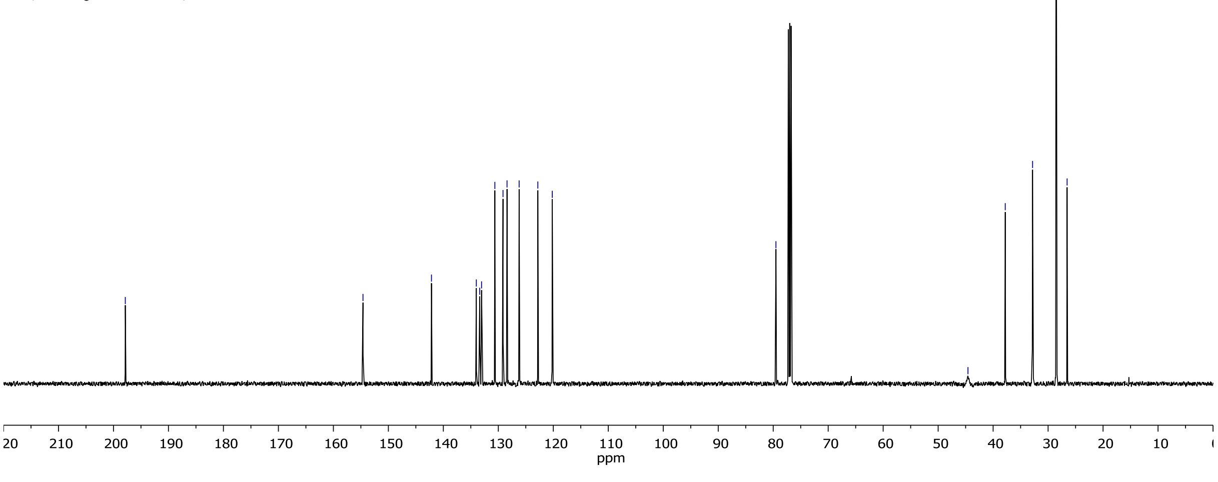

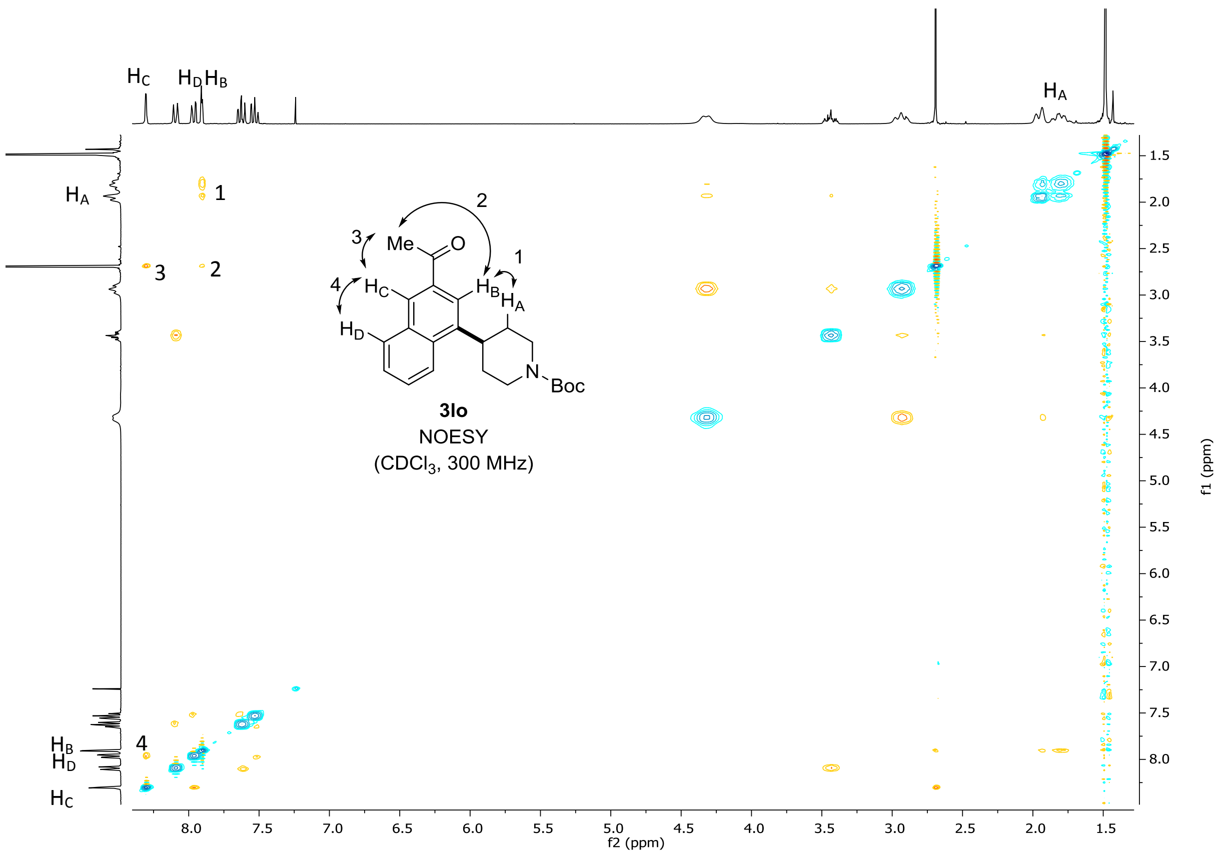

Supplementary Figure 43: ^{1}H, ^{13}C and NOESY-NMR of Compound 3lo.

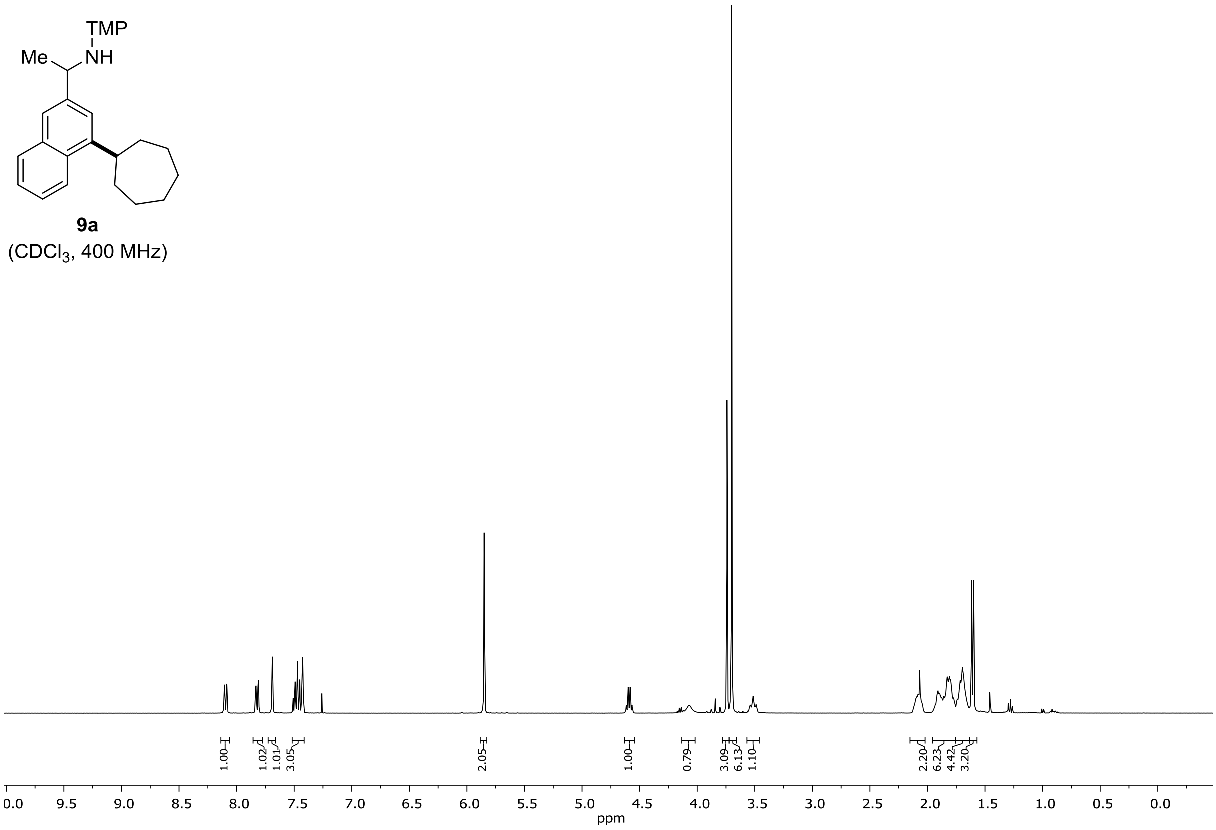

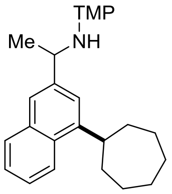

9a
($CDCl_3$, 100 MHz)

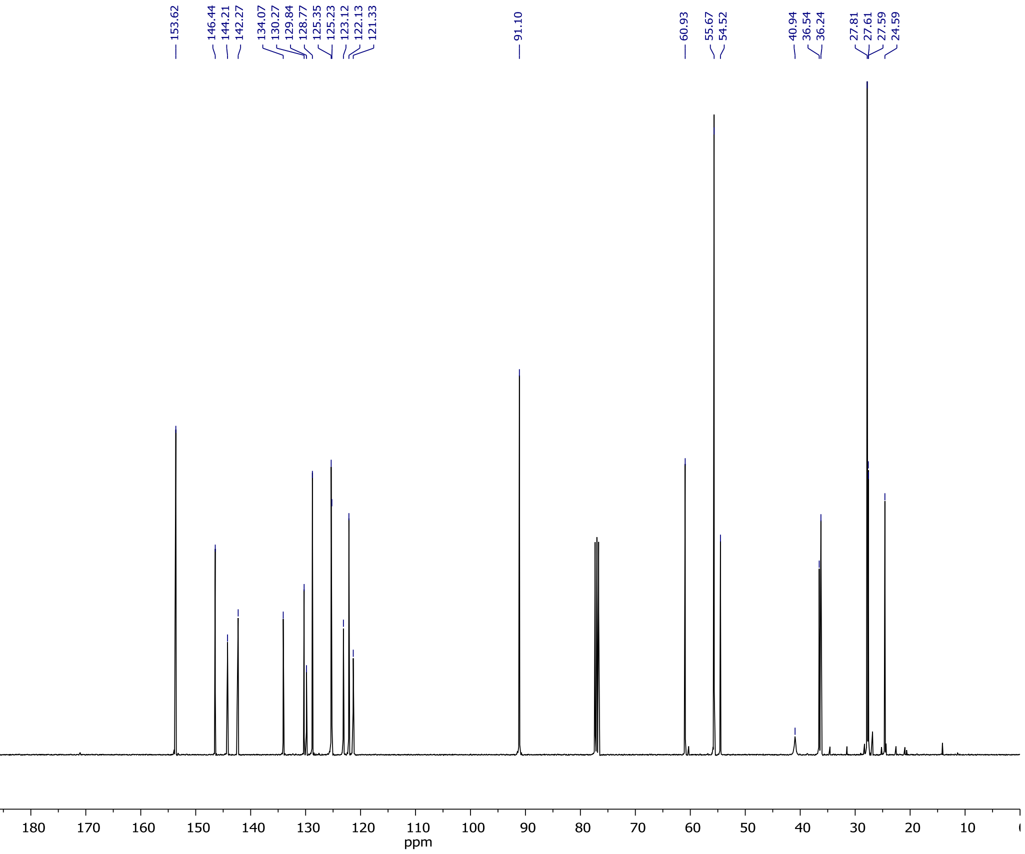

Supplementary Figure 44: ^{1}H and ^{13}C-NMR of Compound 9a.

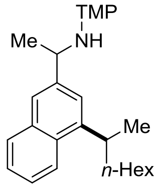

9b
($CDCl_3$, 300 MHz)

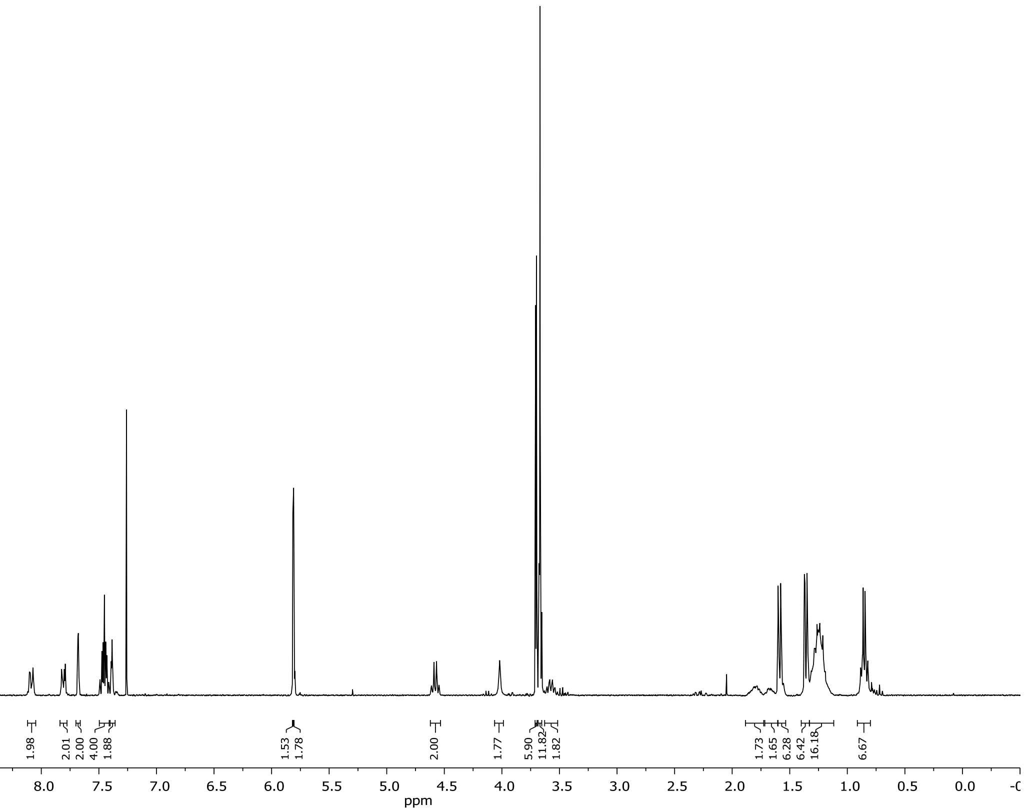

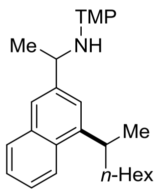

9b
($CDCl_3$, 75 MHz)

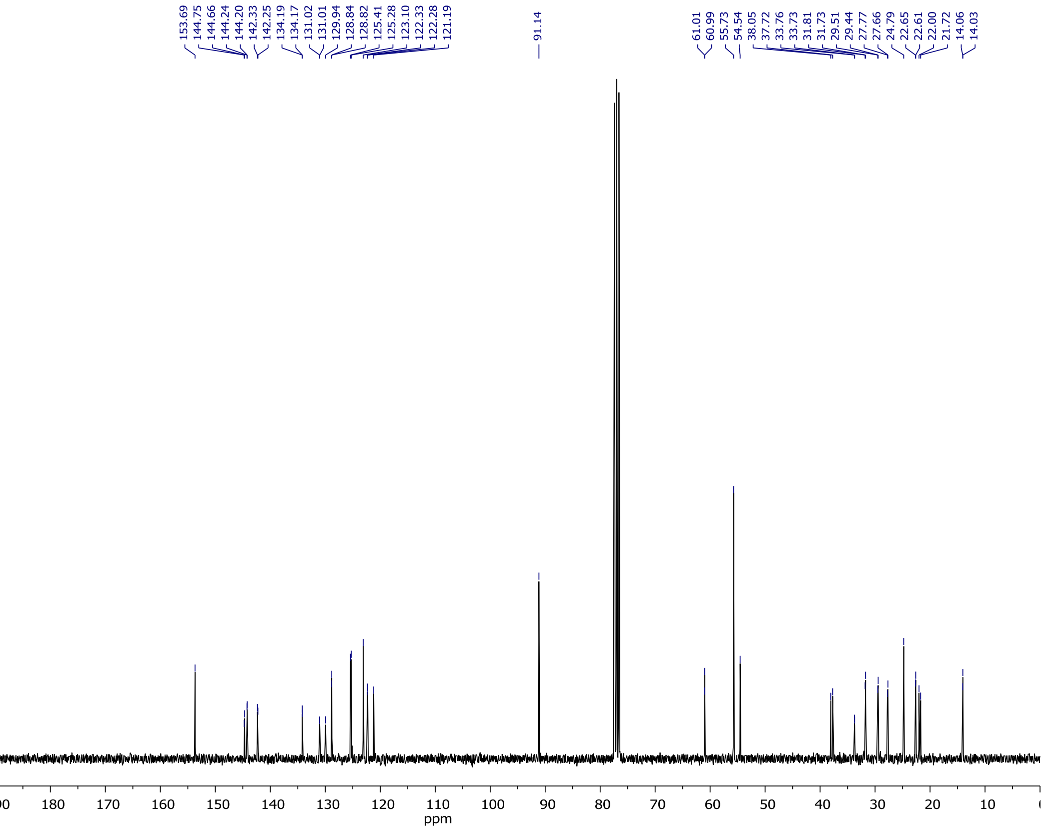

Supplementary Figure 45: ^{1}H and ^{13}C-NMR of Compound 9b.

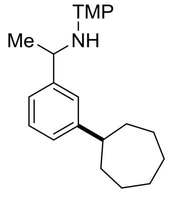

9c
($CDCl_3$, 500 MHz)

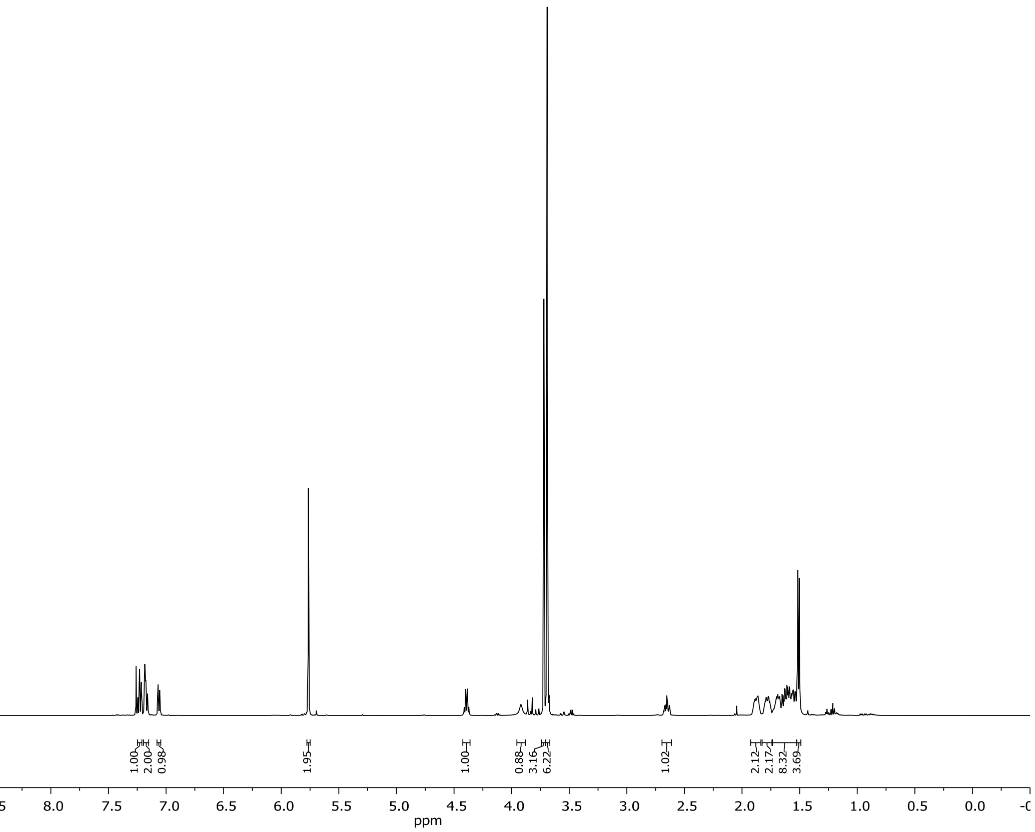

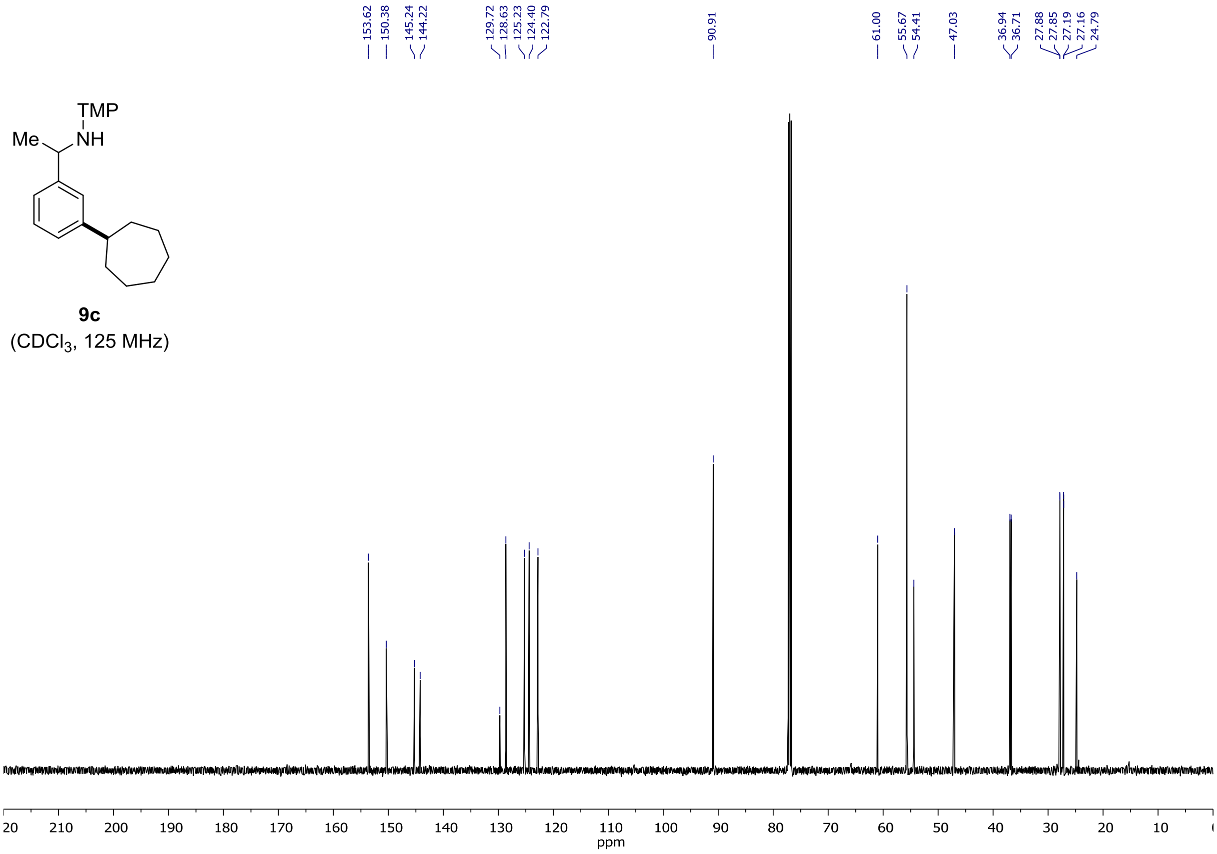

Supplementary Figure 46: ^{1}H and ^{13}C-NMR of Compound 9c.

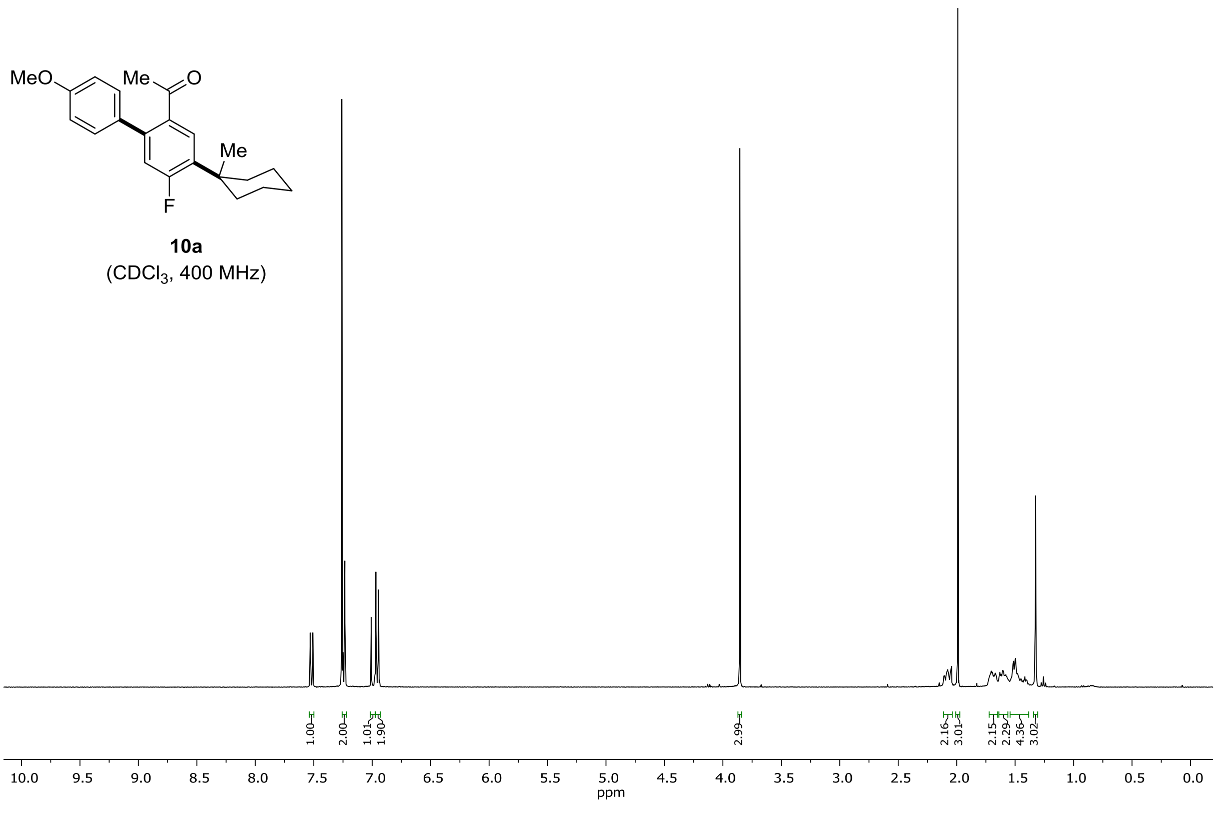

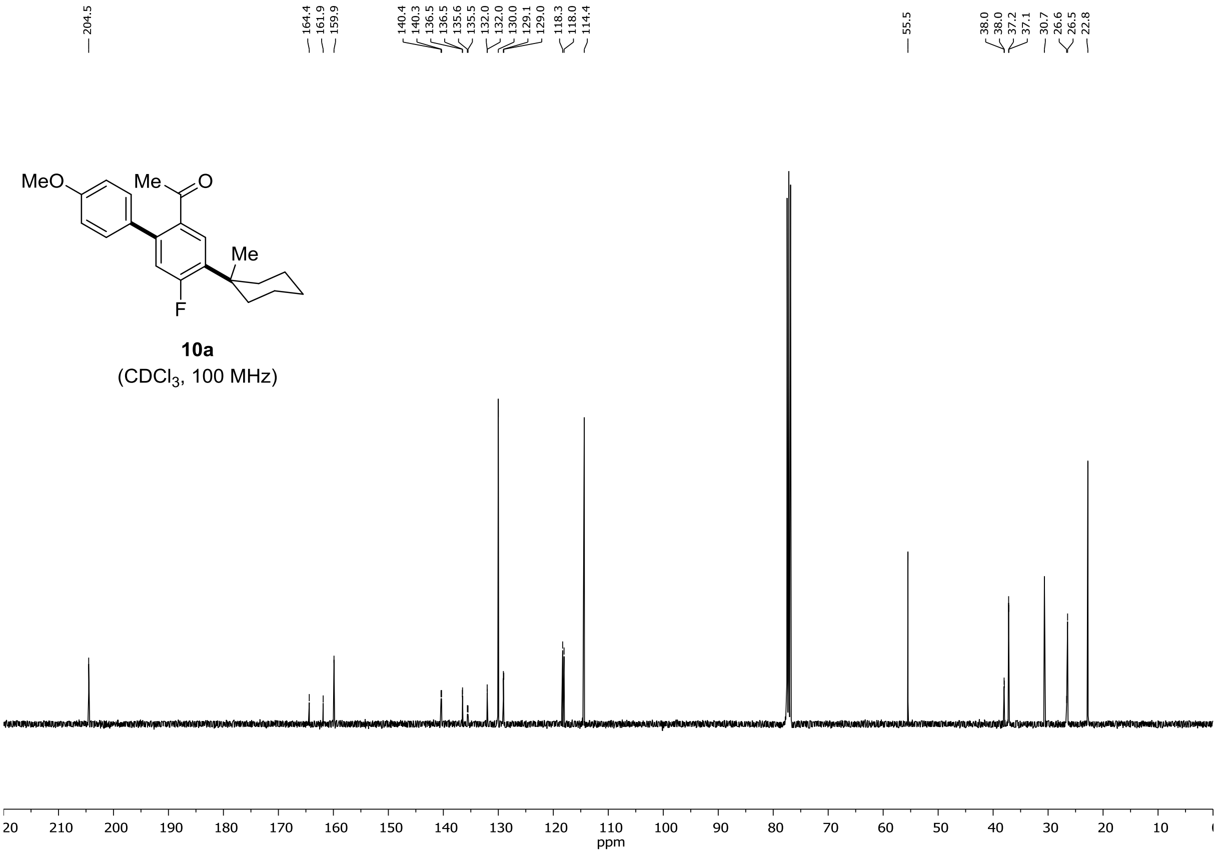

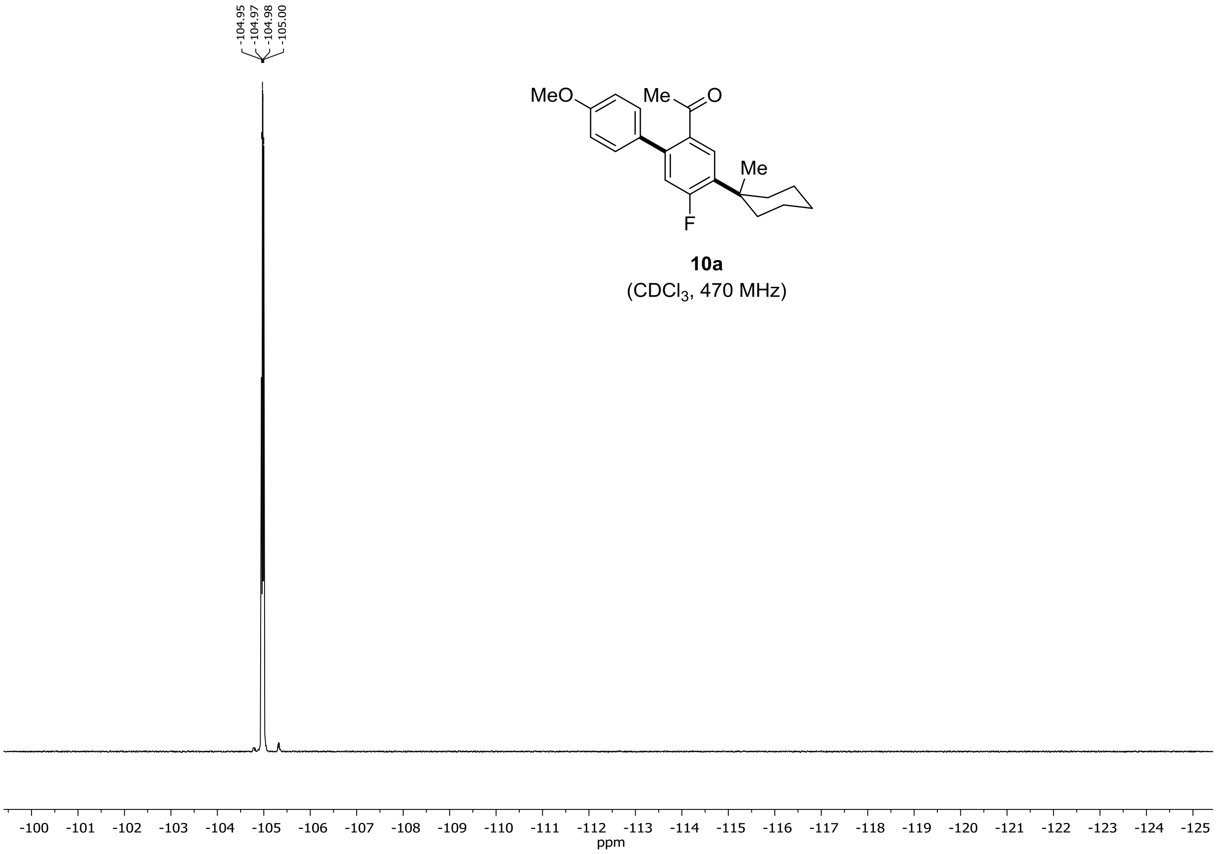

Supplementary Figure 47: ^{1}H, ^{13}C and ^{19}F-NMR of Compound 10a.

10b
($CDCl_3$, 400 MHz)

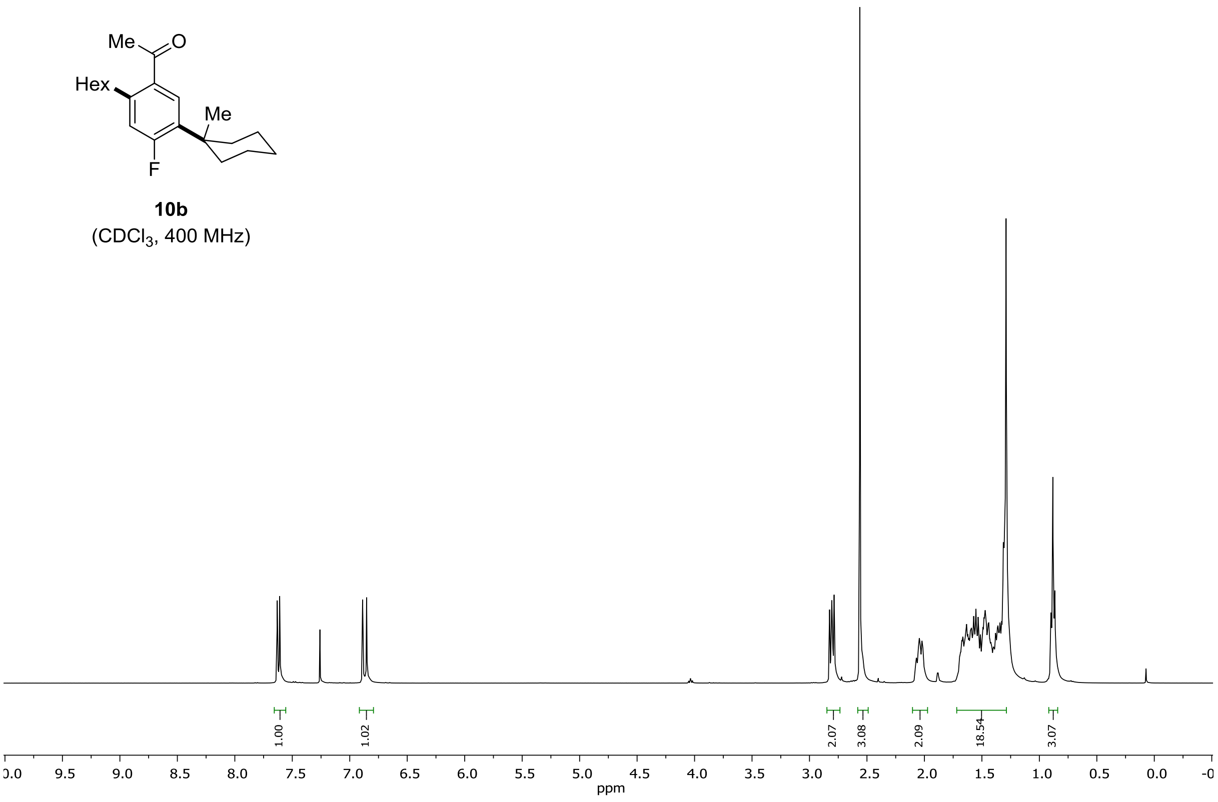

10b
($CDCl_3$, 100 MHz)

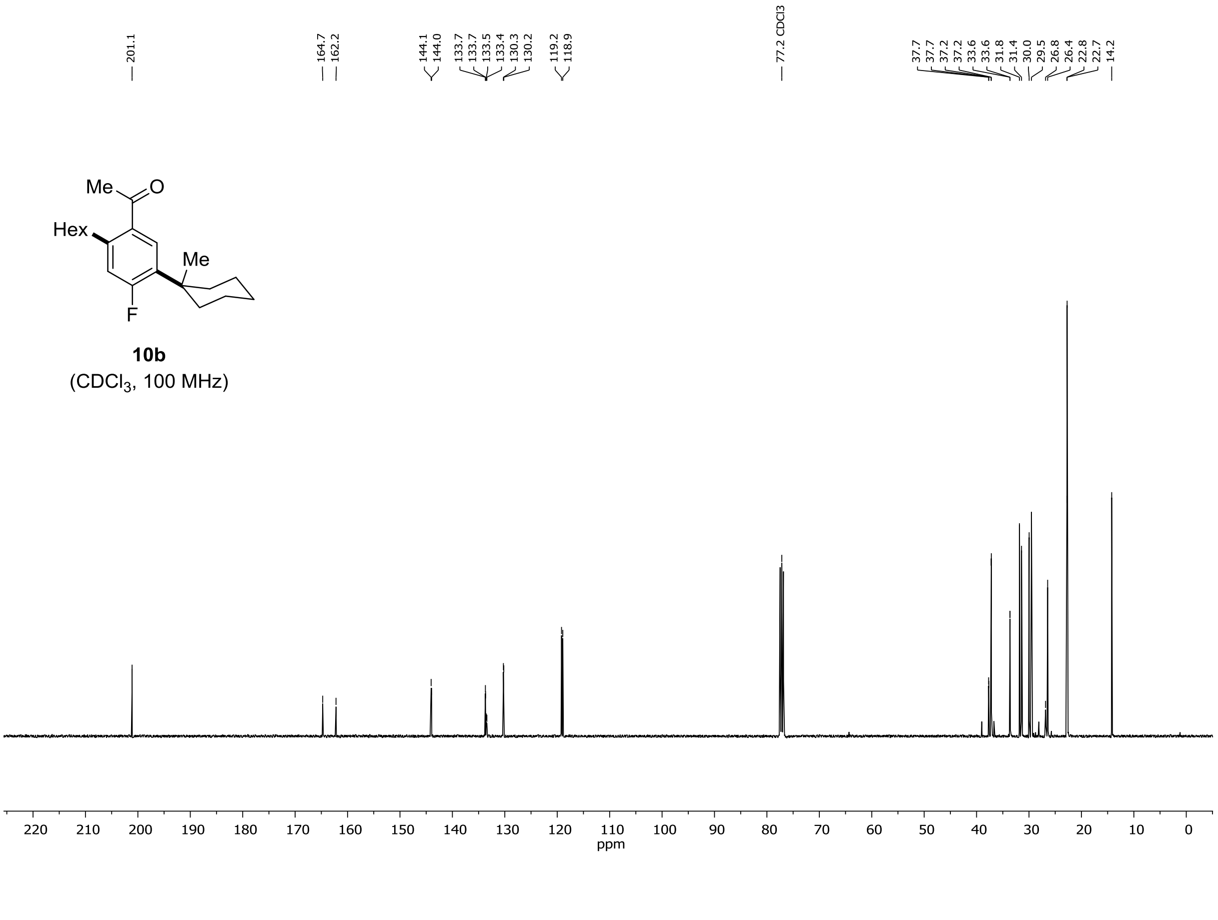

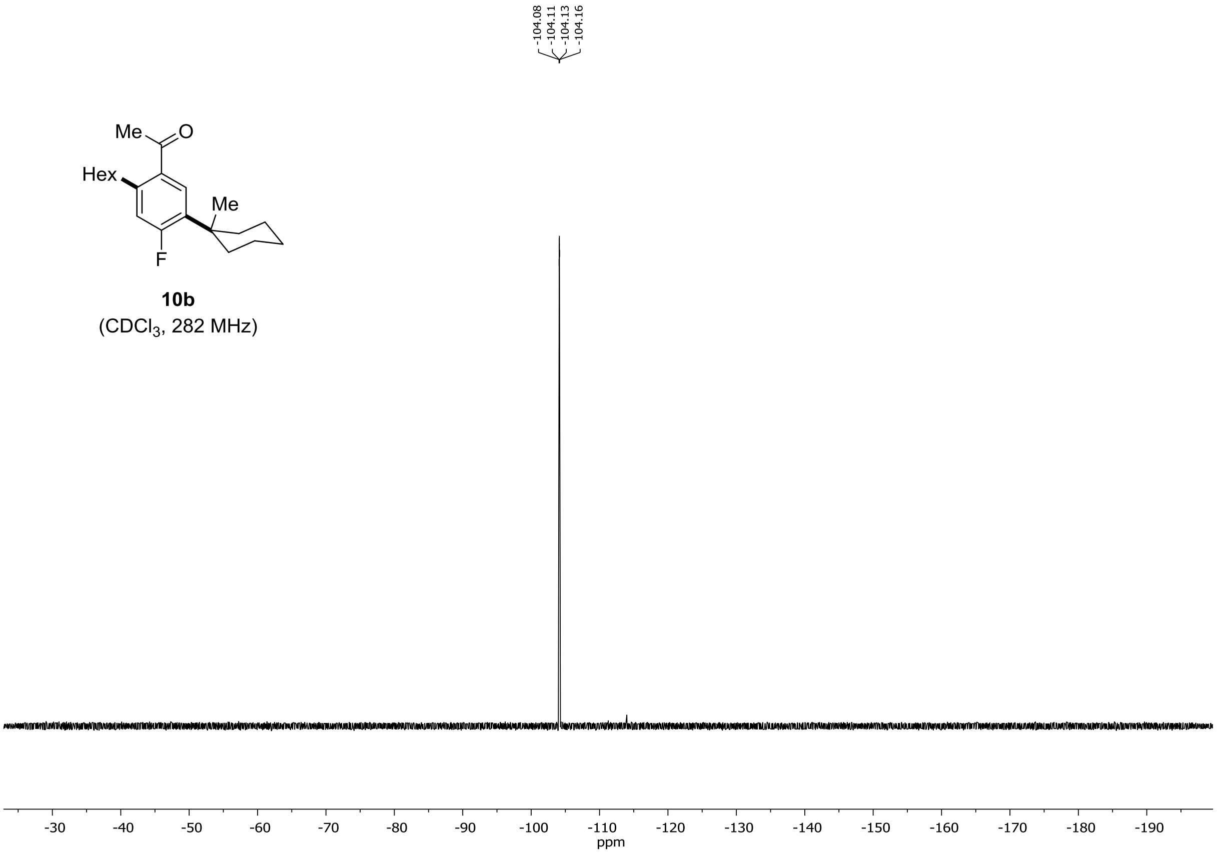

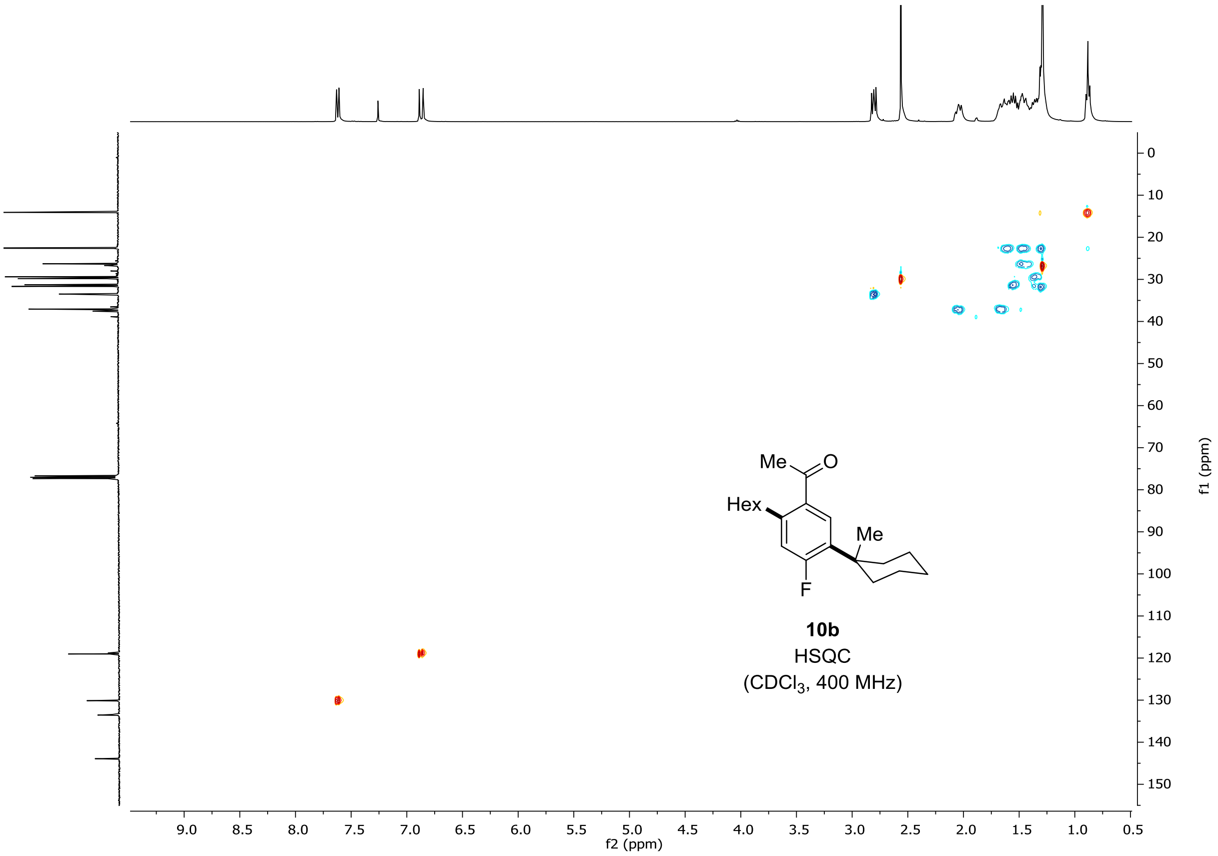

Supplementary Figure 48: ^{1}H, ^{13}C, ^{19}F and HSQC-NMR of Compound 10b.

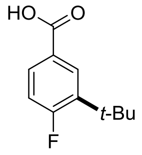

11
($CDCl_3$, 500 MHz)

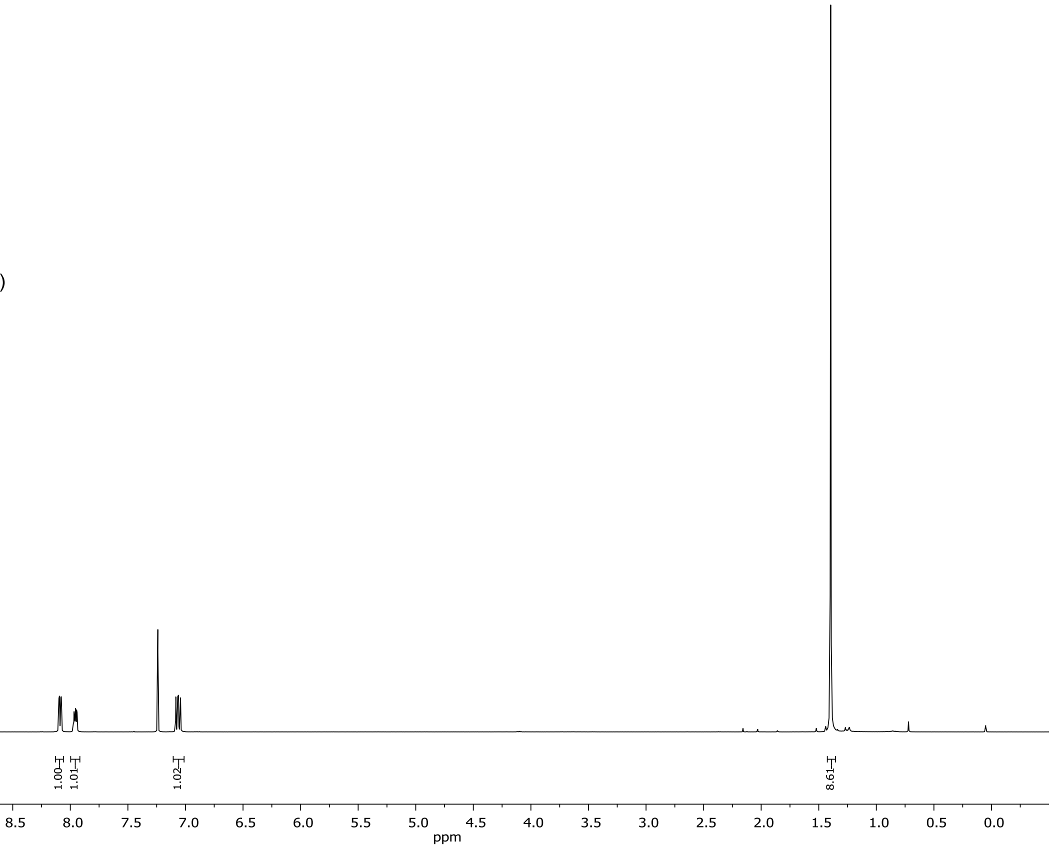

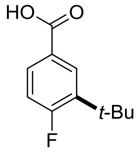

11
($CDCl_3$, 125 MHz)

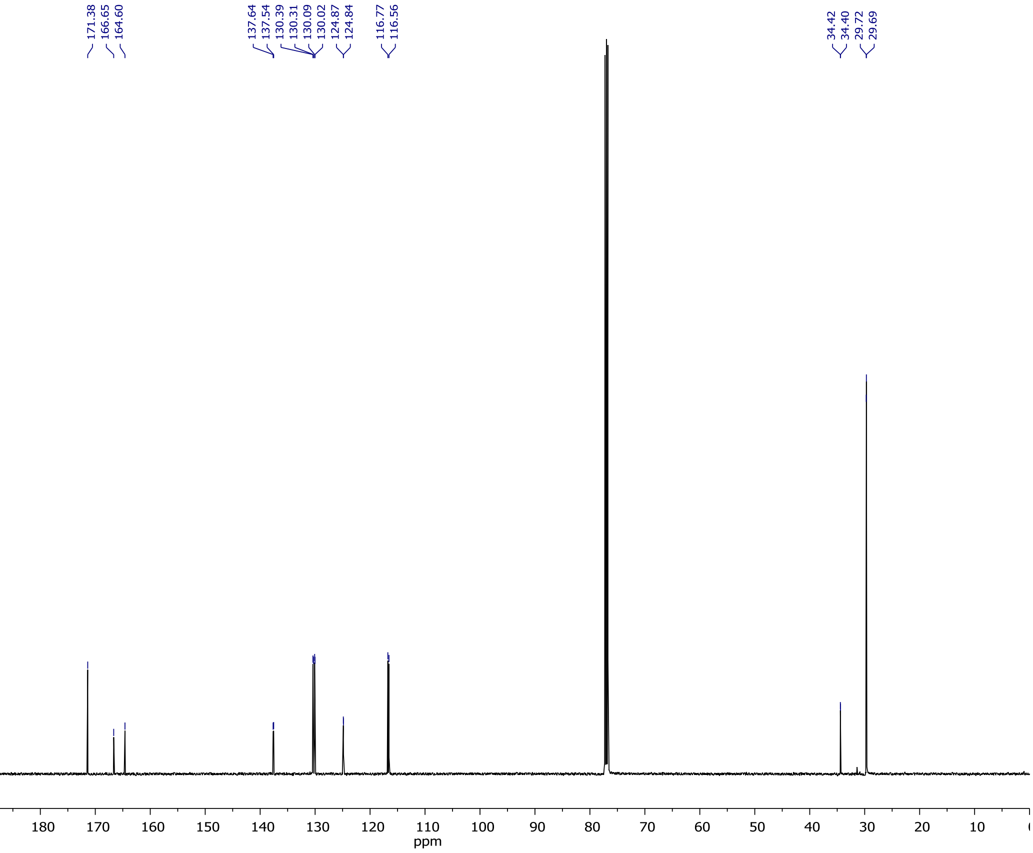

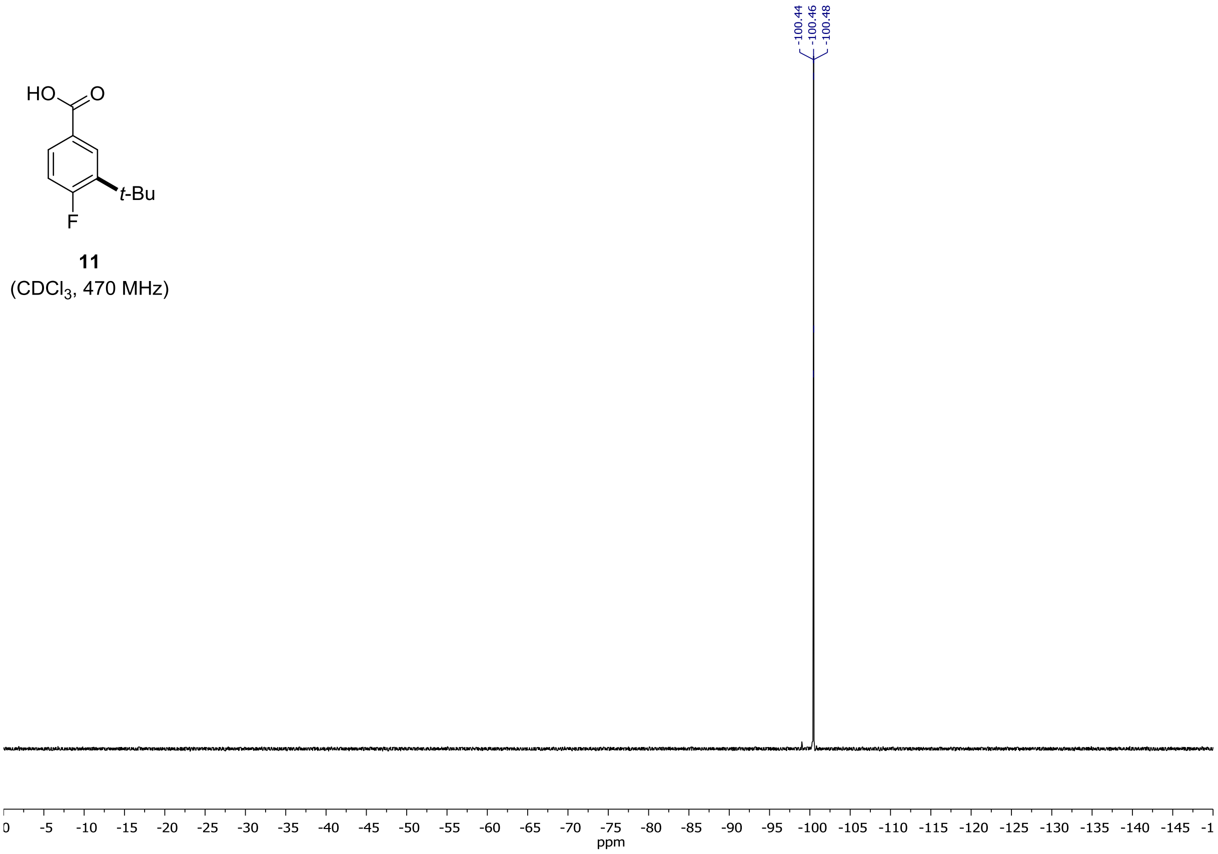

Supplementary Figure 49: ^{1}H, ^{13}C and ^{19}F-NMR of Compound 11.

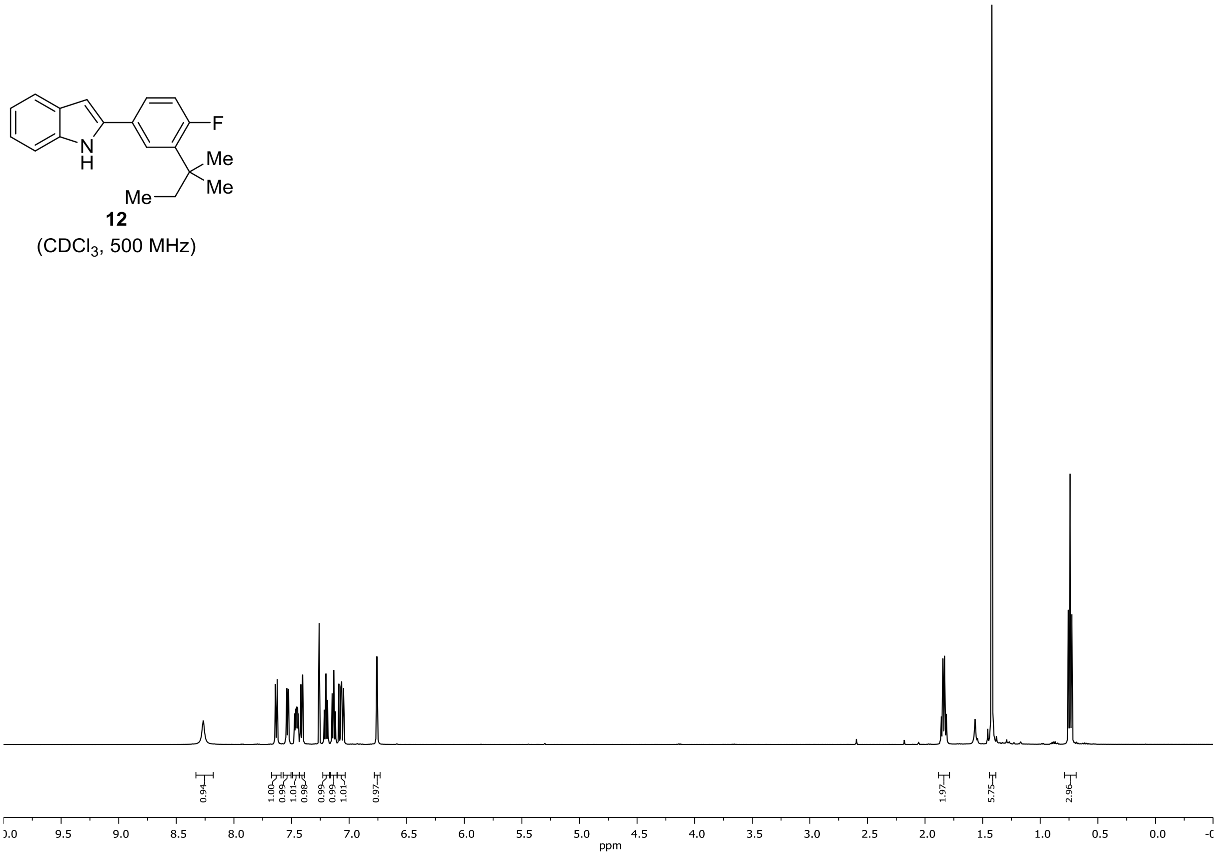

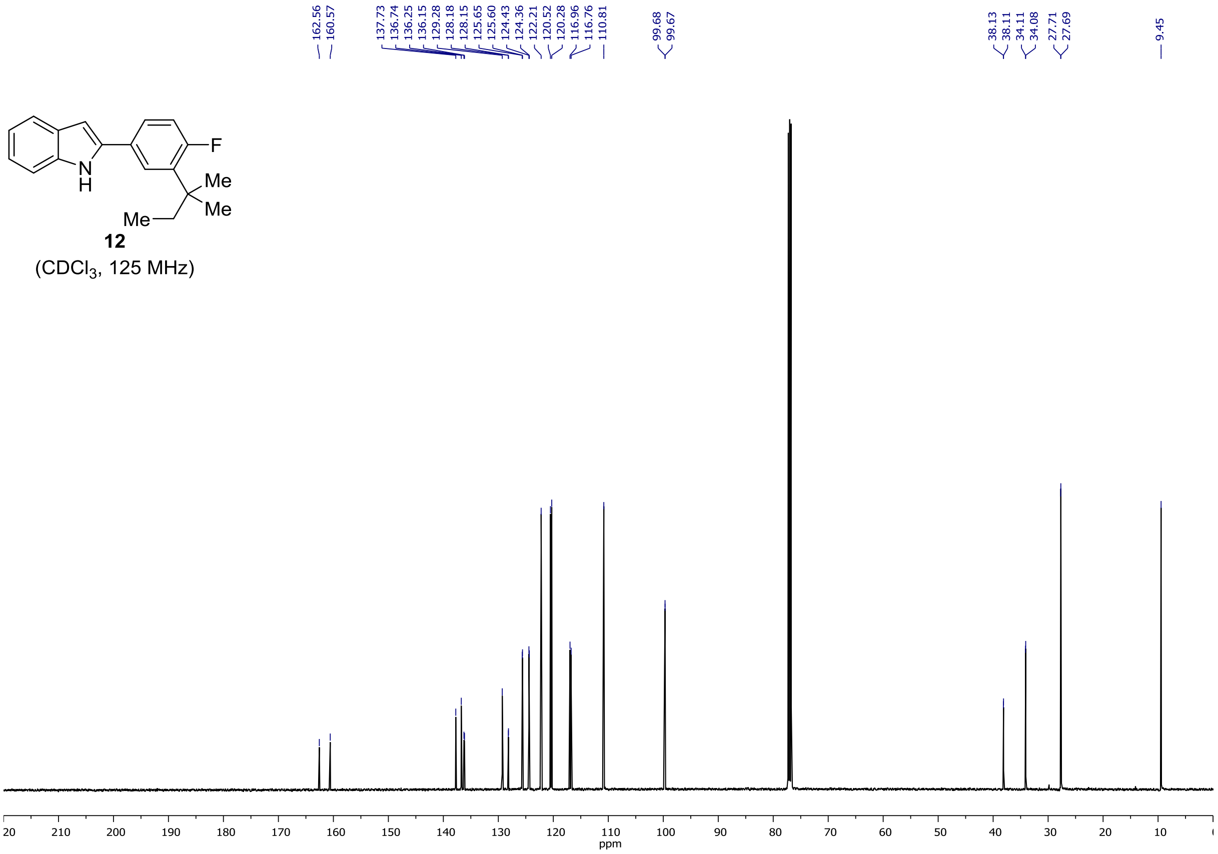

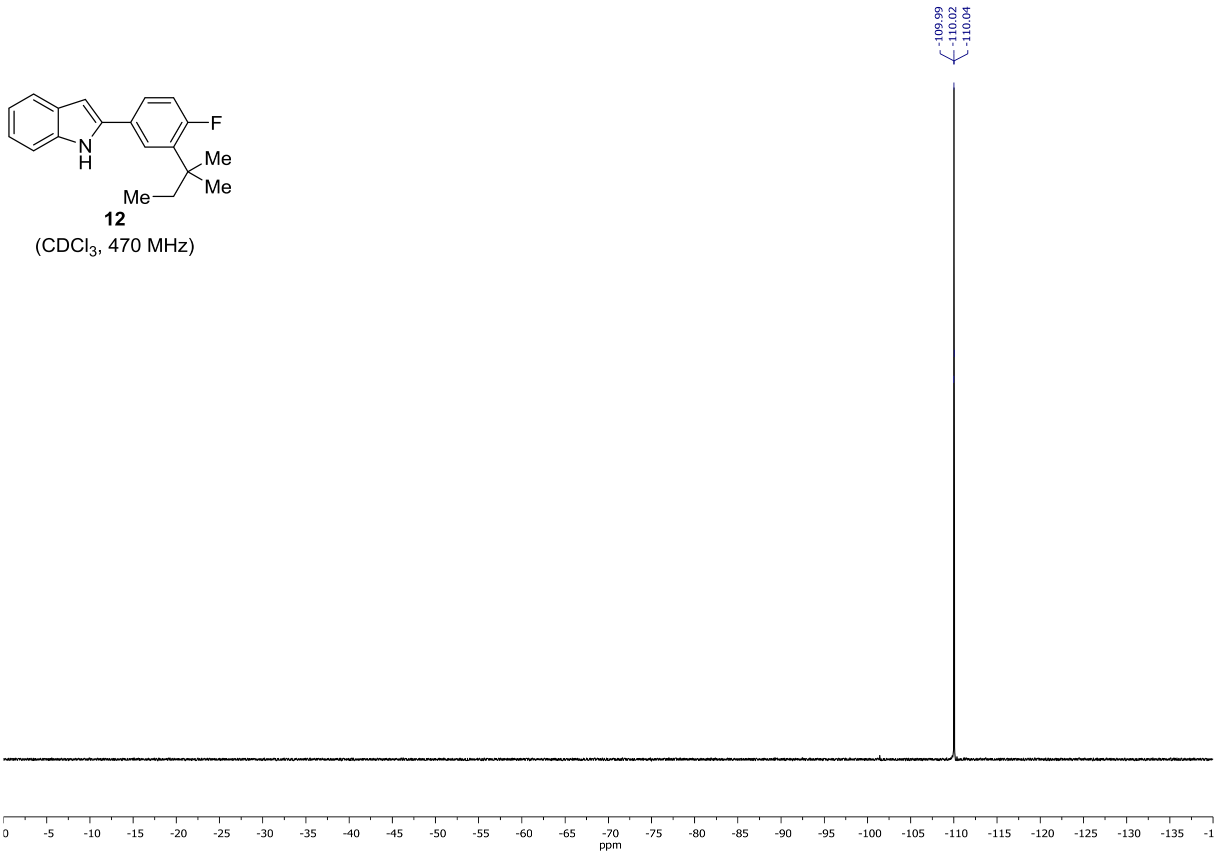

Supplementary Figure 50: ^{1}H, ^{13}C and ^{19}F-NMR of Compound 12.

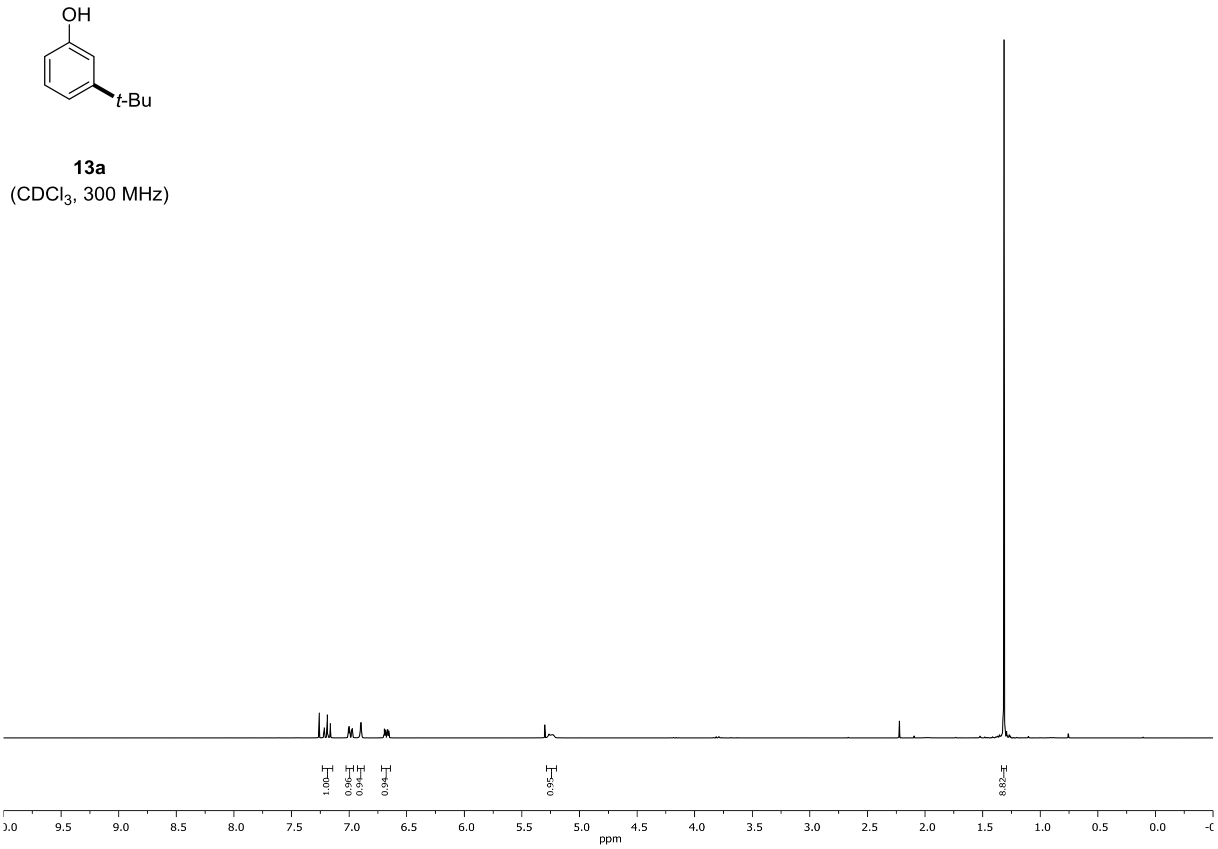

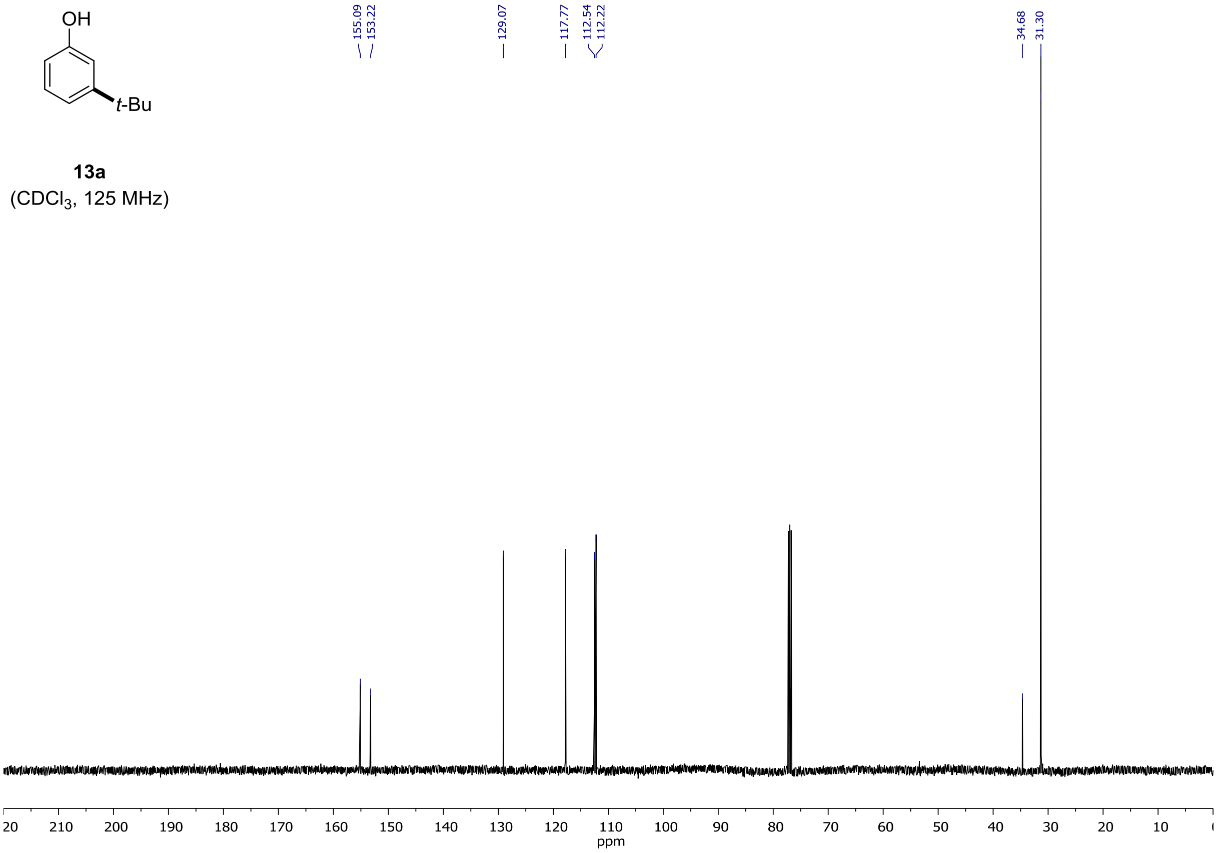

Supplementary Figure 51: ^{1}H and ^{13}C-NMR of Compound 13a.

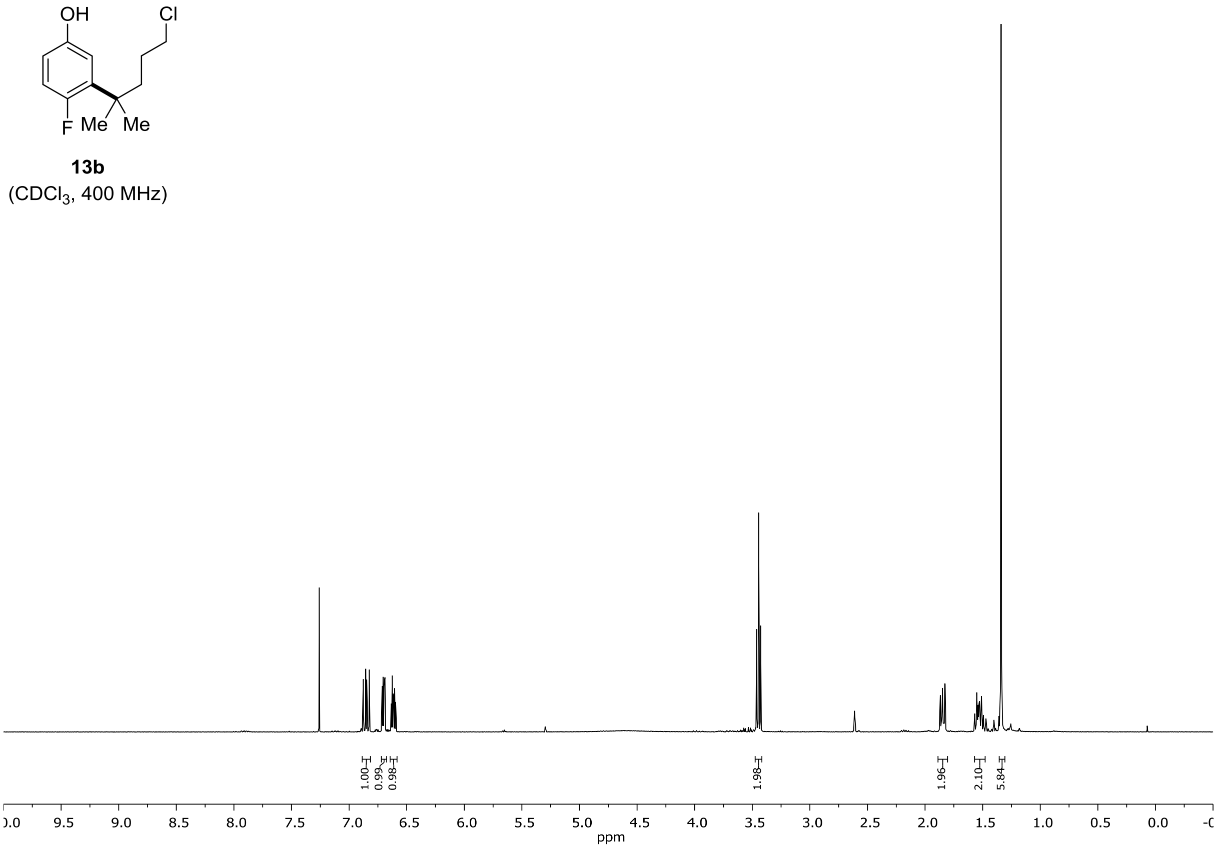

13b
($CDCl_3$, 400 MHz)

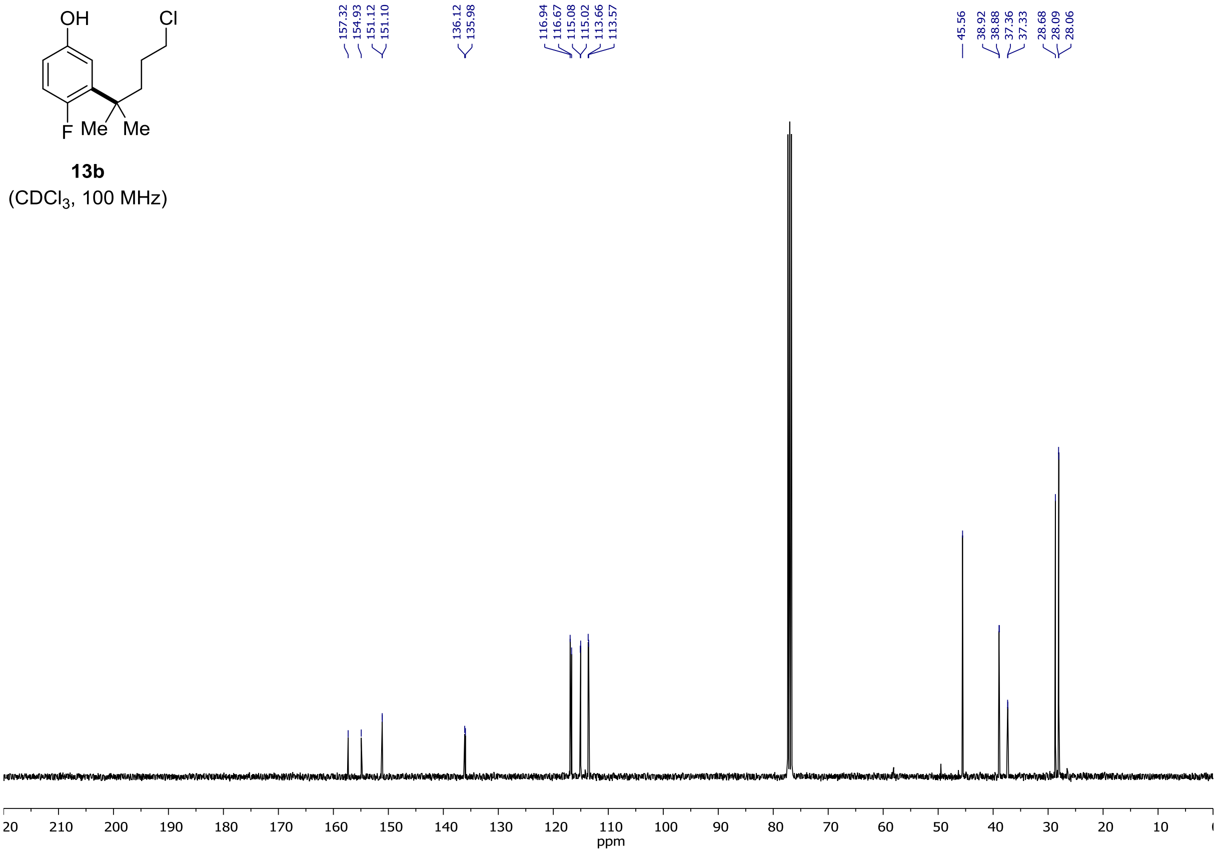

13b
($CDCl_3$, 100 MHz)

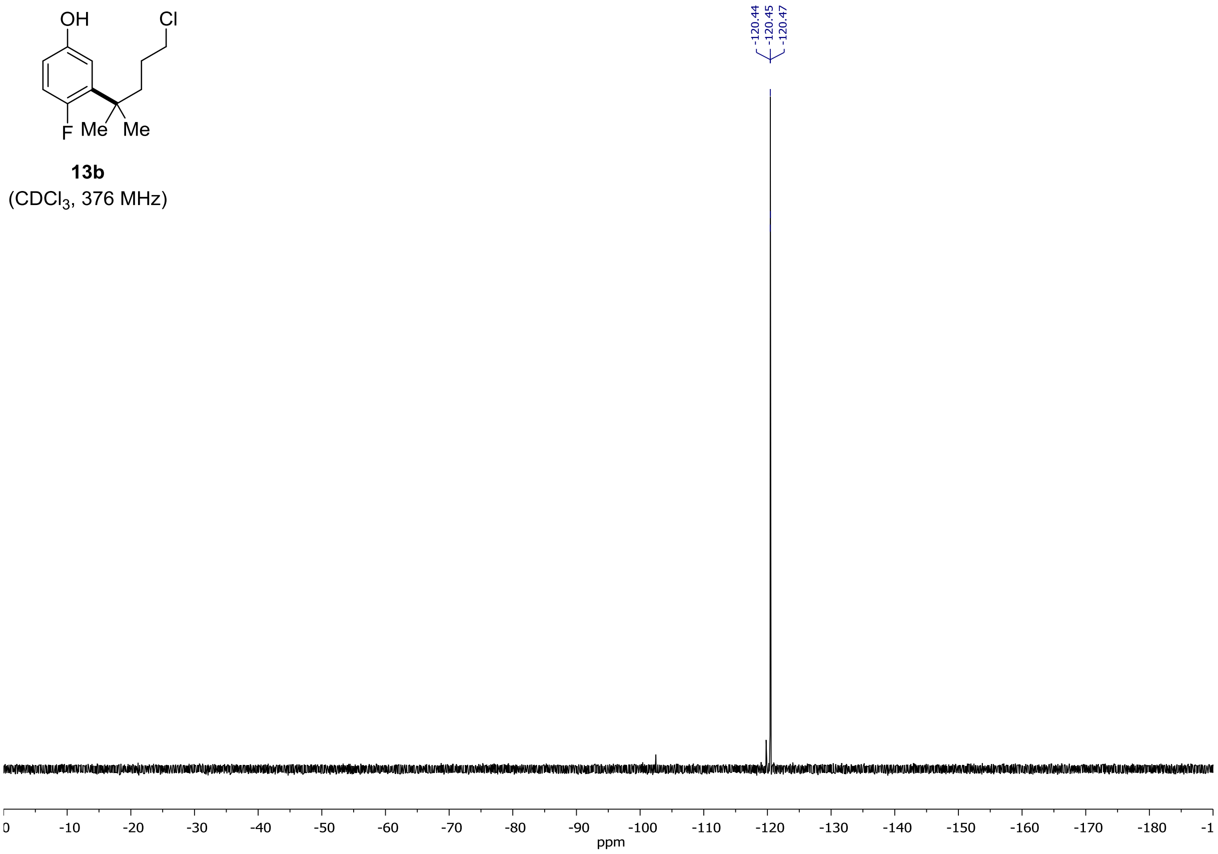

Supplementary Figure 52: ^{1}H, ^{13}C and ^{19}F-NMR of Compound 13b.

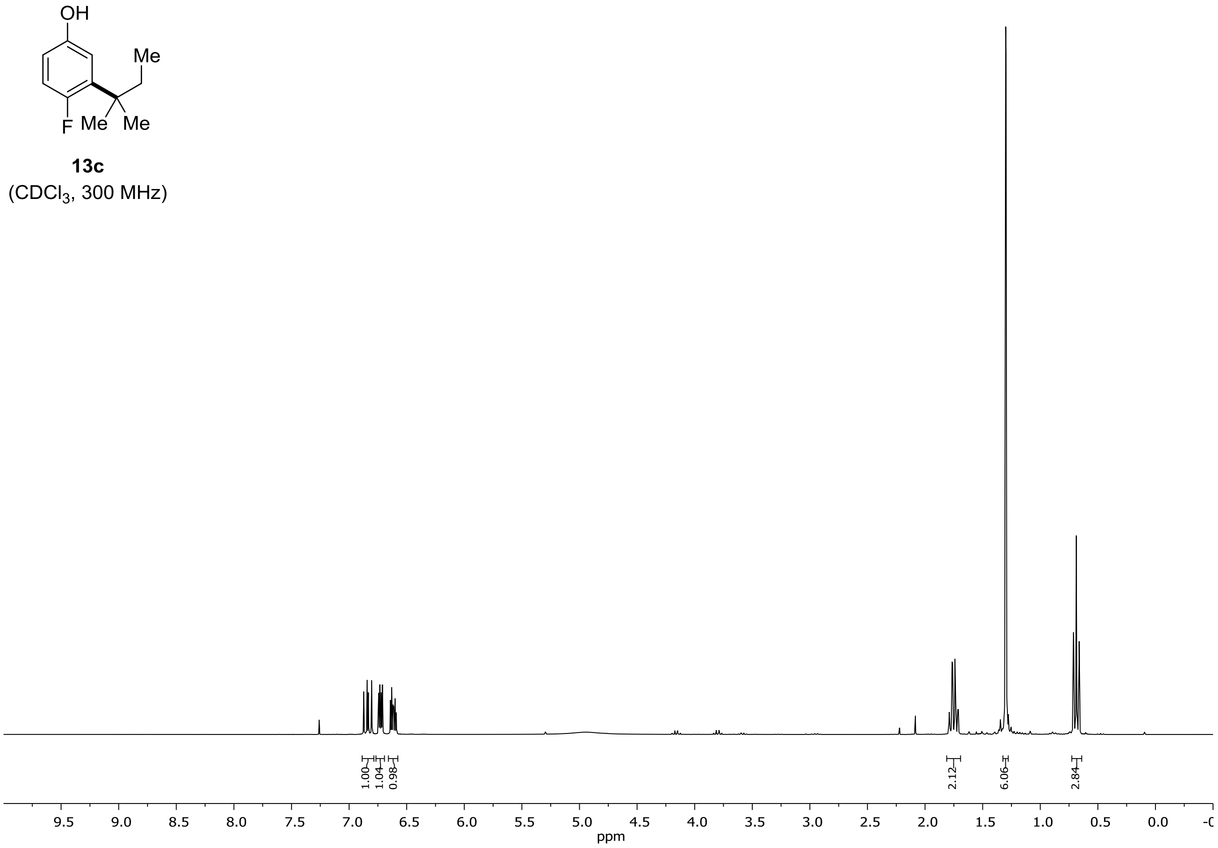

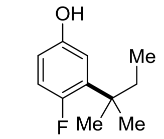

13c
($CDCl_3$, 125 MHz)

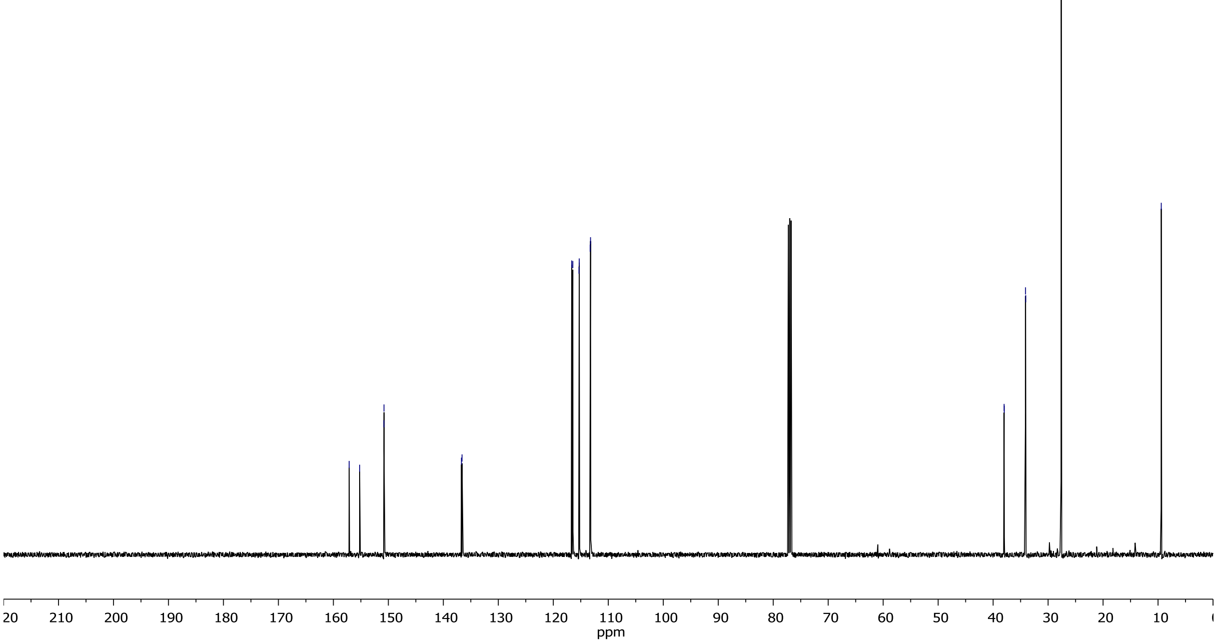

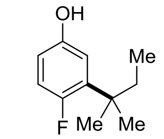

13c
($CDCl_3$, 282 MHz)

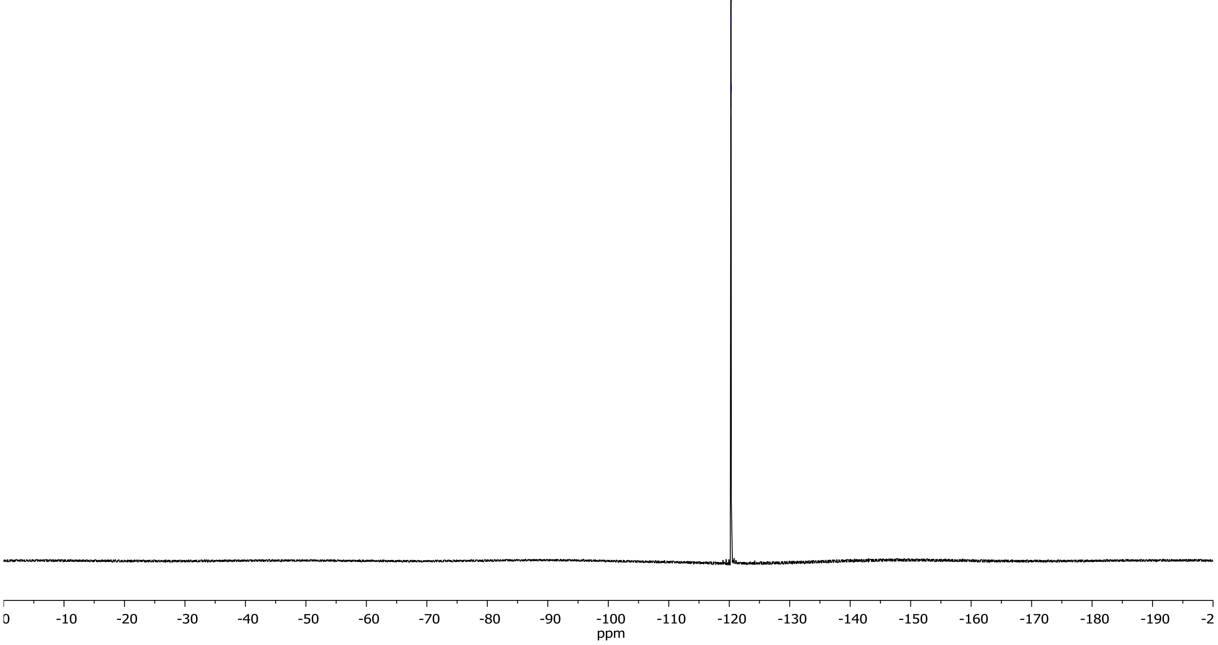

Supplementary Figure 53: ^{1}H, ^{13}C and ^{19}F-NMR of Compound 13c.

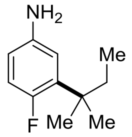

14
($CDCl_3$, 400 MHz)

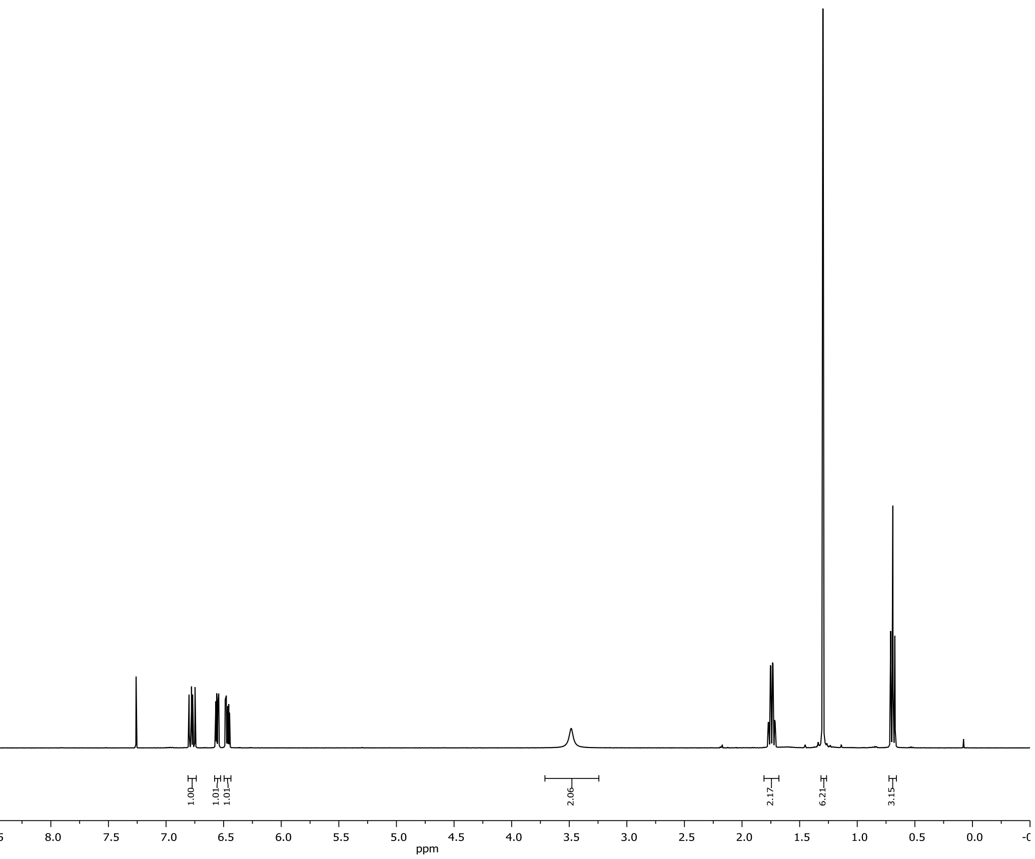

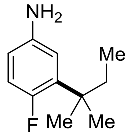

14
($CDCl_3$, 100 MHz)

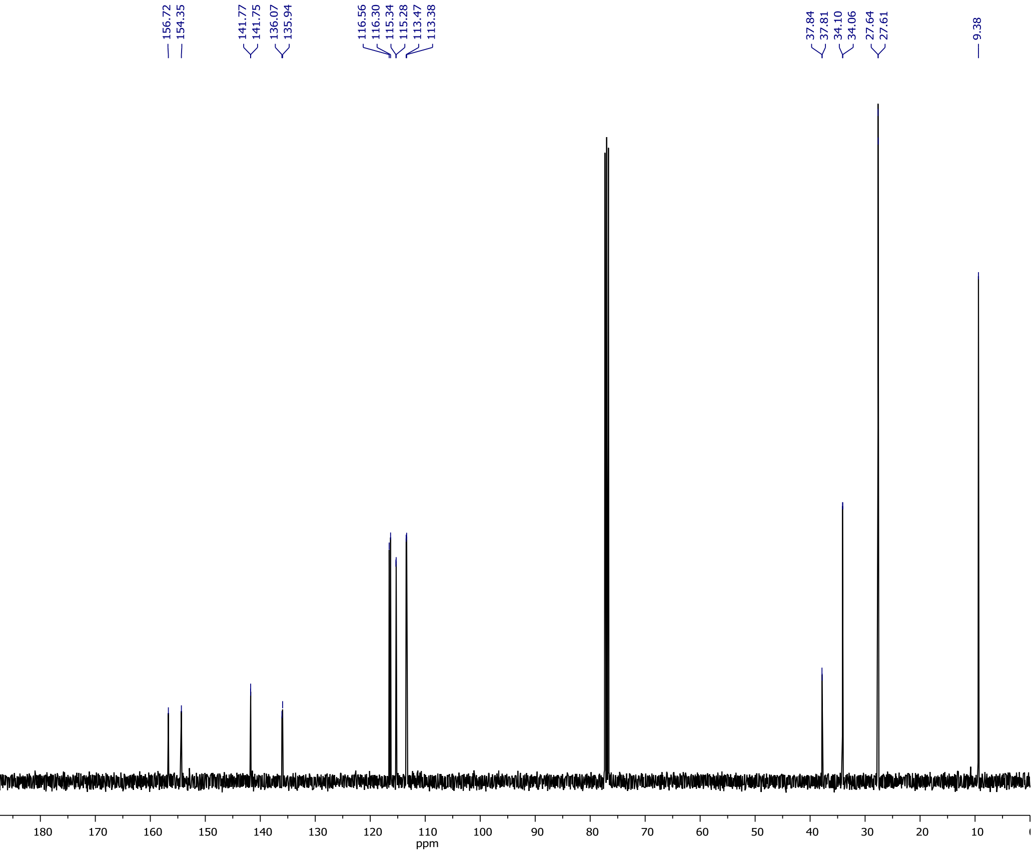

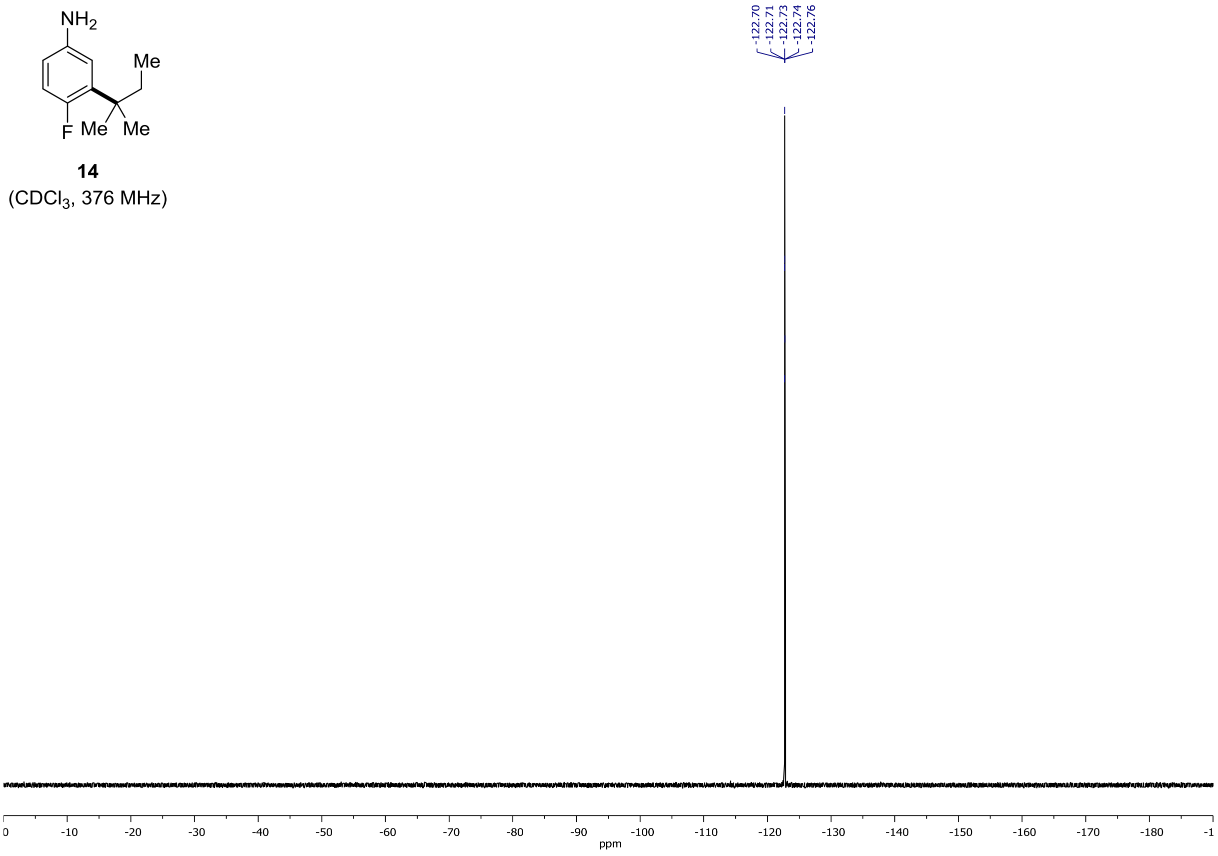

Supplementary Figure 54: ^{1}H, ^{13}C and ^{19}F-NMR of Compound 14.

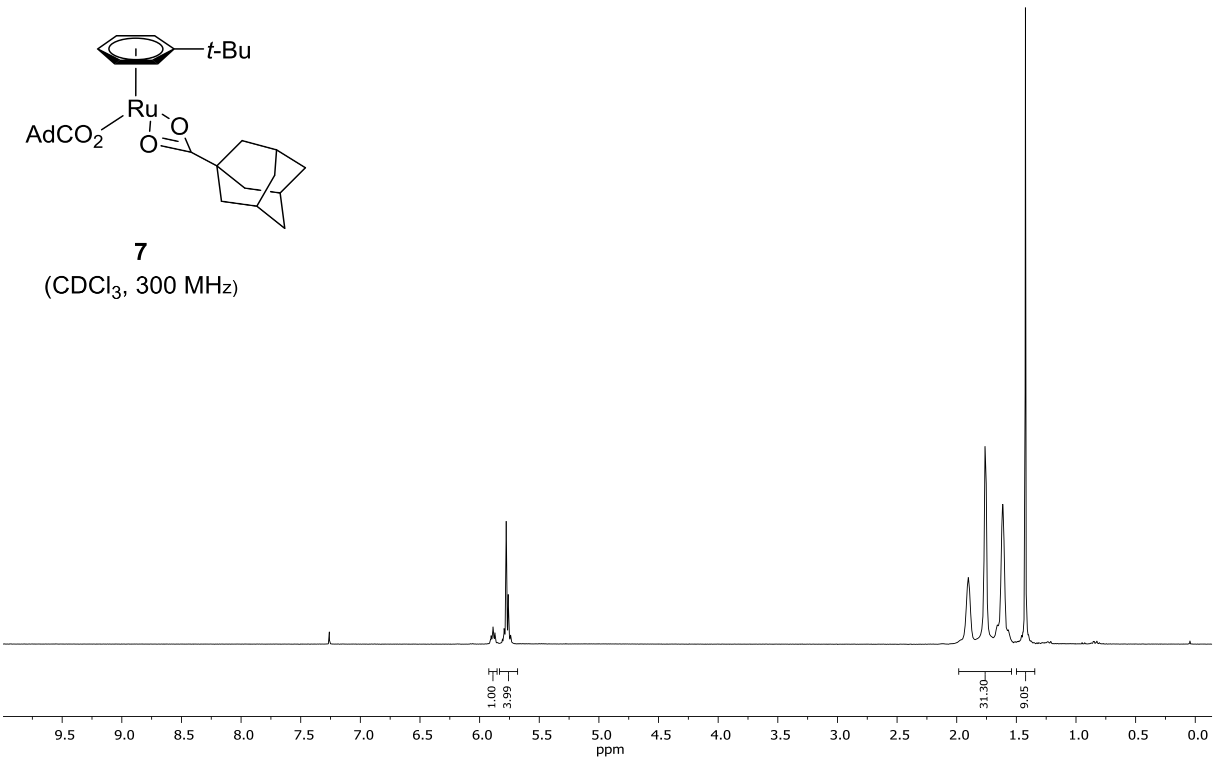

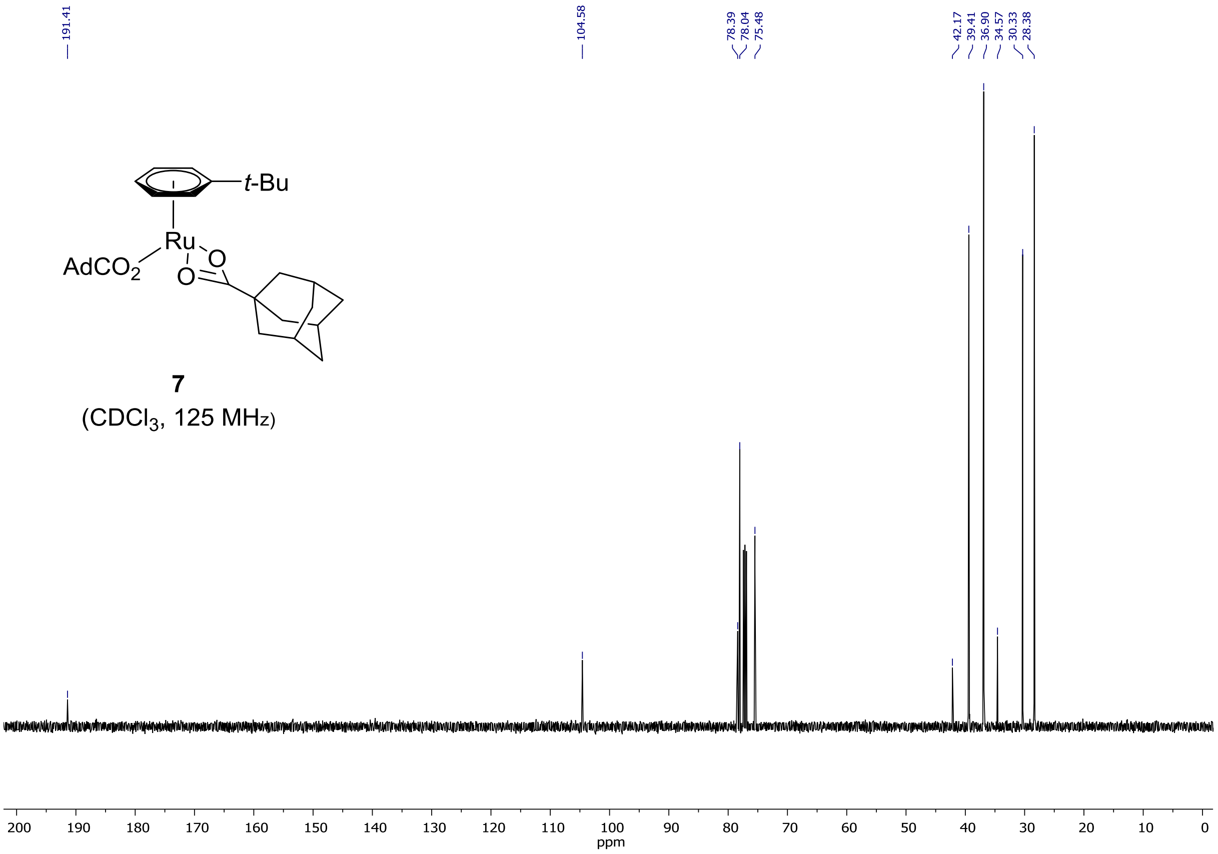

Supplementary Figure 55: ^{1}H and ^{13}C-NMR of Compound 7.

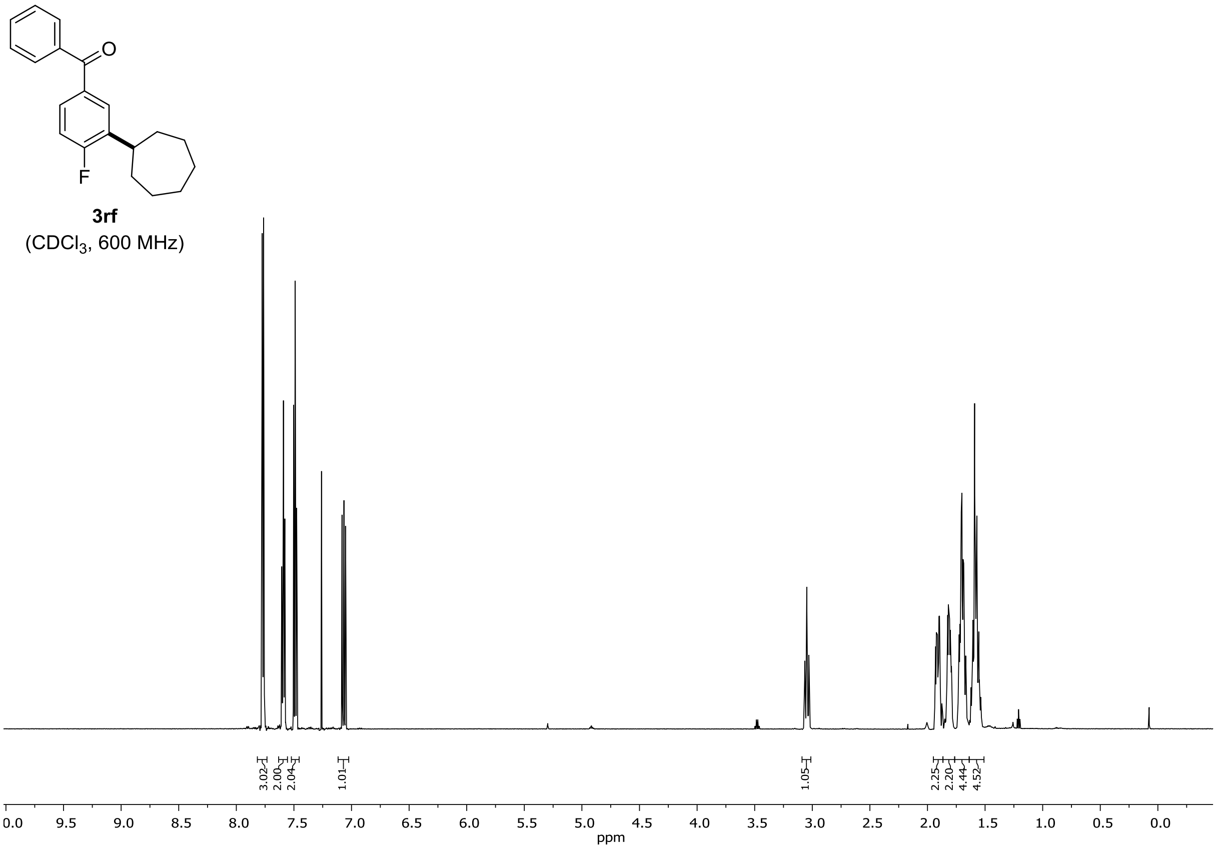

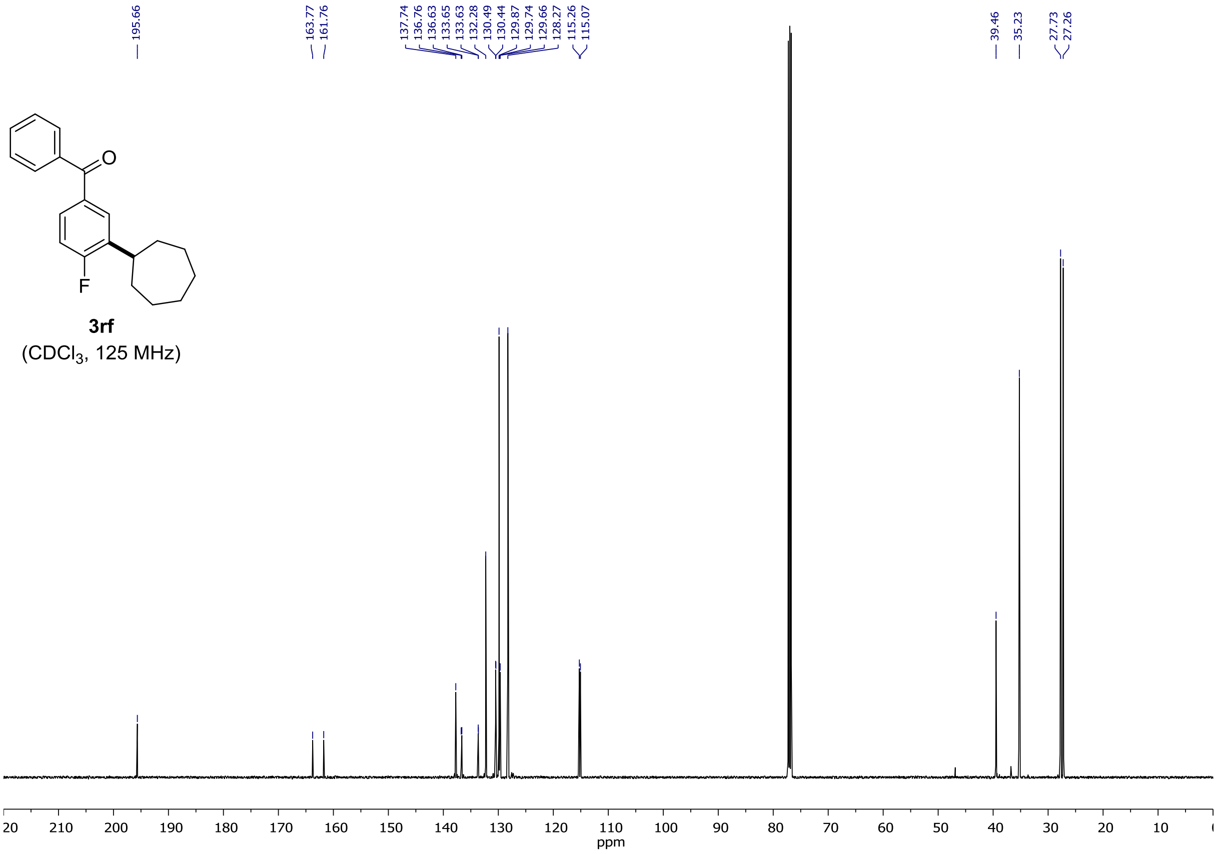

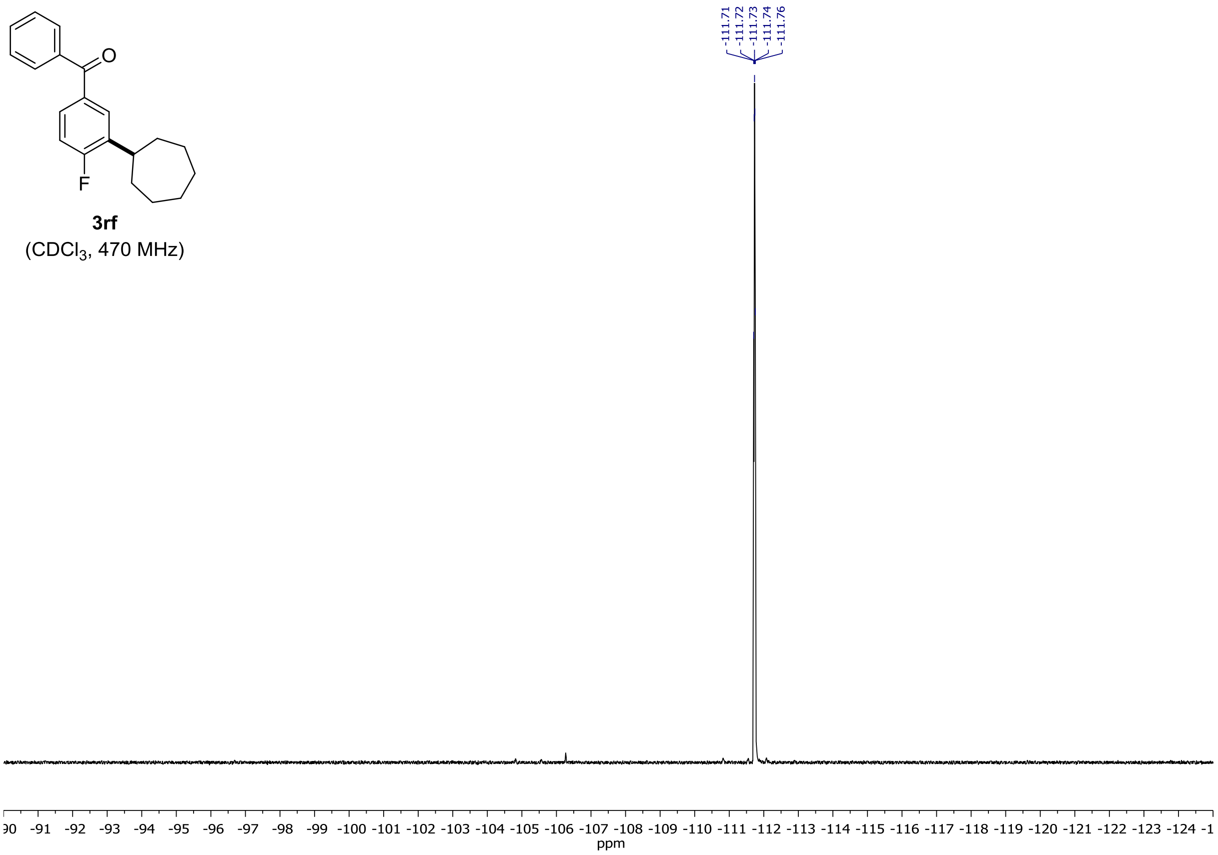

Supplementary Figure 56: ^{1}H, ^{13}C and ^{19}F-NMR of Compound 3rf.

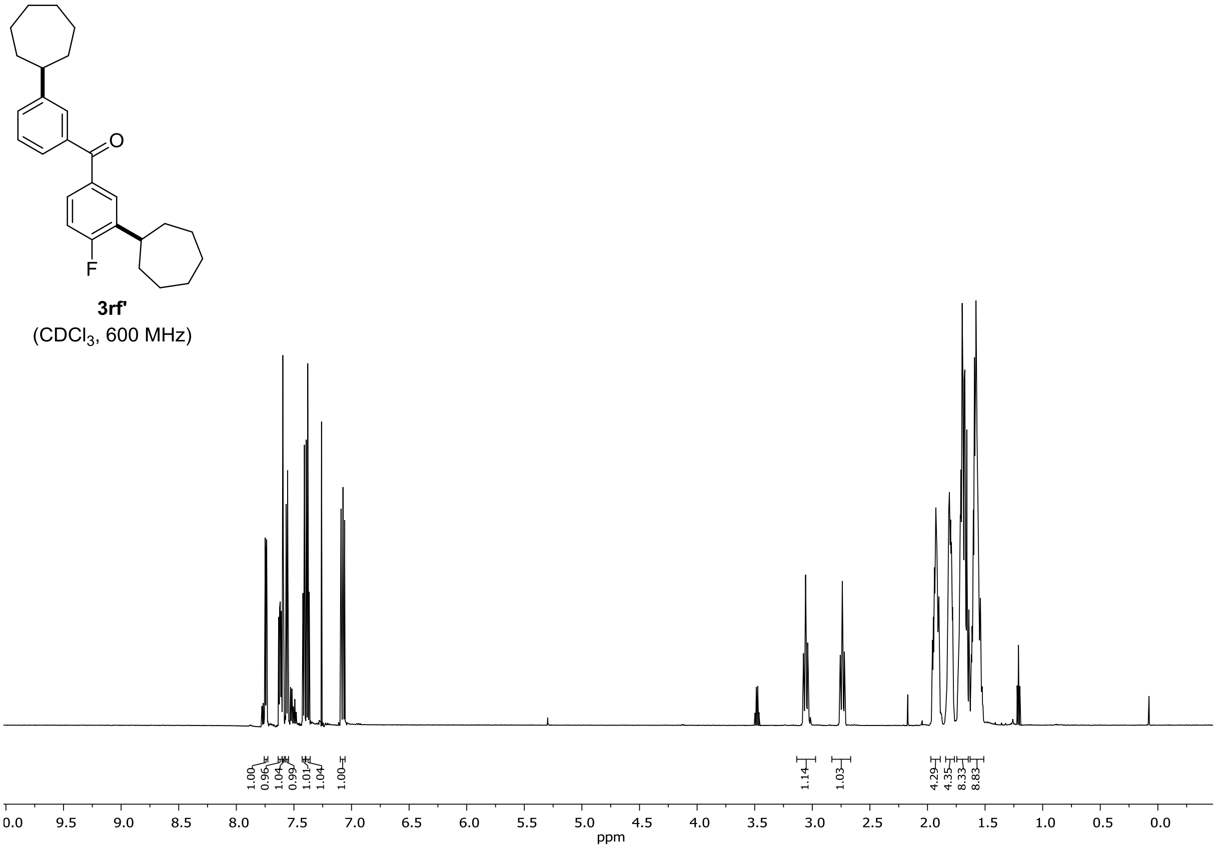
3rf'
(CDCl$_3$, 600 MHz)
F
O
1.00 0.96 1.04 0.99 1.01 1.04 1.00
1.14
1.03
4.29 4.35 8.33 8.83
0.0 9.5 9.0 8.5 8.0 7.5 7.0 6.5 6.0 5.5 5.0 4.5 4.0 3.5 3.0 2.5 2.0 1.5 1.0 0.5 0.0
ppm

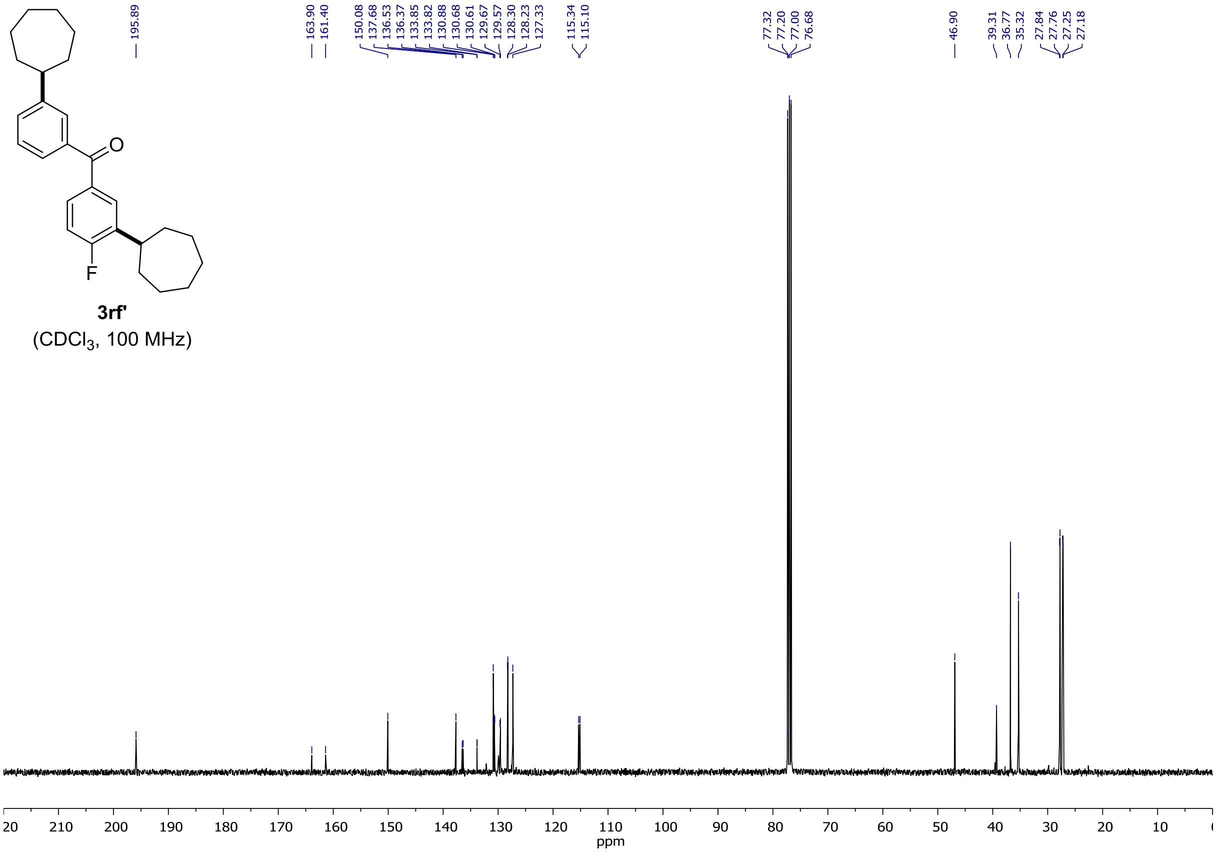
3rf'
(CDCl$_3$, 100 MHz)
F
O
195.89
163.90 161.40
150.08 137.68 136.53 136.37 133.85 133.82 130.88 130.68 130.61 129.67 129.57 128.30 128.23 127.33
115.34 115.10
77.32 77.20 77.00 76.68
46.90
39.31 36.77 35.32
27.84 27.76 27.25 27.18
20 210 200 190 180 170 160 150 140 130 120 110 100 90 80 70 60 50 40 30 20 10
ppm

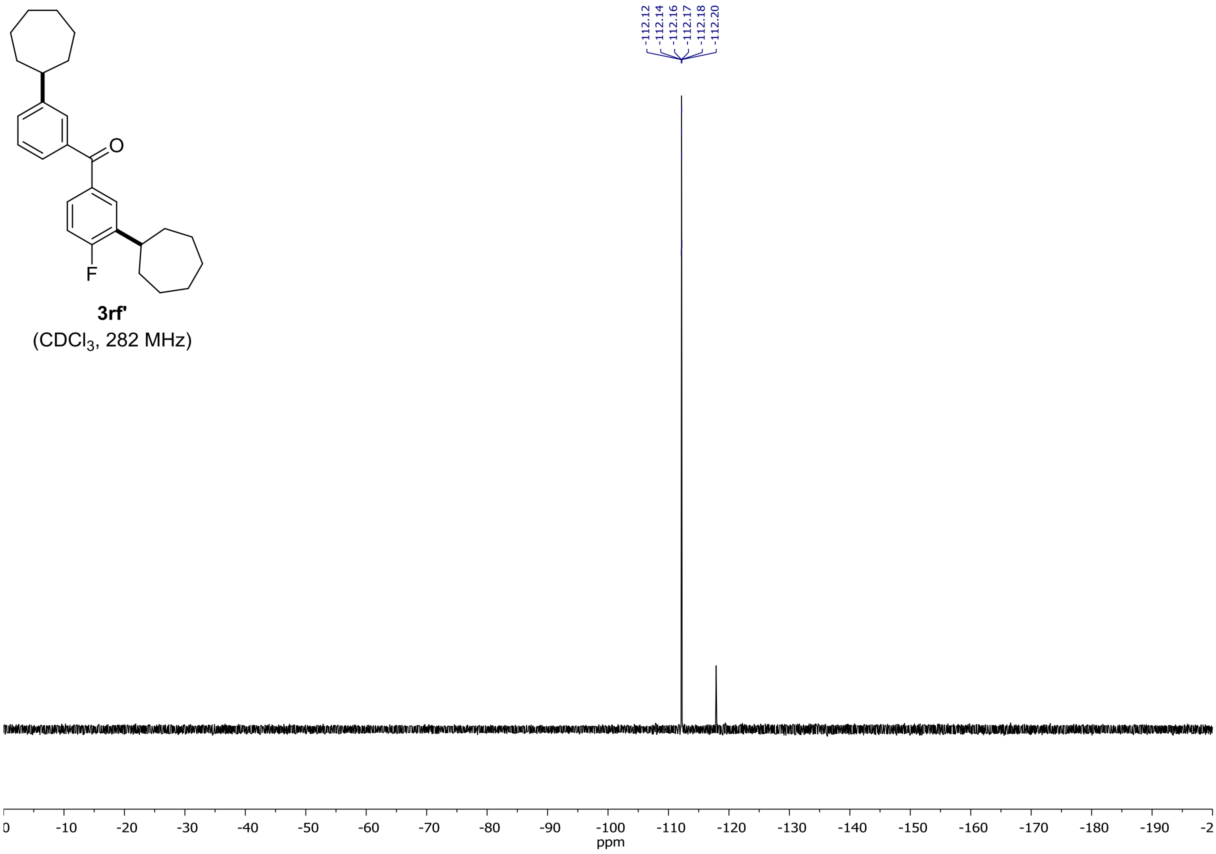

Supplementary Figure 57: ^{1}H, ^{13}C and ^{19}F-NMR of Compound 3rf'.

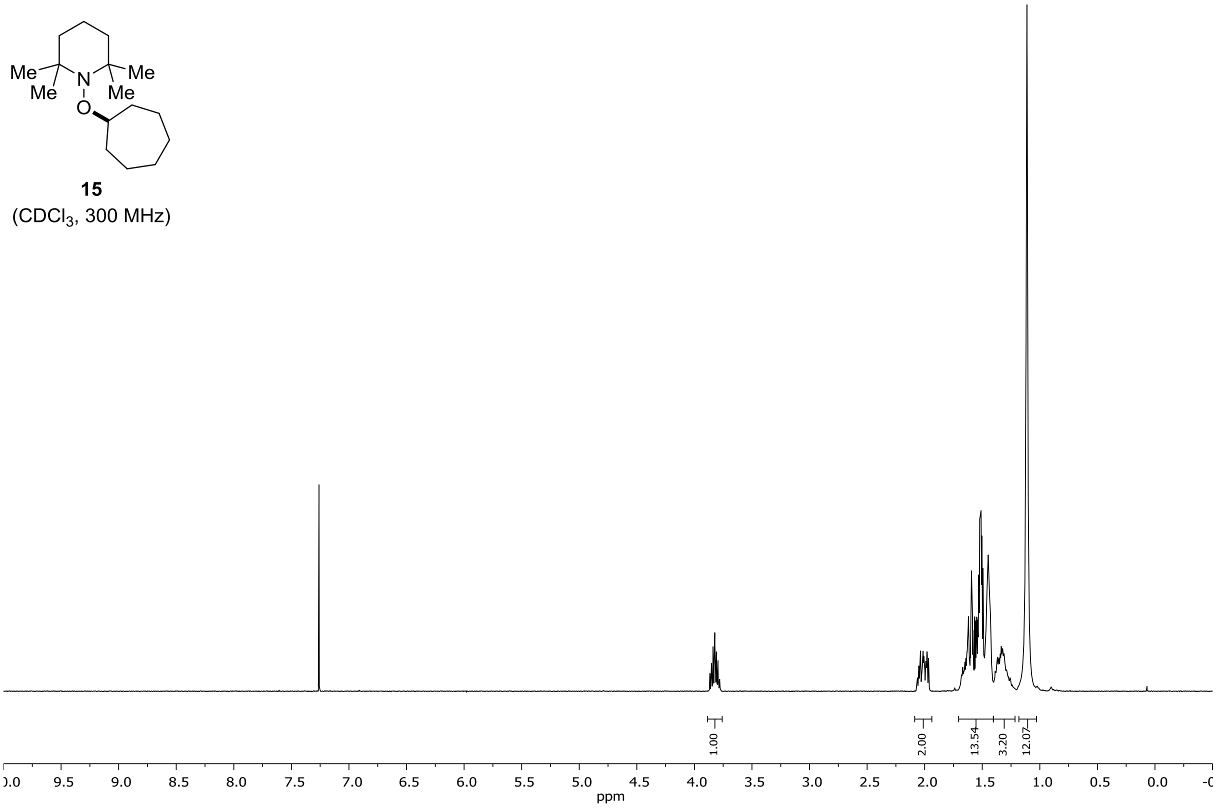

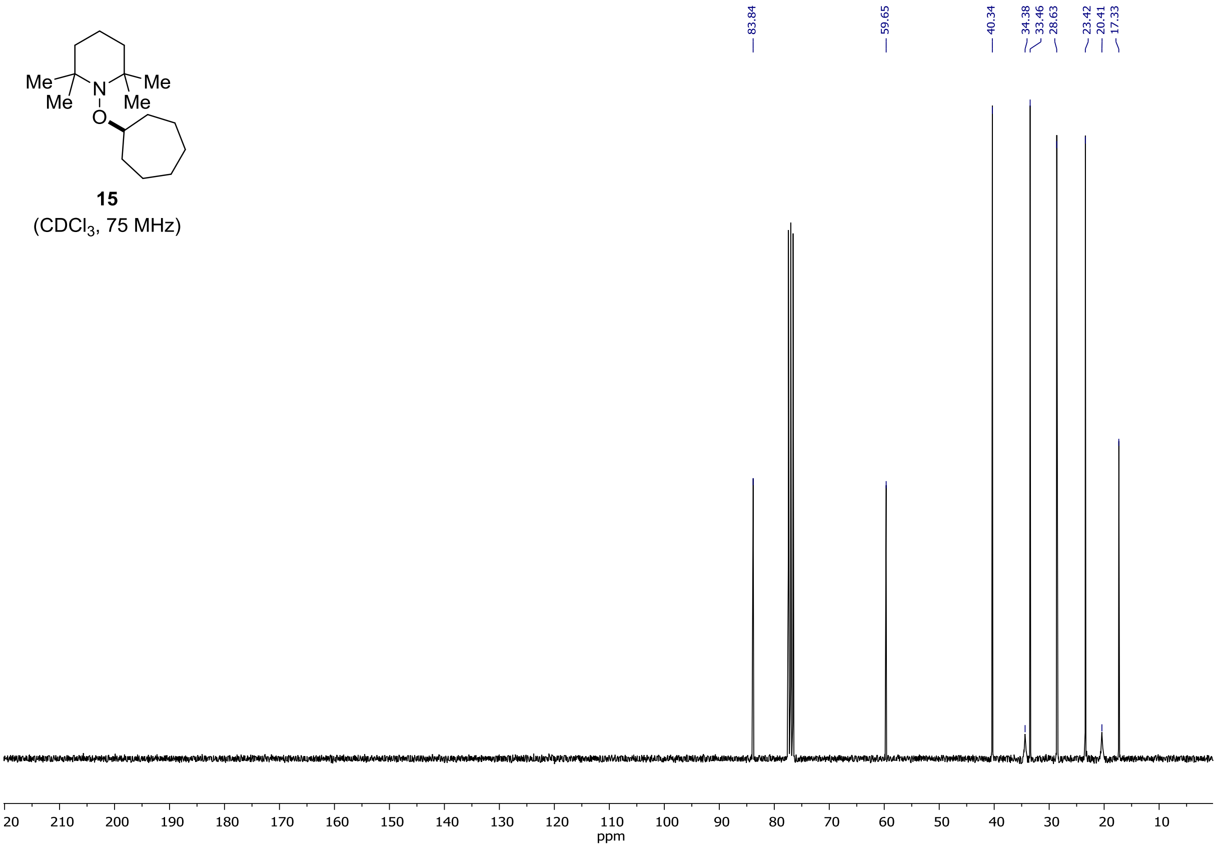

Supplementary Figure 58: ^{1}H and ^{13}C-NMR of Compound 15.

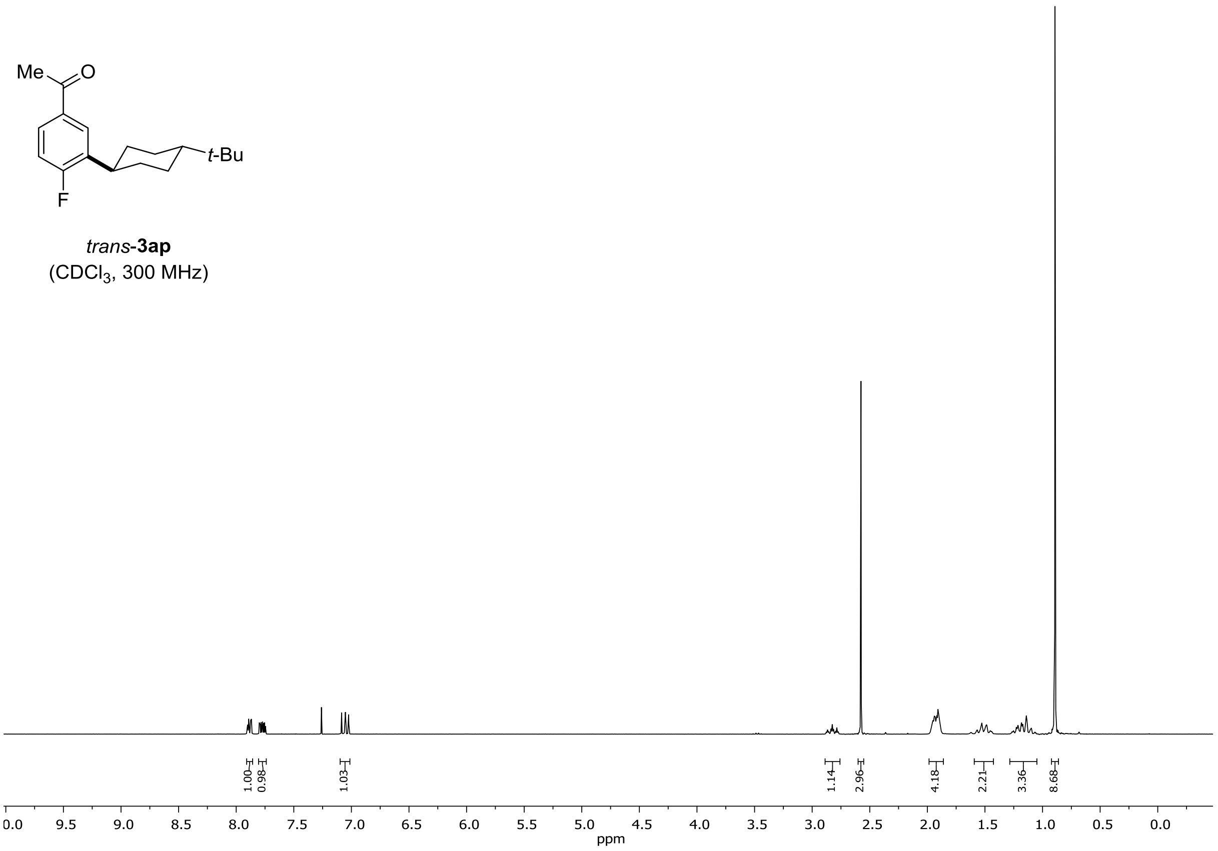

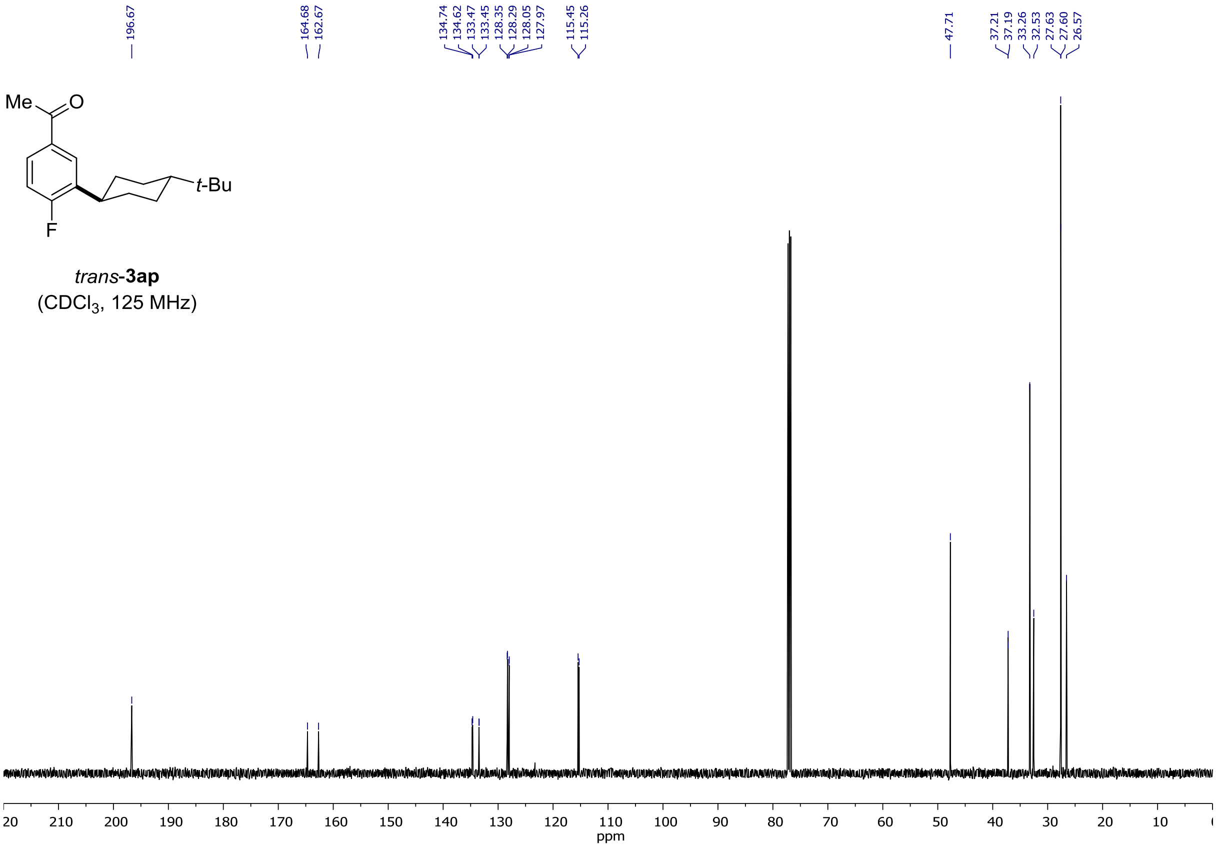

trans-**3ap**
($CDCl_3$, 125 MHz)

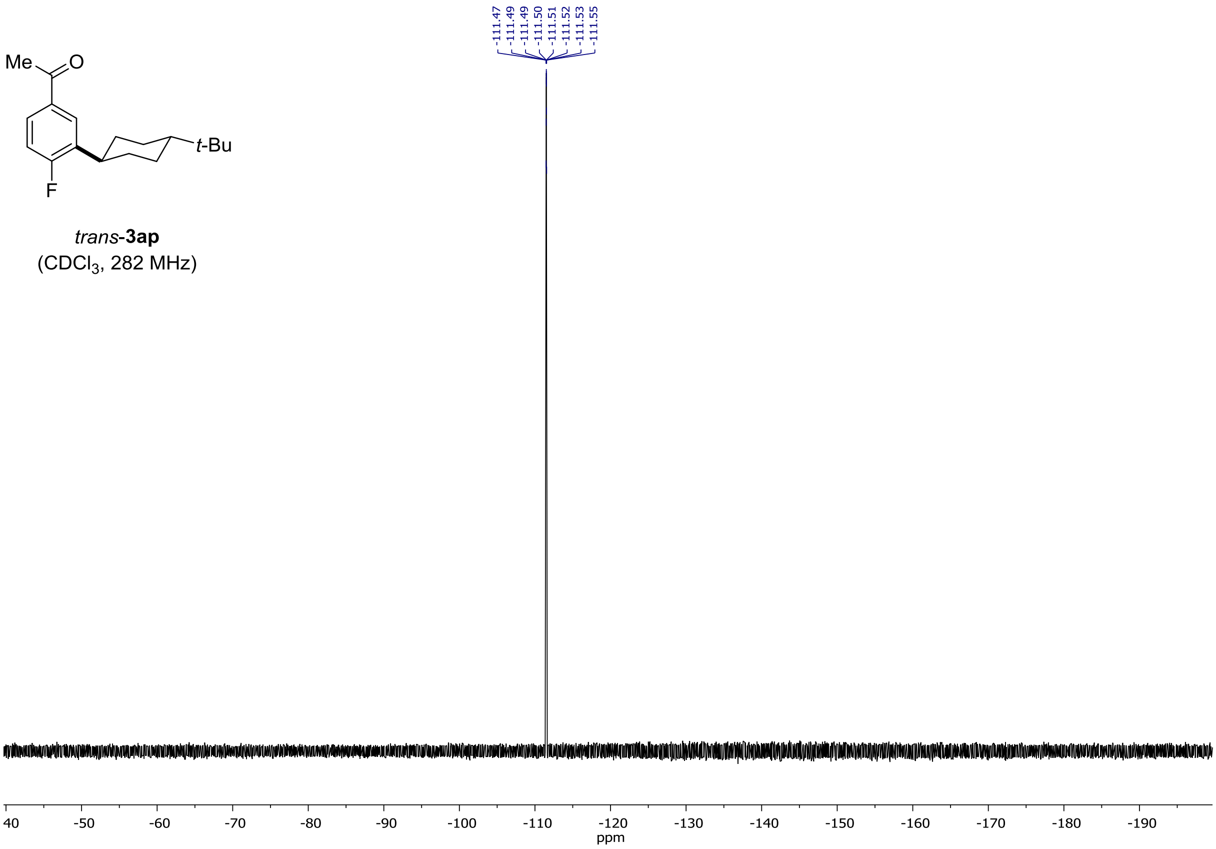

trans-**3ap**
($CDCl_3$, 282 MHz)

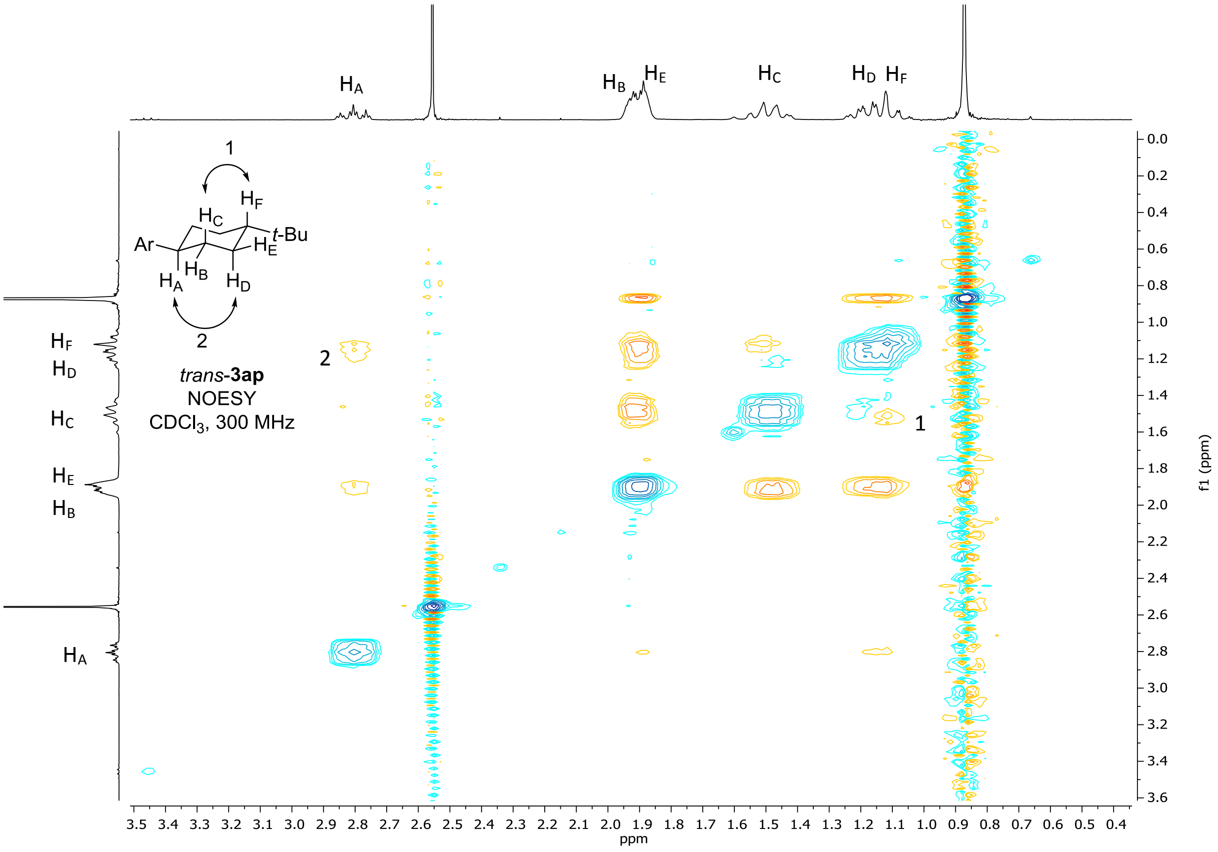

Supplementary Figure 59: ^{1}H, ^{13}C, ^{19}F and NOESY-NMR of Compound *trans*-3ap.

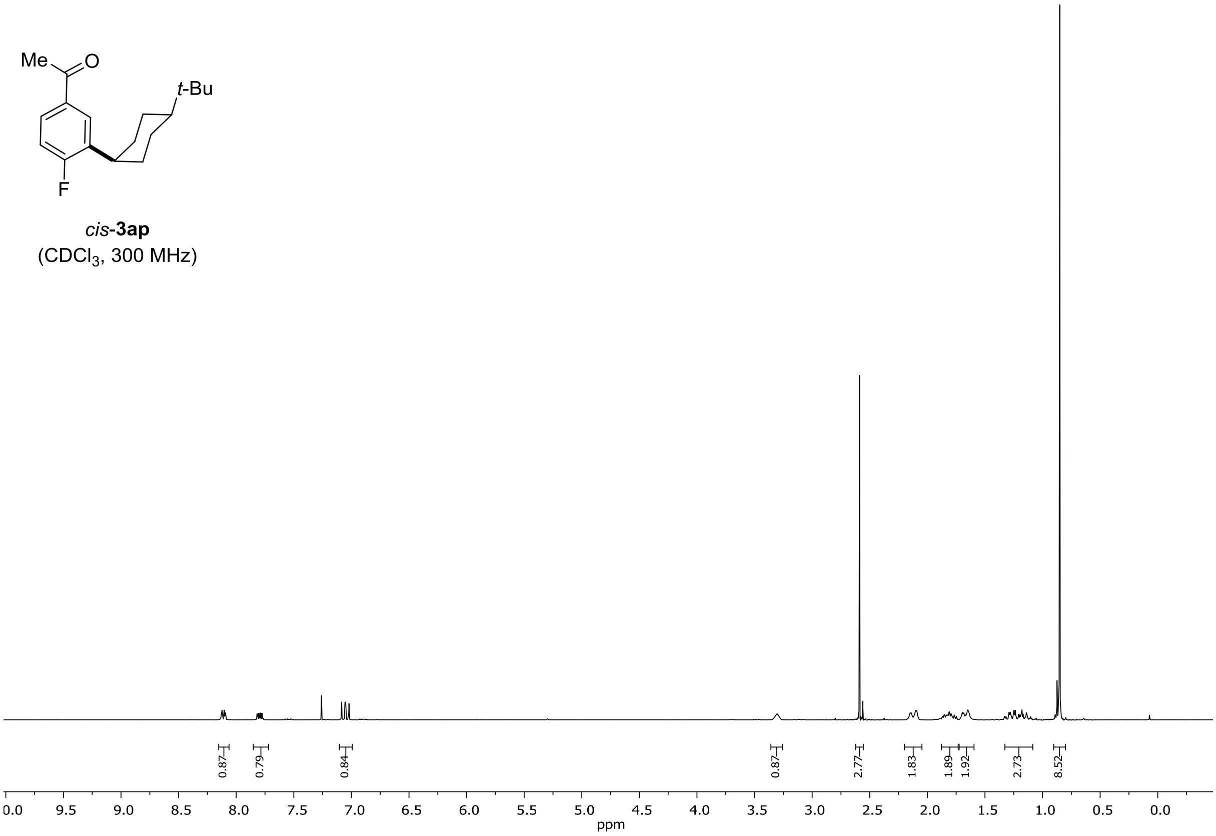

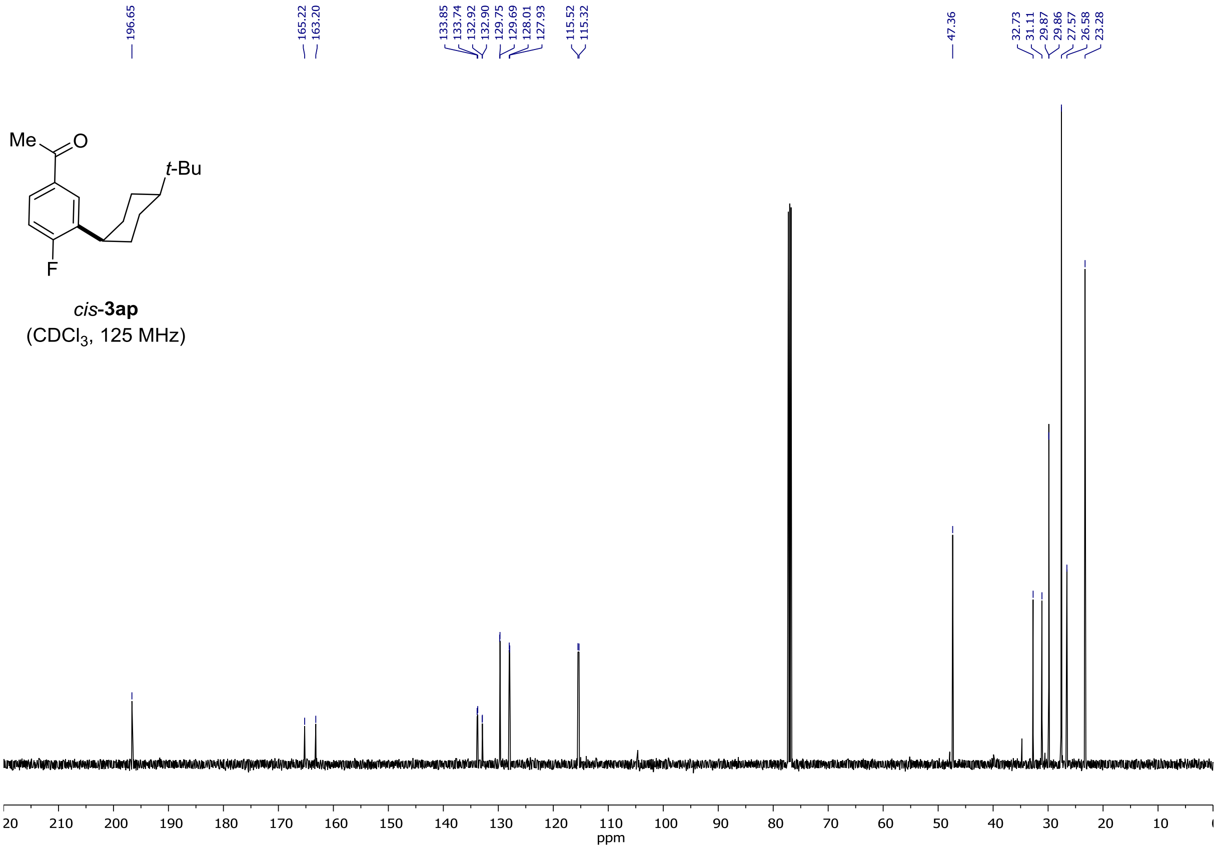

cis-**3ap**
($CDCl_3$, 125 MHz)

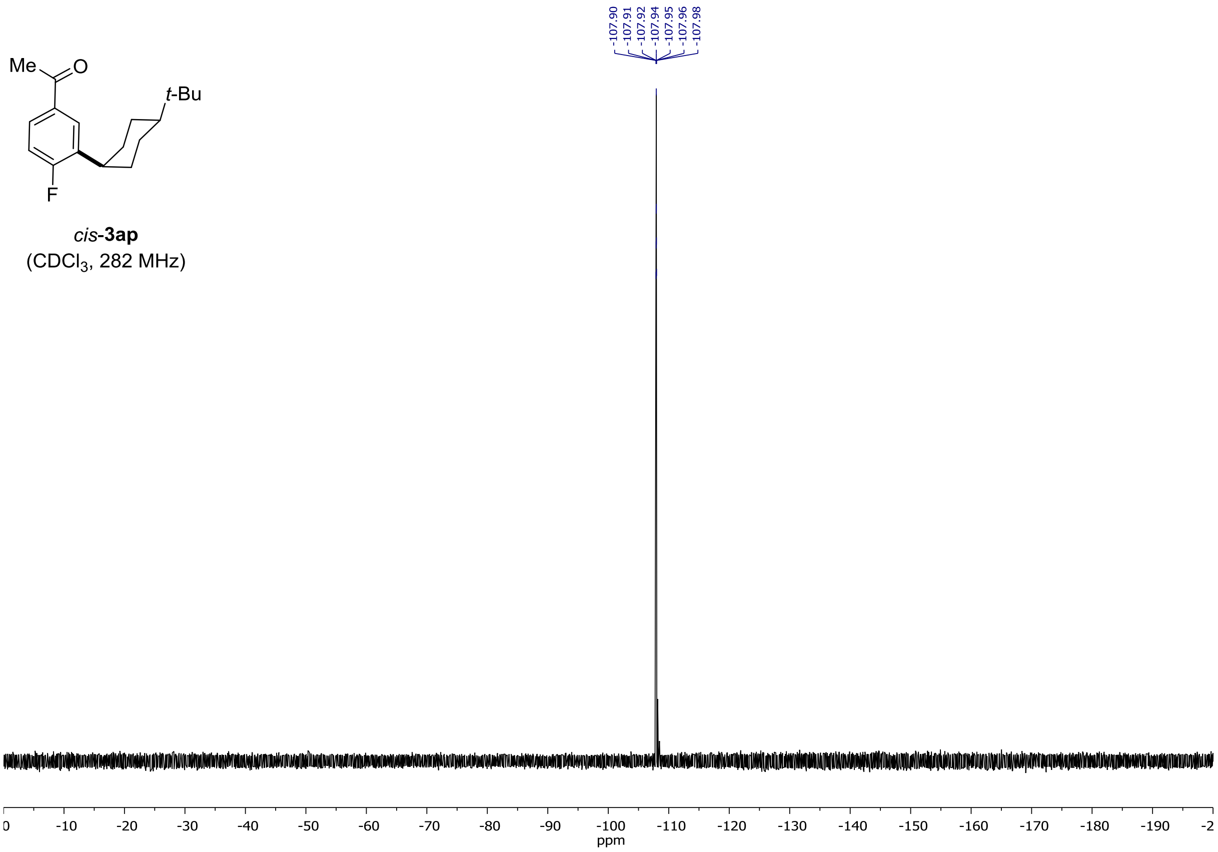

cis-**3ap**
($CDCl_3$, 282 MHz)

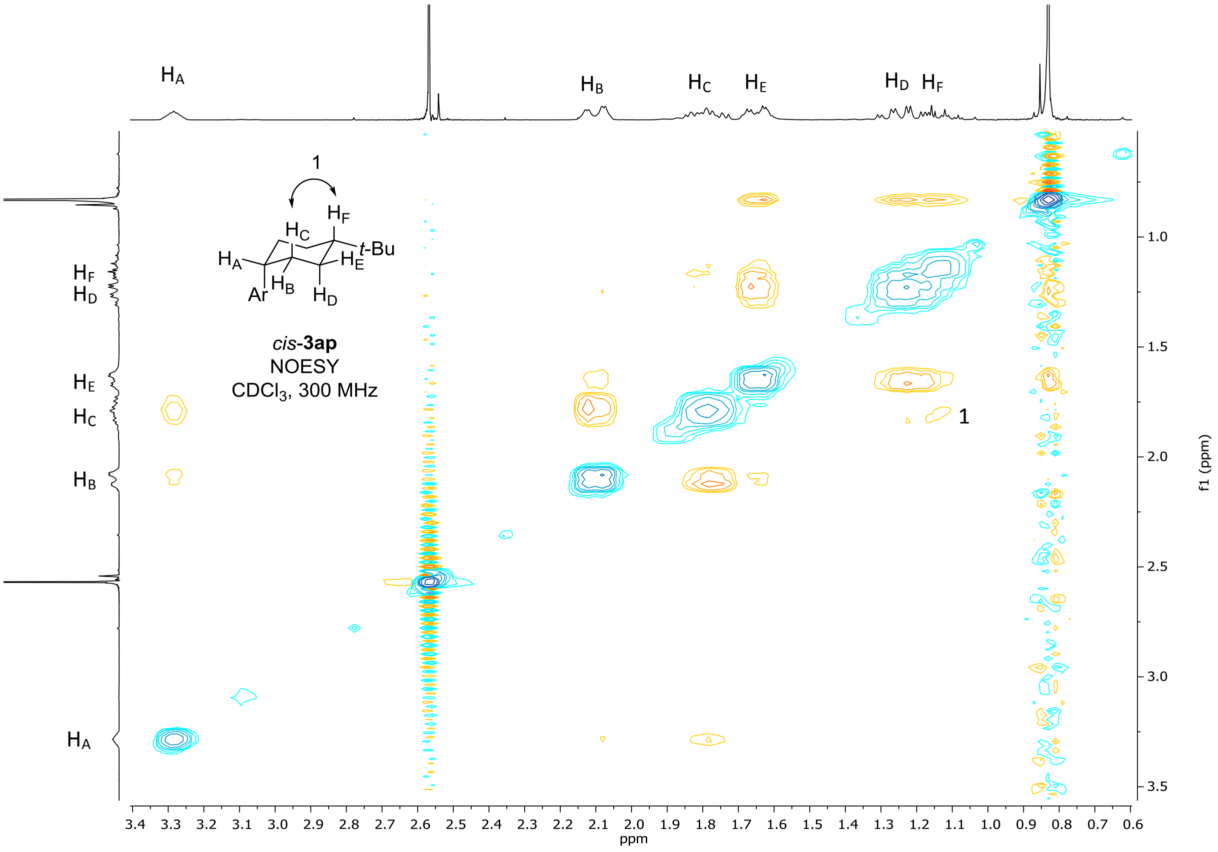

Supplementary Figure 60: ^{1}H, ^{13}C, ^{19}F and NOESY-NMR of Compound *cis*-3ap.
